# Supplementary material for: Detecting the body’s reproductive hormonal brake against tissue overgrowth: Micrin/SgII-70
Source: PLoS One. 2026 Mar 2;21(3):e0340980. doi: 10.1371/journal.pone.0340980 (PMC12952585; doi:10.1371/journal.pone.0340980)
Supplement: S1 File — https://doi.org/10.6084/m9.figshare.27110071.v2. This project outlines the candidate protein and purification techniques utilised. (DOCX) [file pone.0340980.s001.docx]

**Supplementary Information 1 (S1)**

**Candidates & Purifications**

S1 is provided in support of ‘Detecting the body’s reproductive hormonal brake against tissue overgrowth: micrin/SgII-70’ by Hart JE, Davies KG, Mundy CR, Hart AC, Howlett DR & Newton RP (2024). Corresponding author email: [k.davies@herts.ac.uk](mailto:k.davies@herts.ac.uk)

‘Candidate 7500’ is the single name given to multiple peaks in the range m/z 7-8000 in MALDI-TOF MS (the paper’s Figs. 2-6) seen many times during a search for the molecular basis for an organ-mass downregulatory reproductive hormonal activity (Hart, 2014; all references in paper). Bioassay guided fractionations (see later, ‘Physicochemical Purifications’) involved ovarian follicular fluid, ovarian venous plasma and systemic blood plasma and serum mainly from sheep (*Ovis aries*). Anion exchange fractions containing putatively peptidic Candidate 7500 showed antiorganotrophic activity in bioassays in vivo and in vitro in the rat (*Rattus norvegicus*). Edman degradation of Candidate 7500 gel bands yielded seven related N-terminal amino acid sequences (S1 Table 1), with 14-residue ‘EPL001’ canonical, in the form of MKPLTGKVKEFNNI. Synthesized as a peptide, EPL001 proved to be antiorganotrophic and reproductively modulating in vivo, in line with project interests. This, in spite of the EPL001 peptide representing only 20% of the expected ~70 aa predicted for Candidate 7500.

| **SEQ ID NO** | **DESCRIPTION** | **OVINE EDMAN SEQUENCES AND RESIDUE NUMBER** | **PROVENANCE AND OPERATOR COMMENTS** |
| --- | --- | --- | --- |
|  |  | **1 2 3 4 5 6 7 8 9 10 11 12 13 14 15 16 17 18 19 20** |  |
| 1 | First Sighting | M M x V? x P V G? G x F L | Ovarian follicular fluid; spin filters, gel filtration, anionex, SDS-PAGE; ‘Possible sequence large numbers of interesting peaks’ |
| 2 | Second Sighting | M L/K P L T G Q A M E F | Blood plasma; ultrafiltration, 3-30 kDa ‘upstream precipitate’, SDS-PAGE |
| 3 | EPL001 | M K P L T G K V K E F N N I | Blood plasma; upstream precipitate; SDS-PAGE; ‘Relatively clean sequence’ |
| 4 | Beale 4 | x x P x x x x V/L x x F/K N x x | Reference plasma; spin filters, gel filtration, anionex, SDS-PAGE. L & K ‘under signals’ |
| 5 | EPL001 Extension | M K P L T/G K V K x F N N I K/I G F/Y/D x F/V I/V I | Upstream precipitate, aqueous extract; SDS-PAGE; “Co-elution T/G artefact for TG; ‘shadowing latterly’, KGFxVI inferred.” |
| 6 | Harwell 1 | M K V/I T/G Q Y S/V G? K? | Upstream precipitate, tricine gradient gel |
| 7 | Harwell 2 | M N/F P/I/M L N V/A I T/P | Ditto |

**S1 Table 1.** Ovine aa sequences in order of acquisition by automated Edman degradation. Samples analysed at The Babraham Institute, Cambridge, UK (Applied Biosystems Procise, Foster City, CA, US, using ProSorb PVDF cartridges: referenced as Applied Biosystems Procise), except for two purification runs at Harwell Laboratory, South Oxfordshire, UK (‘Harwell’; with Edman sequencing by an Applied Biosystems Procise 491 machine, University of Leicester, Leicester, UK). SEQ ID NO = Sequence Identification Number. Anionex = anion exchange chromatography. ‘Plasma’ = Jugular vein EDTA anticoagulated blood plasma. ‘Upstream precipitate’ and ‘Beale 4’ are explained later under ‘Physicochemical Purifications’.

**Minor Sighting.** S1 Table 1 is reproduced from Hart et al, 2022, which paper includes a detailed description of the seven Edman sequences. Besides these seven, though, there was an eighth minor sequence of potential relevance, derived from anionex FPLC Fraction 10 of ovine late luteal blood serum. The main sequence from a score of Edman cycles (Applied Biosciences Procise) was identified as relating to fibrinopeptide A (discounted Candidate VI: see below). A subsidiary N-terminal undersequence was as follows, in its entirety: MxLKA. This stub is concordant with the second sorting domain of SgII, MLKTGEKPV, with A (alanine) being the N-terminal residue of fibrinopeptide A, note.

A spiralised reading path for the observed Edman sequencing has been supplied previously for EPL001 (Hart et al, 2022, S8 Fig. 1 therein) and is reproduced here as follows.

M L K T G E K P V F K N N I

1 4 7 5 6 10 2 3 8 11 9 12 13 14

-5

+6

-6

+6

+

+1

+1

+1

+2

+4

+2

-2

+1

**S1 Figure 1.** Proposed route of Edman sequencing of sSgII-70 to provide EPL001. The non-contiguity of the C-terminal NNI with the first eleven N-terminal residues is indicated by a dot (•). To obtain EPL001 there are five major moves along the aa chain, towards and away from the C terminus, and eight minor moves, mostly towards the C terminus. A spiral is connoted.

The same concept can be applied to each of the six other available ovine sequences, delivering a spiralised reading path. Below is such a path for Harwell 1, which sequence is of interest as displaying doubleton matches to EPL001, in the form of MK initially & T/G, and four doubleton matches to sSgII-70, in the form of T/G, QY, S/V & G?K?.


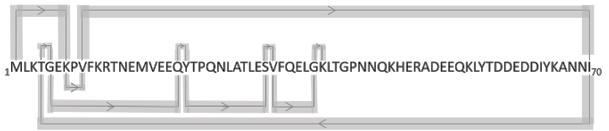


**S1 Figure 2**. Spiralised Edman reading path for Harwell 1 (SEQ ID NO: 6).

In the above linear representation of sSgII-70 the N-terminus and the C-terminus are distant. The proposed molecular reality is adjacency. Edman sequencing provided N-terminal residues from sSgII-70 for all SEQ ID NOS and from its C-terminus, the Ns & Is of SEQ ID NOS: 3-7.

There have been in all 12 candidates for the mammalian inhibitory factor, counting Candidate 7500 as **I** and the EPL001-related Edman sequences of S1 Table 1 collectively as **XI**. Candidates I & XI are analysed in the paper, via decryptions involving MS (S3) and Edman degradation (Hart et al, 2022, S8 therein), to relate to one and the same thing, Candidate **XII**, a novel proteoform of the neuroendocrine prohormone secretogranin II, dubbed sSgII-70.

Eight of Candidates II-X related to sheep material, one to the rat. So, in the course of the physicochemical purification campaign the candidatures of eight ovine proteinaceous entities have been disallowed. The candidate analysis is as follows: **(II)** ‘Haptoglobin-like protein’, c18,500 Da, obtained by gel electrophoresis, with an Edman sequence explored by RT-PCR for identification purposes – an overlarge entity (when a gel band at ~7 kDa was in play: Hart et al, 2017) probably also seen in regard to Tryptic Digest 1, in SDS PAGE, as described in S3, but present in ovarian plasma *and* OVX material, further discrediting its candidacy within a hunt for a *gonadal* hormone; **(III)** ‘Unidentified MS singularity’, *m/z* 17,403 (no match in Protein Data Bank MW database) – discounted as overlarge singularity (i.e. seen only once); **(IV)** ‘Luteal phase entity’, *m/z* 3450 (no clear match in MW database) – RP-HPLC purified form inactive in vitro and the result of an assay in vivo also not supportive of candidature (in retrospect regarded as sSgII-70 N-terminal Grand Fragment: **30mer** match 3453); **(V)** ‘E cadherin-like peptide’ *m/z* c1850 – synthesized and tested negative in vitro and result of assay in vivo not supportive of candidature (notably, pituitary relative weights greater among HPLC fraction recipients than OVX controls and adrenals the same as controls); **(VI)** ‘Fibrinopeptide A’, 1848 Da (seen as *m/z* 1847.25 in S3 Fig. 14b and subject to a definitive 13/14 residues Edman sequencing, with fibrinopeptide B sequence also detected by Edman on another occasion), a degradation product of fibrin – synthesized and found to be contrarily *proliferative* in vitro and *organotrophic* in vivo, the latter at 1mg/kg vs HPLC anionex blank; **(VII)** ‘Albumin fragment’ seen in RP-HPLC purification of ovine ovarian follicular fluid, – contrarily stimulated lymphocyte proliferation in vitro; **(VIII)** ‘Complement fragment’, identified bioinformatically from Edman data derived from sheep serum gel bands; **(IX)** ‘Androglobin’, tryptic peptide fragment of *m/z* 827.5 seen in LC-MS/MS analysis of trypsinised anion exchange fraction of ovine ovarian follicular fluid, Tryptic Digest 12 in S3, having a 6 of 7 residues correspondence to the database sequence of the relevant gonadal entity: **FDLFSA**K.

A later non-ovine possibility, Candidate **X**, was NXPE family member 4 (Q5XI89, formerly FAM55D). This arose as a weak bioinformatics hit when an aqueous extract of rat hypothalamus was subjected to immunoaffinity column purification using an anti-EPL001 antibody (Hart et al, 2017; Hart et al, 2022, S1 therein). The predicted pI of Candidate X, at 9.2, vitiated its candidature in a search for a factor that has acidic characteristics in anion exchange chromatography.

**Immunoprecipitation (IP)**

An effort was made to identify the endogenous antigen of the antibody raised against the exogenous antigen EPL001. In an IP/LC-MS purification the G530 anti-EPL001 goat antibody was bound covalently to resin beads, with control preparations preabsorbed with EPL001 (Hart et al, 2017). Embryos of *Drosophila* were used that were (i) frozen (i.e. formalin unfixed) or (ii) formalin fixed and subjected to antigen retrieval using RIPA buffer, as a basis for preparing aqueous extracts. Similar extracts were prepared using rat hypothalami in three categories: (i) frozen (i.e. formalin unfixed), (ii) formalin fixed and subjected to antigen retrieval using RIPA buffer and (iii) formalin fixed and subjected to antigen retrieval using citrate buffer. Elution samples were acyrylamide gel-purified and the gel slice subjected to in-gel tryptic digestion. The resulting tryptic peptides were fractionated using an HPLC system in line with an LTQ-Orbitrap Velos mass spectrometer (Thermo Scientific). Tandem MS data were analysed by the machine’s software and searched against the UniProt database using the SEQUEST algorithm. Briefly, control results were as follows: fly unfixed, 700 protein matches; fly fixed, 634; rat unfixed, 1634; rat fixed RIPA, 226; rat fixed citrate, 438. Test matches were thus: fly unfixed, 1129 (including 526 test specifics, i.e. items having a zero control score), fly fixed RIPA, 71 (30); rat unfixed, 1681 (424); rat fixed RIPA 406 (213), rat fixed citrate 454 (114). The fixed and unfixed proteomes comprised different populations of proteins in the fly and rat, the fixed test specifics in both species being markedly fewer in number. The ideal was that controls, using EPL001-blocked antibody beads, provided no protein matches while the test groups for each species provided just one, the endogenous antigen. Although the results were wildly at variance with the ideal, the procedure nonetheless delivered sole candidates. The full primary sequences from UniProt of the 30 fly fixed test specifics were searched for partial epitope matches to the EPL001 C terminus. There were 11 hits, none present in fly unfixed test material (Hart et al, 2017, Table 1 therein). Among this group was Q9W2X8, which was deemed to be the sought-for antigen from the fly IHC (op. cit., p21). Tellingly, the timing of gene expression (Flybase) matched the timing of staining during embryonic development. Among rat fixed test specifics there were 7 ‘overlappers’, i.e. present in both RIPA and citrate groups, with partial epitopes (op. cit., Table 2 therein). There were 42 other matches with partial epitopes present only in one antigen retrieval system or the other. There was no good MW match for the sought-for 7 kDa entity. The sole secreted item in the 7 overlappers was ‘SgIIvar’, Q8CGL8, a half-sized splice variant of full-length rSgII, P10362. This was deemed relevant to the hypothalamic IHC antigen on multiple grounds (op. cit., 25-27), including its matching, at 37.1 kDa, a second sought-for western MW of 35-45 kDa from rat hypothalamus anion exchange chromatography. This band, like those around 7 kDa (op. cit. Fig. 10 therein), had been preabsorbable with the EPL001 C-terminus KVKEFNNI (op. cit., Fig 9 therein). The rSgII proteoform and the fly lead candidate Q9W2X8 display the same partial epitope (NNI) and a version of the rat protein’s MS ID peptide is present in the fly protein. A version of Q9W2X8’s N-terminally located MS ID peptide is lacking from the N-terminally truncated 322-residue rSgII proteoform but is present in the 619-residue full-length rSgII, in the same relative position. Fly Q9W2X8 shows detailed SgII-relevant sequence homology, notably in regard to sorting domains. Candidate XII for the mammalian factor was expressed as ‘likely to be secretogranin II related’ (op. cit.).


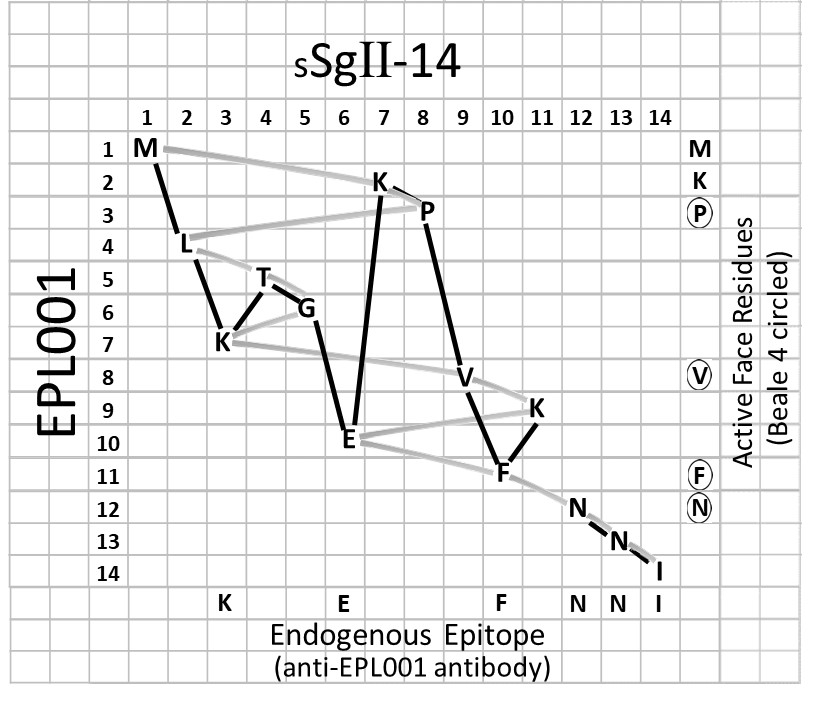

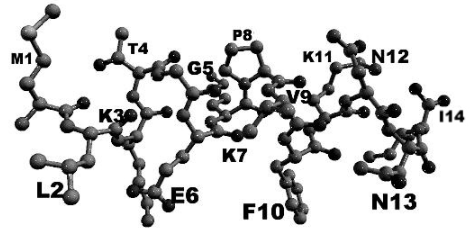

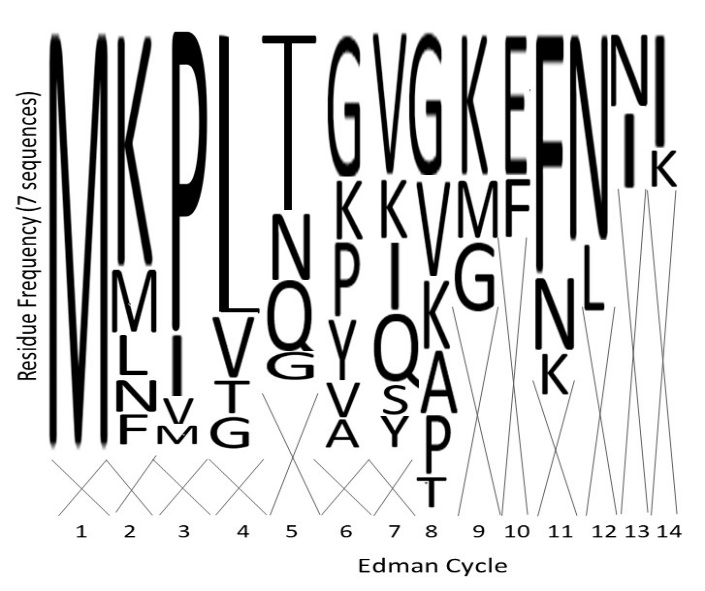


**S1 Figure 3.** 14x14 ‘magic grid’ of sSgII-14 versus EPL001. Following the solid line yields the aa sequence of sSgII-14 across the grid, column by column. The shaded track reads out EPL001 downwards, row by row. EPL001 is interpreted as an Edman misread of sSgII-70, a hormonal proteoform of sSgII. For lysine grid placements see Hart et al, 2022 (S2 therein). The logo plot, lower right, is of seven available ovine N-terminal aa sequences, six of which start with M (S1 Table 1). Appearing across the top of the logo plot are the proposed active face residues – in the form **MKP**xxx**V**xxx**FN**xx – which potentially account for EPL001’s bioactivity. Lower left is a minimized structural prediction (omitting hydrogens) for EPL143, which is sSgII-14 in contiguous form. The model takes the same general form as the grid path for sSgII-14, suggesting that the 14x14 grid overall is structurally predictive. (S1 Fig. 3 is reproduced from Hart et al, 2022, with augmented grid axis labels.)

**Physicochemical Purifications**

Factor purification involved three main physicochemical methods. In chronological order and named for their development locations within the UK, these were:

Babraham Method (Babraham Institute, Cambridge)

Harwell Method (Harwell Laboratory, South Oxfordshire)

Sheffield Method (University of Sheffield, South Yorkshire)

The Babraham Method yielded MS candidate 7500 and, from of an approximately 7.5 kDa polypeptide (SDS PAGE), a partial N-terminal sequence, the Beale 4 (see later, Sheffield Method). The Sheffield Method gave rise to a full 14-residue N-terminus, EPL001, which was consonant with the Beale 4, in material containing MS Candidate 7500. The Harwell Method provided tryptic digest evidence that EPL001 relates to a secretogranin II proteoform, sSgII-70 (S3: Tryptic Digest 1).

**Babraham Method**

This method was used to purify inhibitory factor mainly from ovine materials (Hart, 1999), providing for example the paper’s Fig. 1 rat organometric exemplification (S2). The purification is based on membrane centrifugation for separation and concentration, together with size exclusion and anion exchange chromatography for final purification. The following is a schematic representation of the essential steps:

**Start** with 120 ml of feedstock (e.g. ovarian follicular fluid, ovarian venous plasma); store at -70°C; thaw rapidly

ↆ

Spin filtration (Centriprep 30, 30 kDa cut-off) to give approximately 80 ml 0-30 kDa filtrate

ↆ

Separation/concentraton of filtrate by spin filtration (Centriprep 10 then Centricon 10, 10 kDa cut-off) to give approximately 200 µl 10-30 kDa concentrate

ↆ

Concentrate loaded on Superdex75 size-calibrated gel filtration column equilibrated in phosphate buffered saline (PBS)

ↆ

10-20 kDa fractions pooled and concentrated to 1.0 ml by spin filtration (Centriprep 3, 3 kDa cut-off)

ↆ

Dilution and spin filtration (Centriprep 3 then Centricon 3) for buffer exchange into 20 mM Tris pH 7.5 and further concentration to approximately 200 µl

ↆ

100 µl aliquots of the dialysed concentrate adsorbed onto Mono Q anion exchange FPLC or HPLC columns. Elution of bound material using salt gradient.

ↆ

Fractions diluted with PBS for bioassays in vivo and in vitro (with UV absorbance peak monitoring at 214 nm), with analysis by SDS PAGE and MALDI-TOF MS

To reduce the risk of bacterial contamination, samples were initially spun down in a cold room to bring down cellular and other debris. The spin filter cut-off in the purification procedure would have excluded bacteria. Additionally, prior to chromatography all samples and buffers were sterilised using low-micron filters. No sodium azide was added as an antimicrobial agent at any stage because this would have compromised bioassays in vivo and damaged chromatography pumps. The majority of the of candidates described in this paper were identified in the course of the Babraham work. There were no bacterial hits in the database searches relating to Babraham material.

Below is one account from Babraham lab notes of the preparation of ovine, bovine and porcine ovarian follicular fluid for Mono-Q FPLC:


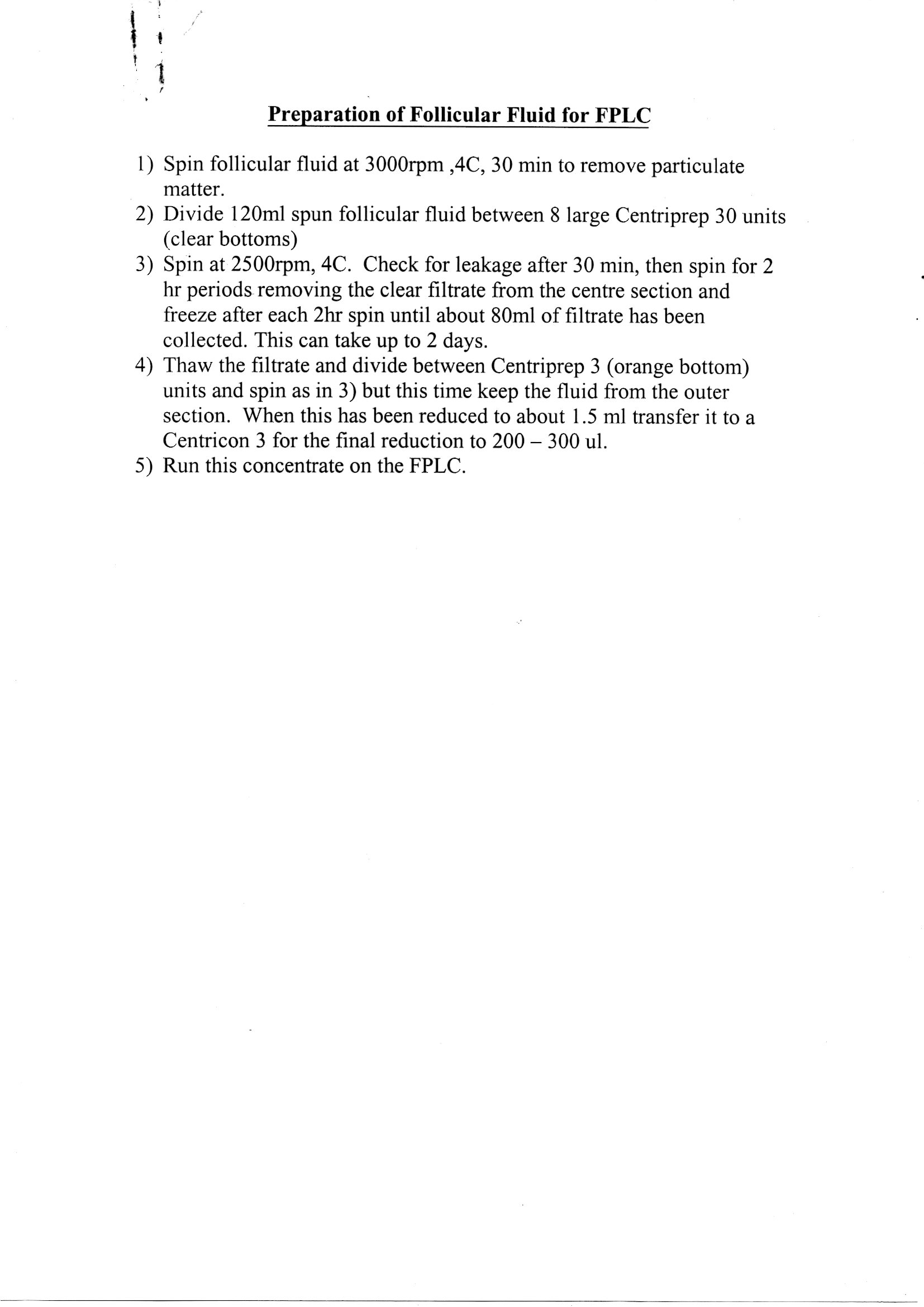


The germinal investigations at Babraham suggested that micrin activity in vivo (organ shrinkage) was confined to the 0.1-0.2 M NaCl anion exchange eluate of the 10-20 kDa fraction (Hart, 1999). No optical density was registered by column UV detector in the region of activity and no bands were visualisable in SDS-PAGE. An anion exchange gradient of 0-1M NaCl was then used, an expansion on the prior 0.1-0.3 M NaCl gradient. Inhibitory activity in the BMC viability assay in vitro elutes ‘early’ and ‘late’, at approximately 0.2 M and below 1.0 M, providing a ‘double dip’ histogram. MALDI-TOF MS showed c7500 peaks present in both fractions, which were independently active in the rat organometric assay in vivo. A later scale-up showed early and late visualisations when an anti-EPL001 antibody was used in Western blots, as in the following graphic, tying together Candidate 7500 with the EPL001 N-terminal sequence.

**S1 Figure 4.** ~7 kDa bands seen in Western blotting with goat anti-EPL001 antiserum (G530) of sheep serum subject to anion exchange chromatography, in low salt and high salt fractions. ‘200’ = 200 mM NaCl, ‘800’ = 800 mM NaCl. (Reproduced from Hart et al, 2017, Supplementary Information therein.)

Anionex late-eluting fractions were subject to reverse phase chromatography, as described in the paper (see ‘Candidate 7500) and depicted in regard to MS data in a supplementary file (S3 Fig. 3). Once again Candidate 7500 was bimodally distributed in early- and late-eluting fractions, with a corresponding double dip histogram in terms of BMC viability.

The eluted anionex c7500 moiety at 1.0M was speculated to represent denatured material, yet it was still active. The early material then disappeared for unknown reasons. Bioactivity in vitro was subsequently detected reliably in late-eluting anion exchange fractions, as illustrated by the following graphic.

**S1 Figure 5.** Effect of ovine serum derived anion exchange fractions on BMSC viability in vitro. Alamar blue was used to assess cell viability. The majority of the activity in this purification run was found in Fractions 19-21, with almost total loss of cell viability. (Note that although peaks of activity are associated with different fraction numbers in this account, this was due to the use of different chromatographic conditions. In the phase of the purification campaign when late-eluting bioactivity came to the fore in anion exchange chromatography, the peaks still corresponded to somewhat less than 1 M NaCl.)

f1

f2

f3

f4

f5

f6

f7

f8

f9

f10

f11

f12

f13

f14

f15

f16

f17

f18

f19

f20

f21

f22

f23

0

20

40

60

80

100

120

f1

f2

f3

f4

f5

f6

f7

f8

f9

f10

f11

f12

f13

f14

f15

f16

f17

f18

f19

f21

f22

f23

**Fraction Number**

**% of Control**

It was found that EDTA plasma exhibited more bioactivity in vitro than heparinised plasma or serum (see Sheffield Method).

Anion exchange Fraction 29 (of 32) of sheep EDTA plasma, active in vitro, disclosed material at *m/z* ~7800 in MALDI-TOF MS (see the paper’s Fig. 3). This material was concentrated and run on SDS-PAGE for analysis. As nothing was found it was dried down, run again and blotted. Although no eluted bands were visible, the region around 7 kDa was cut out anyway and Edman sequencing attempted. This gave a weak result, SEQ ID NO: 4 of S1 Table 1, the Beale 4 (see Sheffield Method).

**Harwell Method**

This method was developed as an alternative to the Babraham Method, with the aim of avoiding membrane steps, to reduce potential losses. An early focus was isolation of 10-20 kDa ovine material. An exemplification of the Harwell Method is given elsewhere (S3: Tryptic Digest 1). There follows a schematic representation of the essential steps.

Start with 30 ml ovarian venous plasma pool

ↆ

Apply plasma pool to size-calibrated Superdex200 column (mobile phase 50 mM Tris/HCl pH 7.5)

ↆ

Collect fractions covering MW range 10-20 kDa (volume approximately 20 ml)

ↆ

Load 20 ml on Mono Q anion exchange FPLC and elute with salt gradient

ↆ

Fractions diluted with PBS for bioassays in vivo and in vitro (with UV absorbance peak monitoring at 214 nm), with analysis by SDS PAGE and MALDI-TOF MS

Note that the two Harwell candidates in S1 Table 1, SEQ ID NOs: 6 & 7, were obtained using upstream precipitate derived via the Sheffield Method, with gel electrophoresis at Harwell.

The handover from Babraham to Harwell was effected using a bioactive Sheffield Reference Plasma (ovine) subjected to MALDI-TOF MS at both locations.

**Sheffield Method**

There follows a verbatim quotation (‘…’) from Hart et al, 2022, S1 File, Sequencing & Purification [with interpolations relevant to the present paper in square brackets].

‘A simplified purification procedure [Sheffield Method], for scale up and to reduce losses, involved ultrafiltration of sheep jugular vein plasma (up to 1.5 L, EDTA anticoagulated, follicular phase of oestrous cycle: Hart, 2000) through a 30 kDa Vivaflow tangential flow membrane. The filtrate was concentrated over a 3 kDa Millipore stirred cell ultrafiltration membrane, with the retentate (~25 ml of 3-30 kDa concentrate) separated using a Mono Q 10/10 anion exchange column in HPLC. Candidate 7500 was identified in fractions active in vitro but in insufficient quantities for sequence determination. Serendipity asserted itself, with the appearance of a precipitate on the 3 kDa filter [Hart, 2008]. This, like the supernatant and anion exchange fractions thereof, proved to be inhibitory in vitro. The ‘upstream precipitate’ was submitted to MALDI-TOF MS (Harwell). Bizarrely, all that flew from this variably discoloured multi-milligram apparent mixture was Candidate 7500. (The same was true when the upstream precipitate from bovine ovarian follicular fluid was subject to MS.) [See the paper’s Fig. 4] A sample of the upstream precipitate was separated by SDS-PAGE on a gradient gel under reducing conditions giving a large number of bands of which the lowest defined was ~7 kDa. This was designated Band 1 and yielded MALDI-TOF peaks of 7.5-8 kDa (personal communication, Pat Barker, Babraham). Trypsinisation for possible database identification resulted in a set of peptides but no identification. [This is S3’s Tryptic Digest 10, within which mass spectrum are described potential predicted tryptic fragments of sSgII-70.] Edman sequencing of Band 1 gave a 14-residue N-terminal sequence deemed ‘relatively clean’: MKPLTGKVKEFNNI [S1 Table 1, SEQ ID NO: 3, EPL001]. The match with the sequence obtained from the [Babraham Method] anion exchange fraction of plasma is xxPxxxxVxxFNxx, privileging V & F at positions 8 & 11, respectively. The residues **P∙V∙FN** were designated the Beale 4 [SEQ ID NO: 4 in S1 Table 1], after the scientist responsible for purification (Dennis Beale, Babraham). [The mass spectrum from the Beale 4 material is the paper’s Fig. 3].’

Although other initial purification methodologies were investigated, including ammonium sulphate and PEG precipitation and various solid phase extraction columns (cation exchange, anion exchange, C18 reverse phase and normal phase), these did not offer significant advantages over Babraham-style ultrafiltration. In these studies the BMC assay was used to monitor activity.

Ovine blood serum was used initially as a starting material for the purification of micrin as it was thought that the clotting process would remove much of the protein that would interfere with the subsequent purification process. It was found however that the cytotoxic activity in the serum as evinced in the BMC cell viability assay was apparently not stable, with a number of batches having no activity whatsoever and where activity was present, often by the second assay this too was lost. It was thought possible that this may be due to enzymatic degradation during the clotting process, which may be avoided by the use of plasma. On studying the effects of serum, heparinised plasma and EDTA plasma from a variety of donor ewes it was found that plasma from blood anticoagulated with EDTA (at 1mg/ml of blood) was significantly more active than serum or heparin plasma (S1 Fig. 6). From this point on EDTA plasma was used as the starting material (Hart, 2000). Inherent differences between serum and plasma are less likely to be at issue here, than the activity of EDTA itself, which is known to be an effective inhibitor of proteolytic enzymes, by mopping up calcium and other divalent ions. This bolsters the evidence that micrin is proteinaceous.

**S1 Figure 6.** Effect of jugular vein serum and plasma from four ewes [codes in box] on BMC survival in vitro. It was found that EDTA plasma was significantly more active than either heparinised plasma or serum.

Purification runs were carried out using either ovine jugular vein EDTA plasma (e.g. 1.5 L; Hart, 2008) or ovine or bovine ovarian follicular fluid (e.g. 120 ml, without EDTA), according to the methodology described above, which can be summarised as follows:

1. Ultrafiltration of start material through a 30 kDa Vivaflow (Sartorius) tangential flow ultrafiltration membrane.
2. Concentration of the filtrate over a 3 kDa Millipore (Merck) stirred cell ultrafiltration membrane.
3. The 3 kDa retentate was separated using a Mono Q 10/10 ion exchange column (Sigma-Aldrich) in HPLC. Buffer A, 20 mM Tris, pH 7; Buffer B, 20 mM Tris pH 7 containing 1 M NaCl. The gradient was 0 – 100 % B over 60 mins, 100% B until 70 mins, 100% - 0% B until 80 mins. 4 ml/min

All runs produced a similar pattern to those with the use of the Babraham Method, in that little or no activity was eluted during the salt gradient followed by a ‘peak’ of activity starting at around 0.8 M NaCl. This pattern was evident regardless of the source of material, blood plasma or ovarian follicular fluid.

**S1 Figure 7.** Human MG63 osteosarcoma cell viability in vitro. Anion exchange chromatography of 3-30 kDa fraction of bovine ovarian follicular fluid (120 ml or more of starting material, abattoir derived). Activity elutes at around 0.8 M NaCl.

To test the role of salt, a dummy run lacking starting material used the standard buffer without a column. Across the fractions there was no decrease in M63 cell viability. The effect was real.

The issue of early eluting versus late eluting Candidate 7500 in anionex is addressable by consideration of an experiment described fully in a separate supplementary file (see S3 Fig. 8 legend). Ovine jugular vein EDTA plasma was subject to ultrafiltration by the Sheffield Method, followed by HPLC anionex. The resulting samples were then desalted using a C18 Sep-Pak cartridge with 0.1% aq. TFA. The cartridge was first eluted with 60% isopropanol (to elute peptides present) and then with chloroform (to elute lipopeptide and other lipid material). The samples were then subject to MALDI-TOF MS, which revealed an array of putative Candidate 7500 grand fragments in the *m/z* 3000s and nothing else of note, except discounted Candidate VI, fibrinopeptide A.

| Fraction pools | Isopropanol | | Chloroform | |
| --- | --- | --- | --- | --- |
|  | Sinapinic acid | CHCA | Sinapinic acid | CHCA |
| 1-5 | - | - | 3429 (31)  3272 (29) | 3351 (30)  3194 (28) |
| 6-10 | 3272 (29)  3196 (28)  3294 (29) | - | 3274 (29) | 3195 (28)  3178 (28)  3161 (28) |
| 61-65 | 3274 (29)  3327 (30) | 3195 (28)  3179 (28)  3160 (28) | - | 3196 (28)  3179 (28)  3161 (28) |
| 66-70 | 3271 (29)  3293 (29) | 3195 (28)  3160 (28) | - | - |

**S1 Table 2.** Peaks in the *m/z* 3000s seen in MALDI-MS when HPLC anionex fractions of ovine systemic blood plasma were investigated (with sSgII mer matches in brackets, as per S3 Table 1). (This table is also provided as S3 Table 5, with detailed peak analysis).

These *m/z* 3000 ‘grand fragments’ are analysed in the paper to arise from the pure-form lability of Candidate 7500, with disintegration during processing. They are not themselves MS artefacts. But when subjected to MALDI-TOF MS the grand fragments themselves show MS artefactuality. This is evident because the 3000 ions find matches, mostly tight, in S1 Table 2, dually decremented in accordance with the Candidate 7500 model of C-terminal residue losses and water reductions. It is notable that none of the 3000 items finds a match on the first predictive data column of S1 Table 2, as sSgII-70 lacking crosslinks. It is unexpected that ‘grand fragments’ of Candidate 7500 are analysable in the same way as the *m/z* 7-8000 ions. Let these 3000 items stand for native Candidate 7500, then *bioactive late eluting material* was mostly released from the column by isopropanol, while *inactive early eluting materia*l mostly came off afterwards, in the lipid solvent chloroform. Denaturation of the Candidate 7500 early material into inactivity is probably indicated, with retention of denaturation in the early eluting 3000 grand fragments. Why this denaturation occurred in subsequent purification runs and not in the forerunner preparations, when early eluting material was bioactive as well, is unknown. A procedural alteration can be guessed at.

Fully scaled-up purification runs typically involved 1.5 L of ovine jugular vein EDTA plasma reduced to about 25 ml of 3-30 kDa concentrate. During the final concentration over the 3 kDa filter it was found that a precipitate was formed, the ‘upstream precipitate’ of S1 Table 1. This was in large (mg) quantities and off-white to yellow or brown (hence the paper’s Fig. 4 heading). When tested, the precipitate was found to have similar activity to that of the supernatant in MG63 osteosarcoma cell viability assays, which had superseded the primary culture BMC assay as being more reproducible.

**S1 Figure 8.** Effect of mixtures of supernatant and precipitate formed during concentration of 3-30 kD fraction on MG63 cell viability.

Furthermore, MALDI-TOF MS analysis of the precipitate revealed only two major peaks at ~7.5 kDa. The paper’s Fig. 4 shows spectra for upstream precipitate from bovine ovarian follicular fluid, top panel, and ovine jugular vein plasma, lower panel. These disparate materials yielded strangely similar twin-peak Candidate 7500 mass spectra. This matter will be considered again later.

A bacterial provenance was suspected for the precipitate. The inclusion of 0.1% sodium azide during the process greatly reduced the amount formed. A white precipitate had been observed on a 3 kDa membrane filter during a prior purification campaign at Harwell in which bacterial contamination had not been an issue.

The following is a quotation (**‘**…**’)** from a lab report [with interpolations relevant to the present paper in square brackets]:

**‘Further improvements in initial stages of purification.** A new ultrafiltration apparatus was tried out which allowed a considerable increase in throughput over the original apparatus. In this improved process the material was: a) filtered through muslin to remove particulate matter, b) filtered through a 0.45 μ filter to remove bacterial/fungal contamination, c) filtered through a 30 kDa tangential flow ultrafiltration apparatus until 90% of the material had passed through and d) the filtrate concentrated over a 3 kDa ultrafiltration membrane in a stirred cell until 10-15 ml remained [a 100-fold concentration]. At this point a precipitate formed which was separated from the supernatant [actively inhibitory in the BMSC assay in vitro] by centrifugation at 3000 rpm for 30 min. After reconstitution in culture medium it was found that the precipitate [resuspended in BMSC culture medium] was biologically active and after Mass. Spec. analysis was found to be almost homogeneous giving 2 peaks at Mol. Wt. 7626 and 7709 [the paper’s Fig. 4 shows a similar result].**’**

A sample of ovine upstream precipitate was run out by SDS-PAGE on a gradient gel under reducing conditions. This produced a large number of bands, the lowest defined being just above 6 kDa.


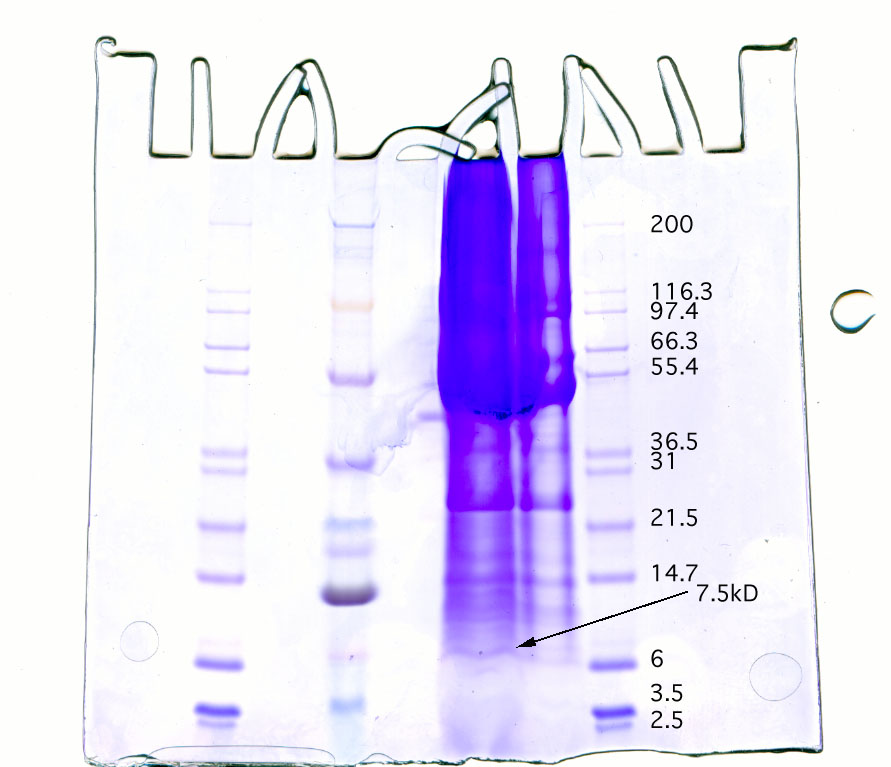


**S1 Figure 9.** SDS-PAGE of upstream precipitate.

This band was cut from the gel and trypsinised, followed by MALDI-TOF MS for possible database identification. While a set of peptides was obtained, no database hits were identified. (This is Tryptic Digest 10 of S4, containing potential matches to predicted tryptic fragments of sSgII-70.) The gel was re-run and blotted and the lowest band of about 6 kDa was sequenced by automated Edman N-terminal sequencing. This produced a major sequence identified as a bacterial protein: acyl carrier protein or flavodoxin-related protein. A subsidiary sequence yielded SEQ ID NO: 2 of S1 Table 1. This could not be identified by BLAST searches.

A further sample of upstream precipitate from sheep jugular vein EDTA plasma was separated by SDS-PAGE and blotted onto immobilon for Edman sequence analysis. Band 1 (see S1 Fig. 9) at ~6 kDa gave the EPL001 sequence, SEQ ID NO: 3 of S1 Table 1, deemed by the operator ‘a relatively clean sequence’. No database hits were obtained using BLASTALL or FASTA searches. Subsequent database searches likewise proved negative (Hart et al, 2017).

Band 2 at ~7 kDa gave a mixed signal, garnering no database hits. Unlike EPL001, with its Beale 4 echo, there was no evidence of this material in downstream anion exchange fractions. Band 3 was a strong single sequence identified in the databases as a bacterial ribosomal protein. Band 4 (~10 kDa) was the same protein with an unidentifiable secondary sequence. Band 5 (~14 KDa) was a suspected bacterial protein


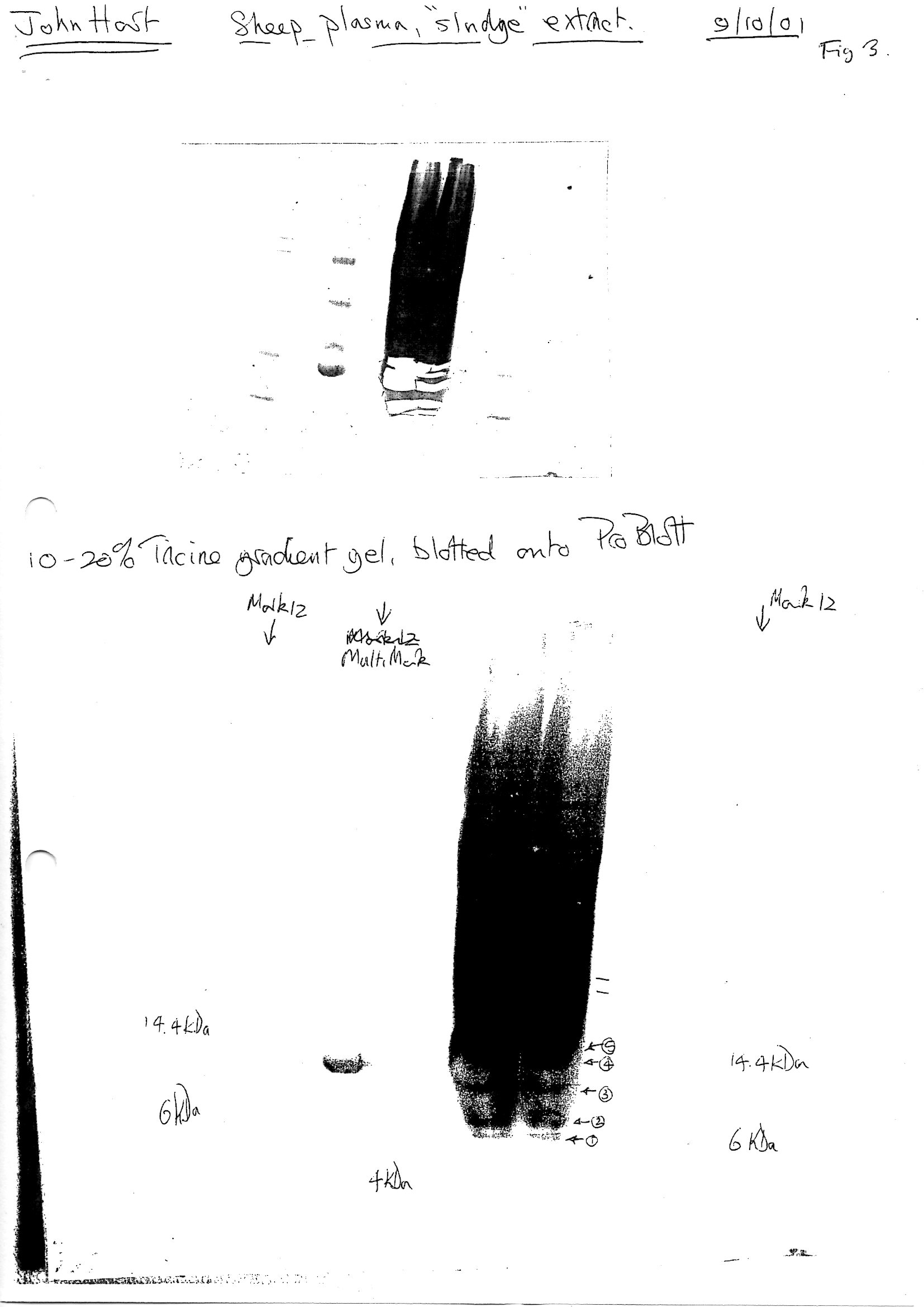


**S1 Figure 10.** Lab book representation of the gel from which the EPL001 sequence was obtained (Band 1) via automated Edman sequencing using an Applied Biosystems Procise instrument. (Personal communication, Pat Barker, Babraham Institute, Cambridge, UK.)

An aqueous extract was prepared from a further batch of upstream precipitate and analysed by SDS-PAGE and blotting. This gave three well-defined bands just above 6 kDa. These were cut out for Edman sequence analysis. Band 1 (just over 6 kDa) gave a strong but mixed signal, in which the EPL001 sequence were clearly seen, with additional residues C-terminally. This is Seq ID NO: 5 in S1 Table 1, the EPL001 Extension. Band 2 was a bacterial cold shock protein sequence. Band 3 was a DNA binding protein of several bacteria.

SDS-PAGE gradient gel of upstream precipitate that was bacterially contaminated was run side by side with comparable material that was ostensibly not contaminated (having been treated with sodium azide). Proteins were extracted from the gel by a solvent extraction method and the soluble material run on MALDI-TOF MS for determination of accurate molecular weights.

The following is a quotation (**‘…’)** from a lab report [with interpolations relevant to the present paper in square brackets]:

**‘**The peaks observed were as follows.

Extracted bands of contaminated material:

*Band 1*: Major peak is at 7572, but there is also material at about 6887 and also at lower molecular weight (4604, 3787, 2767). Heterogeneity around the main peaks is probably due to SDS. [Note that 3787 is about half of 7572, i.e. 7572 ÷ 2 = 3786.]

*Band 2*: Major peak is at 7645, nothing much at other sizes, some heterogeneity as above.

*Band 3*: Main peak is still at 7648, but several lower mol wt peaks and one at 9574.

*Band 4*: Main peak at 7830, some at 6528 and 9575.

*Band 5*: Main peak at 7830, with some at 3916. [Note that 3916 is about half of 7830: 7830 ÷ 2 = 3915.]

The presence of material of about 7500-7800 size in all fractions is rather puzzling. Nevertheless, it suggests that the candidate in Band 1 may well be that to which we have previously attributed a size of 7500 by mass spec analysis.

Uncontaminated gel:

*Band A (=1)*: Main peak at 7579, some at lower sizes (3791, 4607, 5998, 6741) and 8383. [This mass spectrum is available as S3 Fig. 5. The 8383 item is possibly an MS artefactual heterodimer of SgII-70 fragments, as in 3791 + 4607 = 8398, which could account for the 8-9000 items in Bands B-D as well. Solvent extraction of gel material promotes apparent dimerization in a way that electoelution, to be described shortly, does not.]

*Band B (=2)*: Peak at 7649, with some lower mol wt but also more at 8375 and 8938.

*Band C (=4)*: Main peak at 8935, next at 8376 and quite a bit at 4466 [potential sSgII-70 39mer].

*Band D (=5)*: Main peak at 8933, a little at 4468 [potential sSgII-70 39mer] and 9739.

Molecular weights in these eluted fractions increased more in line with expectation. They tend to confirm that the Band 1 size is indeed around 7500 rather than 6000 estimated from the gel. Mass spectometry would be a more accurate size determination than the gel since a number of factors can affect mobility.**’**

For the uncontaminated material the above procedure was repeated using electroelution (Hart, 2008). Gel bands of interest were placed in elution tubes in a Bio-Rad Model 422 Electro-Eluter (used according to the manufacturer’s instructions) with Tris/glycine, then eluted at 10 mA per sample, using a 3500 Da cut-off membrane. The final volume was approximately 250-300 µl. Eluted material was tested in the BMC assay in vitro, with inhibition demonstrated for all bands tested. The MALDI-TOF MS result was as follows, as reported (‘…’) by the operator [with interpolations relevant to the present paper in square brackets]:

***‘****Band 1*: Single peak at 7583. [This uncluttered mass spectrum is the paper’s Fig. 5; analysed via the paper’s Table 1 to be an sSgII-70 65mer, having two isopeptide bonds and no water reductions.]

*Band 2*: Single peak at 7654. [66mer, having two isopeptide bonds and three water reductions]

*Band 3*: Single peak at 7660. [66mer, ditto]**’**

The foregoing supports the view that Candidate 7500 is non-bacterial, while indicating that this entity can be detected across several bands of relevant molecular weight in SDS-PAGE, perplexingly. In this paper’s analysis the different numbers in the range *m/z* 7-8000 of Candidate 7500 are MS artefacts: the various peaks all represent fragments of the same 70mer, deduced to sport a pair of crosslinks. Why do these molecules run as multiple bands? Referring to the Gel Band 1-3 electroelution MS results, differential endogenous water reductions does not appear to explain it, these being deemed by the paper to be MS artefacts.

Washing the upstream precipitate with water and reanalysing the solid caused Candidate 7500 of the paper’s Fig. 4 (upper panel) to disappear, as per the mass spectrum of S1 Fig. 10. This speaks of high solubility in water. The MS results shown in the paper’s Fig. 4 are in fact especially striking. Candidate 7500 appears as double, aa-gapped peaks in two spectra involving different feedstocks from two species: bovine ovarian follicular fluid and ovine jugular vein EDTA plasma. Why did such clean spectra eventuate at all, when co-precipitation of mammalian and bacterial material was evidently at issue, before sodium azide was deployed?


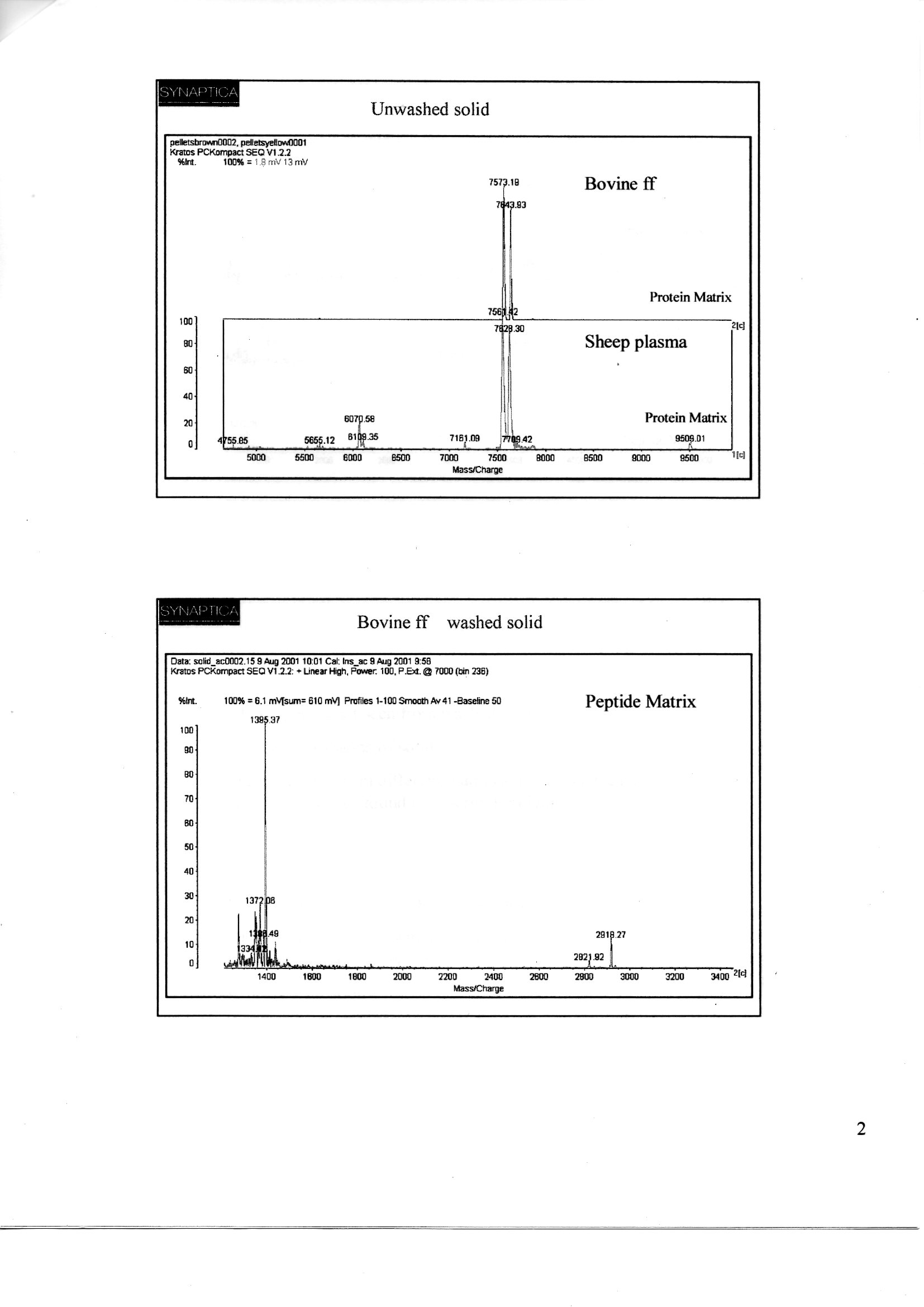


**S1 Figure 11.** Mass spectrum of upstream precipitate of bovine ovarian follicular fluid (ff) subject to washing with water, which removes Candidate 7500. (To be compared with the paper’s Fig. 4, upper panel.) Mass spectrum courtesy of Carolyn Carr, University of Oxford, Oxford, UK.

Reflecting on the 3-30 kDa upstream precipitate obtained by the Sheffield Method and analysed at Babraham, it is clear that the precipitate on the 3 kDa ultrafiltration membrane was largely of bacterial origin, until sodium azide curtailed it, but the EPL001 sequence that resulted from the early stage of this upscaled ultrafiltration exercise is not bacterial (Hart et al, 2017). The EPL001 sequence in any case coheres with the minimal sequence (Beale 4) from maximally purified (anionex) material free of contamination.

A candidate molecule for an endogenous hormone is teasingly difficult to bring into focus in pure preparations. What is to be made of such a moiety that during a crude, bacterially compromised ultrafiltration scale-up obligingly migrates in waves at the forefront in SDS-PAGE and soars aloft alone from the mire in MS?

[ENDS]
