# Supplementary material for: Detecting the body’s reproductive hormonal brake against tissue overgrowth: Micrin/SgII-70
Source: PLoS One. 2026 Mar 2;21(3):e0340980. doi: 10.1371/journal.pone.0340980 (PMC12952585; doi:10.1371/journal.pone.0340980)
Supplement: S2 File — https://doi.org/10.6084/m9.figshare.30688655. This project details the methodology used in exemplifying antiorganotrophism for the candidate molecule and gives results. (DOCX) [file pone.0340980.s002.docx]

**Supplementary Information 2 (S2)**

**Assay Methods & Results**

S2 is provided in support of ‘Detecting the body’s reproductive hormonal brake against tissue

overgrowth: micrin/SgII-70’ by Hart JE, Davies KG, Mundy CR, Hart AC, Howlett DR & Newton RP (2024 submission). Corresponding author email: [k.davies@herts.ac.uk](mailto:k.davies@herts.ac.uk)

Assay methods and results relating to the testing of synthetic peptides may be found in Hart et al, 2022, the paper’s ref [22].

Ovine materials were obtained from adult ewes in flocks maintained for experimental purposes at The Babraham Institute, Cambridge, UK and at Harwell Laboratory, South Oxfordshire, UK. Bovine and porcine material was obtained from abattoirs. Rats were held in dedicated licensed facilities at Babraham and Harwell and at the University of Sheffield, Sheffield, UK.

The paper displays as Fig. 1 an exemplification of antiorganotrophism (tissue reduction) in vivo. The rat organometric assay in question was conducted according to a standardised method over four days with intraperitoneal dosing (Hart, 1990b [3]), as is the case with all the rat organometric assays described here in S2. Data are presented thus: the mean relative (post-exsanguination) organ weights of the test rats were first expressed as a percentage of the control means. These figures were then subtracted from 100 to yield the percentage difference between test and control means, the results being plotted against a zero baseline representing control values. All data have been archived. The details of the Fig. 1 assay are provided later in this supplementary file.

The rat assay in vivo formed part of a bioassay-guided fractionation. The early work was based on physicochemical purification, as shown in the following flowchart, when the focus was on factor eluting early (low salt) in anionex (S1 Babarham Method).


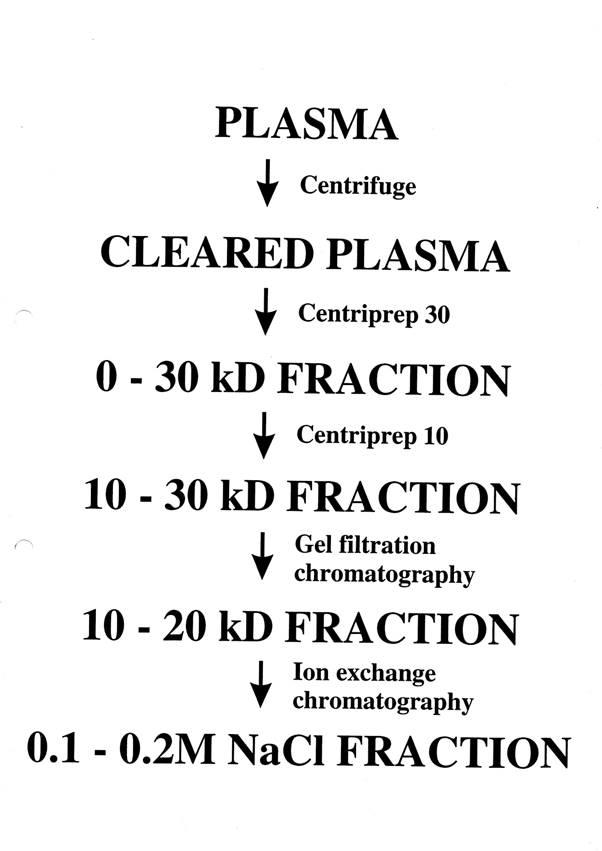


**S2 Figure 1.** Purification (S1 Babraham Method).

Preliminary results with the rat organometric assay were presented in the patent literature (Hart, 1999 [10]; Hart, 2000 [16]). Organ shrinkage is readily provoked in what is a relatively crude assay in vivo, but the exact organometric pattern is unpredictably related to the totality of factors present at each stage of the purification. Here for example is a result obtained with raw plasma.


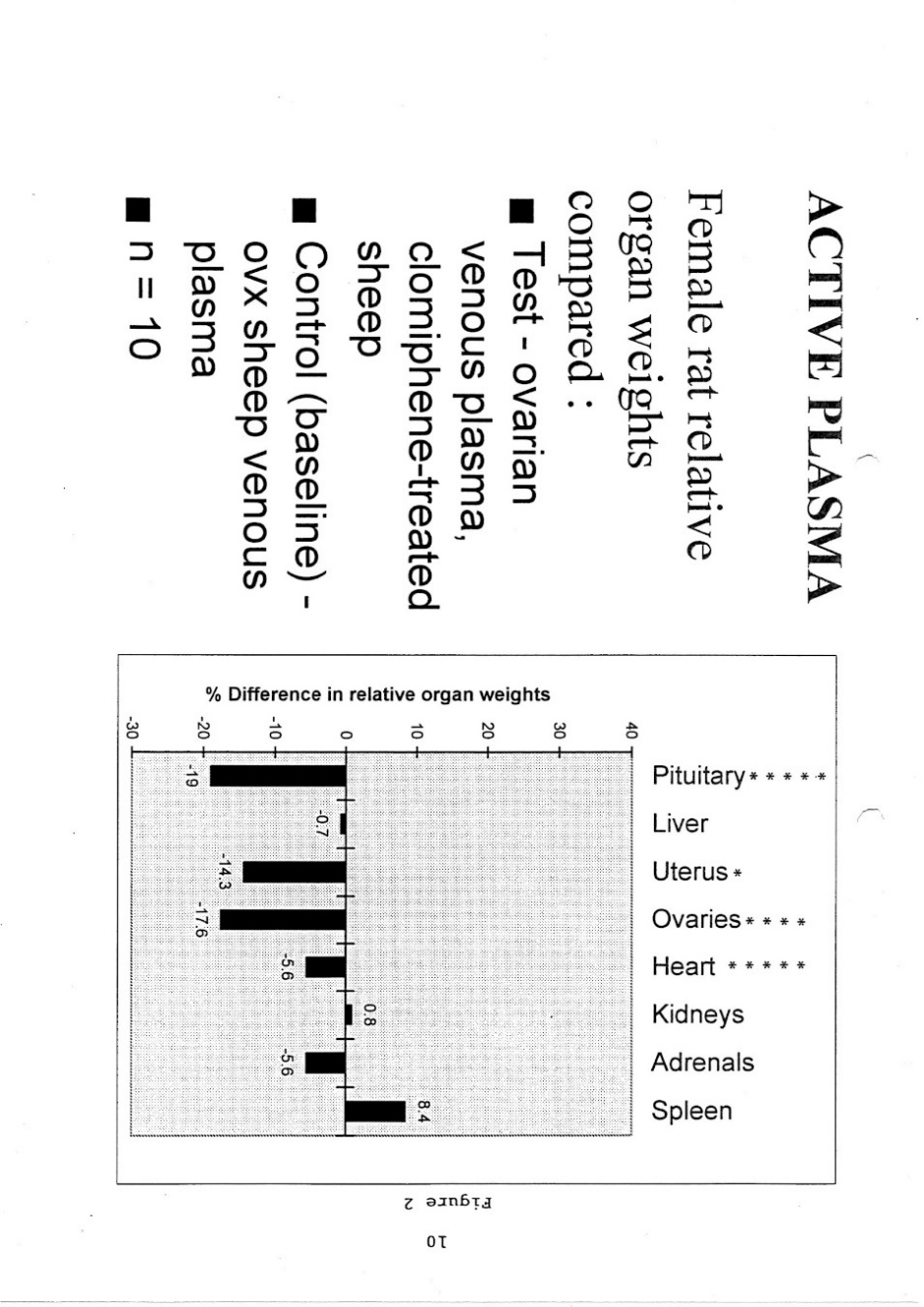


**S2 Figure 2.** Neat plasmas compared, with use of clomiphene in donor OV-INTACT sheep as a putative micrin stimulator (Hart, 2014 [7]) to provide rat-organ-shrinking ‘Active Plasma’. Ovarian venous plasma from a clomiphene-treated sheep versus jugular vein plasma from an OVX sheep untreated with clomiphene. Babraham result from Hart, 1999 [10]. The statistical analysis involved t tests: * = P<0.05; ** = P<0.02; *** = P<0.01; **** = P<0.002; ***** = P<0.001.


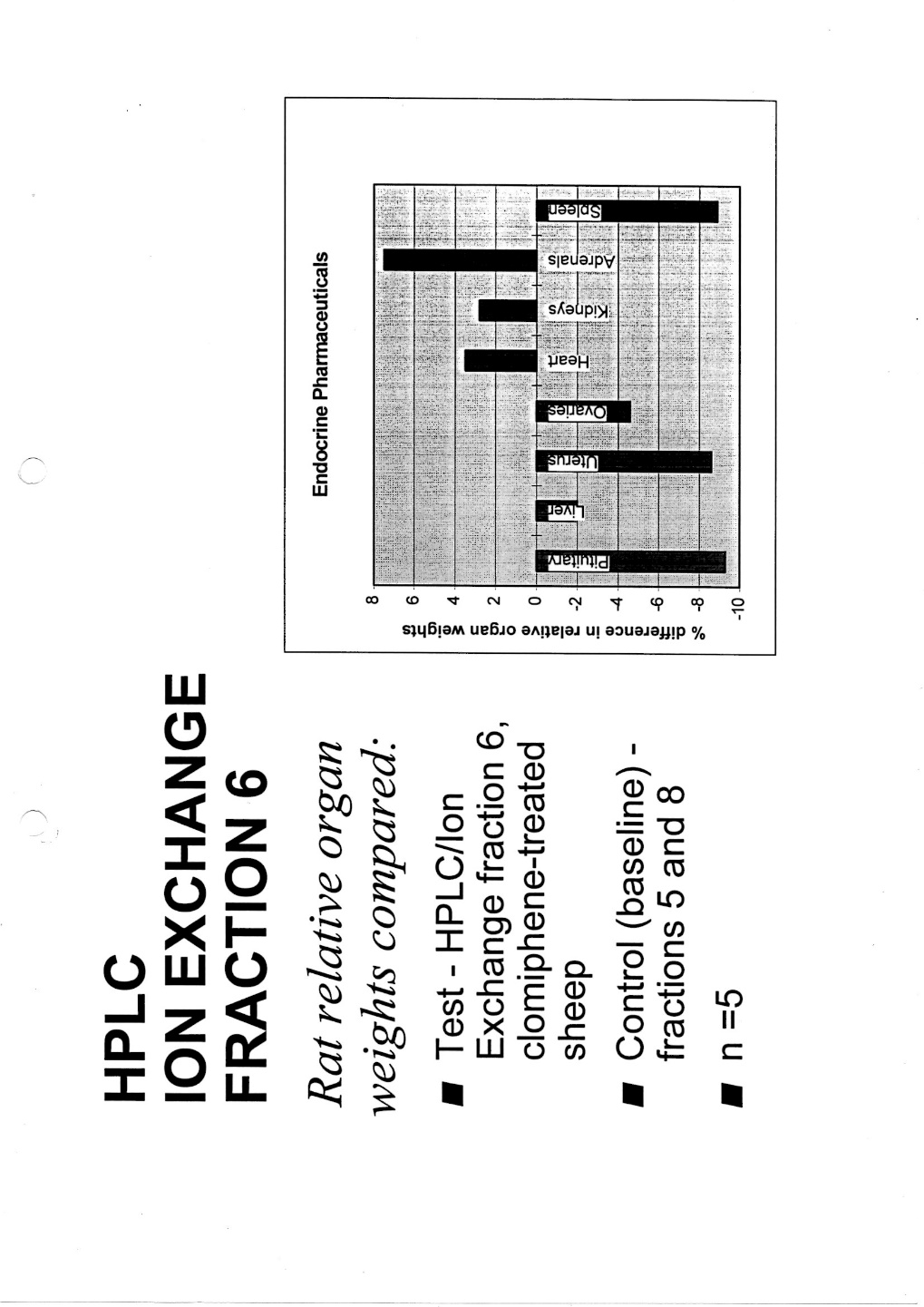


**S2 Figure 3**. Comparison of anionex HPLC fractions of ovarian venous plasma subject to prior size fractionation (S1 Babraham Method). Note the micrin signature of ‘pituitary down, adrenals up’. See next figure for fraction details.


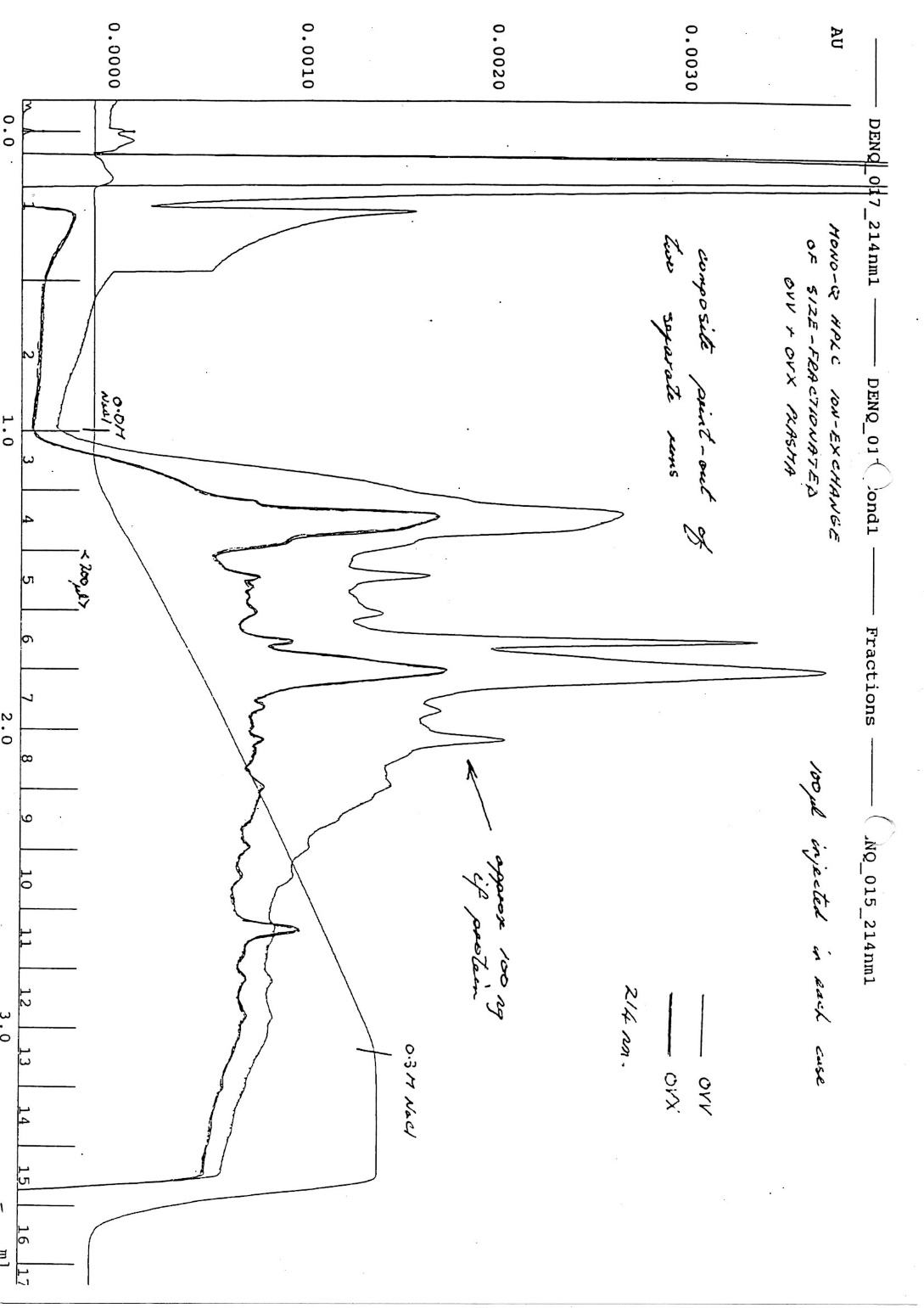


**S2 Figure 4.** UV trace showing anionex HPLC fractions used in regard to the assay result displayed as S2 Fig. 3.

Candidate 7500 was identified bimodally in early (low salt) and late (high salt) eluting anionex fractions using MALDI, both types of fractions independently demonstrating antiorganotrophic activity in the rat assay in vivo.

Described in the paper are the results of an organometric assay in male rats. Next are those results in the form of the standard histogram.


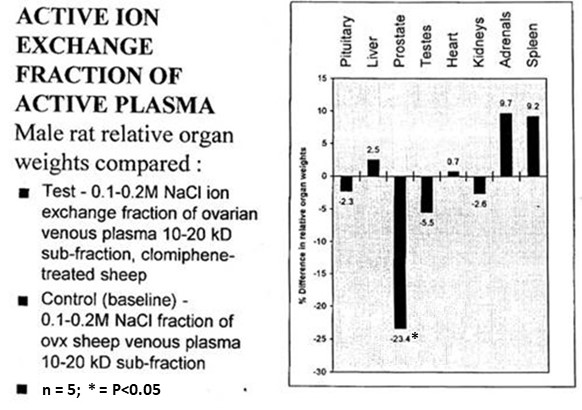


**S2 Figure 5.** Male rat organometric assay, with clomiphene used in donor OV-INTACT sheep as a putative micrin stimulator (Hart, 2014 [7]). Conclusion within the hypothesis of a tissue-reducing hormone: micrin from female sheep shrinks the rat prostate, while providing the ‘pituitary down, adrenals up’ signature; cross-species bioactivity is evinced, cross-sex. Babraham result from Hart, 1999 [10]. The statistical analysis involved t-tests.

Referring now to the paper’s Fig. 1, the legend for that is reproduced here for ready reference:

‘Organometric effects in rats in vivo of plasma anionex fractions from OV-INTACT vs OVX sheep. Post-mortem organ weights expressed as a percentage of body weight for two groups of seven intact adult female Sprague Dawley rats. The zero baseline is provided by rats receiving for four days by intraperitoneal injection a fraction (spin and gel filtered to 10-20 kDa, eluting in HPLC anionex at ~0.2 M NaCl; S1 Babraham Method) of the jugular vein plasmas pooled from two OVX sheep. The other group of rats received the same fraction in the same amount from ovarian venous plasmas pooled from six OV-INTACT sheep. No significant body weight changes were seen to confound the picture of organ shrinkage with adrenal enlargement. * = P<0.05, one-tailed t test. (OV-INTACT versus PBS controls, adrenals +8.53%, significant: S2.)’

The OV-INTACT sheep were at approximately day 8 of their oestrus cycle. To determine the stage of the cycle in the ewes, a vasectomised ram was introduced to the flock (Harwell Laboratory, South Oxfordshire, UK). At various times a raddle was used to demonstrate with ewes had been covered. Once a ewe was covered that day was counted as day 0 in the cycle. Ewes were selected on this basis and treated with saline (20ml) on each of 4 days before the plasma collection day. (This was a control procedure for another group of ewes receiving a separate intervention.) At approximately day 8, the sheep were anaesthetised with pentobarbitone (Sagatal Rhone Mereiux). Anaesthesia was maintained with Halothane in oxygen. Ovarian blood (5-600 ml) was collected from both ovaries through cannulae, into heparinised tubes (1 iu/ml blood). (It was later shown that the use of EDTA as anticoagulant led to superior retention of bioactivity: S1 & later.) Blood tubes were stored on ice until the completion of collection when they were centrifuged at approximately 3660 G for 30 min. The plasma was then separated into aliquots and stored at –20 °C. In addition to six OV-INTACT sheep, plasma was obtained from two OVX ewes (saline treated). Ewes were ovariectomised on 31^st^ July. Blood was collected from one on 19^th^ August of the same year, from the other on 2^nd^ September. These animals were anaesthetised with pentobarbitone. The jugular vein was exposed and approximately 1 L of blood withdrawn. Plasma was separated by centrifugation and stored as for the OV-INTACT sheep. At the time of the blood collection the mean body weight of the six OV-INTACT ewes was 82.5 kg (range 71-95), with a mean age of 9.3 years (range 7-12). The two OVX ewes weighed 70 kg and were aged 11 and 12 years. After a recovery period, animals were returned to the flock.

Plasmas in the two groups were bulked separately and subjected to the Babraham Method of purification, as described in S1: spin filtration, gel filtration, HPLC anionex. The rats received pooled anionex fractions. The following is a quotation [**‘…’**] from the Harwell lab report [with interpolations relevant to the present paper in square brackets]:

**‘**The test plasma [OV-INTACT & OVX] had been subjected to centrifugation/filtration [spin filter] to remove proteins of molecular weight >30kD. A second centrifugation/filtration step concentrated everything in the nominal molecular weight range 10-30kD, representing an approximately 400 fold concentration of these plasma components. The concentrate was applied to a Superdex 75 gel filtration column, the chromatography controlled by a Pharmacia FPLC system. 6 fractions, covering the molecular weight range 10-20kD [understood at this stage in the project to encompass the likely MW of the target molecule] were collected from this column, this range having been established by prior calibration using proteins of known molecular weight under the same running conditions. Monitoring the column eluent at 214 nm showed these fractions included an absorbance peak corresponding to molecular weights of approximately 12kD.

The fractions from the Superdex column were pooled and concentrated, using a 3kD cut-off centrifugation/filtration membrane and the concentrate loaded on to a Pharmacia MonoQ anion exchange column [HPLC], with the aim of resolving multiple molecular species which may underlie the single gel filtration peak. The column was developed with a salt gradient [0-0.3 M NaCl in tris buffer], but only a single broad absorbance peak was obtained, representing fused peaks, at a salt concentration of approximately 0.2M. The fractions covering these peaks were retained. Fraction volume was 0.5 ml.

Each fraction [pool] was diluted to 28 ml with sterile phosphate buffered saline. This volume of diluent was calculated to give the equivalent of 1.0 ml of raw sheep plasma in 1 ml for dosing in 7 rats in the bioassay. 1.1 ml volumes from each diluted pool were dispensed aseptically into individual vials.**’**

Female Sprague Dawley rats (SPF) were obtained from Charles River, Kent, UK, a facility licenced by the United Kingdom Home Office. They were checked and all appeared fit and healthy. The animals were weighed 6 days later and the weights were entered into Artemis II. The rats were allocated to groups of 7 such that the mean bodyweight and the standard deviation were similar between the groups. Throughout the study the rats were housed two per cage with food and water available ad libitim. Two days after weighing the animals were reweighed (‘Start Weight’) and began to receive intraperitoneal injections of 1 ml of OVX anionex plasma fraction or the OV-INTACT equivalent. (A control group received sterile phosphate buffered saline, PBS.) Injections were performed daily for 4 days at a similar time of day, when behaviour and general condition were observed. On the fifth day each animal was weighed (‘Death Weight’), anaesthetised with CO_2_ and approximately 5 ml of blood was withdrawn by cardiac puncture. Gross morphological findings at autopsy were absent. Organs, as per the tables, were removed, trimmed free of fat and weighed within 5 min of removal. Organs from the first three animals in each group were fixed in 10% buffered formyl saline for histology (which yielded no significant findings).

For each animal the mass of each organ was calculated as a percentage of Death Weight. The mean and standard deviation of these relative organ masses were calculated.

The Harwell report reads as follow:

**‘**Analysis of body weights by analysis of variance showed no significant differences (p<0.05) between groups for either body weight at start of dosing or at death. A t-test on the weights of all animals at the start compared with those at the end showed no significant change in weight had occurred over the period of administration.**’**

**S2 Table 1.** Rat organ weights (g) and body weights (g), means ± standard deviation (n = 7), in reference to the rat organometric assay of the paper’s Fig. 1.

Group A = PBS

Group E = OVX

Group G = OV-INTACT


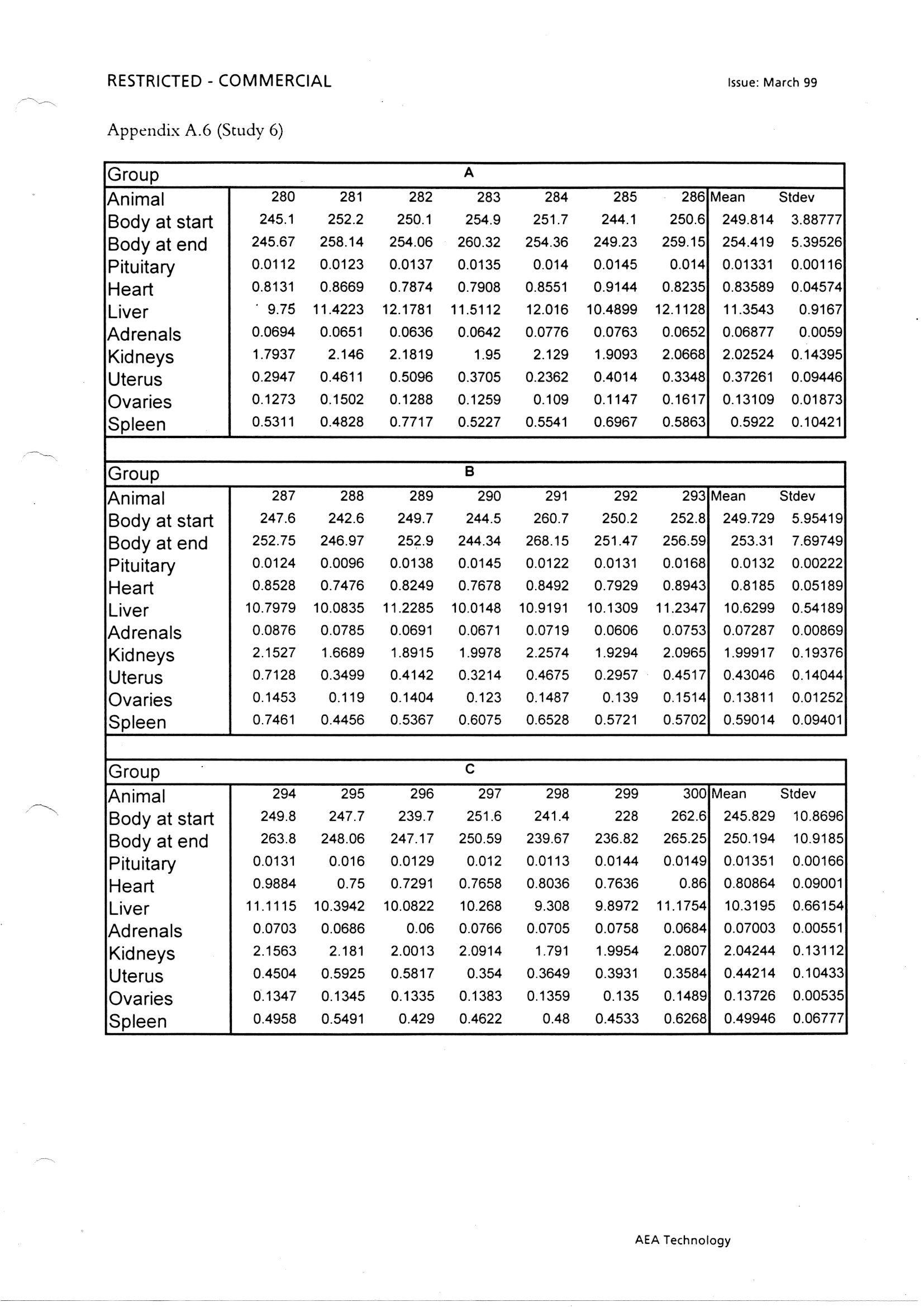


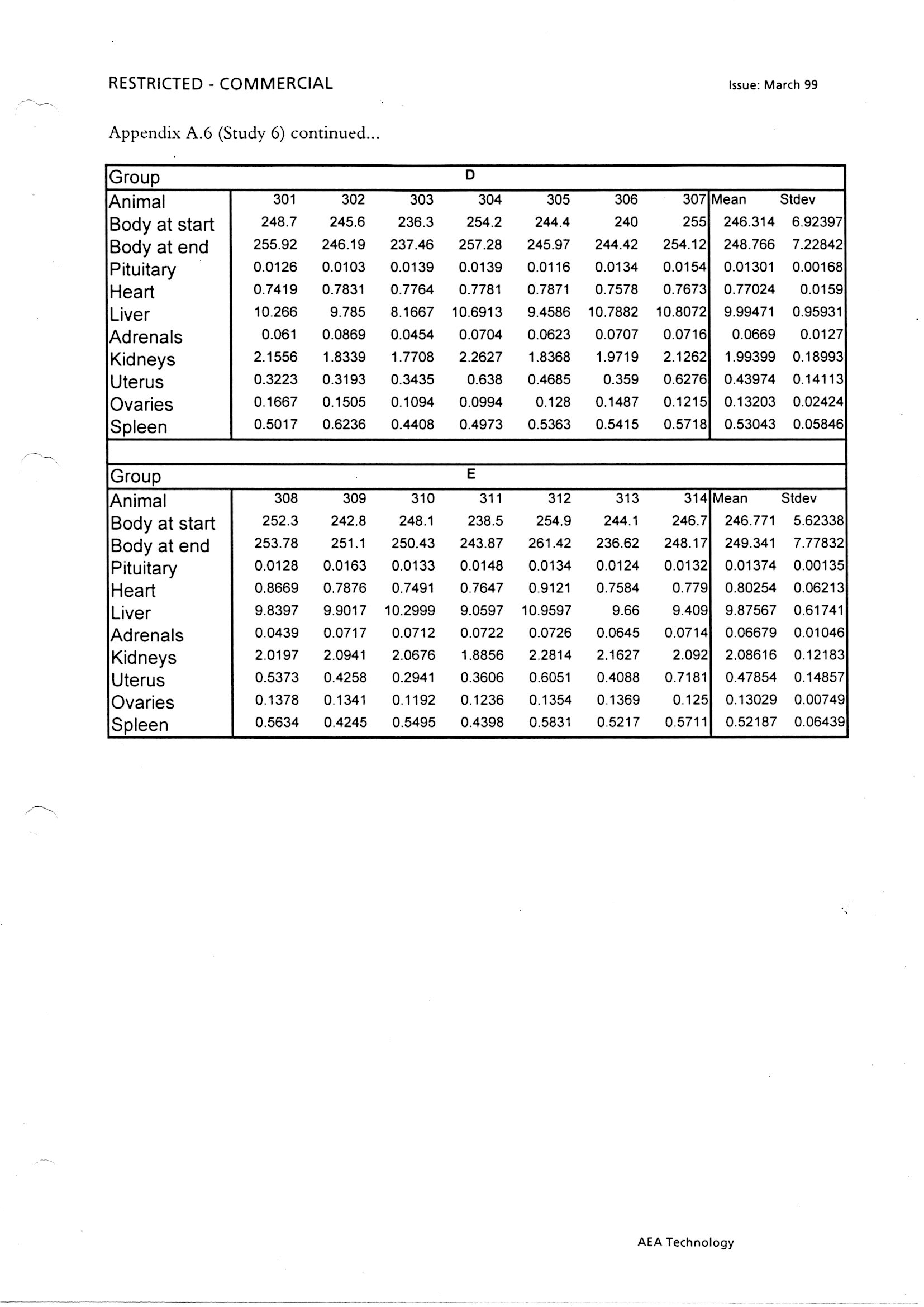


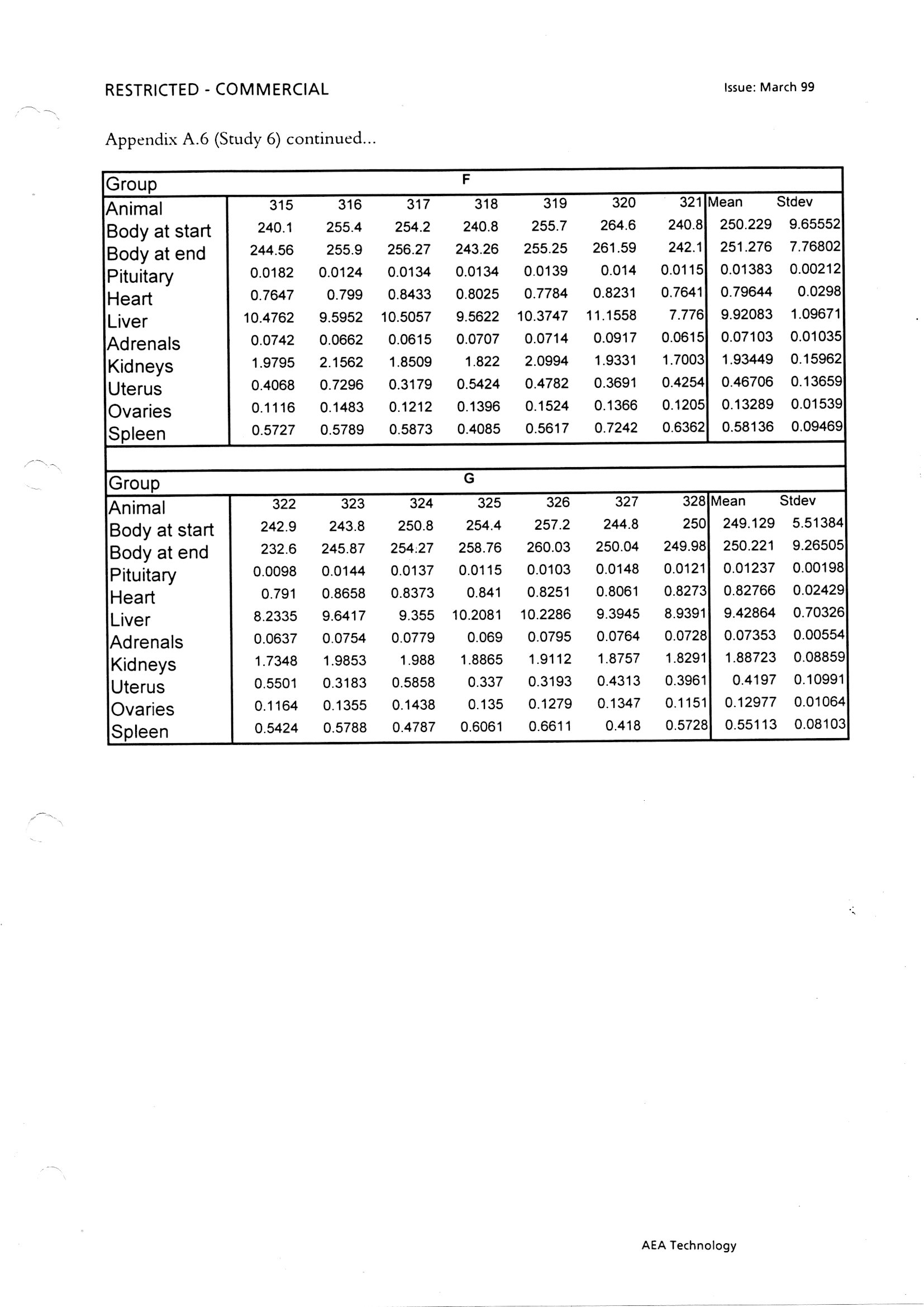


**S2 Table 2.** Rat relative organ weights (%) and body weights at Death (g), means ± standard deviation (n = 7), in reference to the rat organometric assay of the paper’s Fig. 1.

Group A = PBS

Group E = OVX

Group G = OV-INTACT


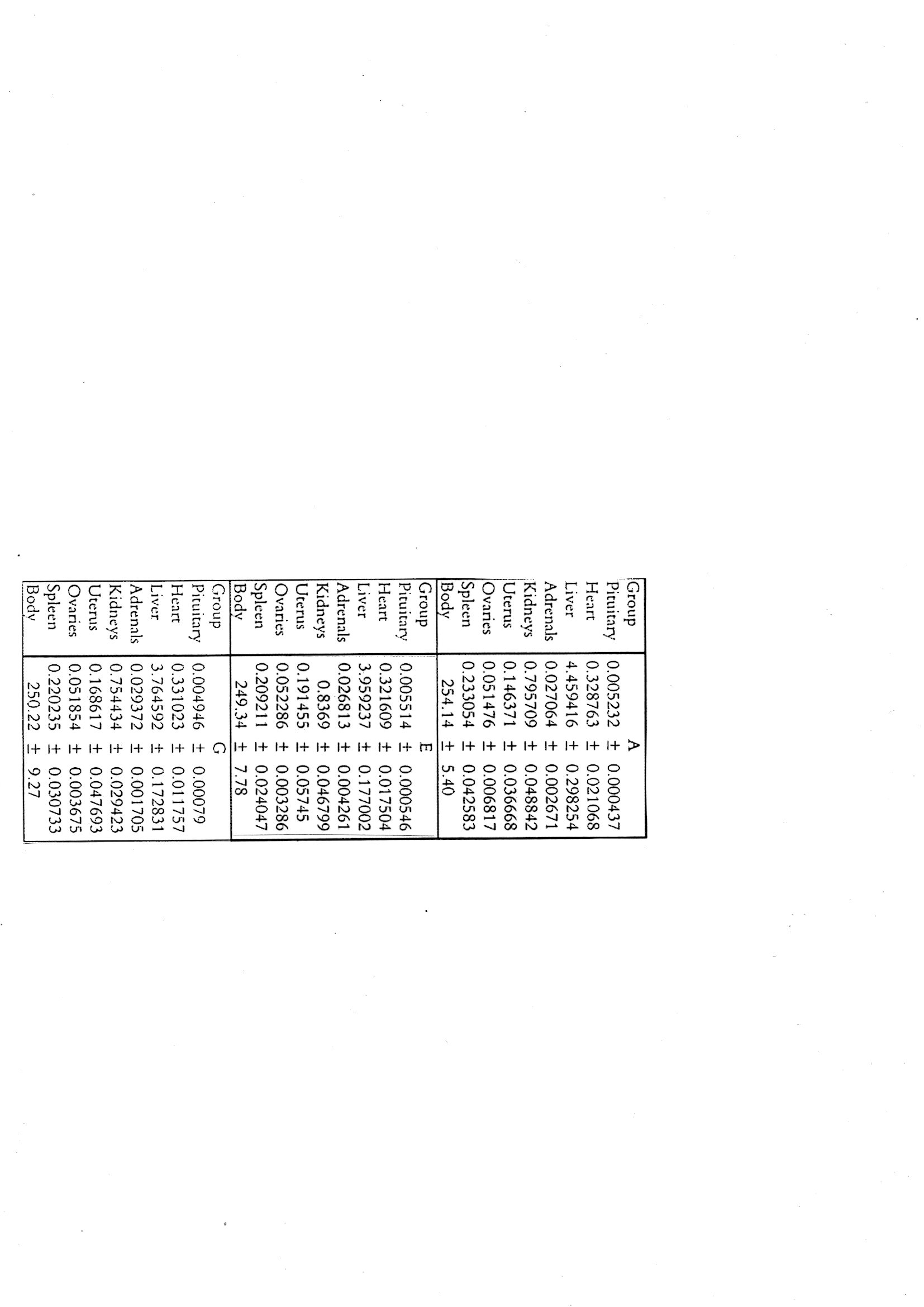


As between OVX and PBS, the only significant difference was that in the former there was a lower liver weight (-11.1%, p<0.05, one tailed t test). (Pituitary +5.39%, adrenals –0.93%, both ns.)

As between OV-INTACT and PBS, there were three relative organ weights significantly different (p<0.05) in the former group as compared with the latter: liver (–15.58%), kidneys (–5.19%) and adrenals (+8.53%). (Pituitary –5.47%, ns).

The paper’s Fig. 1 portrays the following comparison between rats receiving OV-INTACT and OVX anionex plasma fractions: pituitary –10.30% (i.e. OV-INTACT pituitary lower than OVX; significant), heart +2.93 (ns), liver –4.92% (s), adrenals +9.55 (ns), kidneys –9.85 (s), uterus –11.93 (ns), ovaries –0.83 (ns), spleen +5.27% (ns).

The results depicted in the paper’s Fig. 1 have been replicated, as below.

**S2 Figure 6.** Rat organometric assay, anionex HPLC fractions compared from OV-INTACT and OVX sheep. Harwell result. Study conducted as per the paper’s Fig. 1. Statistics: t tests.

A further replication of the paper’s Fig. 1 is shown next.


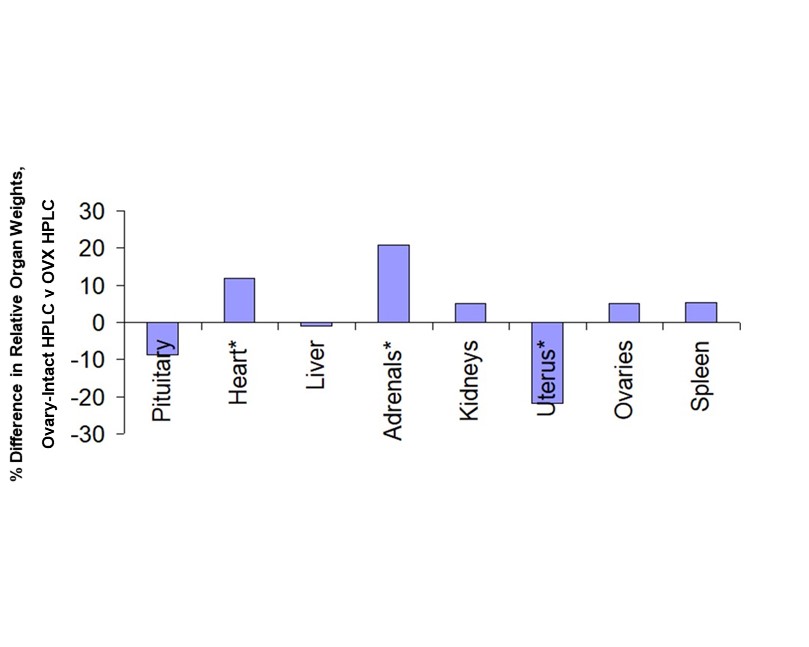


**S2 Figure 7.** Rat organometric assay, anionex HPLC fractions compared from OV-INTACT and OVX sheep. Harwell result. Study conducted as per the paper’s Fig. 1. Statistics: t tests.

* = P<0.05. Note that in this case the adrenal part of the micrin signature of ‘pituitary down, adrenals up’ reached statistical significance and the pituitary part did not. This is the reverse of the situation in the paper’s Fig. 1.

A uterus-only version of the rat organometric assay in vivo is described in the paper. It was carried out on a 3-30 kDa fraction of sheep ultrafiltered plasma subject to anionex HPLC (S1 Sheffield Method). Here next are the same data presented as a histogram.


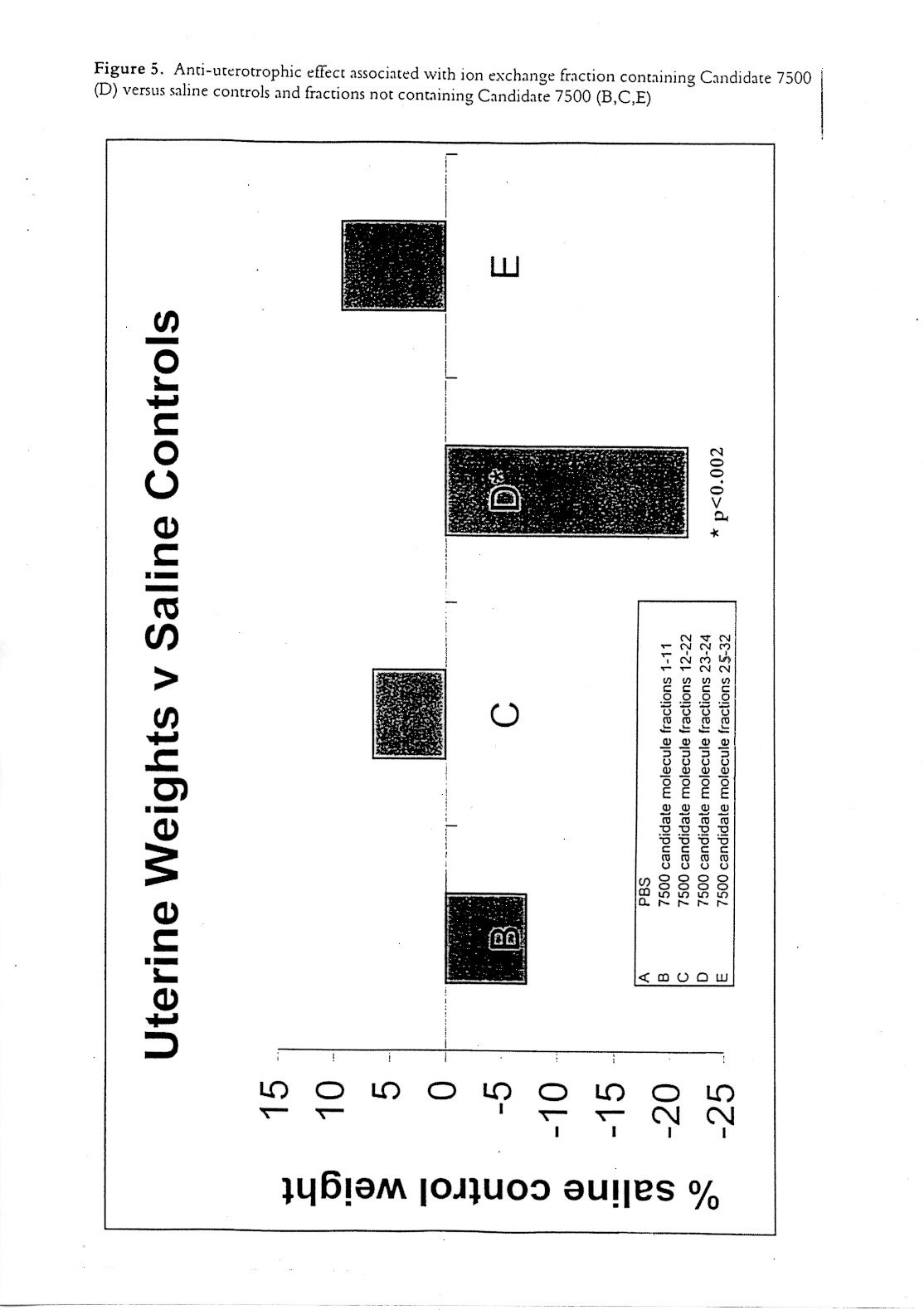


**S2 Figure 8.** Anti-uterotrophic effect in adult intact rats (n = 7) associated with HPLC anionex pooled Fractions 23 & 24 containing late-eluting Candidate 7500 (D), validated by MALDI-TOF MS, versus pooled fractions not containing Candidate 7500 (B, C, E). The zero baseline represents controls (A), which received PBS. Statistics: ANOVA.

**Renal studies**

*Basic model system:*

Surgically remove one rat kidney and the other grows, showing ‘compensatory renal growth’

(CRG) – to the point where the new kidney grows to be less than the former joint mass but

equal to the former joint glomerular filtration rate.

*Particular advantage:*

This system features a residual kidney under upregulatory pressure (notably from IGF-1). It is

a ‘boosted organ’ model involving direct administration into the organ of interest. This model

is thus of potentially greater sensitivity than the 4-day rat organometric assay.

|  | **Vehicle** | **Candidate 7500** | Stats Veh. vs Can. 7500 |
| --- | --- | --- | --- |
| Right Kidney Wet Weight (g) day 0 | 1.11 ± 0.03 | 1.07 ± 0.05 |  |
| Left Kidney Wet Weight (g) day 7 | 1.58 ± 0.06 | 1.32 ± 0.05 |  |
| **Compensatory Renal Growth (%)** | **41.7 ± 2.7** | **23.2 ± 2.6** | **<0.005** |
| Right Kidney Dry Weight (mg) | 252 ± 7 | 253 ± 11 |  |
| Left Kidney Dry Weight (mg) | 314 ± 16 | 296 ± 11 |  |
| **Compensatory Renal Growth (%)** | **25.9 ± 5.9** | **13.5 ± 1.1** | **<0.012** |
| Heart Wet Weight (g) | 0.94 ± 0.01 | 0.83 ± 0.02 | <0.01 |
| Fractional heart Wt (%) | 0.298 ± 0.01 | 0.27 ± 0.005 | <0.05 |
| Right Kidney Protein (mg) | 208 ± 30 | 245 ± 40 |  |
| Left Kidney Protein (mg) | 308 ± 52 | 309 ± 54 |  |
| **Compensatory Renal Growth (%)** | **65 ± 49** | **28 ± 14** | **<0.2** |
| Right Kidney DNA (mg) | 2.26 ± 0.15 | 2.37 ± 0.23 |  |
| Left Kidney DNA (mg) | 4.07 ± 0.27 | 3.15 ± 0.47 |  |
| **Compensatory Renal Growth (%)** | **81 ± 23** | **42 ± 15** | **<0.05** |

**S2 Table 3.** Effect of Candidate 7500 on compensatory renal growth. Hypertrophy of the left kidney was induced by removal of the right kidney. The remaining kidney was then treated with either vehicle or Candidate 7500 over 7 days using an osmotic infusion mini-pump. The animals (n = 4) were then sacrificed, the kidneys removed and wet weight, dry weight, total protein and total DNA determined. Data are mean +/– SEM. Compensatory renal growth (in terms of kidney wet weight gain) was inhibited by about 45%. This was significant in regard to three of the four CRG parameters assessed (P = <0.05, Student’s t test, equal variance). References: Hart, 2000 [16] & Hart, 2008 [18].

The renal series results are summarised in the following table.

**S2 Table 4.** Summary of renal studies. ‘EPL002’ = micrin containing anionex HPLC fractions of bovine ovarian follicular fluid subject to prior spin and gel filtration (S1 Babraham Method); EPL001 = synthetic 14mer peptide based on an Edman N-terminal sequence (S1); UNx = unilateral nephrectomy; SNx = unilateral nephrectomy with surgical reduction of residual kidney. All changes significant (t test) except a reduction in protein (indicated by NS). The antibody raised against EPL001 exaggerated compensatory renal growth, an observation in line with the immunoneutralisation of endogenous micrin. Results published in Haylor et al, 2009 [25].

**S2 Figure 8a.** Apart from a reduction in CRG in male UNx rats, a significant decrease in heart wet weight amounting >10% was also seen in animals treated with Candidate 7500 (‘Micrin’), as detailed in S2 Table 3. Mean +/– SEM (P = <0.01, significant, t test, n = 4). This finding is concordant with the inhibitory result seen in a cardiac hypertrophy assay in vitro described later. (Besides heart and residual kidney, other organ weights not determined.)

Reflecting on the observed cardiac shrinkage in regard to Candidate 7500 fractions, the rat CRG system is a boosted organ model in regard to the residual kidney. So is the post-UNx heart boosted? Unlateral nephrectomy increases blood levels of ANF, atrial natriuretic factor (Averbukh Z, Berman S, Weissgarten J, Cohn M, Golik A, Cohen N, Shaked U & Modai D, 1994. Relationship between Renal Mass and Atrial Natriuretic Peptide Release: 1. Paradoxical Effect of Unilateral Nephrectomy on Serum Atrial Natriuretic Peptide in Rats. *Kidney and Blood Pressure Research*, **17**, 101-107). ANF is a hormonal regulator of salt and water balance and is a marker of cardiac hypertrophy. Unilateral nephrectomy therefore appears to induce an upregulatory influence on cardiac mass and exaggerate an anti-cardiotrophic effect of micrin. In the UNx model system the heart is a ‘boosted’ organ.

CRG methodology from Haylor et al, 2009 [25]:


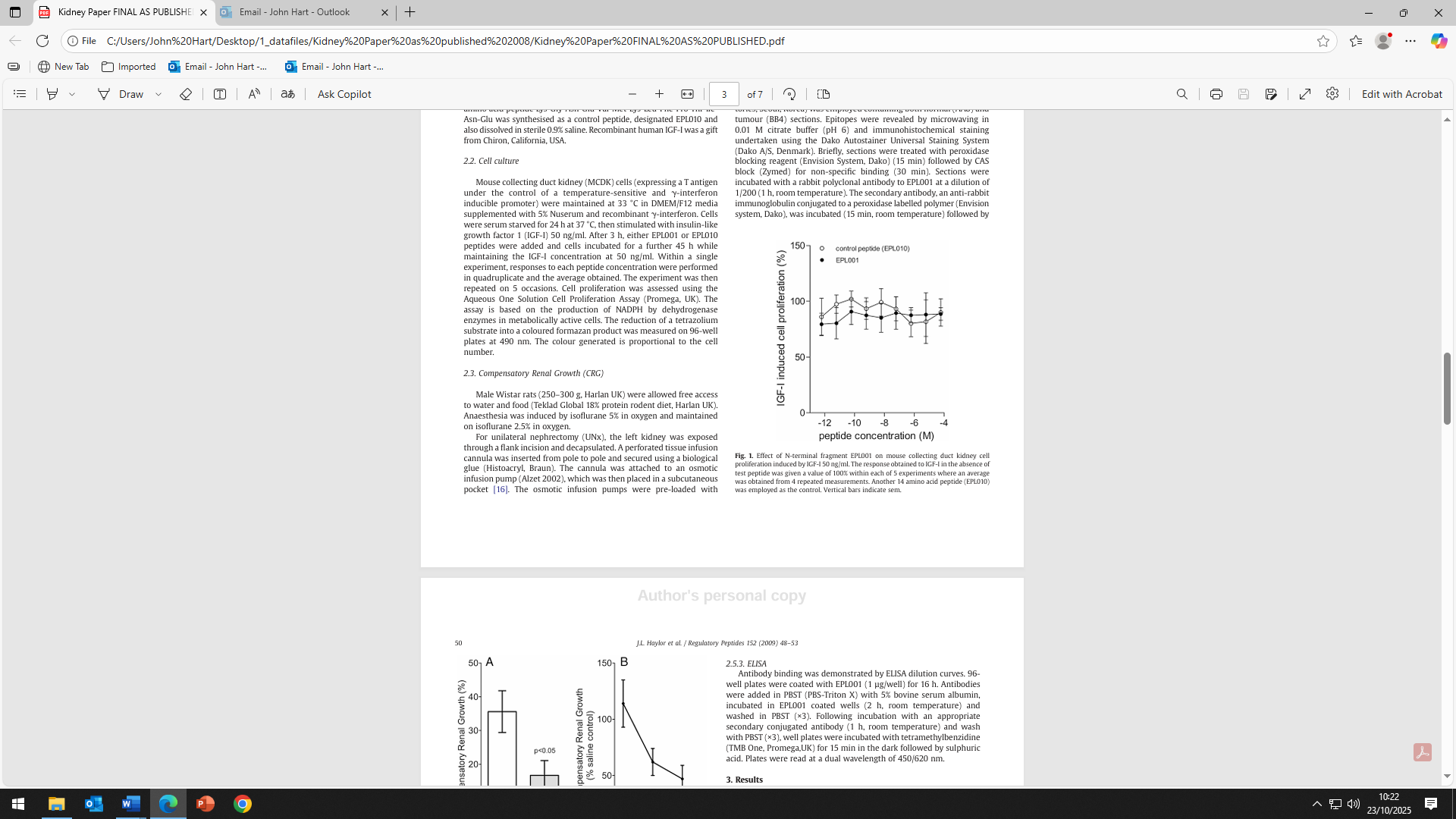

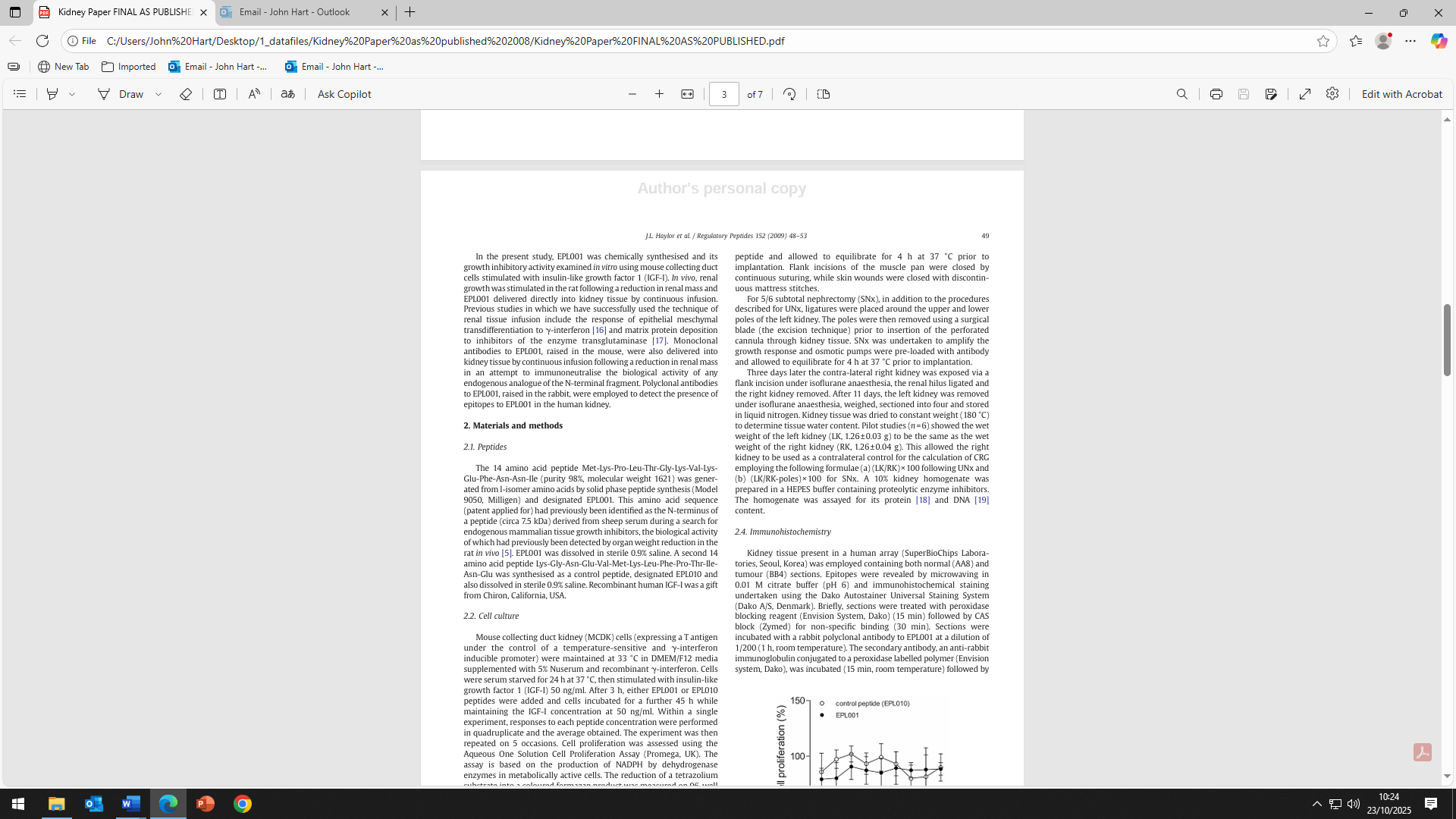


An experiment assessing the effects in aged sheep of ovariectomy on liver and kidney weights is discussed in S6, with a supporting graphic.

A rat assay in vitro was used to track activity in the bioassay-guided fractionation campaigns. This involved primary cultures of femoral and tibial bone marrow cells (BMCs). The methodology below is taken from a patent (Hart, 2000 [16]).


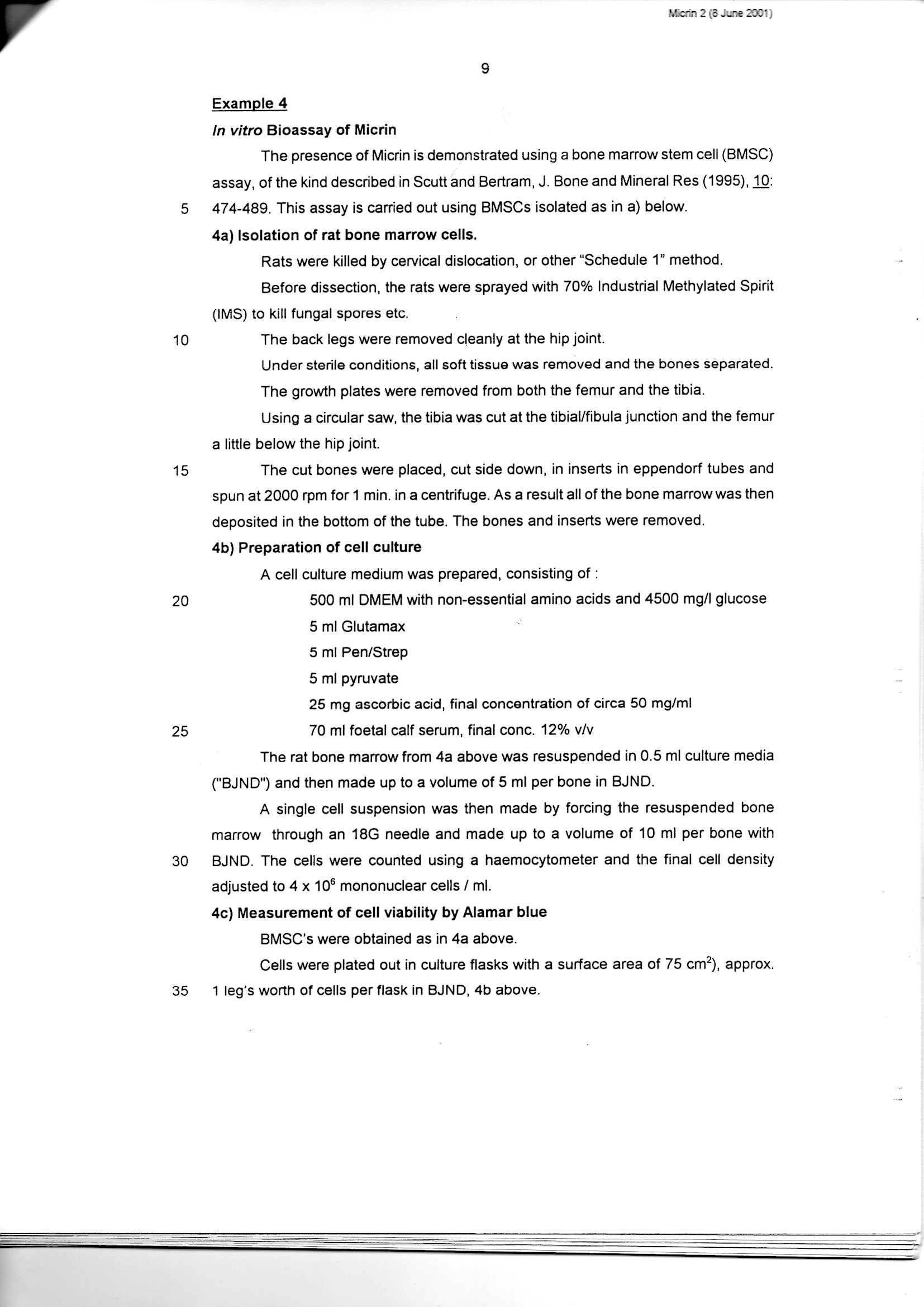


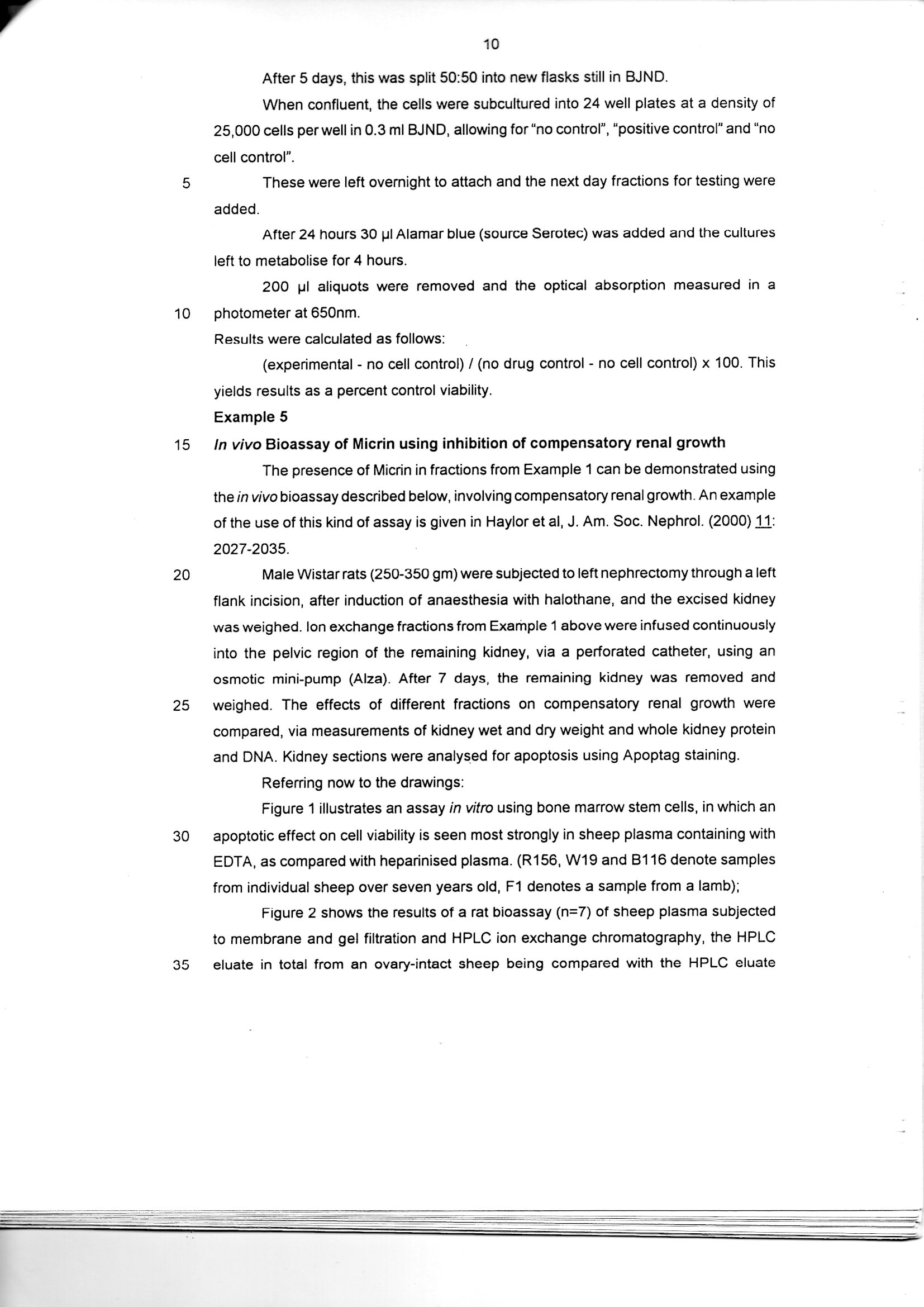


A cell shrinking activity, relating to the ovaries, could be readily demonstrated using neat plasma from OV-INTACT sheep, with such activity lacking in plasma from OVX sheep (Hart, 2000 [16]). The OV-INTACT plasma is putatively micrin-plus, the OVX plasma putatively micrin-minus.

**S2 Figure 9.** Cell size of BMC in vitro, with cells exposed to saline, OVX sheep jugular vein EDTA plasma and OV-INTACT sheep jugular vein EDTA plasma.


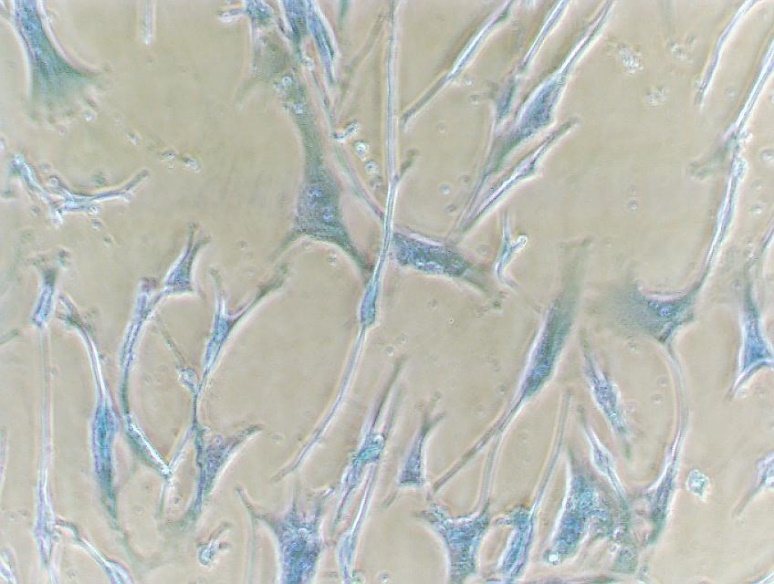


Saline control


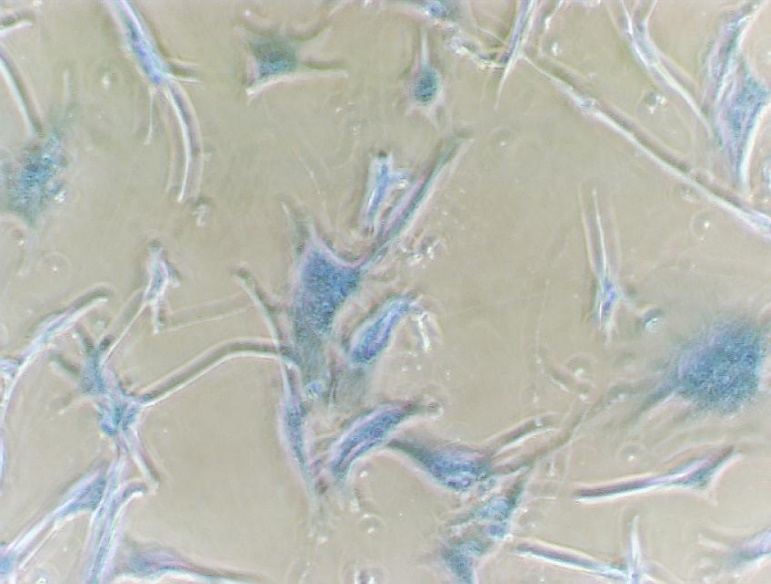


OVX


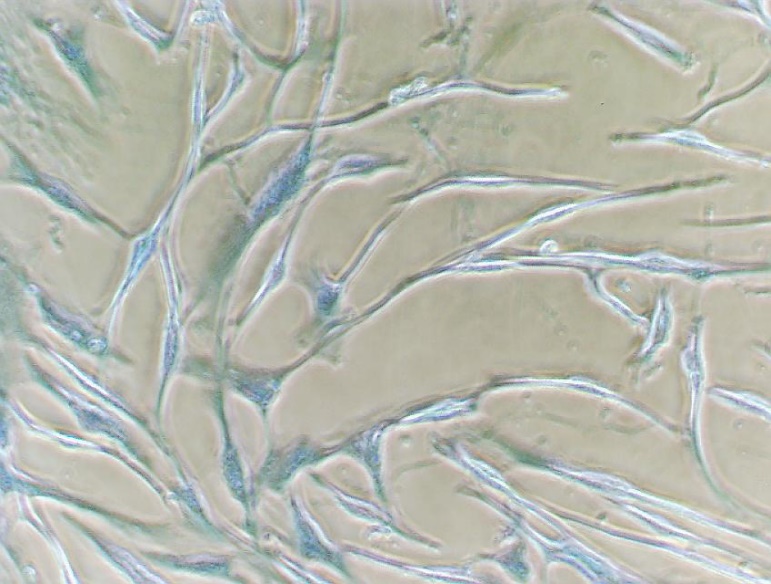


OV-INTACT

Cell size was evaluated using Magiscan image analysis, though the results are obvious to the microscope-aided eye. Cells in the saline control group are in the middle in terms of gross size, with cells in the OVX group larger and cells in the OV-INTACT group smaller. As a rough proxy for cellular volume, cross-sectional areas were assessed, all cells in 10 microscopic fields at x40 magnification. The results are shown in S2 Table 5. The OVX mean was above that of saline controls by 16%, while the OV-INTACT mean was 30% below it. The OV-INTACT mean was 40% below the OVX mean. With large variances, these data are ns by t test. The differences will have been greater in volume terms. Considering a sphere, the cross-sectional area is proportional to the radius squared, the volume to the radius cubed.

| **BMC *in vitro*** | **Cell cross-sectional area,  mean ± SD, arbitrary units** |
| --- | --- |
| 1. Saline control | 0.670 ± 0.382 |
| 2. Plasma from ovariectomised sheep | 0.779 ± 0.547 |
| 3. Plasma from ovary-intact sheep | 0.467 ± 0.233 |

**S2 Table 5.** BMC sizes after different treatments (Hart, 2000 [16])

For the in vitro assays, the sources of test materials were as follows (Hart, 2000 [16]):


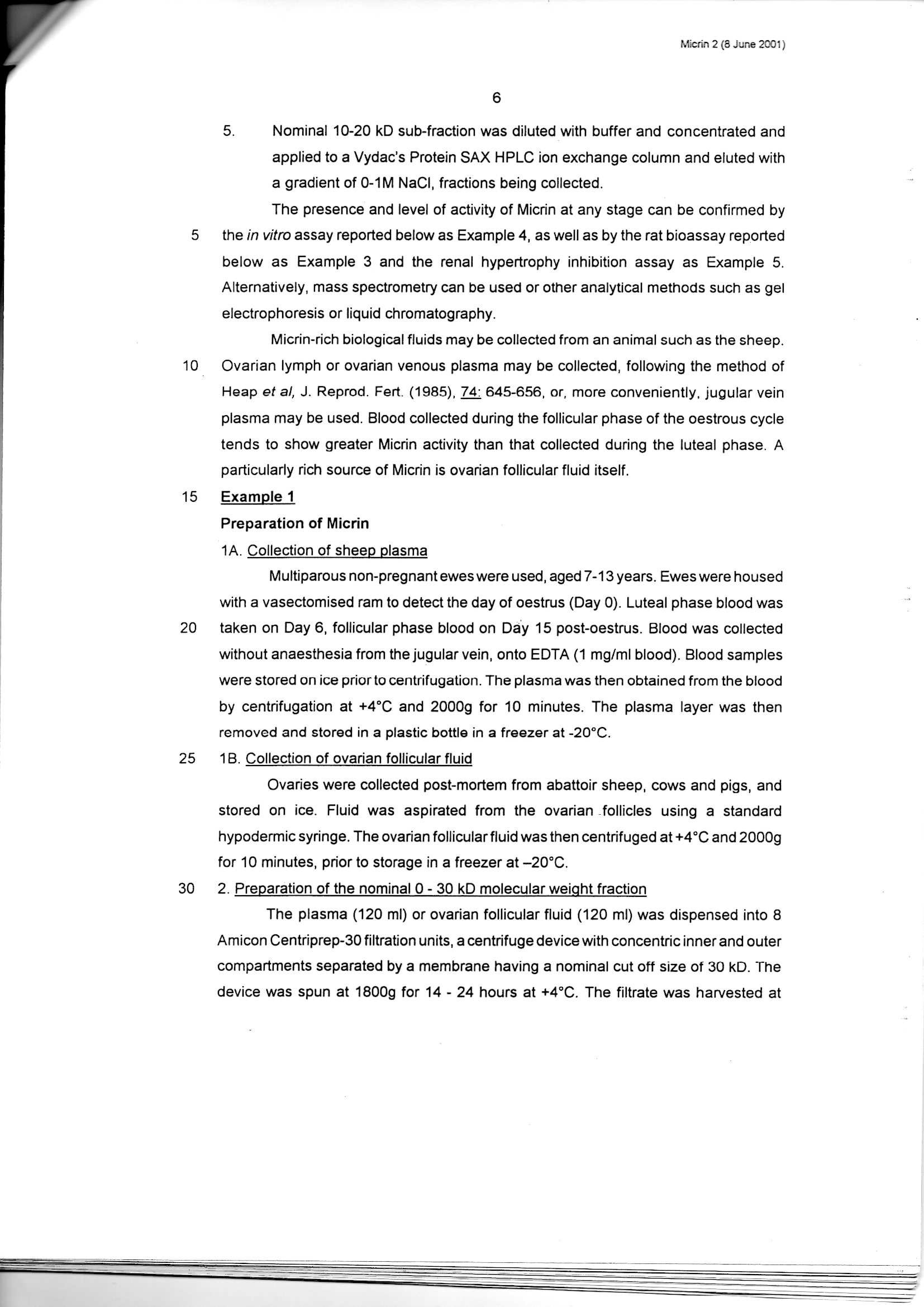


Note that the rat organometric assay in vivo, including that represented in the paper’s Fig. 1 used heparinised blood plasma from sheep. The assays in vitro in contrast used ovine plasma from blood harvested over EDTA, as this anticoagulant had been shown to lead to greater retention of cell-killing activity in the BMC assay (S1 Fig. 5). The assays in vivo used material subject to spin and gel filtration, prior to anionex chromatography (S1 Babraham Method). The assay in vitro used a simplified scaled-up purification involving ultrafiltration followed by anionex chromatography (S1 Sheffield Method).

Ovarian follicular fluid was more active in vitro than systemic blood plasma, but in view of practicalities, the latter was favoured in the form of jugular vein follicular phase EDTA plasma from OV-INTACT sheep.

**S2 Figure 10.** Ovariectomy diminishes micrin-like activity. Intact data: mean +/– SD

In the BMC assay in vitro use was made of jugular vein whole plasma from OVX sheep (n = 2) and ovarian vein whole plasma from OV-INTACT sheep (n = 22), the neat plasmas being putatively micrin-minus and micrin-plus, respectively. Cell survival after 48h (assessed by Alamar Blue) was normalised against one of the OVX samples as 100%, with the other at 98%. For the 22 OV-INTACT samples the mean +/– SEM was 25.7 +/– 4.7% (P<0.0001, two-tailed t test). In a follow-up experiment, a 3-30 kDa filtrate of ovine plasma taken from OV-INTACT animals killed 89% of BMC *in vitro*. Material from OVX sheep was associated with the demise of 17% of cells.

f1

f2

f3

f4

f5

f6

f7

f8

f9

f10

f11

f12

f13

f14

f15

f16

f17

f18

f19

f20

f21

f22

f23

0

20

40

60

80

100

120

f1

f2

f3

f4

f5

f6

f7

f8

f9

f10

f11

f12

f13

f14

f15

f16

f17

f18

f19

f20

f21

f22

f23

**HPLC fraction number**

**BMC survival as % of Control**

**S2 Figure 11.** Effect on BMC viability of anionex HPLC fractions. Cytotoxic activity is largely localised to Fractions 19 & 20.


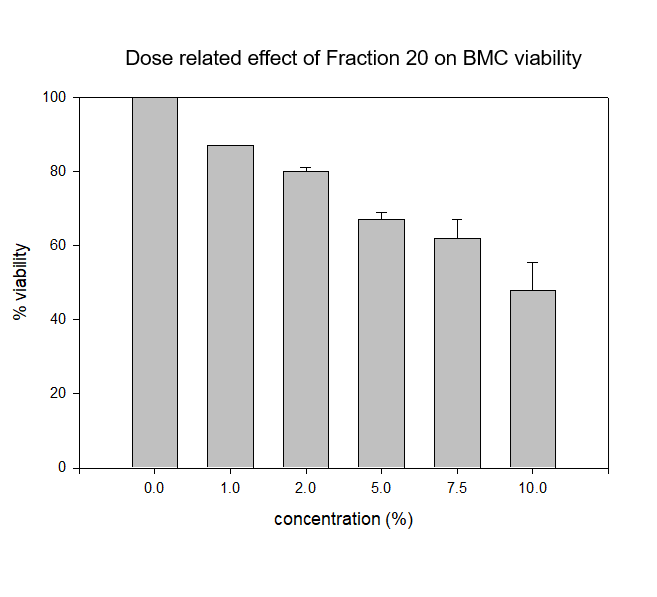


**S2 Figure 12** illustrates a typical dose-response curve for an anionex HPLC fraction highly apoptotic in vitro. ‘% viability’ = BMC survival as % of Control. ‘concentration (%)’ = percentage of cell medium comprising the HPLC fraction.

Reduction of BMC viability involves apoptosis, as judged by Annexin-V. HPLC Fractions 19 & 20 produced a significant increase in the number of apoptotic cells among BMCs *in vitro*. There was no significant effect on cell necrosis, suggesting that this is a specific physiological effect and not due to non-specific toxicity.

|  |  | % control 1 | % control 2 | % Fraction 19 | % Fraction 20 |
| --- | --- | --- | --- | --- | --- |
| Region 1 | Healthy cells | 26 | 38 | 17 | 10 |
| Region 2 | Apoptotic cells | 4 | 3 | 44 | 59 |
| Region 3 | Free nuclei | 15 | 17 | 3 | 0.4 |
| Region 4 | Necrotic cells | 55 | 41 | 35 | 30 |
| Region 6 | Pre-apop cells | na | na | 17 | 6 |

**S2 Table 6.** Effect of anionex HPLC fractions on BMC apoptosis as determined by flow cytometry.

Annexin-V-Fluos labels apoptotic cells by binding to phosphatidylserine on the cell membrane, permitting separation by flow cytometry. In the early stages of apoptosis the translocation of phosphatidylserine to the outer membrane occurs allowing the protein to bind. In this experiment the simultaneous addition of propidium iodide allows discrimination between necrotic and apoptotic cells.

Method (lab notes):

1. Approximately 1 x 10^6^ cells were treated for 72 h with the fractions.
2. The cells were then harvested using 0.2% EDTA and washed with PBS.
3. The cells were then resuspended in 0.1 ml staining solution (50 mM HEPES pH 7.4, anti-annexin-V-FITC, propidium iodide).
4. After 15 min, 0.4 ml HEPES was added and the cells analysed for apoptosis.
5. FITC was detected using 488 nm excitation filter and a 515 nm bandpass filter and propidium iodide detected using a 488 nm excitation filter and a >600 nm bandpass filter.

Apoptosis was confirmed by DNA end-labelling.

**S2 Table 7.** Effect of anionex HPLC fractions on BMC cell death as determined using the Boehringer Manheim cell death assay.

Apoptosis is accompanied by DNA fragmentation which generates mono- and oligonucleosomes which are tightly complexed with the core histones and are therefore protected from cleavage. The enrichment factor of the cytoplasm of apoptotic cells is due

to the build-up of mono- and oligonucleosomes from DNA degradation prior to membrane breakdown. Cam = campothecin, an established inducer of apoptosis, used here as a positive control. BKG = background.

Method (lab notes):

1. Cells are seeded in 48 well microtitre plates at a density of 15000 cells per well. The cells are then treated with the fractions for 48 h and then analysed as follows.
2. The microtitre plate is centrifuged for 10 min at 200g and the medium removed.
3. The cells are resuspended in 200 ul lysis buffer and shaken (on a plate shaker) for 30 min at room temperature.
4. The microtitre plate is then centrifuged for 10 min at 200g and 20ul of the extract transferred to the streptavidin coated microtitre plate.
5. 80 ul immunomix (prepared as described in the instructions) was added to each well.
6. This was incubated for 2 h at room temperature and then washed three times with incubation buffer.
7. 100 ul of pnp substrate was added to each well. The plate was incubated for 15 min and then read using 405 nm as the measurement wavelength and 490 as the reference wavelength.

-0.1

0

0.1

0.2

0.3

0.4

0.5

0.6

2500

5000

10000

20000

40000

**Cell Number**

**Absorbance**

**Micrin**

**Control**

**S2 Figure 13.** Effect of BMC plate density on inhibitory activity of Fraction 19.

BMC were plated out at increasing cell density and then the effect assessed on their viability of anionex HPLC Fraction 19. It was found that there was a stoichiometric relationship between the number of cells killed and the amount of fraction applied. ‘Absorbance’ = cell viability (Absorbance units).


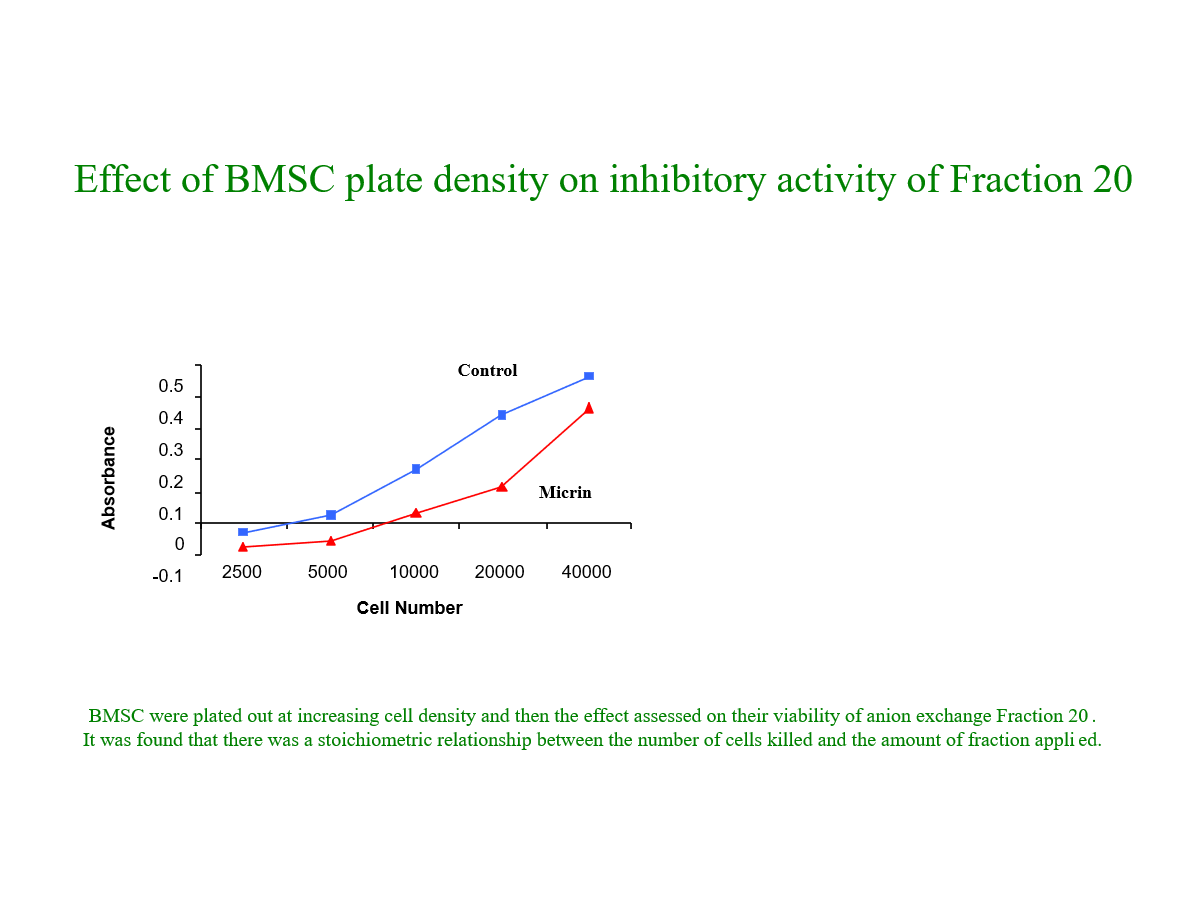


**S2 Figure 14.** Effect of BMC plate density on inhibitory activity of Fraction 20.

BMC were plated out at increasing cell density and then the effect assessed on their viability of anionex HPLC Fraction 20. It was found that there was a stoichiometric relationship between the number of cells killed and the amount of fraction applied. ‘Absorbance’ = cell viability (Absorbance units).

Tumour cells could also be induced to undergo apoptosis by the same HPLC fractions.

**S2 Figure 15.** Effects of anionex HPLC fractions on prostate cancer cells (PC3) and breast cancer cells (MCF7). Reduced viability has been demonstrated in other cancer cell line, e.g. DU145 prostate cells and MC6 breast cancer cells (Hart, 2000 [16]).

Confirming, micrin containing HPLC fractions reduce the viability of prostate cancer cells *in vitro* and also that of breast cancer cells.

| Treatment | Viability (% control) | |
| --- | --- | --- |
|  | PC3 (prostate cancer cell line) | MCF-7 (breast cancer cell line) |
| Micrin | 14.3 | 41.7 |
| Camptothecin | 43.9 | 56.2 |

**S2 Table 8.** Reduction of cancer cell viability, micrin versus camptothecin. Camptothecin, an inducer of apoptosis, was used as a positive control at 3 μM. Micrin was deemed to be present in subnanomolar quantities. Viable cells were quantitated after 48 hours using Alamar Blue. Induction of apoptosis was subsequently confirmed by Annexin V staining and flow cytometry.

Anionex HPLC Fractions 19 & 20 were shown to contain MS Candidate 7500. An example of a MALDI mass spectrum of Fraction 20 is shown as S3 Fig. 6.


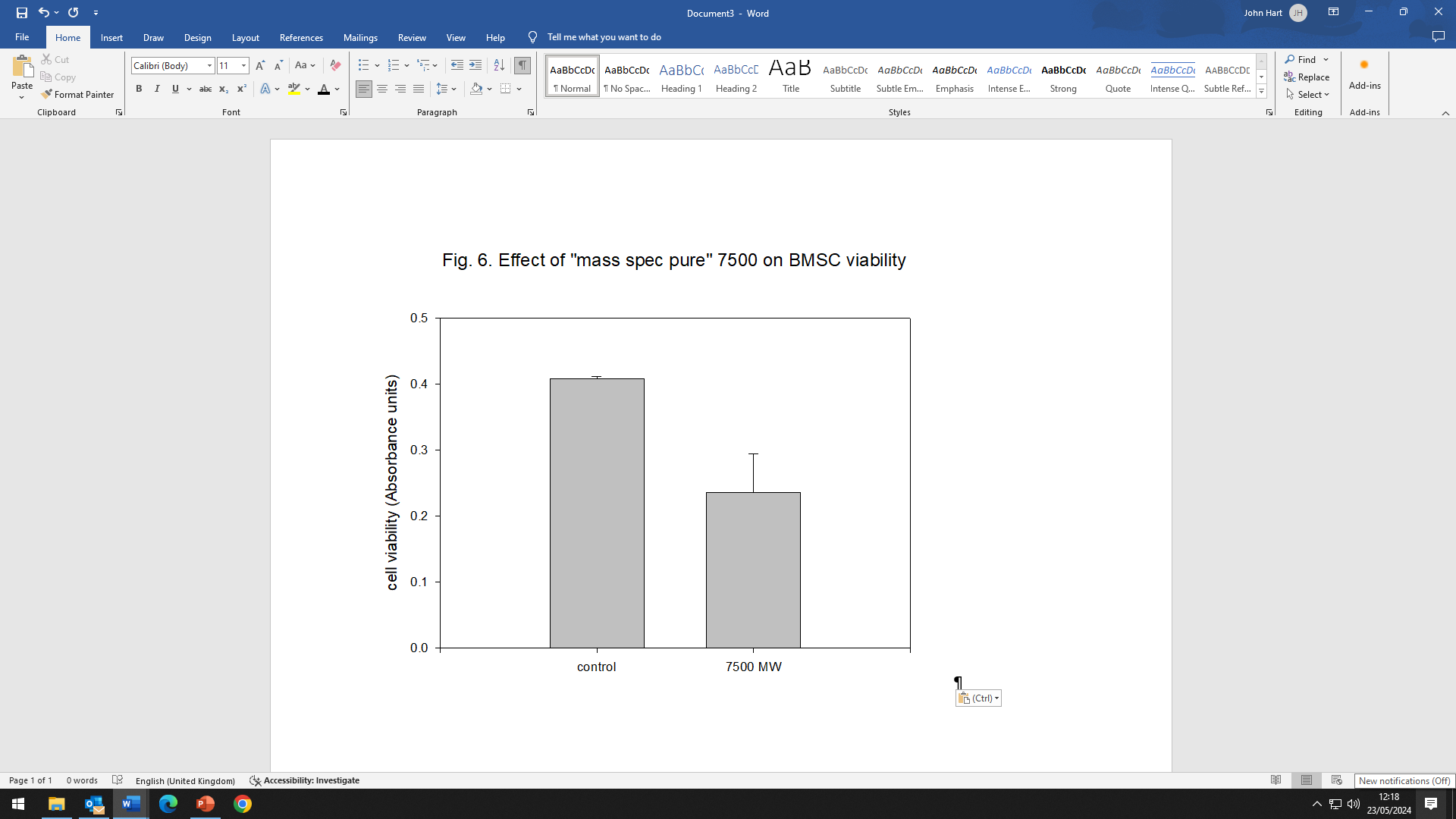


**S2 Figure 16.** Effect of ‘MALDI mass spec pure’ Candidate 7500 on BMC viability.

BMC were treated with anionex HPLC fractions containing Candidate 7500 or control fractions lacking Candidate 7500.

Ovine jugular vein blood serum was collected at different phases of the ovine oestrus cycle as described above and purified to anionex HPLC fractions using the Babraham Method (S1). There was a peak of Candidate 7500 in the follicular phase with a second smaller peak in late luteal (S2 Fig. 17), with the follicular phase results shown with earlier fractions in S2 Fig. 18.


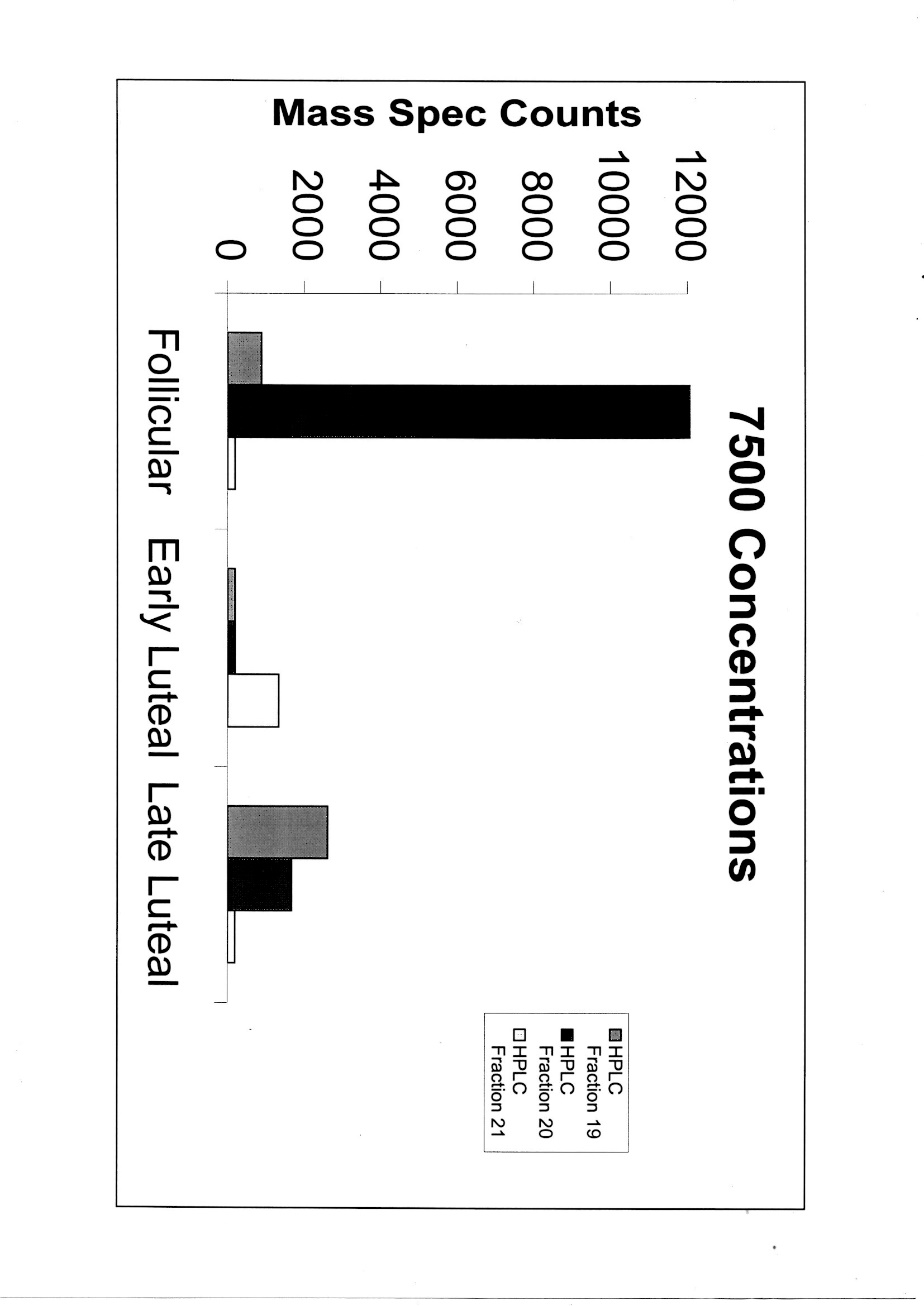


**S2 Figure 17.**


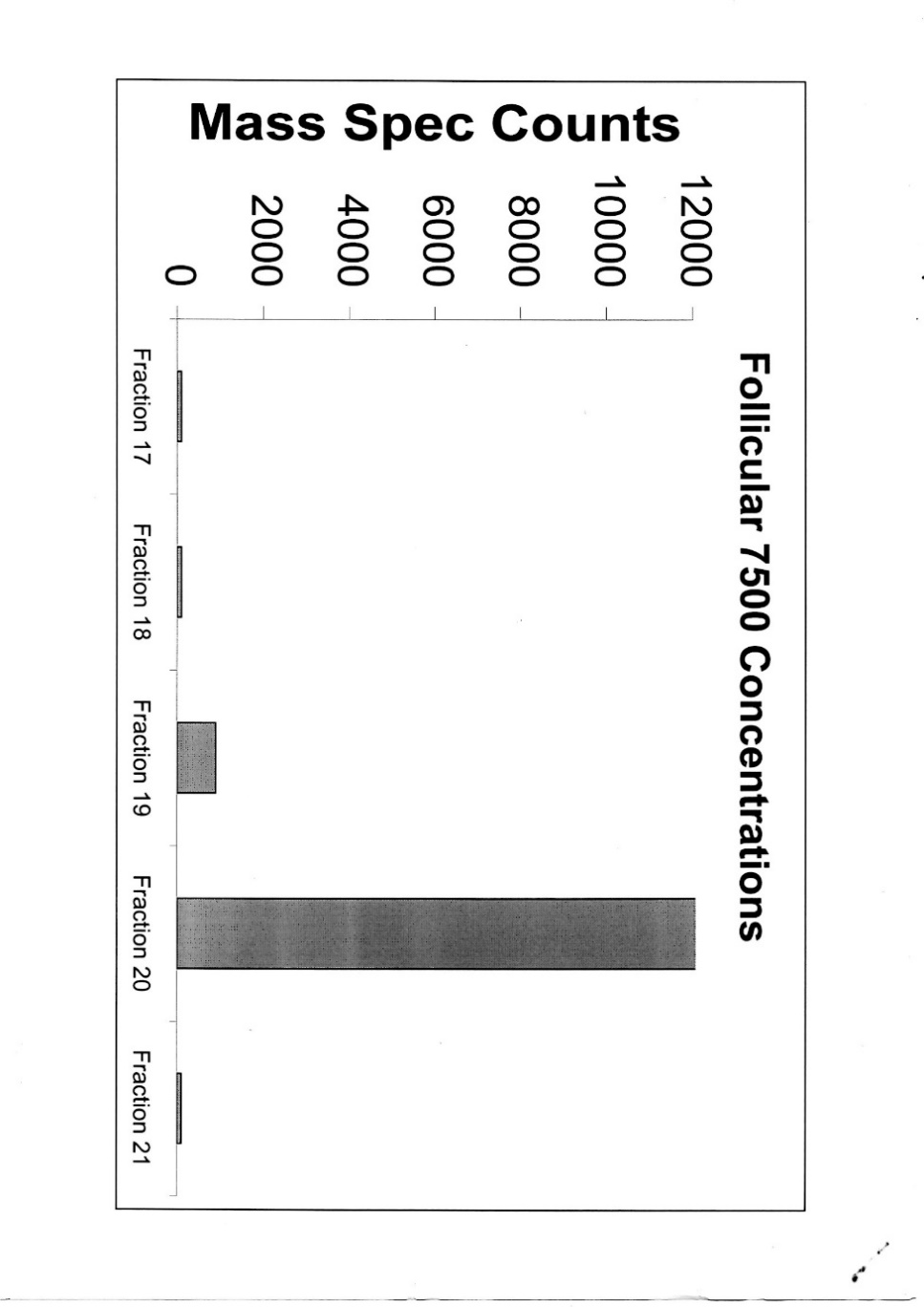


**S2 Figure 18.**

| Fraction No | BMC viability % |
| --- | --- |
| 1 | 98 |
| 2 | **72** |
| 3 | 79 |
| 4 | 111 |
| 5 | 92 |
| 6 | 91 |
| 7 | 81 |
| 8 | 88 |
| 9 | 94 |
| 10 | 85 |
| 11 | 93 |
| 12 | 86 |
| 13 | 93 |
| 14 | **71** |
| 15 | 73 |
| 16 | 90 |
| 17 | 77 |

**S2 Table 9.** Example of double dip (low salt/high salt) effect of anionex HPLC fractions on BMC viability, with lowest values emphasized. Both low and high salt fractions contain Candidate 7500, as described in the paper.

As the high salt form proved easier to obtain, the focus switched to that. A concern was that effects in vitro might be due to high salt rather than an expression of the sought-for bioactivity. This concern was allayed by the effects of desalting, which left the activity unchanged, as illustrated by the following graphic.

**S2 Figure 19.** The effects on BMC viability of an extended series of anionex HPLC fractions.

**S2 Figure 20.** Micrin inhibits the development of cardiac myocyte hypertrophy *in vitro*. This is a model in which neonatal cardiac myocytes are induced to increase in size and to express atrial natriuretic factor, ANF, a marker associated with cardiac hypertrophy. Micrin inhibited ANF expression by about 40%. Neonatal cardiac myocytes were prepared from 3-day-old Wistar rats according to a published method (Hart, 2008 [18]) and treated with anionex HPLC Fractions 28/29 (containing Candidate 7500) or Fractions 23/24 (negative control, devoid of Candidate 7500). Total RNA was prepared from cells 24h after the addition of FCS/micrin, with ANF, and glyceraldehyde 3-phosphate dehydrogenase (GADPH, house-keeping gene control) estimated by RTPCR. Results (vertical axis) were expressed as a ratio of ANF:GAPDH mRNA expression values.

**S2 Figure 21.** Effect of immunoprecipiated sheep plasma (3-30 kDa) filtrate (SPF ImmP, putatively ‘micrin minus’) on MDA-MB231 cell growth in vitro compared with SPF, putatively ‘micrin plus’. *P = <0.05, paired t test. Alamar Blue cell viability assay. A rabbit polyclonal anti-EPL001 antibody was used for immunoaffinity separation.

To quote Hart 2008 [18], [with interpolations for clarity in square brackets]:

‘Partial removal of micrin was achieved using the antiserum to the micrin peptide fragment [EPL001: MKPLTGKVKEFNNI], an immunoprecipitation approach, as is now described. SPF was immunoprecipitated with antibody and protein A with the 2nd bleed of antiserum diluted 1 in 10. Cellular growth of MDA-MB231 breast cancer cells in response to SPF and immunoprecipitated SPF (SPF ImmP) was assessed using a standard Alamar Blue assay, of the kind described below for prostate and breast tumour cells. The inhibitory effect of SPF on cell viability was significantly inhibited by prior immunoprecipitation (Figure […]; the asterisk denotes a significant difference, p<0.05, paired t-test). Neither PBS nor normal rabbit serum (10%), substituted for SPF, affected cellular growth of these MDA-MB231 cells.’


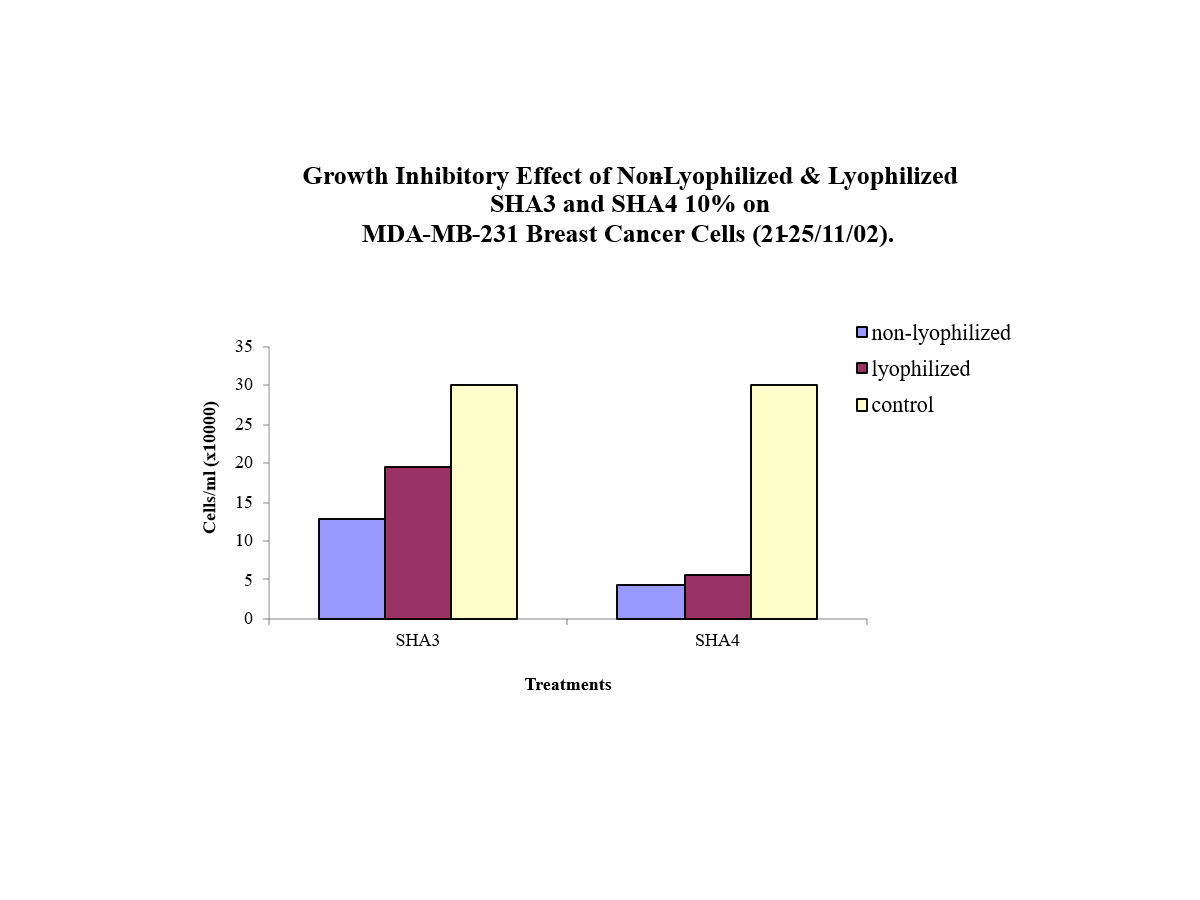


**S2 Figure 22.** Growth inhibitory effect of non-lyophilized and lyophilized (freeze-dried) SHA3 and SHA4 at a concentration of 10% on MDA-MB231 breast cancer cells in vitro. SHA3 & SHA4 = batches of early eluting (low salt) anionex HPLC fractions, stored in liquid form at -20 °C or lyophilized and stored at room temperature for several days. MDA-MB231 breast cancer cells were plated overnight in 24-well culture plate (1.5 × 10^4^ cells/well), then stimulated with 10% of SHA3 and SHA4 pooled fractions 1-10 (proportional to the volume of the medium in each well, i.e.110μl of each fraction or lyophilized equivalent was added into 1ml medium). Cells were counted by Coulter Counter after 72h. Lyophilization, i.e. freeze-drying of anionex fractions for sample preservation, *reduced* retention of cell-killing activity.

Angiogenesis was assessed in vitro by a commercial contractor (TCS Cellworks, Buckingham, UK). The wording of the original report has been retained, with minor modifications for clarity. The angiogenesis assay is based on the co-cultivation of fibroblasts and human umbilical vascular endothelial cells (HUVECs) and the measurement of venule formation. The source material was bovine ovarian follicular fluid subject to spin filtration, gel fitration and anion exchange chromatography (S1 Babraham Method), subject to MALDI MS validation.

T1 = control (combined anionex Fractions 24 & 25, lacking Candidate 7500).

T2 = test (combined anionex Fractions 28 & 29, containing Candidate 7500)

The spectra for the four fractions are reproduced after the report, together with an MS analysis. It was found that anionex fractions containing Candidate 7500 inhibited capillary formation by nearly 60% over a 14-day study period. T2, containing Candidate 7500, was thus highly anti-angiogenic. T1 was pro-angiogenic.

**Title Evaluation of the pro- or anti-angiogenic activity of test compounds supplied by Endocrine Pharmaceuticals**

**Objective(s) To assess the effects of test compounds in the TCS Human Angiogenesis Kit (AngioKit)**

**Summary** There were interesting results recorded for both of the test compounds. (24 +25) caused stimulation whereas (28+29) inhibited venule formation, the latter effect being comparable to that elicited by suramin (20μM), a well-known anti-angiogenic compound.

**Test Article**

Test compounds were received from [a UK university]. There was insufficient material for duplicate assays so compounds 24 and 25 were combined together (T_1_) and compounds 28 and 29 (T_2_) were also combined together. Compounds were then diluted in AngioKit medium and stored at 4°C for the duration of the experiment. The medium in each well was removed and replaced with medium containing the appropriate test compound on day 1. This operation was repeated on days 4, 7 & 10.

**Materials**

TCS AngioKit Used: Batch No. 24632T Plate No. C010629-

**Methods**

Each test concentration was tested in one well.

Controls used were:

1. Untreated control. – medium alone
2. Positive Control – medium containing 2ng/mL VEGF
3. Negative control – medium containing 20μM suramin.

**Image Recording & Analysis of Results**

Comparison of venule length was conducted using the ‘AngioSys’ image analysis system developed specifically for the analysis of images produced using the AngioKit. Four images taken from predetermined positions within each well were recorded. Since the test compounds were analysed in single wells in this study, each concentration of test compound therefore yielded 4 images for analysis. Images were always taken from as close to the centre of each quadrant as possible (figure 1).

4

3

1

2

4

3

**S2 Figure 23.** The order in which images were recorded

**Statistical analysis**

All statistical analyses were carried out using the Stat 100 programme from BIOSOFT Ltd. using ANOVA and Duncan’s Multiple Comparison Test to measure differences between the test compounds with the untreated control values.

**Results**

**S2 Figure 24.**

**S2 Table 10.** Measurements of venule length (pixels) following treatment

Control levels of total venule length are shown in S2 Table 9 and are consistent with the predicted response to untreated and negative control factors. The lack of response, compared to untreated controls, due to the addition of 2ng/mL VEGF is unusual.

There was a statistically significant decrease in tubule length caused by addition of compound T_2_. There was also a statistically significant increase in tubule length caused by addition of compound T_1._

**Discussion**

There were interesting results recorded for both of the test compounds. T_1_ caused stimulation whereas T_2_ inhibited venule formation, the latter effect being comparable to that elicited by suramin (20μM), a well-known anti-angiogenic compound. While these results are indicative of compounds that could have a very active influence on the angiogenic process it must be remembered that this experiment was conducted in only one well of an AngioKit for each of the compounds tested. As such it would be advisable to repeat this assessment with sufficient material to conduct dose response studies. This is particularly true for compound T_2_, which appeared to have such a dramatic effect.

The results of the angiogenesis study have been presented in a patent: Hart, 2008 [18], Fig 6 therein.

**S2 Figures 25-28** are on the following pages, showing the mass spectra relating to T1 (Fractions 24 & 25) & T2 (Fractions 28 & 29) in the angiogenesis assay in vitro. Legends and analysis follow the figures.


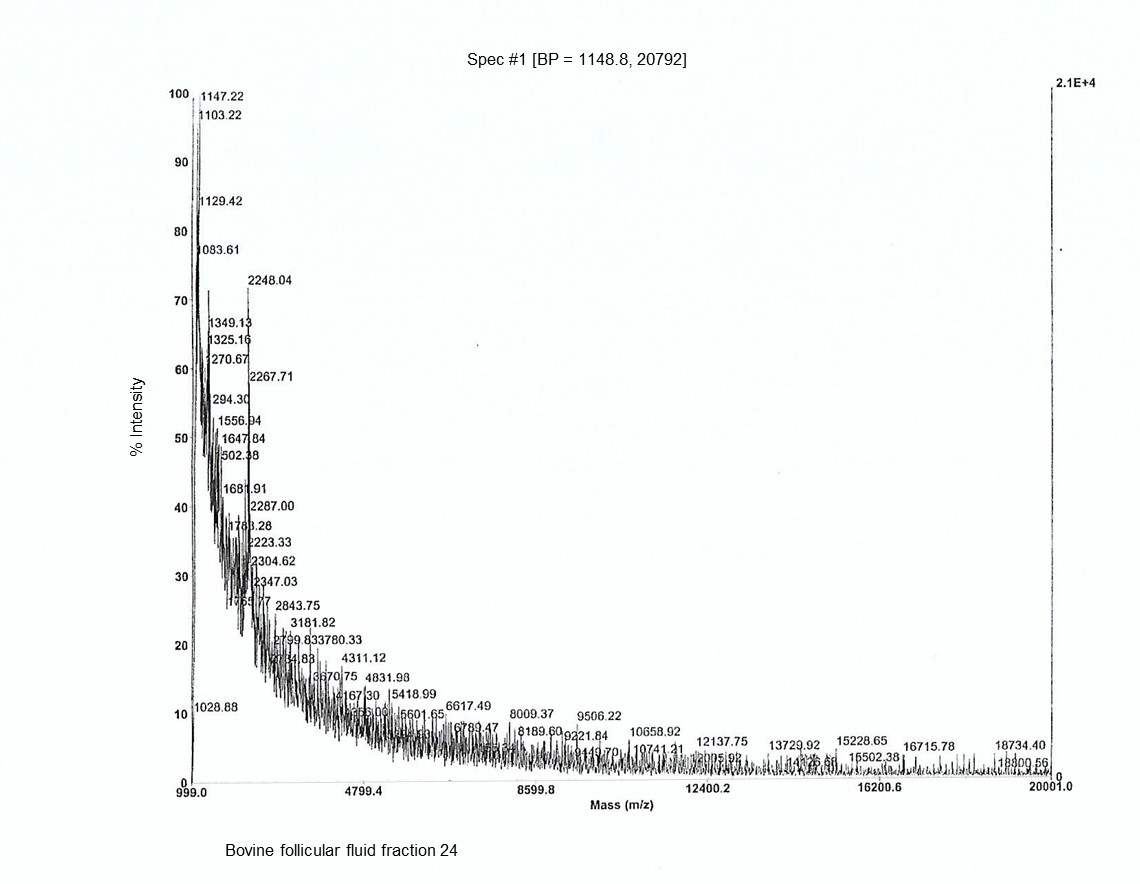


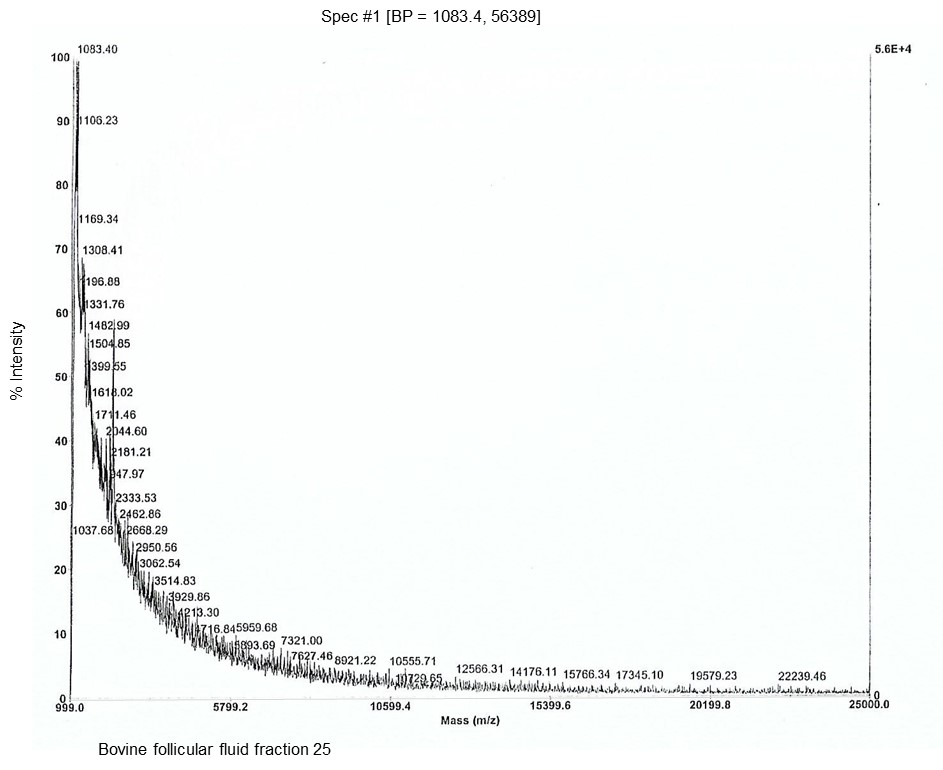


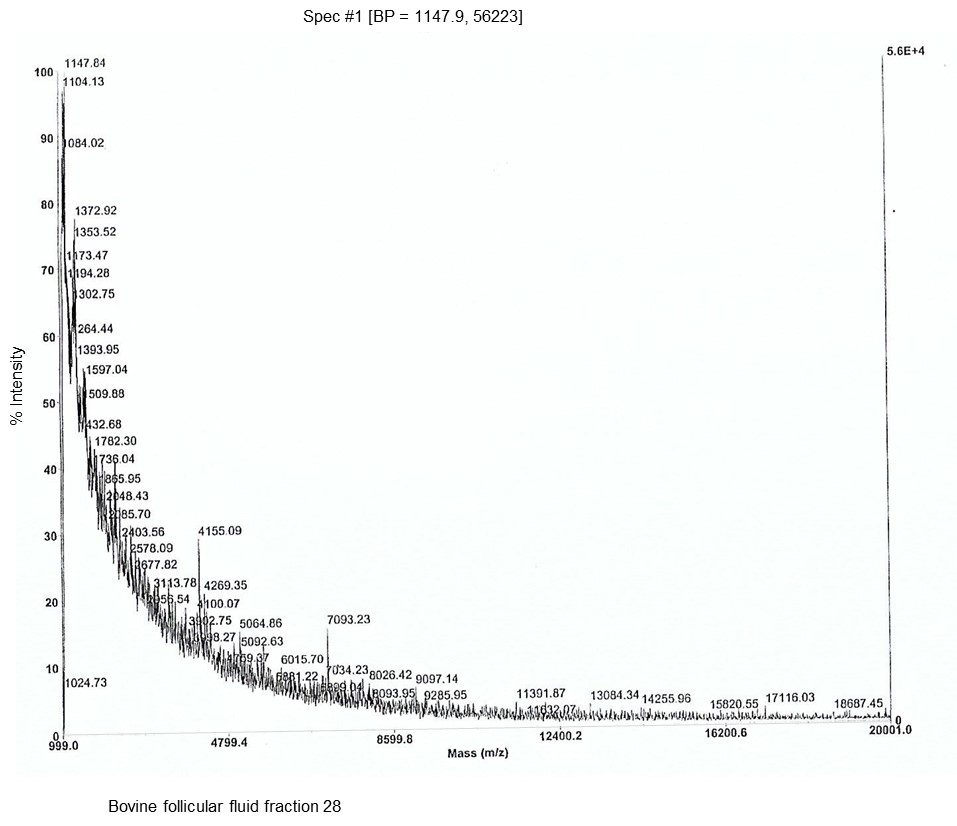


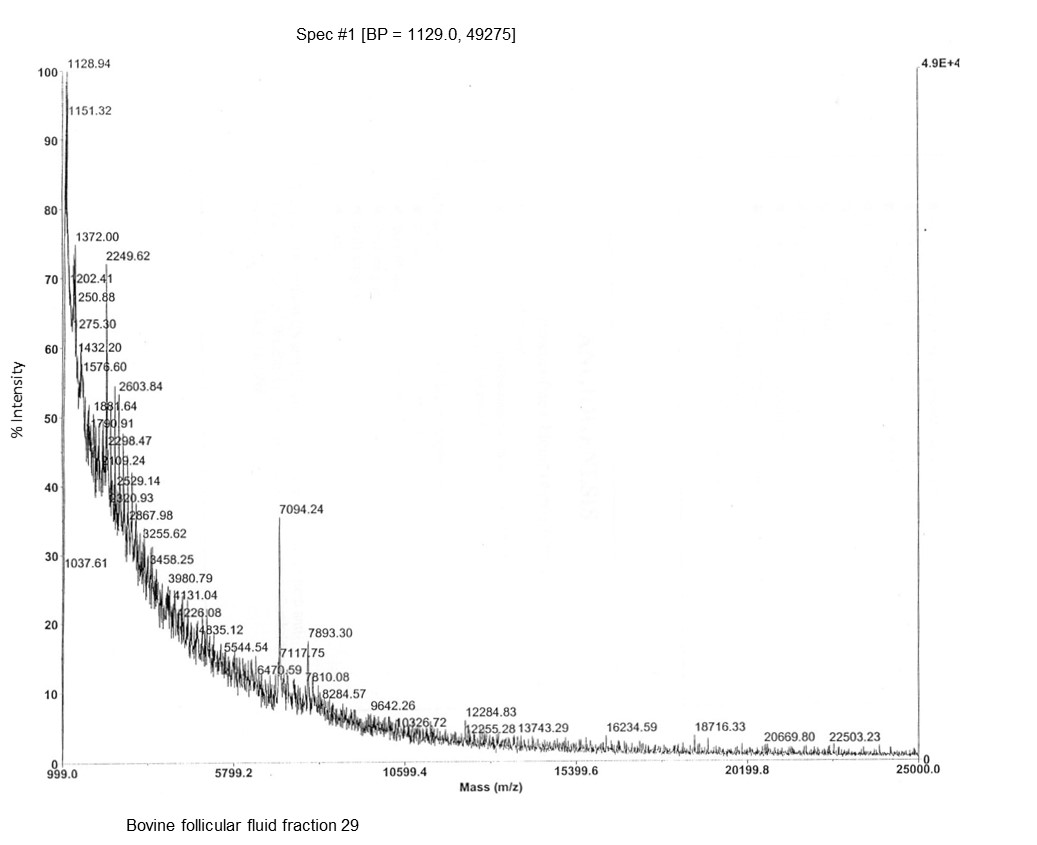


**S2 Figure 25 Legend.** Bovine ovarian follicular fluid anionex Fraction 24, obtained by the Babraham method of purification (S1). The characteristic lefthand slopes in MALDI spectra are interpreted in the paper as SgII-70 fragment ramps. Within this interpretation, matches to prominent peaks in the ramp are as follows:

1148 base peak (bSgII-70 **11mer** match 1153 in S3 Table 3)

2248 (**20mer** match 2252)

**S2 Figure 26 Legend.** Bovine ovarian follicular fluid anionex Fraction 25, obtained by the Babraham method of purification (S1). The characteristic lefthand slopes in MALDI spectra are interpreted in the paper as SgII-70 fragment ramps. Within this interpretation, a match to the most prominent peak in the ramp is as follows:

1083 base peak (bSgII-70 **10mer** match 1079 in S3 Table 3)

**S2 Figure 27 Legend.** Bovine ovarian follicular fluid anionex Fraction 28, obtained by the Babraham method of purification (S1). The characteristic lefthand slopes in MALDI spectra are interpreted in the paper as SgII-70 fragment ramps. Within this interpretation, matches to the most prominent peak in the ramp and other peaks are as follows:

1147 base peak (bSgII-70 **11mer** match 1153 in S3 Table 3)

4155 (**36mer** match 4159)

7093 (**62mer** next-integer match 7092)

**S2 Figure 28 Legend.** Bovine ovarian follicular fluid anionex Fraction 29, obtained by the Babraham method of purification (S1). The characteristic lefthand slopes in MALDI spectra are interpreted in the paper as SgII-70 fragment ramps. Within this interpretation, matches to prominent peaks in the ramp and other peaks are as follows:

1129 base peak (bSgII-70 **10mer** match 1133 in S3 Table 3)

2249 (**20mer** match 2252)

7094 (**62mer** match 7092)

7893 (**69mer** integer match 7893)

Ten peaks have been highlighted from the four MALDI spectra of S2 Figs. 24-27. These can be analysed together within a decremented bSgII-70 model.

∑Observed (n = 10) = 35239

∑Matches = 35258

∑Observed/∑Matches x100 = 35239/35258 x100 = **99.95%**

(A further example of a MALDI spectrum from an anionex fraction of bovine ovarian follicular fluid is shown as S3 Fig. 1, where ∑Observed (n = 5)/∑Matches x100 = **99.94%**.)

Divergent results as between the test and ‘control’ groups are especially intriguing.

Fractions 28 & 29 were combined to form T2, which was *anti-angiogenic* to a significant extent. These fractions contained Candidate 7500, including an integer and a next-integer match to items in the bovine decremented bSgII-70 MS data analysis of S3 Table 3. On the basis that ‘Candidate 7500 = bSgII-70 = micrin’, this is micrin acting as an agonist inhibitor of angiogenesis.

Fractions 24 & 25 were combined to form T1, which was *pro-angiogenic* to a significant extent. These fractions did not contain Candidate 7500 but are analysed using S3 Table 3 to have contained smaller N-terminal fragments of Candidate 7500/bSgII-70. The unrecognised presence of micrin in sera used for example in cell culture media has been considered (Hart, 2008 [18]). The fragments may be acting to antagonise the native hormone, achieving disinhibition.

The angiogenesis work faltered in bewilderment. ‘Candidate 7500’, yes, but what did it mean that there were different MALDI peaks in the *m/z* 7000s and what of the low MW species providing the base peaks in intensity plots? The answer offered in the paper is this: all are different-length N-terminal fragments of a secretogranin II derivative, SgII-70.

**Substituent study to simulate effects of covalent crosslinking on Edman degradation**

Edman Nonsequentialism, i.e. aberrant N-terminal sequencing, is described in the paper and is deemed to have resulted from a spiralised charged target polypeptide disintegrating within the machine’s reaction chamber, with the anomalous rupture of peptide bonds and aberrant availability of free amines out of sequence under the influence of steric factors; all of this occurring in a relatively reproducible and informative fashion. Nonsequentialism proves to be analysable in detail to obtain EPL001 from sSgII-70 (Hart et al, 2022 [22]). Consideration has been given to the effect on Edman sequencing of proposed intramolecular transamidation K-Q crosslinking, using a substituent technique. The plain master peptide EPL001, MKPLTGKVKEFNNI, was read faithfully by an Edman machine (Applied Biosystems Procise 494HT at Alta Bioscience, Redditch, UK). EPL001 was immobilized inside the sequencing instrument by blotting the peptide onto a PVDF membrane. The bound peptide was then reacted with the Edman reagent, phenylisothiocyanate, at high pH, with the altered N-terminal residue then subject to cleavage with anhydrous acid. The cleaved product was converted to its stable phenylthiohydantoin with aqueous acid, then analysed using the on-board HPLC. Identification of amino acids was achieved by comparing HPLC elution times with a standard mixture. The process was then repeated for the next residue and so on. Crosslinking was simulated using substituent amino acids added to sSgII-14 as follows, to see if any sequence like EPL001 could be generated: the side chain of Ac-E-OMe was linked to the side chain of lysine and the side chain of Ac-K-OMe was linked to the side chain of glutamic acid, where ‘Ac’ means protective N-terminal acetylation and ‘Me’ means protective C-terminal methylation. The results were thus, with ‘x’ representing an unknown component: (a) MLK(Ac-E-OMe)TGEKPVKFNNI was read as MLxTGEKPVKFNNI; (b) MLKTGE(Ac-K-OMe)KPVKFNNI was read as MLKTGxKPVKFNNI; and (c) MLK(Ac-E-OMe)TGE(Ac-K-OMe)KPVKFNNI was read as MLxTGxKPVKFNNI. Summarising, nothing like EPL001 eventuated in Edman from side-chain-augmented sSgII-14. The Edman machine classed the cleaved backbone residue connected to a substituent amino acid as eluting anomalously, registering an x. In the case of a true intramolecular crosslink the side connection would be to an amino acid from elsewhere in the peptide chain, which might mean that nothing is released for HPLC analysis. Another x. The results of the substituent study uphold the undeviating robustness of Edman degradation as a sequencing technique and suggest there is more to the apparent misread than intramolecular covalent crosslinking, which crosslinking in any case the paper dismisses as absent from sSgII-70.

**ELISA prototype**

Men tend to have more analyte in plasma than women:

€ 8.6-59.0 nM

 3.5-18.4 nM

n = 5, t test, P = <0.05, significant

**S2 Figure 29.** ELISA prototype results snapshot.

There follows an extract from a patent, in the form of Hart, 2008 [18], describing the prototype ELISA assay, in which SEQ ID NO: 9 = EPL001 (MKPLTGKVKEFNNI). The primary antibody was an anti-EPL001 rabbit polyclonal antibody described in Hart et al, 2017 [17].

‘6b. Enzyme linked immunosorbent assay (ELISA)

Direct measurements of micrin levels have been made using an ELISA, now described, this being a typical example of an immunoassay which can be used for micrin measurements. The peptide fragment of micrin (SEQ ID NO: 9) was adsorbed (for 16 hours at 4°C) onto the wells of a 96-well plate. Standards (i.e. dilutions of micrin peptide fragment) or samples (plasma or other body fluids or tissue extracts) were mixed with a fixed concentration of antibody for 16 hours at 4ºC. This mixture was then added to the blocked, washed and drained coated wells in triplicate and incubated for 2 hours at room temperature. In this technique, any unbound antibody in the mixture can bind to the immobilised micrin peptide fragment on the plate. The amount of free antibody is dependent on the amount of micrin in the mixture. Thus, the more micrin in the sample or standard, the less free antibody available. The plates were then washed and drained, following which an appropriate concentration of secondary (anti-rabbit) antibody was added and incubated for 1 hour at room temperature. After washing and draining the plates, TMB One (Promega) was added to each well (10 mins) followed by sulphuric acid. The colour reaction was read immediately in an ELISA Plate reader at 620 nm. The above procedure is in accordance with standard ELISA practice (Nagai, R*.*, *J. Biol. Chem*., 277(50):48905-48912, 2002), and used Reactibind microtitre plates as recommended for this type of competitive assay (from Pierce Biotechnology, Rockford, Illinois).

An ELISA assay of 5 male and 5 female plasma samples indicated that plasma micrin levels for males were in the range 8.6-59.0 nmol and for females 3.5 -18.4 nmol, the difference being statistically significant (p<0.05 Student’s t-test).’

[ENDS]
