# Supplementary material for: Detecting the body’s reproductive hormonal brake against tissue overgrowth: Micrin/SgII-70
Source: PLoS One. 2026 Mar 2;21(3):e0340980. doi: 10.1371/journal.pone.0340980 (PMC12952585; doi:10.1371/journal.pone.0340980)
Supplement: S3 File — https://doi.org/10.6084/m9.figshare.27110284.v2. This project presents MALDI-TOF MS data of the polypeptide Candidate 7500 and describes it as a novel proteoform of the neuroendocrine prohormone secretogranin II, dubbed sSgII-70. (DOCX) [file pone.0340980.s003.docx]

**Supplementary Information 3 (S3)**

**Mass Spectrometry**

S3 is provided in support of ‘Detecting the body’s reproductive hormonal brake against tissue overgrowth: micrin/SgII-70’ by Hart JE, Davies KG, Mundy CR, Hart AC, Howlett DR & Newton RP (2024). Corresponding author email: [k.davies@herts.ac.uk](mailto:k.davies@herts.ac.uk)

Candidate 7500’s multiple peaks in the range *m/z* 7-8000 in MALDI-TOF MS are variant ions of a unitary polypeptide, namely sSgII-70. This view informs the following analysis, which will argue that the observed heterogeneity in MALDI MS is due to (a) C-terminal truncation (i.e. loss of aa residues from the C-terminus by peptide bond cleavage) and (b) water losses in variable degrees.

The core data fittings involve specimen MALDI spectra from ovine and bovine purification runs, analysing a dozen or so MS peaks from each species in the range *m/z* 7-8000. C-terminal and N-terminal truncations have been tried, based on the SgII-70 sequence deductions, with C-terminal truncation favoured for tightness of fit between observations and matches. The data fitting exercise is then followed by a series of bespoke analyses.

In MALDI the sample is mixed with an organic matrix to aid desorption and ionization. The matrix is a low molecular weight, UV absorbing, acid. Both matrix and sample are dissolved in 50/50 0.1% trifluoroacetic acid/acetonitrile. Approximately 1 µl of the matrix/sample mixture is spotted onto a MALDI plate and allowed to co-crystallise in a single spot. A laser imparts energy. The matrix heats up rapidly and sublimates, along with the sample molecules co-crystallised with it at much lower concentration. The singly protonated positive ions so produced (MH^+^) ablate into the vacuum and are accelerated in an electrical field before being reflected between magnets, for separation on the basis of mass-to-charge ratio (*m/z*). In a Time-of-Flight (TOF) setup the ions are accelerated to equal energy for mass measurement. MALDI is tolerant of salts and other background molecules, simplifying sample preparation. It is regarded as a soft ionization method, with indirect energy transfer from the matrix to the analyte. This avoids sample decomposition, a perception running counter to the rampant facile fragmentation described in the present report.

In the representative spectra of the paper’s Figs. 2-5, which were obtained at different times from different ovine materials (ignoring the bovine top panel of Fig. 4) on different instruments (notably a PE Biosystems Voyager DE PRO System 6022 at Babraham), there are 12 main mass-annotated *m/z* 7-8000 peaks, as per the paper’s Table 1. These have a mean value of 7593.42. All of these peaks are separated from selected other peaks by integer masses of water molecules (18.015 Da x n, at average isotopic mass): 7930.24 – 7878.47 = 51.77 = 18.015 x **2.88**; 7878.47 – 7805.98 = 72.49 = 18.015 x **4.02**; 7709.72 – 7583.64 = 126.08 = 18.015 x **7.00**; 7709.42 – 7583.64 = 125.78 = 18.015 x **6.98**; 7628.30 – 7484.53 = 143.53 = 18.015 x **7.98**; 7583.64 – 7565.93 = 17.71 = 18.015 x **0.98**; 7583.64 – 7561.42 = 22.22 = **1.23**; 7484.53 – 7161.09 = 323.44 = 18.015 x **17.95**. The gap between the highest and lowest main peaks is equivalent to 46 water molecules, thus: 7930.24 – 7102.24 = 828.00 = 18.015 x **45.96**. The fusion of amino acid sidechains within the polypeptide could be at issue here, in variable degrees, with the release of water molecules, or there could be water-releasing fragmentation, as in MS/MS, except involving post-source decay not collisional dissociation. Note though that the fourfold water difference at 72.49 could be an alanine residue at 71.08 (water reduced) and the two sevenfold differences of ~126 could be Q or K at ~128. The eighteenfold water difference of 323.44 could be a loss of three or four residues and the forty-sixfold water difference could be a lost chain of ~7 amino acids. All this hints that besides varying degrees of water loss there may be variable numbers of residues present.

The aa sequence of the proposed factor sSgII-70 has been deduced as:

_1_MLKTGEKPVFKRTNEMVEEQYTPQNLATLESVFQELGKLTGPNNQKHERADEEQKLYTDDEDDIYKANNI_70_

This polypeptide primary structure is exhibited in the paper’s Fig. 11, with evidential support. The predicted mass of the intact 70mer is 8175.01 Da (Expasy Compute pI/Mw, as cited in the paper’s references; using average isotopic masses in all analyses). The protonated version of this, MH^+^, is 8176.02, with the addition of a hydrogen ion at 1.008 Da. Such a peak has not been observed in any modality of MS with any starting material, though a 70mer monomer-by-deduction has been described in a putative quadruple homodimer, 2+2+2+2 (S3 Fig. 10/S3 Table 11): peak of 64974.65 ÷ 8 = deduced monomer of 8121.83 (**70mer** S3 Table 1 integer match 8121.97, triply water reduced).

Key spectra are the paper’s Figs. 3 & 5. The 7-8000 peaks therein must relate to incomplete sSgII-70 sequences, in the current analysis, yet Edman data (Applied Biosystems Procise; see the paper’s references) from the Fig. 3 sample yielded anionex Beale 4 (S1 Table 1, SEQ ID NO: 4): xxPxxxxVxxFNxx. Meanwhile, gel Band 1 of Fig. 5 in an earlier purification run delivered the EPL001 sequence: MKPLTGKVKEFNNI (S1 Table 1, SEQ ID NO: 3). Methionine is at one end of sSgII-70, NNI at the other. The sequencing data support the view that MALDI causes residue losses artefactually. These could occur from the N terminus, the C terminus or both. Internal losses are unlikely as these would lead to minor peaks additively equivalent to the master molecule. Such pairings have not been seen, though a potential 42mer fragment of sSgII-70 at 4785.16 can be seen in the paper’s Fig. 2 (discussed in S1). Potentially explaining the MS heterogeneity so far, then, are variable residue losses and varying degrees of water loss.

C-terminal and N-terminal truncation models are based on the sequence deduction for SgII-70, successively decremented into 69mers, 68mers and so on downwards, by losses at either end. The molecular weight for a neutral polypeptide of each size has been determined using Expasy Compute pI/Mw. To obtain a protonated form (MH^+^) of the *intact* 70mer a hydrogen ion is added, at 1.008 Da, as described above. Calculations for decremented forms are based on breakages at peptide bonds, C-N peptide bonds being weaker than C-C bonds. For the 69mer and below in the C-terminal truncation analysis, there is then a molecular weight subtraction of 17.007 Da, being the mass of a hydroxyl group (OH). For the 69mer and below in the N-terminal truncation analysis, there is a molecular weight subtraction of 1.008 Da, being the mass of a hydrogen atom. By these means is provided M, in both truncation series. This is then protonated (+1.008) to give predicted masses in the form of MH^+^, which the MS detector could potentially ‘see’. Water decrements across the tables involve successive reductions by 18.015 Da (H_2_0).

**C-terminal truncation** in the *ovine* 7-8000 analysis (S3 Table 1, being an extended version of the paper’s Table 1) delivers a mean deviation of **2.66** at a mean error (i.e. difference as a percentage of match) of **0.035%**; seven integer or next-integer matches (**58**%); ∑Observed (n = 12) 91120/∑Calculated Matches 91113 x100 = **100.01**%; and a chi-squared P = 0.024 (S3 Table 5).

**N-terminal truncation** in the *ovine* 7-8000 analysis (S3 Table 2) delivers a mean deviation of **3.10** at a mean error of **0.041%**; three integer or next-integer matches (**25**%); ∑Observed (n=12) 91120/∑Calculated Matches 91129 x100 = **99.99**%; and a chi-squared P = 0.022 (S3 Table 6).

**C-terminal truncation** in the *bovine* 7-8000 analysis (S3 Table 3) delivers a mean deviation of **2.99** at a mean error of **0.040%**; two integer or next-integer matches (**15**%); ∑Observed (n = 13) 97097/∑Calculated Matches 97117 x100 = **99.98**%; and a chi-squared P = 0.027 (S3 Table 7).

**N-terminal truncation** in the *bovine* 7-8000 core analysis (S3 Table 4) delivers a mean deviation of **5.13** at a mean error of **0.069%**; one next-integer match (**8**%); ∑Observed )n = 13) 97097/∑Calculated Matches 97073 x100 = **100.03**%; and a chi-squared P = 0.057 (ns) (S3 Table 8).

**Summarising:** using end-truncation models based on SgII-70 predicted sequences, C-terminal residue losses from sSgII-70 and bSgII-70, together with water losses, provide a better data fit in both the ovine and bovine 7-8000 analyses to explain the observed MS peak heterogeneity than SgII-70 N-terminal truncation with water losses.

In the ovine analysis, then, within the preferred C-terminal truncation model, the four-waters difference alluded to above between 7878.47 & 7805.98 does not represent the suggested loss of an alanine residue, as these two items are four waters apart (18.015 x 4 = 72.06) on the **68mer** tier (i.e. the 70mer lacking –NI). Meanwhile sense is made of the seven-waters difference by placing 7709.72 on the **67mer** tier (i.e. the 70mer lacking –NNI, with six water losses) and 7583.64 on the **65mer** tier (i.e. the 70mer lacking –KANNI, with two water losses). The eighteenfold water gap of 323.44 between 7484.53 and 7161.09 is explained by a –DIY difference between a **65mer** and a **62mer** and three water losses. The forty-sixfold water difference arises from a comparison of a **68mer** lacking –NI, at 7930.24, and lacking water losses, with a **62mer** at 7102.24, the latter lacking –DIYKANNI and having seven water losses.

Myriad peaks in spectra from the project’s main centre for MALDI-TOF MS (Babraham, using a PE Biosystems Voyager DE PRO System 6022) accord with the preferred ovine C-terminal truncation analysis. Loose fits find support elsewhere. For example, the match to the predicted **65mer** 7493 in the paper’s Table 1 is poor at 7484, but spectra from active in vitro anionex fractions of ovine blood plasma in separate purification runs gave single peaks at 7494 & 7495. The main peak in the 7000s in the paper’s Fig. 2, the c7100 **62mer**, was seen multiple times in fractions of ovine ovarian follicular fluid. (For bovine and porcine equivalents see S3 Figs. 1 & 2.) Many other matches could be cited. The C-terminal truncation analysis brings enlightenment to data from a second MALDI-TOF MS resource (Harwell Laboratory, South Oxfordshire, UK, using a Kratos PC Kompact). There, for example, a 3-30 kDa ultrafiltered upstream precipitate of sheep plasma yielded surprisingly clean spectra with peaks of 7-8000, when the material was in solid form (the paper’s Fig. 4) and in aqueous suspension (personal communication, Carolyn Carr, Oxford University, Oxford, UK; S1, Sheffield Method). Besides the four Fig. 4 ovine peaks analysed in Table 1 there was in the aqueous suspension a **66mer** (7637, next-integer match 7638), a **64mer** (7361, match 7365) and a **63mer** (7287, next-integer match 7288). The 7628 peak in the paper’s Fig. 4 is a loose-fitting **66mer** (match 7621.43). An integer match ion at 7628 had been seen previously however at Babraham in FPLC anionex fractions of ovine plasma (S3 Fig. 3). Accompanying this peak is a second at 6968 (next-integer match 6967).

A third MALDI-TOF MS resource (personal communication, Ed Dudley, University of Swansea, Swansea, UK, using an Applied Biosystems Voyager DE STR) found in ovine ultrafiltered plasma an entity at 7848 (**68mer**) and another adjacent in lower abundance at 7867, which is a water molecule higher. A fourth MALDI-TOF MS resource (personal communication, Julian Hiscox, University of Leeds, Leeds, UK, using a M@LDI L/R System, Waters, Manchester, UK) identified species at *m/z* 7705 (**67mer**), 7505 (**65mer**) & 7184 (**62mer**) from gel eluate of sheep plasma subject to prior removal of albumin, lipid, nucleic acids and salt and prepared on a 10-20% Tris-HCl Criterion gel system (Bio-Rad, Watford, UK). Gel bands were also transferred to PVDF for Edman sequencing but there was insufficient material for identification.

|  | **S3 Table 1. C-terminal losses analysis for Ovine Candidate 7500** | | | | | | |  |  |  |  |
| --- | --- | --- | --- | --- | --- | --- | --- | --- | --- | --- | --- |
|  |  |  |  |  |  |  |  |  |  |  |  |
| **sSgII-70** | **C-terminal** |  | **+1.008** | **-18.015** | **-18.015** | **-18.015** | **-18.015** | **-18.015** | **-18.015** | **-18.015** | **-18.015** |
| **mer** | **residue loss** | **M (Da)** | **MH^+^** | **(H_2_O)** | **(H_2_O)** | **(H_2_O)** | **(H_2_O)** | **(H_2_O)** | **(H_2_O)** | **(H_2_O)** | **(H_2_O)** |
| 70 | - | 8175.01 | 8176.02 | 8158.00 | 8139.99 | 8121.97 | 8103.96 | 8085.94 | 8067.93 | 8049.91 | 8031.90 |
| 69 | -I | 8044.85 | 8045.86 | 8027.84 | 8009.83 | 7991.81 | 7973.80 | 7955.78 | 7937.77 | 7919.75 | 7901.74 |
| 68 | -NI | 7930.74 | 7931.75 | 7913.73 | 7895.72 | 7877.70 | 7859.69 | 7841.67 | 7823.66 | 7805.64 | 7787.63 |
| 67 | -NNI | 7816.64 | 7817.65 | 7799.63 | 7781.62 | 7763.60 | 7745.59 | 7727.57 | 7709.56 | 7691.54 | 7673.53 |
| 66 | -ANNI | 7745.56 | 7746.57 | 7728.55 | 7710.54 | 7692.52 | 7674.51 | 7656.49 | 7638.48 | 7620.46 | 7602.45 |
| 65 | -KANNI | 7617.38 | 7618.39 | 7600.37 | 7582.36 | 7564.34 | 7546.33 | 7528.31 | 7510.30 | 7492.28 | 7474.27 |
| 64 | -YKANNI | 7454.21 | 7455.22 | 7437.20 | 7419.19 | 7401.17 | 7383.16 | 7365.14 | 7347.13 | 7329.11 | 7311.10 |
| 63 | -IYKANNI | 7341.05 | 7342.06 | 7324.04 | 7306.03 | 7288.01 | 7270.00 | 7251.98 | 7233.97 | 7215.95 | 7197.94 |
| 62 | -DIYKANNI | 7225.96 | 7226.97 | 7208.95 | 7190.94 | 7172.92 | 7154.91 | 7136.89 | 7118.88 | 7100.86 | 7082.85 |
| 61 | -DDIYKANNI | 7110.87 | 7111.88 | 7093.86 | 7075.85 | 7057.83 | 7039.82 | 7021.80 | 7003.79 | 6985.77 | 6967.76 |
| 60 | -EDDIYKANNI | 6981.76 | 6982.77 | 6964.75 | 6946.74 | 6928.72 | 6910.71 | 6892.69 | 6874.68 | 6856.66 | 6838.65 |
| 59 |  | 6866.67 | 6867.68 | 6849.66 | 6831.65 | 6813.63 | 6795.62 | 6777.60 | 6759.59 | 6741.57 | 6723.56 |
| 58 |  | 6751.58 | 6752.59 | 6734.57 | 6716.56 | 6698.54 | 6680.53 | 6662.51 | 6644.50 | 6626.48 | 6608.47 |
| 57 |  | 6650.47 | 6651.48 | 6633.46 | 6615.45 | 6597.43 | 6579.42 | 6561.40 | 6543.39 | 6525.37 | 6507.36 |
| 56 |  | 6487.3 | 6488.31 | 6470.29 | 6452.28 | 6434.26 | 6416.25 | 6398.23 | 6380.22 | 6362.20 | 6344.19 |
| 55 |  | 6374.14 | 6375.15 | 6357.13 | 6339.12 | 6321.10 | 6303.09 | 6285.07 | 6267.06 | 6249.04 | 6231.03 |
| 54 |  | 6245.96 | 6246.97 | 6228.95 | 6210.94 | 6192.92 | 6174.91 | 6156.89 | 6138.88 | 6120.86 | 6102.85 |
| 53 |  | 6117.83 | 6118.84 | 6100.82 | 6082.81 | 6064.79 | 6046.78 | 6028.76 | 6010.75 | 5992.73 | 5974.72 |
| 52 |  | 5988.72 | 5989.73 | 5971.71 | 5953.70 | 5935.68 | 5917.67 | 5899.65 | 5881.64 | 5863.62 | 5845.61 |
| 51 |  | 5859.6 | 5860.61 | 5842.59 | 5824.58 | 5806.56 | 5788.55 | 5770.53 | 5752.52 | 5734.50 | 5716.49 |
| 50 |  | 5744.51 | 5745.52 | 5727.50 | 5709.49 | 5691.47 | 5673.46 | 5655.44 | 5637.43 | 5619.41 | 5601.40 |
| 49 |  | 5673.44 | 5674.45 | 5656.43 | 5638.42 | 5620.40 | 5602.39 | 5584.37 | 5566.36 | 5548.34 | 5530.33 |
| 48 |  | 5517.25 | 5518.26 | 5500.24 | 5482.23 | 5464.21 | 5446.20 | 5428.18 | 5410.17 | 5392.15 | 5374.14 |
| 47 |  | 5388.13 | 5389.14 | 5371.12 | 5353.11 | 5335.09 | 5317.08 | 5299.06 | 5281.05 | 5263.03 | 5245.02 |
| 46 |  | 5250.99 | 5252.00 | 5233.98 | 5215.97 | 5197.95 | 5179.94 | 5161.92 | 5143.91 | 5125.89 | 5107.88 |
| 45 |  | 5122.82 | 5123.83 | 5105.81 | 5087.80 | 5069.78 | 5051.77 | 5033.75 | 5015.74 | 4997.72 | 4979.71 |
| 44 |  | 4994.69 | 4995.70 | 4977.68 | 4959.67 | 4941.65 | 4923.64 | 4905.62 | 4887.61 | 4869.59 | 4851.58 |
| 43 |  | 4880.58 | 4881.59 | 4863.57 | 4845.56 | 4827.54 | 4809.53 | 4791.51 | 4773.50 | 4755.48 | 4737.47 |
| 42 |  | 4766.48 | 4767.49 | 4749.47 | 4731.46 | 4713.44 | 4695.43 | 4677.41 | 4659.40 | 4641.38 | 4623.37 |
| 41 |  | 4669.36 | 4670.37 | 4652.35 | 4634.34 | 4616.32 | 4598.31 | 4580.29 | 4562.28 | 4544.26 | 4526.25 |
| 40 |  | 4612.31 | 4613.32 | 4595.30 | 4577.29 | 4559.27 | 4541.26 | 4523.24 | 4505.23 | 4487.21 | 4469.20 |
| 39 |  | 4511.21 | 4512.22 | 4494.20 | 4476.19 | 4458.17 | 4440.16 | 4422.14 | 4404.13 | 4386.11 | 4368.10 |
| 38 |  | 4398.05 | 4399.06 | 4381.04 | 4363.03 | 4345.01 | 4327.00 | 4308.98 | 4290.97 | 4272.95 | 4254.94 |
| 37 |  | 4269.87 | 4270.88 | 4252.86 | 4234.85 | 4216.83 | 4198.82 | 4180.80 | 4162.79 | 4144.77 | 4126.76 |
| 36 |  | 4212.82 | 4213.83 | 4195.81 | 4177.80 | 4159.78 | 4141.77 | 4123.75 | 4105.74 | 4087.72 | 4069.71 |
| 35 |  | 4099.66 | 4100.67 | 4082.65 | 4064.64 | 4046.62 | 4028.61 | 4010.59 | 3992.58 | 3974.56 | 3956.55 |
| 34 |  | 3970.55 | 3971.56 | 3953.54 | 3935.53 | 3917.51 | 3899.50 | 3881.48 | 3863.47 | 3845.45 | 3827.44 |
| 33 |  | 3842.41 | 3843.42 | 3825.40 | 3807.39 | 3789.37 | 3771.36 | 3753.34 | 3735.33 | 3717.31 | 3699.30 |
| 32 |  | 3695.24 | 3696.25 | 3678.23 | 3660.22 | 3642.20 | 3624.19 | 3606.17 | 3588.16 | 3570.14 | 3552.13 |
| 31 |  | 3596.11 | 3597.12 | 3579.10 | 3561.09 | 3543.07 | 3525.06 | 3507.04 | 3489.03 | 3471.01 | 3453.00 |
| 30 |  | 3509.03 | 3510.04 | 3492.02 | 3474.01 | 3455.99 | 3437.98 | 3419.96 | 3401.95 | 3383.93 | 3365.92 |
| 29 |  | 3379.91 | 3380.92 | 3362.90 | 3344.89 | 3326.87 | 3308.86 | 3290.84 | 3272.83 | 3254.81 | 3236.80 |
| 28 |  | 3266.75 | 3267.76 | 3249.74 | 3231.73 | 3213.71 | 3195.70 | 3177.68 | 3159.67 | 3141.65 | 3123.64 |
| 27 |  | 3165.65 | 3166.66 | 3148.64 | 3130.63 | 3112.61 | 3094.60 | 3076.58 | 3058.57 | 3040.55 | 3022.54 |
| 26 |  | 3094.57 | 3095.58 | 3077.56 | 3059.55 | 3041.53 | 3023.52 | 3005.50 | 2987.49 | 2969.47 | 2951.46 |
| 25 |  | 2981.41 | 2982.42 | 2964.40 | 2946.39 | 2928.37 | 2910.36 | 2892.34 | 2874.33 | 2856.31 | 2838.30 |
| 24 |  | 2867.31 | 2868.32 | 2850.30 | 2832.29 | 2814.27 | 2796.26 | 2778.24 | 2760.23 | 2742.21 | 2724.20 |
| 23 |  | 2739.17 | 2740.18 | 2722.16 | 2704.15 | 2686.13 | 2668.12 | 2650.10 | 2632.09 | 2614.07 | 2596.06 |
| 22 |  | 2642.06 | 2643.07 | 2625.05 | 2607.04 | 2589.02 | 2571.01 | 2552.99 | 2534.98 | 2516.96 | 2498.95 |
| 21 |  | 2540.95 | 2541.96 | 2523.94 | 2505.93 | 2487.91 | 2469.90 | 2451.88 | 2433.87 | 2415.85 | 2397.84 |
| 20 |  | 2377.78 | 2378.79 | 2360.77 | 2342.76 | 2324.74 | 2306.73 | 2288.71 | 2270.70 | 2252.68 | 2234.67 |
| 19 |  | 2249.65 | 2250.66 | 2232.64 | 2214.63 | 2196.61 | 2178.60 | 2160.58 | 2142.57 | 2124.55 | 2106.54 |
| 18 |  | 2120.53 | 2121.54 | 2103.52 | 2085.51 | 2067.49 | 2049.48 | 2031.46 | 2013.45 | 1995.43 | 1977.42 |
| 17 |  | 1991.42 | 1992.43 | 1974.41 | 1956.40 | 1938.38 | 1920.37 | 1902.35 | 1884.34 | 1866.32 | 1848.31 |
| 16 |  | 1892.28 | 1893.29 | 1875.27 | 1857.26 | 1839.24 | 1821.23 | 1803.21 | 1785.20 | 1767.18 | 1749.17 |
| 15 |  | 1761.09 | 1762.10 | 1744.08 | 1726.07 | 1708.05 | 1690.04 | 1672.02 | 1654.01 | 1635.99 | 1617.98 |
| 14 |  | 1631.97 | 1632.98 | 1614.96 | 1596.95 | 1578.93 | 1560.92 | 1542.90 | 1524.89 | 1506.87 | 1488.86 |
| 13 |  | 1517.87 | 1518.88 | 1500.86 | 1482.85 | 1464.83 | 1446.82 | 1428.80 | 1410.79 | 1392.77 | 1374.76 |
| 12 |  | 1416.77 | 1417.78 | 1399.76 | 1381.75 | 1363.73 | 1345.72 | 1327.70 | 1309.69 | 1291.67 | 1273.66 |
| 11 |  | 1260.58 | 1261.59 | 1243.57 | 1225.56 | 1207.54 | 1189.53 | 1171.51 | 1153.50 | 1135.48 | 1117.47 |
| 10 |  | 1132.4 | 1133.41 | 1115.39 | 1097.38 | 1079.36 | 1061.35 | 1043.33 | 1025.32 | 1007.30 | 989.29 |
|  |  |  |  |  |  |  |  |  |  |  |  |
|  |  |  |  |  |  |  |  |  |  |  |  |
| **Figure** | **OBSERVED** |  | **CALCULATED** |  | **DIFFERENCE** |  | **DEVIATION** |  | **% ERROR** |  |  |
| 5 | 7930.24 |  | 7931.75 |  | -1.51 |  | 1.510 |  | 0.019 |  |  |
| 3 | 7878.47 |  | 7877.70 |  | 0.77 |  | 0.770 |  | 0.010 |  |  |
| 3 | 7805.98 |  | 7805.64 |  | 0.34 |  | 0.340 |  | 0.004 |  |  |
| 3 | 7709.72 |  | 7709.56 |  | 0.16 |  | 0.160 |  | 0.002 |  |  |
| 4 | 7709.42 |  | 7709.56 |  | -0.14 |  | 0.140 |  | 0.002 |  |  |
| 4 | 7628.30 |  | 7620.46 |  | 7.84 |  | 7.840 |  | 0.103 |  |  |
| 5 | 7583.64 |  | 7582.36 |  | 1.28 |  | 1.280 |  | 0.017 |  |  |
| 5 | 7565.93 |  | 7564.34 |  | 1.59 |  | 1.590 |  | 0.021 |  |  |
| 4 | 7561.42 |  | 7564.34 |  | -2.92 |  | 2.920 |  | 0.039 |  |  |
| 2 | 7484.53 |  | 7492.28 |  | -7.75 |  | 7.750 |  | 0.103 |  |  |
| 4 | 7161.09 |  | 7154.91 |  | 6.18 |  | 6.180 |  | 0.086 |  |  |
| 2 | 7102.24 |  | 7100.86 |  | 1.38 |  | 1.380 |  | 0.019 |  |  |
| **TOTAL** | 91120.98 | **TOTAL** | 91113.76 | **TOTAL** | 7.22 | **TOTAL** | 31.86 | **TOTAL** | 0.426 |  |  |
| **MEAN** | 7593.42 | **MEAN** | 7592.81 | **MEAN** | 0.60 | **MEAN** | 2.655 | **MEAN** | 0.035 |  |  |
| **Observed vs Calculated as a percentage** | | | 100.008 |  |  |  |  |  |  |  |  |

|  | **S3 Table 2. N-terminal losses analysis for Ovine Candidate 7500** | | | | | | |  |  |  |  |
| --- | --- | --- | --- | --- | --- | --- | --- | --- | --- | --- | --- |
|  |  |  |  |  |  |  |  |  |  |  |  |
| **sSgII-70** | **N-terminal** |  | **+1.008** | **-18.015** | **-18.015** | **-18.015** | **-18.015** | **-18.015** | **-18.015** | **-18.015** | **-18.015** |
| **mer** | **residue loss** | **M (Da)** | **MH^+^** | **(H_2_O)** | **(H_2_O)** | **(H_2_O)** | **(H_2_O)** | **(H_2_O)** | **(H_2_O)** | **(H_2_O)** | **(H_2_O)** |
| 70 | - | 8175.01 | 8176.02 | 8158.00 | 8139.99 | 8121.97 | 8103.96 | 8085.94 | 8067.93 | 8049.91 | 8031.90 |
| 69 | M- | 8042.81 | 8043.82 | 8025.80 | 8007.79 | 7989.77 | 7971.76 | 7953.74 | 7935.73 | 7917.71 | 7899.70 |
| 68 | ML- | 7929.65 | 7930.66 | 7912.64 | 7894.63 | 7876.61 | 7858.60 | 7840.58 | 7822.57 | 7804.55 | 7786.54 |
| 67 | MLK- | 7801.48 | 7802.49 | 7784.47 | 7766.46 | 7748.44 | 7730.43 | 7712.41 | 7694.40 | 7676.38 | 7658.37 |
| 66 | MLKT- | 7700.37 | 7701.38 | 7683.36 | 7665.35 | 7647.33 | 7629.32 | 7611.30 | 7593.29 | 7575.27 | 7557.26 |
| 65 | MLKTG- | 7643.32 | 7644.33 | 7626.31 | 7608.30 | 7590.28 | 7572.27 | 7554.25 | 7536.24 | 7518.22 | 7500.21 |
| 64 | MLKTGE- | 7514.21 | 7515.22 | 7497.20 | 7479.19 | 7461.17 | 7443.16 | 7425.14 | 7407.13 | 7389.11 | 7371.10 |
| 63 | MLKTGEK- | 7386.03 | 7387.04 | 7369.02 | 7351.01 | 7332.99 | 7314.98 | 7296.96 | 7278.95 | 7260.93 | 7242.92 |
| 62 | MLKTGEKP- | 7288.92 | 7289.93 | 7271.91 | 7253.90 | 7235.88 | 7217.87 | 7199.85 | 7181.84 | 7163.82 | 7145.81 |
| 61 | MLKTGEKPV- | 7189.78 | 7190.79 | 7172.77 | 7154.76 | 7136.74 | 7118.73 | 7100.71 | 7082.70 | 7064.68 | 7046.67 |
| 60 | MLKTGEKPVF- | 7042.61 | 7043.62 | 7025.60 | 7007.59 | 6989.57 | 6971.56 | 6953.54 | 6935.53 | 6917.51 | 6899.50 |
|  |  |  |  |  |  |  |  |  |  |  |  |
|  |  |  |  |  |  |  |  |  |  |  |  |
| **Figure** | **OBSERVED** |  | **CALCULATED** |  | **DIFFERENCE** |  | **DEVIATION** |  | **% ERROR** |  |  |
| 5 | 7930.24 |  | 7930.66 |  | -0.42 |  | 0.420 |  | 0.005 |  |  |
| 3 | 7878.47 |  | 7876.61 |  | 1.86 |  | 1.860 |  | 0.024 |  |  |
| 3 | 7805.98 |  | 7804.55 |  | 1.43 |  | 1.430 |  | 0.018 |  |  |
| 3 | 7709.72 |  | 7712.41 |  | -2.69 |  | 2.690 |  | 0.035 |  |  |
| 4 | 7709.42 |  | 7712.41 |  | -2.99 |  | 2.990 |  | 0.039 |  |  |
| 4 | 7628.30 |  | 7629.32 |  | -1.02 |  | 1.020 |  | 0.013 |  |  |
| 5 | 7583.64 |  | 7590.28 |  | -6.64 |  | 6.640 |  | 0.087 |  |  |
| 5 | 7565.93 |  | 7572.27 |  | -6.34 |  | 6.340 |  | 0.084 |  |  |
| 4 | 7561.42 |  | 7557.26 |  | 4.16 |  | 4.160 |  | 0.055 |  |  |
| 2 | 7484.53 |  | 7479.19 |  | 5.34 |  | 5.340 |  | 0.071 |  |  |
| 4 | 7161.09 |  | 7163.82 |  | -2.73 |  | 2.730 |  | 0.038 |  |  |
| 2 | 7102.24 |  | 7100.71 |  | 1.53 |  | 1.530 |  | 0.022 |  |  |
| **TOTAL** | 91120.98 | **TOTAL** | 91129.49 | **TOTAL** | -8.51 | **TOTAL** | 37.15 | **TOTAL** | 0.492 |  |  |
| **MEAN** | 7593.42 | **MEAN** | 7594.12 | **MEAN** | -0.71 | **MEAN** | 3.096 | **MEAN** | 0.041 |  |  |
| **Observed vs Calculated as a percentage** | | | 99.991 |  |  |  |  |  |  |  |  |

|  | **S3 Table 3. C-terminal losses analysis for Bovine Candidate 7500** | | | | | | |  |  |  |  |
| --- | --- | --- | --- | --- | --- | --- | --- | --- | --- | --- | --- |
|  |  |  |  |  |  |  |  |  |  |  |  |
| **bSgII-70** | **C-terminal** |  | **+1.008** | **-18.015** | **-18.015** | **-18.015** | **-18.015** | **-18.015** | **-18.015** | **-18.015** | **-18.015** |
| **mer** | **residue loss** | **M (Da)** | **MH^+^** | **(H_2_O)** | **(H_2_O)** | **(H_2_O)** | **(H_2_O)** | **(H_2_O)** | **(H_2_O)** | **(H_2_O)** | **(H_2_O)** |
| 70 | - | 8149.00 | 8150.01 | 8131.99 | 8113.98 | 8095.96 | 8077.95 | 8059.93 | 8041.92 | 8023.90 | 8005.89 |
| 69 | -I | 8018.83 | 8019.84 | 8001.82 | 7983.81 | 7965.79 | 7947.78 | 7929.76 | 7911.75 | 7893.73 | 7875.72 |
| 68 | -NI | 7904.73 | 7905.74 | 7887.72 | 7869.71 | 7851.69 | 7833.68 | 7815.66 | 7797.65 | 7779.63 | 7761.62 |
| 67 | -NNI | 7790.63 | 7791.64 | 7773.62 | 7755.61 | 7737.59 | 7719.58 | 7701.56 | 7683.55 | 7665.53 | 7647.52 |
| 66 | -ANNI | 7719.55 | 7720.56 | 7702.54 | 7684.53 | 7666.51 | 7648.50 | 7630.48 | 7612.47 | 7594.45 | 7576.44 |
| 65 | -KANNI | 7591.37 | 7592.38 | 7574.36 | 7556.35 | 7538.33 | 7520.32 | 7502.30 | 7484.29 | 7466.27 | 7448.26 |
| 64 | -YKANNI | 7428.20 | 7429.21 | 7411.19 | 7393.18 | 7375.16 | 7357.15 | 7339.13 | 7321.12 | 7303.10 | 7285.09 |
| 63 | -IYKANNI | 7315.04 | 7316.05 | 7298.03 | 7280.02 | 7262.00 | 7243.99 | 7225.97 | 7207.96 | 7189.94 | 7171.93 |
| 62 | -DIYKANNI | 7199.95 | 7200.96 | 7182.94 | 7164.93 | 7146.91 | 7128.90 | 7110.88 | 7092.87 | 7074.85 | 7056.84 |
| 61 | -DDIYKANNI | 7084.86 | 7085.87 | 7067.85 | 7049.84 | 7031.82 | 7013.81 | 6995.79 | 6977.78 | 6959.76 | 6941.75 |
| 60 | -EDDIYKANNI | 6955.74 | 6956.75 | 6938.73 | 6920.72 | 6902.70 | 6884.69 | 6866.67 | 6848.66 | 6830.64 | 6812.63 |
| 59 |  | 6840.66 | 6841.67 | 6823.65 | 6805.64 | 6787.62 | 6769.61 | 6751.59 | 6733.58 | 6715.56 | 6697.55 |
| 58 |  | 6725.57 | 6726.58 | 6708.56 | 6690.55 | 6672.53 | 6654.52 | 6636.50 | 6618.49 | 6600.47 | 6582.46 |
| 57 |  | 6624.46 | 6625.47 | 6607.45 | 6589.44 | 6571.42 | 6553.41 | 6535.39 | 6517.38 | 6499.36 | 6481.35 |
| 56 |  | 6461.29 | 6462.30 | 6444.28 | 6426.27 | 6408.25 | 6390.24 | 6372.22 | 6354.21 | 6336.19 | 6318.18 |
| 55 |  | 6348.13 | 6349.14 | 6331.12 | 6313.11 | 6295.09 | 6277.08 | 6259.06 | 6241.05 | 6223.03 | 6205.02 |
| 54 |  | 6219.95 | 6220.96 | 6202.94 | 6184.93 | 6166.91 | 6148.90 | 6130.88 | 6112.87 | 6094.85 | 6076.84 |
| 53 |  | 6091.82 | 6092.83 | 6074.81 | 6056.80 | 6038.78 | 6020.77 | 6002.75 | 5984.74 | 5966.72 | 5948.71 |
| 52 |  | 5962.71 | 5963.72 | 5945.70 | 5927.69 | 5909.67 | 5891.66 | 5873.64 | 5855.63 | 5837.61 | 5819.60 |
| 51 |  | 5833.59 | 5834.60 | 5816.58 | 5798.57 | 5780.55 | 5762.54 | 5744.52 | 5726.51 | 5708.49 | 5690.48 |
| 50 |  | 5718.50 | 5719.51 | 5701.49 | 5683.48 | 5665.46 | 5647.45 | 5629.43 | 5611.42 | 5593.40 | 5575.39 |
| 49 |  | 5647.42 | 5648.43 | 5630.41 | 5612.40 | 5594.38 | 5576.37 | 5558.35 | 5540.34 | 5522.32 | 5504.31 |
| 48 |  | 5491.24 | 5492.25 | 5474.23 | 5456.22 | 5438.20 | 5420.19 | 5402.17 | 5384.16 | 5366.14 | 5348.13 |
| 47 |  | 5362.12 | 5363.13 | 5345.11 | 5327.10 | 5309.08 | 5291.07 | 5273.05 | 5255.04 | 5237.02 | 5219.01 |
| 46 |  | 5205.93 | 5206.94 | 5188.92 | 5170.91 | 5152.89 | 5134.88 | 5116.86 | 5098.85 | 5080.83 | 5062.82 |
| 45 |  | 5077.76 | 5078.77 | 5060.75 | 5042.74 | 5024.72 | 5006.71 | 4988.69 | 4970.68 | 4952.66 | 4934.65 |
| 44 |  | 4949.63 | 4950.64 | 4932.62 | 4914.61 | 4896.59 | 4878.58 | 4860.56 | 4842.55 | 4824.53 | 4806.52 |
| 43 |  | 4862.55 | 4863.56 | 4845.54 | 4827.53 | 4809.51 | 4791.50 | 4773.48 | 4755.47 | 4737.45 | 4719.44 |
| 42 |  | 4748.45 | 4749.46 | 4731.44 | 4713.43 | 4695.41 | 4677.40 | 4659.38 | 4641.37 | 4623.35 | 4605.34 |
| 41 |  | 4651.33 | 4652.34 | 4634.32 | 4616.31 | 4598.29 | 4580.28 | 4562.26 | 4544.25 | 4526.23 | 4508.22 |
| 40 |  | 4594.28 | 4595.29 | 4577.27 | 4559.26 | 4541.24 | 4523.23 | 4505.21 | 4487.20 | 4469.18 | 4451.17 |
| 39 |  | 4493.17 | 4494.18 | 4476.16 | 4458.15 | 4440.13 | 4422.12 | 4404.10 | 4386.09 | 4368.07 | 4350.06 |
| 38 |  | 4380.01 | 4381.02 | 4363.00 | 4344.99 | 4326.97 | 4308.96 | 4290.94 | 4272.93 | 4254.91 | 4236.90 |
| 37 |  | 4251.84 | 4252.85 | 4234.83 | 4216.82 | 4198.80 | 4180.79 | 4162.77 | 4144.76 | 4126.74 | 4108.73 |
| 36 |  | 4194.79 | 4195.80 | 4177.78 | 4159.77 | 4141.75 | 4123.74 | 4105.72 | 4087.71 | 4069.69 | 4051.68 |
| 35 |  | 4081.63 | 4082.64 | 4064.62 | 4046.61 | 4028.59 | 4010.58 | 3992.56 | 3974.55 | 3956.53 | 3938.52 |
| 34 |  | 3952.51 | 3953.52 | 3935.50 | 3917.49 | 3899.47 | 3881.46 | 3863.44 | 3845.43 | 3827.41 | 3809.40 |
| 33 |  | 3824.38 | 3825.39 | 3807.37 | 3789.36 | 3771.34 | 3753.33 | 3735.31 | 3717.30 | 3699.28 | 3681.27 |
| 32 |  | 3677.20 | 3678.21 | 3660.19 | 3642.18 | 3624.16 | 3606.15 | 3588.13 | 3570.12 | 3552.10 | 3534.09 |
| 31 |  | 3578.07 | 3579.08 | 3561.06 | 3543.05 | 3525.03 | 3507.02 | 3489.00 | 3470.99 | 3452.97 | 3434.96 |
| 30 |  | 3490.99 | 3492.00 | 3473.98 | 3455.97 | 3437.95 | 3419.94 | 3401.92 | 3383.91 | 3365.89 | 3347.88 |
| 29 |  | 3361.88 | 3362.89 | 3344.87 | 3326.86 | 3308.84 | 3290.83 | 3272.81 | 3254.80 | 3236.78 | 3218.77 |
| 28 |  | 3248.72 | 3249.73 | 3231.71 | 3213.70 | 3195.68 | 3177.67 | 3159.65 | 3141.64 | 3123.62 | 3105.61 |
| 27 |  | 3147.61 | 3148.62 | 3130.60 | 3112.59 | 3094.57 | 3076.56 | 3058.54 | 3040.53 | 3022.51 | 3004.50 |
| 26 |  | 3076.54 | 3077.55 | 3059.53 | 3041.52 | 3023.50 | 3005.49 | 2987.47 | 2969.46 | 2951.44 | 2933.43 |
| 25 |  | 2963.38 | 2964.39 | 2946.37 | 2928.36 | 2910.34 | 2892.33 | 2874.31 | 2856.30 | 2838.28 | 2820.27 |
| 24 |  | 2849.27 | 2850.28 | 2832.26 | 2814.25 | 2796.23 | 2778.22 | 2760.20 | 2742.19 | 2724.17 | 2706.16 |
| 23 |  | 2721.14 | 2722.15 | 2704.13 | 2686.12 | 2668.10 | 2650.09 | 2632.07 | 2614.06 | 2596.04 | 2578.03 |
| 22 |  | 2624.02 | 2625.03 | 2607.01 | 2589.00 | 2570.98 | 2552.97 | 2534.95 | 2516.94 | 2498.92 | 2480.91 |
| 21 |  | 2522.92 | 2523.93 | 2505.91 | 2487.90 | 2469.88 | 2451.87 | 2433.85 | 2415.84 | 2397.82 | 2379.81 |
| 20 |  | 2359.74 | 2360.75 | 2342.73 | 2324.72 | 2306.70 | 2288.69 | 2270.67 | 2252.66 | 2234.64 | 2216.63 |
| 19 |  | 2231.61 | 2232.62 | 2214.60 | 2196.59 | 2178.57 | 2160.56 | 2142.54 | 2124.53 | 2106.51 | 2088.50 |
| 18 |  | 2102.50 | 2103.51 | 2085.49 | 2067.48 | 2049.46 | 2031.45 | 2013.43 | 1995.42 | 1977.40 | 1959.39 |
| 17 |  | 1973.38 | 1974.39 | 1956.37 | 1938.36 | 1920.34 | 1902.33 | 1884.31 | 1866.30 | 1848.28 | 1830.27 |
| 16 |  | 1874.25 | 1875.26 | 1857.24 | 1839.23 | 1821.21 | 1803.20 | 1785.18 | 1767.17 | 1749.15 | 1731.14 |
| 15 |  | 1761.09 | 1762.10 | 1744.08 | 1726.07 | 1708.05 | 1690.04 | 1672.02 | 1654.01 | 1635.99 | 1617.98 |
| 14 |  | 1631.97 | 1632.98 | 1614.96 | 1596.95 | 1578.93 | 1560.92 | 1542.90 | 1524.89 | 1506.87 | 1488.86 |
| 13 |  | 1517.87 | 1518.88 | 1500.86 | 1482.85 | 1464.83 | 1446.82 | 1428.80 | 1410.79 | 1392.77 | 1374.76 |
| 12 |  | 1416.77 | 1417.78 | 1399.76 | 1381.75 | 1363.73 | 1345.72 | 1327.70 | 1309.69 | 1291.67 | 1273.66 |
| 11 |  | 1260.58 | 1261.59 | 1243.57 | 1225.56 | 1207.54 | 1189.53 | 1171.51 | 1153.50 | 1135.48 | 1117.47 |
| 10 |  | 1132.40 | 1133.41 | 1115.39 | 1097.38 | 1079.36 | 1061.35 | 1043.33 | 1025.32 | 1007.30 | 989.29 |
|  |  |  |  |  |  |  |  |  |  |  |  |
| **Figure** | **OBSERVED** |  | **CALCULATED** |  | **DIFFERENCE** |  | **DEVIATION** |  | **% ERROR** |  |  |
| **S3 Fig. 1** | 7893.30 |  | 7893.73 |  | -0.43 |  | 0.430 |  | 0.005 |  |  |
| **4** | 7643.93 |  | 7647.52 |  | -3.59 |  | 3.590 |  | 0.047 |  |  |
| **4** | 7573.19 |  | 7574.36 |  | -1.17 |  | 1.170 |  | 0.015 |  |  |
| **4** | 7561.42 |  | 7556.35 |  | 5.07 |  | 5.070 |  | 0.067 |  |  |
| **6** | 7687.50 |  | 7684.53 |  | 2.97 |  | 2.970 |  | 0.039 |  |  |
| **6** | 7609.94 |  | 7612.47 |  | -2.53 |  | 2.530 |  | 0.033 |  |  |
| **6** | 7500.72 |  | 7502.30 |  | -1.58 |  | 1.580 |  | 0.021 |  |  |
| **6** | 7482.74 |  | 7484.29 |  | -1.55 |  | 1.550 |  | 0.021 |  |  |
| **6** | 7370.96 |  | 7375.16 |  | -4.20 |  | 4.200 |  | 0.057 |  |  |
| **6** | 7352.91 |  | 7357.15 |  | -4.24 |  | 4.240 |  | 0.058 |  |  |
| **6** | 7222.74 |  | 7225.97 |  | -3.23 |  | 3.230 |  | 0.045 |  |  |
| **6** | 7104.01 |  | 7110.88 |  | -6.87 |  | 6.870 |  | 0.097 |  |  |
| **S3 Fig. 1** | 7094.24 |  | 7092.87 |  | 1.37 |  | 1.370 |  | 0.019 |  |  |
| **TOTAL** | 97097.60 | **TOTAL** | 97117.58 | **TOTAL** | -19.98 | **TOTAL** | 38.800 | **TOTAL** | 0.524 |  |  |
| **MEAN** | 7469.05 | **MEAN** | 7470.58 | **MEAN** | -1.54 | **MEAN** | 2.985 | **MEAN** | 0.040 |  |  |
| **Observed vs Calculated as a percentage** | | | 99.979 |  |  |  |  |  |  |  |  |

|  | **S3 Table 4. N-terminal losses analysis for Bovine Candidate 7500** | | | | | | |  |  |  |  |
| --- | --- | --- | --- | --- | --- | --- | --- | --- | --- | --- | --- |
|  |  |  |  |  |  |  |  |  |  |  |  |
| **bSgII-70** | **N-terminal** |  | **+1.008** | **-18.015** | **-18.015** | **-18.015** | **-18.015** | **-18.015** | **-18.015** | **-18.015** | **-18.015** |
| **mer** | **residue loss** | **M (Da)** | **MH^+^** | **(H_2_O)** | **(H_2_O)** | **(H_2_O)** | **(H_2_O)** | **(H_2_O)** | **(H_2_O)** | **(H_2_O)** | **(H_2_O)** |
| 70 | - | 8149.00 | 8150.01 | 8131.99 | 8113.98 | 8095.96 | 8077.95 | 8059.93 | 8041.92 | 8023.90 | 8005.89 |
| 69 | M- | 8016.80 | 8017.81 | 7999.79 | 7981.78 | 7963.76 | 7945.75 | 7927.73 | 7909.72 | 7891.70 | 7873.69 |
| 68 | ML- | 7903.64 | 7904.65 | 7886.63 | 7868.62 | 7850.60 | 7832.59 | 7814.57 | 7796.56 | 7778.54 | 7760.53 |
| 67 | MLK- | 7775.47 | 7776.48 | 7758.46 | 7740.45 | 7722.43 | 7704.42 | 7686.40 | 7668.39 | 7650.37 | 7632.36 |
| 66 | MLKT- | 7674.36 | 7675.37 | 7657.35 | 7639.34 | 7621.32 | 7603.31 | 7585.29 | 7567.28 | 7549.26 | 7531.25 |
| 65 | MLKTG- | 7617.31 | 7618.32 | 7600.30 | 7582.29 | 7564.27 | 7546.26 | 7528.24 | 7510.23 | 7492.21 | 7474.20 |
| 64 | MLKTGE- | 7488.19 | 7489.20 | 7471.18 | 7453.17 | 7435.15 | 7417.14 | 7399.12 | 7381.11 | 7363.09 | 7345.08 |
| 63 | MLKTGEK- | 7360.02 | 7361.03 | 7343.01 | 7325.00 | 7306.98 | 7288.97 | 7270.95 | 7252.94 | 7234.92 | 7216.91 |
| 62 | MLKTGEKP- | 7262.90 | 7263.91 | 7245.89 | 7227.88 | 7209.86 | 7191.85 | 7173.83 | 7155.82 | 7137.80 | 7119.79 |
| 61 | MLKTGEKPV- | 7163.77 | 7164.78 | 7146.76 | 7128.75 | 7110.73 | 7092.72 | 7074.70 | 7056.69 | 7038.67 | 7020.66 |
| 60 | MLKTGEKPVF- | 7016.59 | 7017.60 | 6999.58 | 6981.57 | 6963.55 | 6945.54 | 6927.52 | 6909.51 | 6891.49 | 6873.48 |
|  |  |  |  |  |  |  |  |  |  |  |  |
|  |  |  |  |  |  |  |  |  |  |  |  |
| **Figure** | **OBSERVED** |  | **CALCULATED** |  | **DIFFERENCE** |  | **DEVIATION** |  | **% ERROR** |  |  |
| **S3 Fig. 1** | 7893.30 |  | 7891.70 |  | 1.60 |  | 1.600 |  | 0.020 |  |  |
| **4** | 7643.93 |  | 7639.34 |  | 4.59 |  | 4.590 |  | 0.060 |  |  |
| **4** | 7573.19 |  | 7567.28 |  | 5.91 |  | 5.910 |  | 0.078 |  |  |
| **6** | 7561.42 |  | 7564.27 |  | -2.85 |  | 2.850 |  | 0.038 |  |  |
| **6** | 7687.50 |  | 7686.40 |  | 1.10 |  | 1.100 |  | 0.014 |  |  |
| **6** | 7609.94 |  | 7603.31 |  | 6.63 |  | 6.630 |  | 0.087 |  |  |
| **6** | 7500.72 |  | 7492.21 |  | 8.51 |  | 8.510 |  | 0.114 |  |  |
| **6** | 7482.74 |  | 7489.20 |  | -6.46 |  | 6.460 |  | 0.086 |  |  |
| **6** | 7370.96 |  | 7363.09 |  | 7.87 |  | 7.870 |  | 0.107 |  |  |
| **6** | 7352.91 |  | 7345.08 |  | 7.83 |  | 7.830 |  | 0.107 |  |  |
| **6** | 7222.74 |  | 7227.88 |  | -5.14 |  | 5.140 |  | 0.071 |  |  |
| **6** | 7104.01 |  | 7110.73 |  | -6.72 |  | 6.720 |  | 0.095 |  |  |
| **S3 Fig. 1** | 7094.24 |  | 7092.72 |  | 1.52 |  | 1.520 |  | 0.021 |  |  |
| **TOTAL** | 97097.60 | **TOTAL** | 97073.21 | **TOTAL** | 24.39 | **TOTAL** | 66.730 | **TOTAL** | 0.898 |  |  |
| **MEAN** | 7469.05 | **MEAN** | 7467.17 | **MEAN** | 1.88 | **MEAN** | 5.133 | **MEAN** | 0.069 |  |  |
| **Observed vs Calculated as a percentage** | | | 100.025 |  |  |  |  |  |  |  |  |

| **Chi-squared tests** | |  |  |  |
| --- | --- | --- | --- | --- |
| **O = Observed** | | **E = Expected (i.e. match)** | | |
|  |  |  |  |  |
|  |  |  |  |  |
| **S3 Table 5: Ovine Candidate 7500 C-terminal losses chi-squared test** | | | | |
|  |  |  |  |  |
| **OBSERVED** | **Expected** | **O - E** | **(O - E)^2^** | **(O - E)^2^ /E** |
| 7930.24 | 7931.75 | -1.51 | 2.2801 | 0.0003 |
| 7878.47 | 7877.70 | 0.77 | 0.5929 | 0.0001 |
| 7805.98 | 7805.64 | 0.34 | 0.1156 | 0.0000 |
| 7709.72 | 7709.56 | 0.16 | 0.0256 | 0.0000 |
| 7709.42 | 7709.56 | -0.14 | 0.0196 | 0.0000 |
| 7628.30 | 7620.46 | 7.84 | 61.4656 | 0.0081 |
| 7583.64 | 7582.36 | 1.28 | 1.6384 | 0.0002 |
| 7565.93 | 7564.34 | 1.59 | 2.5281 | 0.0003 |
| 7561.42 | 7564.34 | -2.92 | 8.5264 | 0.0011 |
| 7484.53 | 7492.28 | -7.75 | 60.0625 | 0.0080 |
| 7161.09 | 7154.91 | 6.18 | 38.1924 | 0.0053 |
| 7102.24 | 7100.86 | 1.38 | 1.9044 | 0.0003 |
|  |  |  |  |  |
|  |  |  | **Chi-squared P =** | 0.0237 |
|  |  |  |  |  |

|  |  |  |  |  |
| --- | --- | --- | --- | --- |
| **S3 Table 6: Ovine Candidate 7500 N-terminal losses chi-squared test** | | | | |
|  |  |  |  |  |
| **OBSERVED** | **Expected** | **O - E** | **(O - E)^2^** | **(O - E)^2^ /E** |
| 7930.24 | 7930.66 | -0.42 | 0.1764 | 0.0000 |
| 7878.47 | 7876.61 | 1.86 | 3.4596 | 0.0004 |
| 7805.98 | 7804.55 | 1.43 | 2.0449 | 0.0003 |
| 7709.72 | 7712.41 | -2.69 | 7.2361 | 0.0009 |
| 7709.42 | 7712.41 | -2.99 | 8.9401 | 0.0012 |
| 7628.30 | 7629.32 | -1.02 | 1.0404 | 0.0001 |
| 7583.64 | 7590.28 | -6.64 | 44.0896 | 0.0058 |
| 7565.93 | 7572.27 | -6.34 | 40.1956 | 0.0053 |
| 7561.42 | 7557.26 | 4.16 | 17.3056 | 0.0023 |
| 7484.53 | 7479.19 | 5.34 | 28.5156 | 0.0038 |
| 7161.09 | 7163.82 | -2.73 | 7.4529 | 0.0010 |
| 7102.24 | 7100.71 | 1.53 | 2.3409 | 0.0003 |
|  |  |  |  |  |
|  |  |  | **Chi-squared P =** | 0.0215 |
|  |  |  |  |  |

|  |  |  |  |  |
| --- | --- | --- | --- | --- |
| **S3 Table 7: Bovine Candidate 7500 C-terminal losses chi-squared test** | | | | |
|  |  |  |  |  |
| **OBSERVED** | **Expected** | **O - E** | **(O - E)^2^** | **(O - E)^2^ /E** |
| 7893.30 | 7893.73 | -0.43 | 0.1849 | 0.0000 |
| 7643.93 | 7647.52 | -3.59 | 12.8881 | 0.0017 |
| 7573.19 | 7574.36 | -1.17 | 1.3689 | 0.0002 |
| 7561.42 | 7556.35 | 5.07 | 25.7049 | 0.0034 |
| 7687.50 | 7684.53 | 2.97 | 8.8209 | 0.0011 |
| 7609.94 | 7612.47 | -2.53 | 6.4009 | 0.0008 |
| 7500.72 | 7502.30 | -1.58 | 2.4964 | 0.0003 |
| 7482.74 | 7484.29 | -1.55 | 2.4025 | 0.0003 |
| 7370.96 | 7375.16 | -4.20 | 17.6400 | 0.0024 |
| 7352.91 | 7357.15 | -4.24 | 17.9776 | 0.0024 |
| 7222.74 | 7225.97 | -3.23 | 10.4329 | 0.0014 |
| 7104.01 | 7110.88 | -6.87 | 47.1969 | 0.0066 |
| 7094.24 | 7092.87 | 1.37 | 1.8769 | 0.0003 |
|  |  |  |  |  |
|  |  |  | **Chi-squared P =** | 0.0211 |
|  |  |  |  |  |

|  |  |  |  |  |
| --- | --- | --- | --- | --- |
| **S3 Table 8: Bovine Candidate 7500 N-terminal losses chi-squared test** | | | | |
|  |  |  |  |  |
| **OBSERVED** | **Expected** | **O - E** | **(O - E)^2^** | **(O - E)^2^ /E** |
| 7893.30 | 7891.70 | 1.60 | 2.5600 | 0.0003 |
| 7643.93 | 7639.34 | 4.59 | 21.0681 | 0.0028 |
| 7573.19 | 7567.28 | 5.91 | 34.9281 | 0.0046 |
| 7561.42 | 7564.27 | -2.85 | 8.1225 | 0.0011 |
| 7687.50 | 7686.40 | 1.10 | 1.2100 | 0.0002 |
| 7609.94 | 7603.31 | 6.63 | 43.9569 | 0.0058 |
| 7500.72 | 7492.21 | 8.51 | 72.4201 | 0.0097 |
| 7482.74 | 7489.20 | -6.46 | 41.7316 | 0.0056 |
| 7370.96 | 7363.09 | 7.87 | 61.9369 | 0.0084 |
| 7352.91 | 7345.08 | 7.83 | 61.3089 | 0.0083 |
| 7222.74 | 7227.88 | -5.14 | 26.4196 | 0.0037 |
| 7104.01 | 7110.73 | -6.72 | 45.1584 | 0.0064 |
| 7094.24 | 7092.72 | 1.52 | 2.3104 | 0.0003 |
|  |  |  |  |  |
|  |  |  | **Chi-squared P =** | 0.0570 |
|  |  |  |  |  |

|  |  |  |  |  |
| --- | --- | --- | --- | --- |
| **S3 Table 9: Ovine Minor Peaks chi-squared test** | | | |  |
|  |  |  |  |  |
| **OBSERVED** | **Expected** | **O - E** | **(O - E)^2^** | **(O - E)^2^ /E** |
| 6109.35 | 6102.85 | 6.50 | 42.2500 | 0.0069 |
| 6070.58 | 6064.79 | 5.79 | 33.5241 | 0.0055 |
| 5655.12 | 5655.44 | -0.32 | 0.1024 | 0.0000 |
| 5070.90 | 5069.78 | 1.12 | 1.2544 | 0.0002 |
| 4785.16 | 4791.51 | -6.35 | 40.3225 | 0.0084 |
| 4755.85 | 4755.48 | 0.37 | 0.1369 | 0.0000 |
| 4748.66 | 4749.47 | -0.81 | 0.6561 | 0.0001 |
|  |  |  |  |  |
|  |  |  | **Chi-squared P =** | 0.0213 |

The Ovine Minor Peaks are described in the paper.

∑Observed (n = 7) = 37195.62

∑Matches = 37189.32

∑Observed/∑Matches x100 = 37195.62/37189.32 x100 = **100.02%**


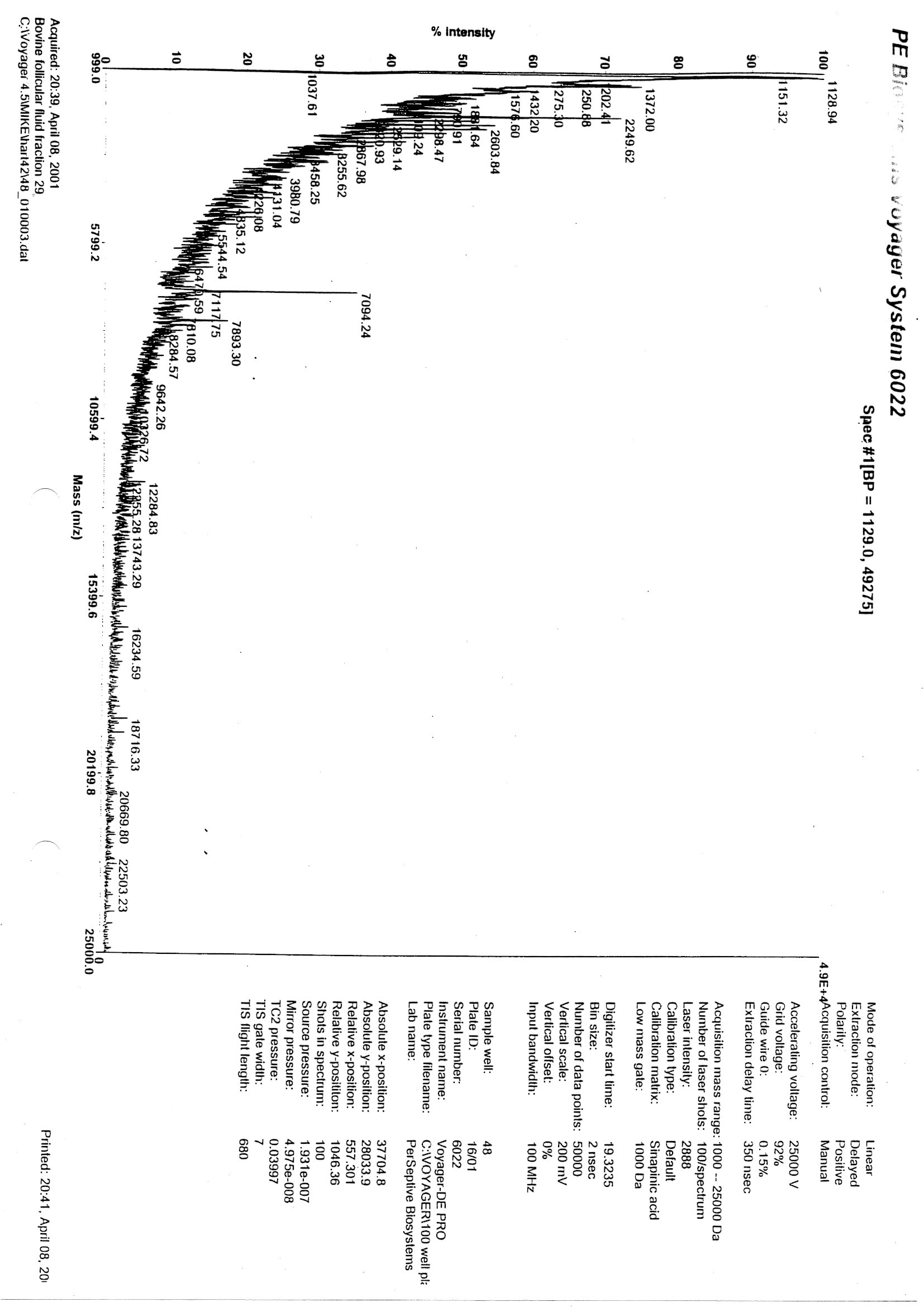


**S3 Figure 1.** Bovine ovarian follicular fluid anionex Fraction 29, obtained by the Babraham method of purification (S1). The peaks at *m/z* 7094 and 7893 have been used in the bovine core analysis (S3 Tables 3 & 4). The characteristic lefthand slopes in MALDI spectra are interpreted in the paper as SgII-70 fragment ramps. Within this interpretation, matches to prominent peaks in the ramp are as follows:

1129 base peak (**10mer** S3 Table 3 match 1133)

1151 (**11mer** match 1153)

1372 (**12mer** match 1363)

2249 (**20mer** match 2252)

2603 (**22mer** match 2607)

∑Observed (n = 5) = 8503

∑Matches = 8508

∑Observed/∑Matches x100 = 8503/8508 x100 = **99.94%**

Further MALDI mass spectra of anionex fractions of bovine ovarian follicular fluid are shown in S2 in connection with an angiogenesis study, where ∑Observed (n = 10)/∑Matches x100 = **99.95%**.


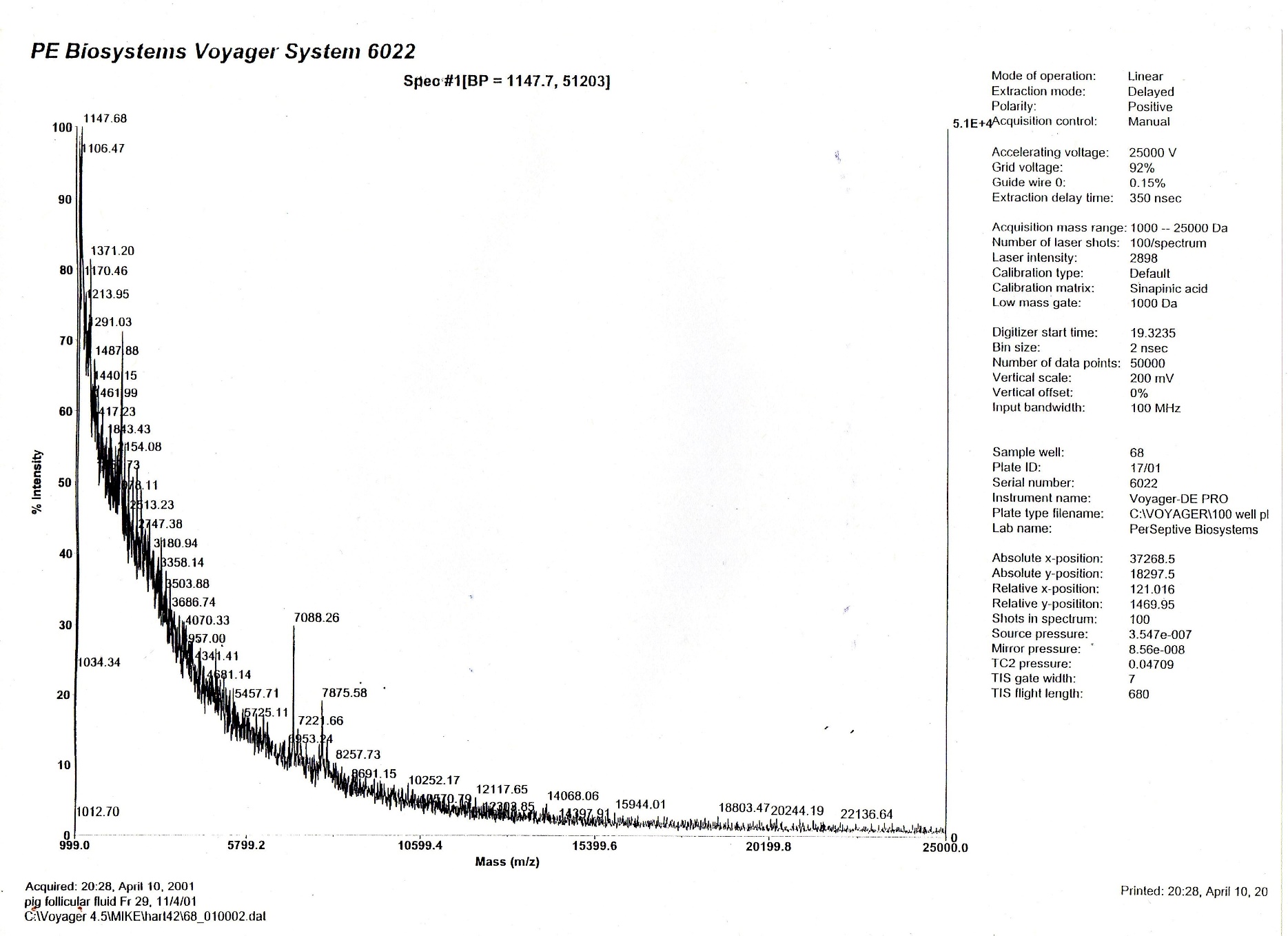


**S3 Figure 2.** Pig ovarian follicular fluid anionex Fraction 29, obtained by the Babraham method of purification (S1). An apparent pSgII-70 **68mer** peak at *m/z* 7875.58 finds a predictive next-integer match at 7876.72, with four water losses and a difference of –1.14, and an apparent **61mer** at 7088.26 finds a match at 7092.89, with two water losses and a difference of –4.63.

The base peak at 1147.68 is an apparent pSgII-70 **11mer** (match 1153.50).


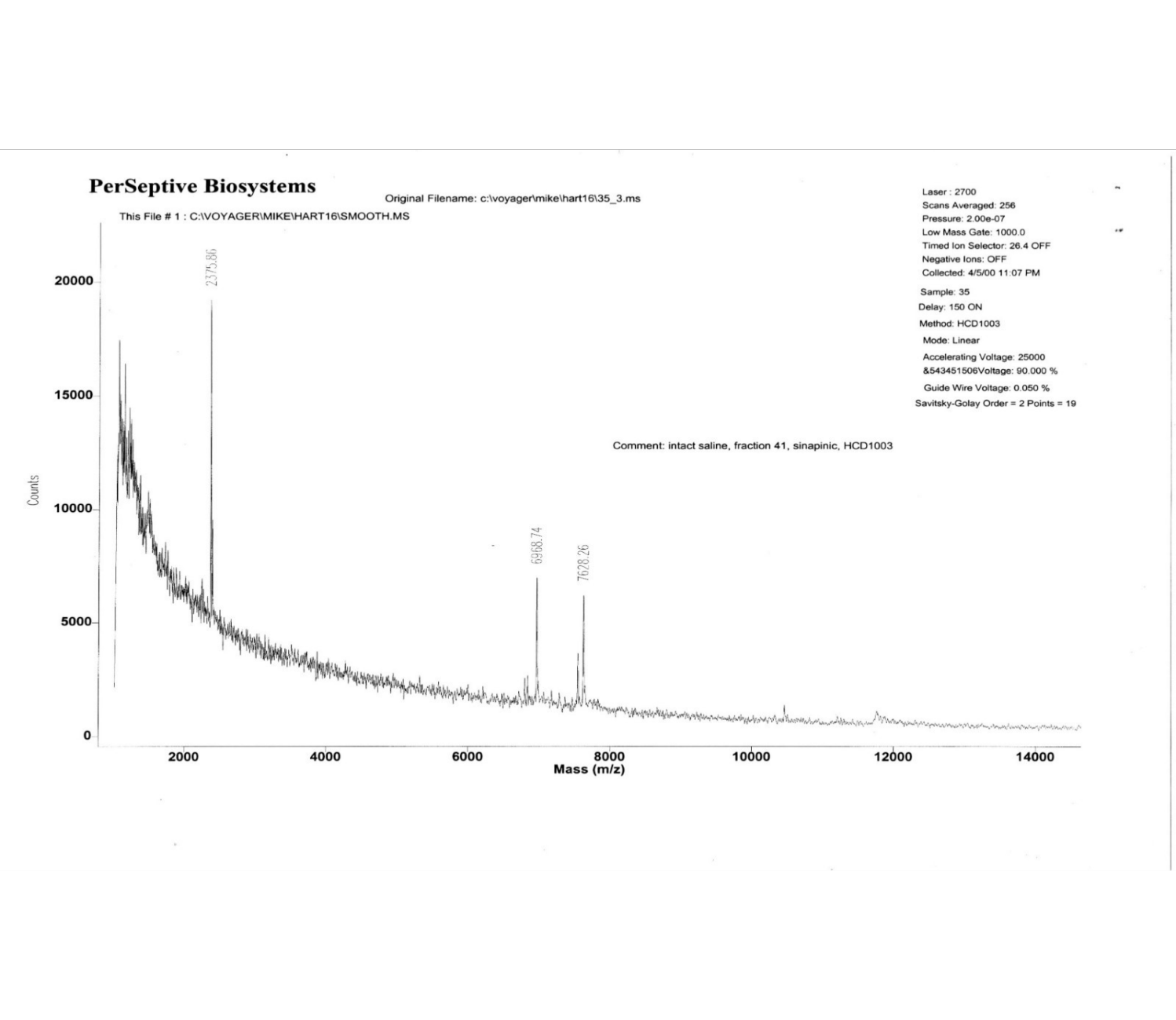


**S3 Figure 3.** Mass spectrum of FPLC anionex late-eluting fraction of ovine plasma obtained by the Babraham Method (S1). Disintegration was apparent when this anionex fraction was subjected to C18 reverse phase chromatography (RP-HPLC), as described in a Babraham lab report.


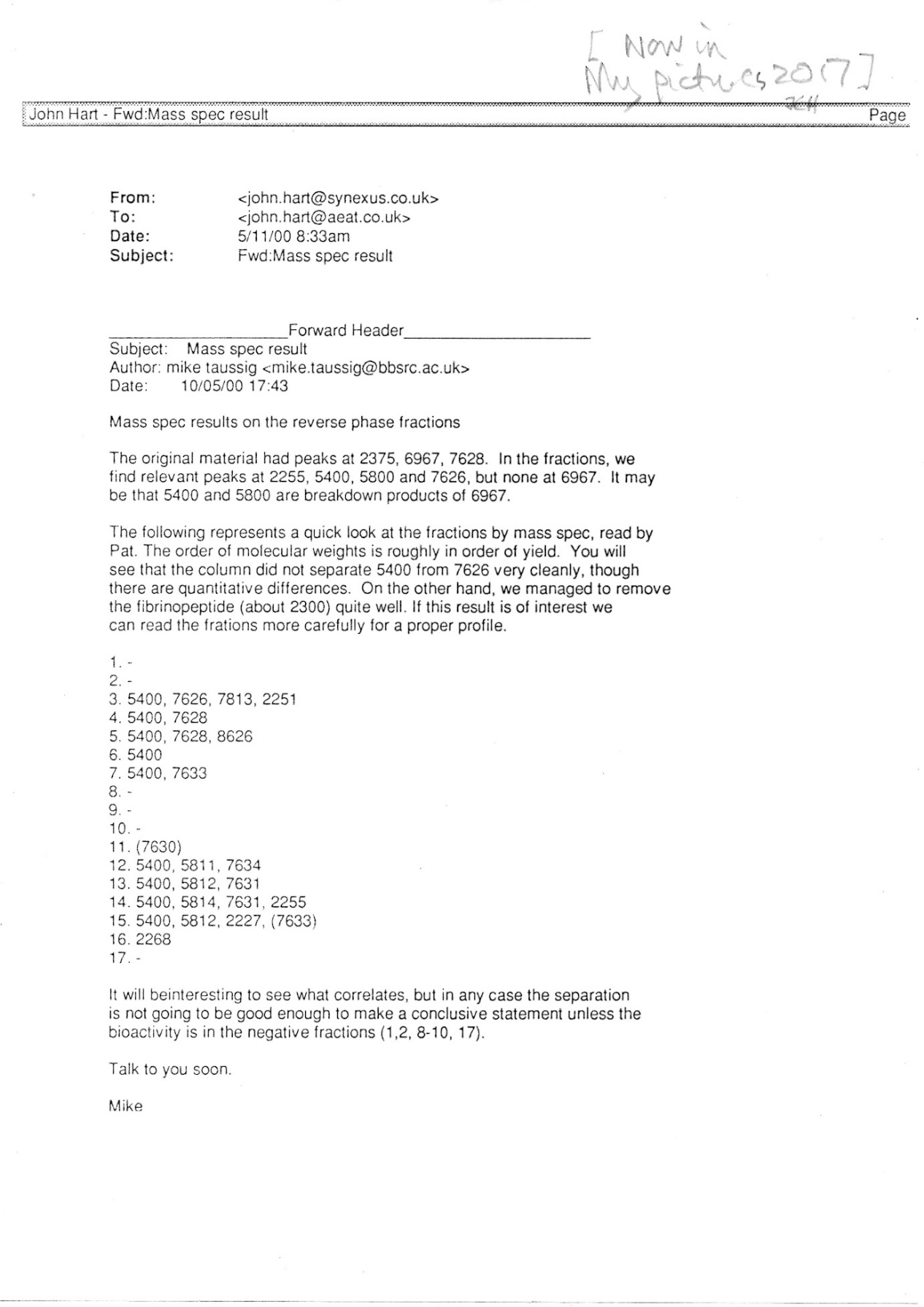


Just as there is bimodal distribution in anionex, with Candidate 7500 eluting ‘early’ and ‘late’, so there is bimodalism in RP-HPLC. Amphipathicity is indicated. The RP-HPLC fractions produced a weak double peak of activity in the BMC viability assay, with activity corresponding to the presence of Candidate 7500. Mention in the lab report of fibrinopeptide (as in S1’s discounted Candidate VI, fibrinopeptide A) is erroneous, this being *m/z* 1848. ‘Pat’ in the report is Pat Barker (personal communication, Babraham Institute, Cambridge, UK).

The peak matches to S3 Table 1 in the anionex fraction are: 7628 (7620, **66mer**), 6986 (6982, **60mer**) & 2375 (2378, **20mer**). This is within the interpretation that what is being seen are all sSgII-70 fragments. Fragmentation is thus evident in anionex and even more so in the subsequent RP fractionation, with Candidate 7500 and N-terminal Grand Fragments thereof distributed bimodally in both.

The actual peak values (i.e. not the rough estimates in the lab report) in the positive RP-HPLC fractions (3-8, 11-16), in yield order and analysed for sSgII-70 (S3 Table 1), are as follows, providing a picture of rampant fragmentation.

3: 5395 (**48mer** match 5392), 7626 (**66mer** match 7620), 7813 (**67mer** match 7817), 2251 (**19mer** next-integer match 2250)

4: 5400 (**48mer** match 5410), 7628 (**66mer** match 7620)

5: 5398 (**48mer** match 5392), 7630 (**66mer** match 7638), 8626 (see S3 Fig. 5 for a consideration of ‘Superheavyweight’ dimers)

6: 5398 (**48mer** match 5392)

7: 5394 (**48mer** match 5392), 7633 (**66mer** match 7638)

11: 7630 (**66mer** match 7638)

12: 5399 (**48mer** match 5392), 5811 (**51mer** match 5806), 7634 (**66mer** match 7638)

13: 5399 (**48mer** match 5392), 5812 (**51mer** match 5806), 7631 (**66mer** match 7638)

14: 5398 (**48mer** match 5392), 5814 (**52mer** match 5806), 7631 (**66mer** match 7638), 2255 (**20mer** match 2252)

15: 5401 (**48mer** match 5410), 5812 (**51mer** match 5806), 2227 (**19mer** match 2232), 7633 (**66mer** match 7638)

16: 2268 (**20mer** match 2270).


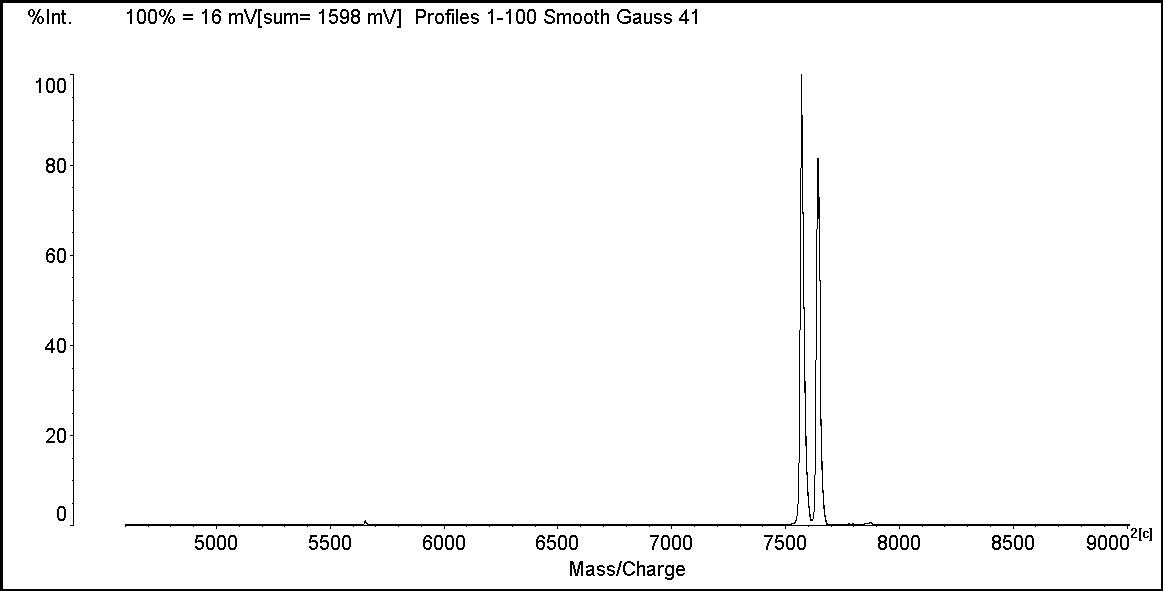


**S3 Figure 4.** Bovine ovarian follicular fluid, upstream precipitate (S1), added without treatment to a protein matrix. This mass spectrum, which reproduces the result shown in the paper’s Fig. 4, upper panel, was obtained on a Kratos Kompact SEQ MALDI-TOF mass spectrometer, in linear mode with pulsed extraction set at 7000 Da, using sinapinic acid as matrix and calibrated against insulin. The two well-defined peaks are at 7572.19 and 7642.96. The separation between these peaks is consistent with protein chains differing by an alanine residue. Analysis using S3 Table 2 MS heterogeneity analysis for Bovine Candidate 7500 indicates however that the more prevalent 7572.19 item is a bSgII-70 **65mer** (S3 Table 3 match 7574) lacking –KANNI and a single water molecule, while the 7642.96 item is a **66mer** (match 7648) lacking –ANNI and four water molecules, explaining the ‘alanine difference’. Mass spectrum courtesy of Carolyn Carr, University of Oxford, Oxford, UK.


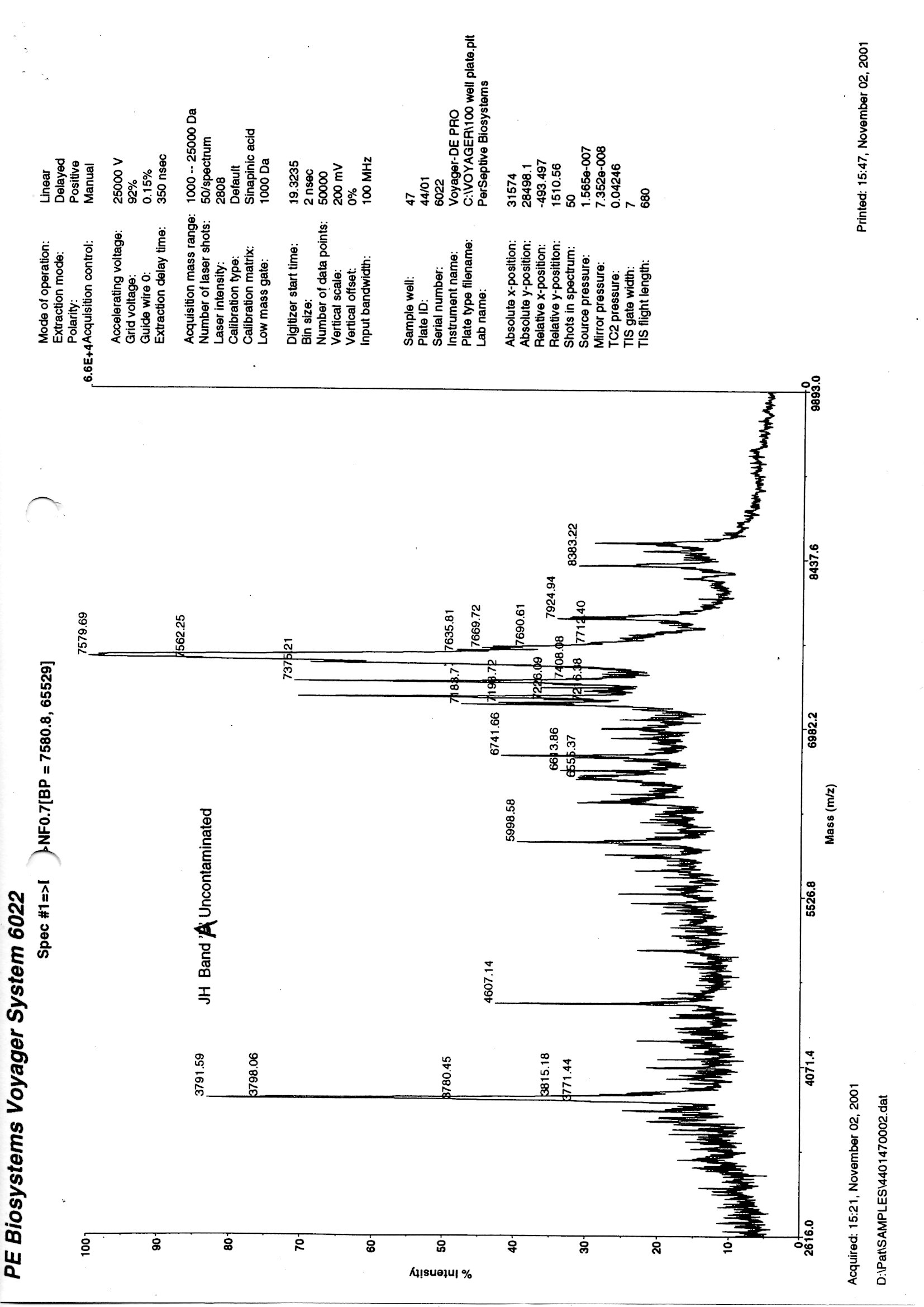


**S3 Figure 5**. MALDI-TOF mass spectrum of a solid ‘upstream precipitate’ that formed on the 3 kDa membrane during a 3-30 kDa ultrafiltration of ovine jugular vein EDTA plasma. Matrix sinapinic acid, calibration against carbonic anhydrase. This spectrum relates to a forerunner preparation to that represented by the paper’s Fig. 5. The latter, displaying an uncluttered Candidate 7500 spectrum, was the result of electroelution of SDS-PAGE Band 1 at ~7 kDa (S1, Sheffield Method), which had cell-inhibitory bioactivity in vitro. In the present case, proteins were extracted from the gel by a solvent extraction method and the soluble material run on MALDI-TOF for determination of accurate molecular weights.

Sample designation: JH = John E Hart, co-author; hand correction to **‘A’** by Pat Barker (personal communication, Babraham Institute, Cambridge, UK), where Band A = Band 1; ‘Uncontaminated’ = sample lacking in apparent bacterial contamination, with sodium azide used in the purification procedure as an antimicrobial agent.

The following analysis of prominent peak *m/z* values in S3 Fig. 5 is based on S3 Table 1.

3791 (**33mer** match 3789)

4607 (**40mer** match 4613)

5998 (**53mer** match 5992)

6741 (**59mer** integer match 6741)

7579 base peak (**65mer** match 7582)

7924 (**69mer** match 7919)

∑Observed (n = 6) = 36640

∑Matches = 36636

∑Observed/∑Matches x100 = 36640/36636 x100 = **100.01%**

As in the paper’s Fig. 5, the base peak here is analysed to be a **65mer**, i.e. sSgII-70 lacking –KANNI, with two water losses. But whereas Fig. 5 is uncluttered, the present spectrum resembles more the lower panel of the paper’s Fig. 4, except that the minor peaks are higher in relation to the base peak and there is a raggedness to the spectrum that may be due to the presence of SDS, absent from the Fig. 4 preparation. There are multi-hundred stepped peaks (3791, 4607, 5998, 6741, 7579), analysed above to be N-terminal Grand Fragments. The ion at 8383 is a Superheavyweight in that it is above the predicted mass of sSgII-70 MH^+^ at *m/z* 8176. The 8383 Superheavyweight is close to the sum of the two lowest peaks: 3791 + 4607 = 8398. Heterodimerisation can be suspected, as is articulated in the paper.

The present mass spectrum, S3 Fig. 5, of *solvent extracted* EPL001 Gel Band 1, can be judged to be rampantly artefactual in terms of C-terminal molecular losses, unlike the paper’s Fig. 5 spectrum of *electroeluted* EPL001 Gel Band 1, which is restrainedly so.


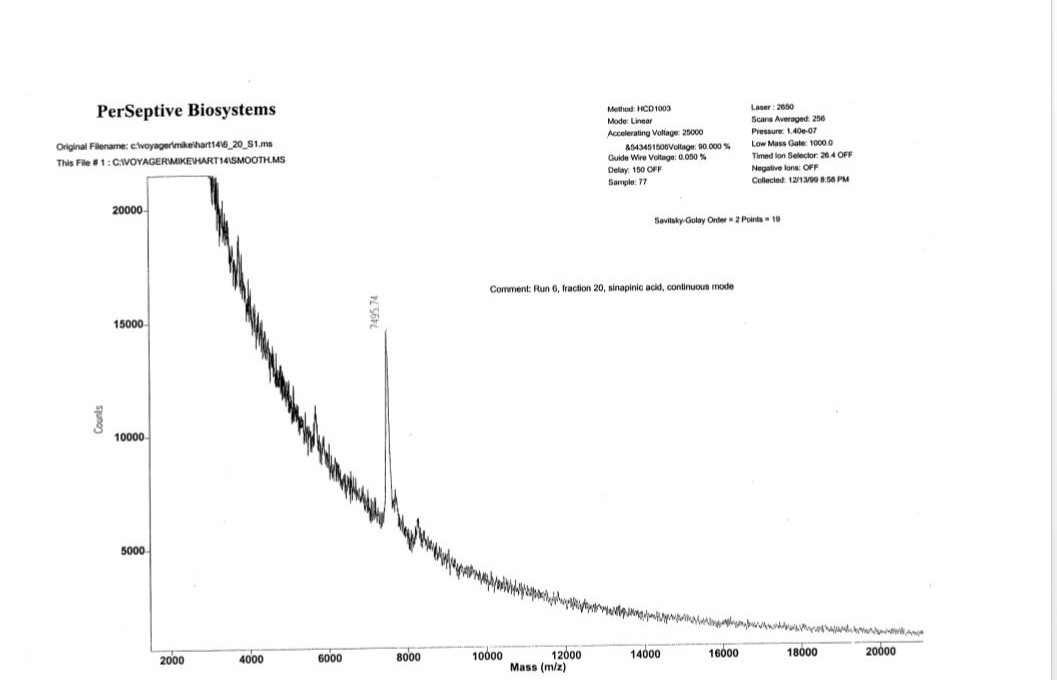


**S3 Figure 6.** MALDI-TOF mass spectrum of anionex Fraction 20 of ovine systemic plasma previously subjected to spin and gel filtration (S1 Babraham Method). Fraction 20 showed a single peak in MALDI-TOF at *m/z* 7495, betokening an sSgII-70 **65mer** (match 7492) with *seven* water losses, according to S3 Table 1. Reread within minutes using the same matrix, sinapinic acid, this fraction again yielded only a single peak, at 7550, still corresponding to a **65mer** (match 7546) but now with only *four* water losses. Why the different outcomes? The 7495 item was detected when the instrument was in continuous mode (this spectrum), the 7550 item when it was in delayed mode (S3 Fig. 7).


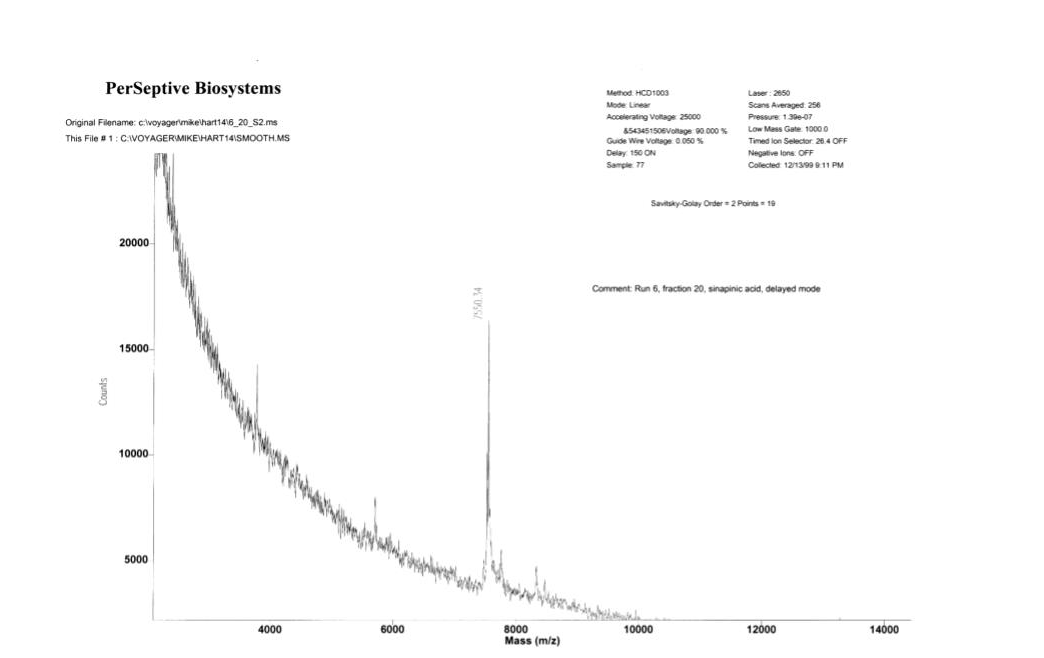


**S3 Figure 7.** See S3 Figure 6 legend.


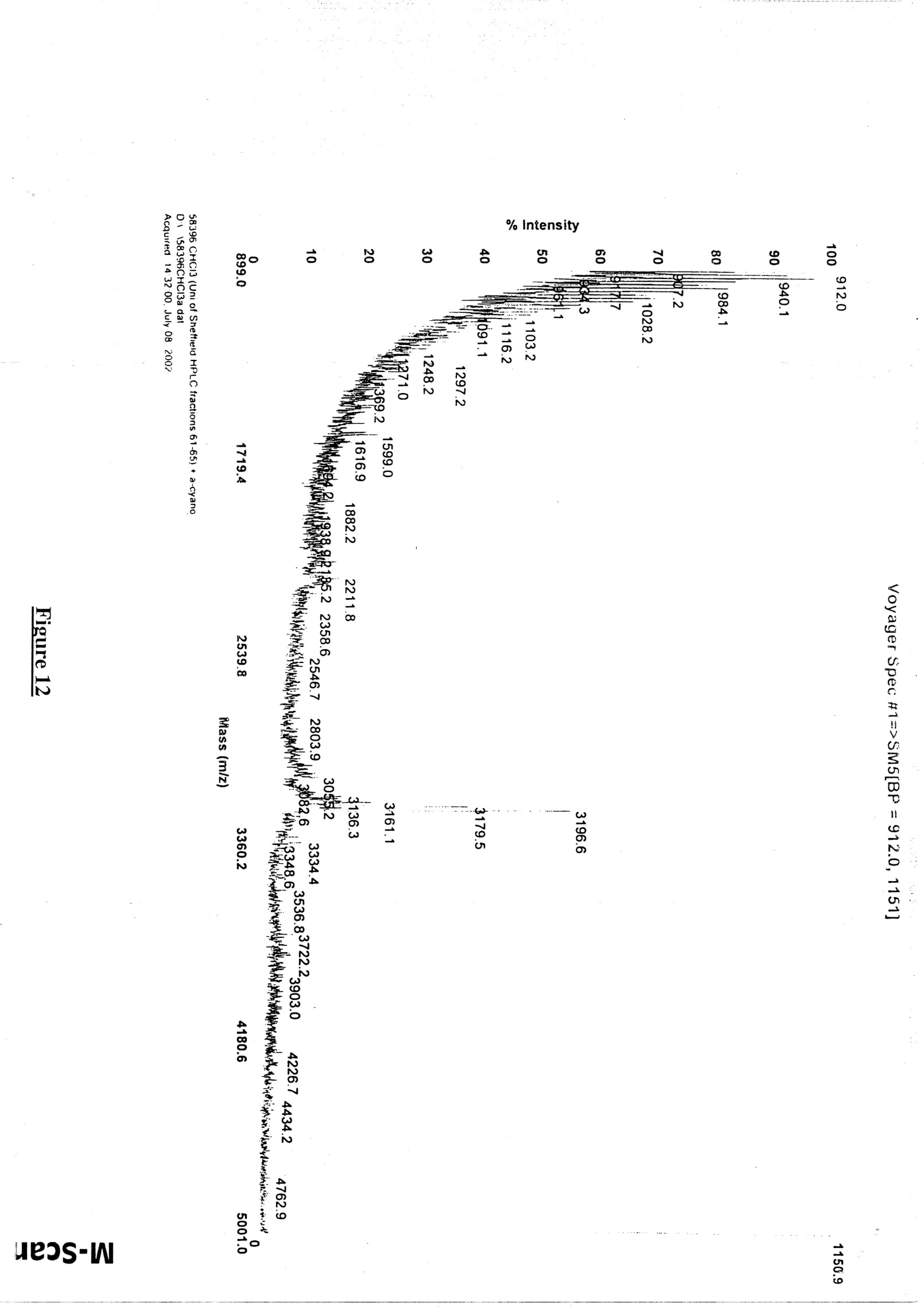


**S3 Figure 8.** Mass spectrum from a report by commercial supplier M-Scan of Wokingham, UK, entitled ‘MALDI-TOF…Analysis of Samples Eluted from an Ion Exchange Column.’ Ovine jugular vein EDTA plasma was subject to ultrafiltration by the Sheffield Method (S1), followed by HPLC anionex. Fractions 61-65, active in vitro and pooled, showed ions at *m/z* 3196, 3179 & 3161. These peaks find a trio of adjacent water-gapped matches in S3 Table 1 on the **28mer** tier at 3195, 3177 & 3159. Ions from this group appeared in five fraction pools involving the use of the MS matrix chemical α-cyano-4-hydoxycinnamic acid (CHCA), with the instrument in reflectron mode (S3 Table 5, below). When the instrument was used in linear mode with sinapinic acid as an alternative matrix five fraction pools showed peaks matching a **29mer** in S1 Table 1, at around 3273. Extracts from the M-Scan report are reproduced below, with the *m/z* 3000 results tabulated as S3 Table 10.


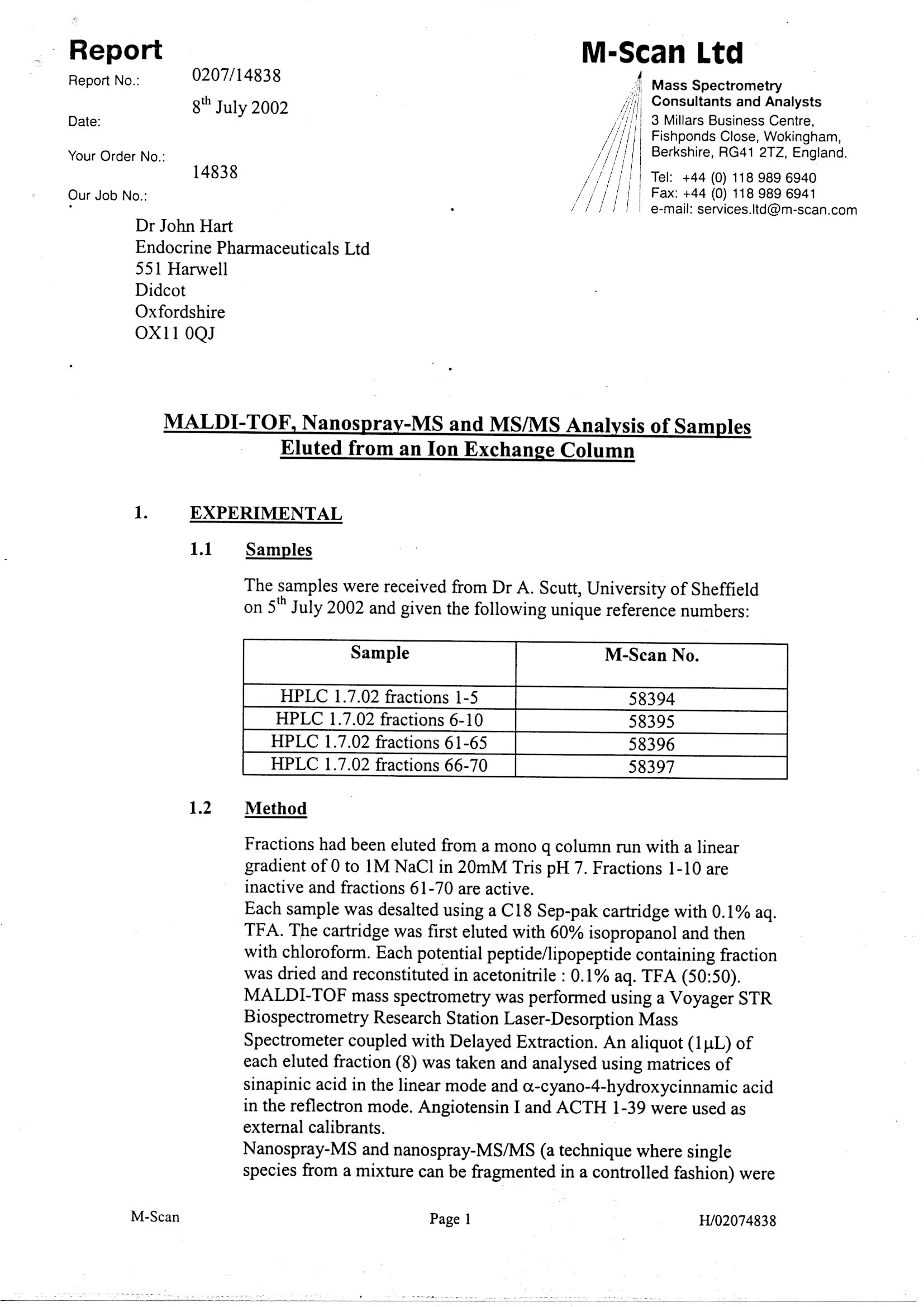


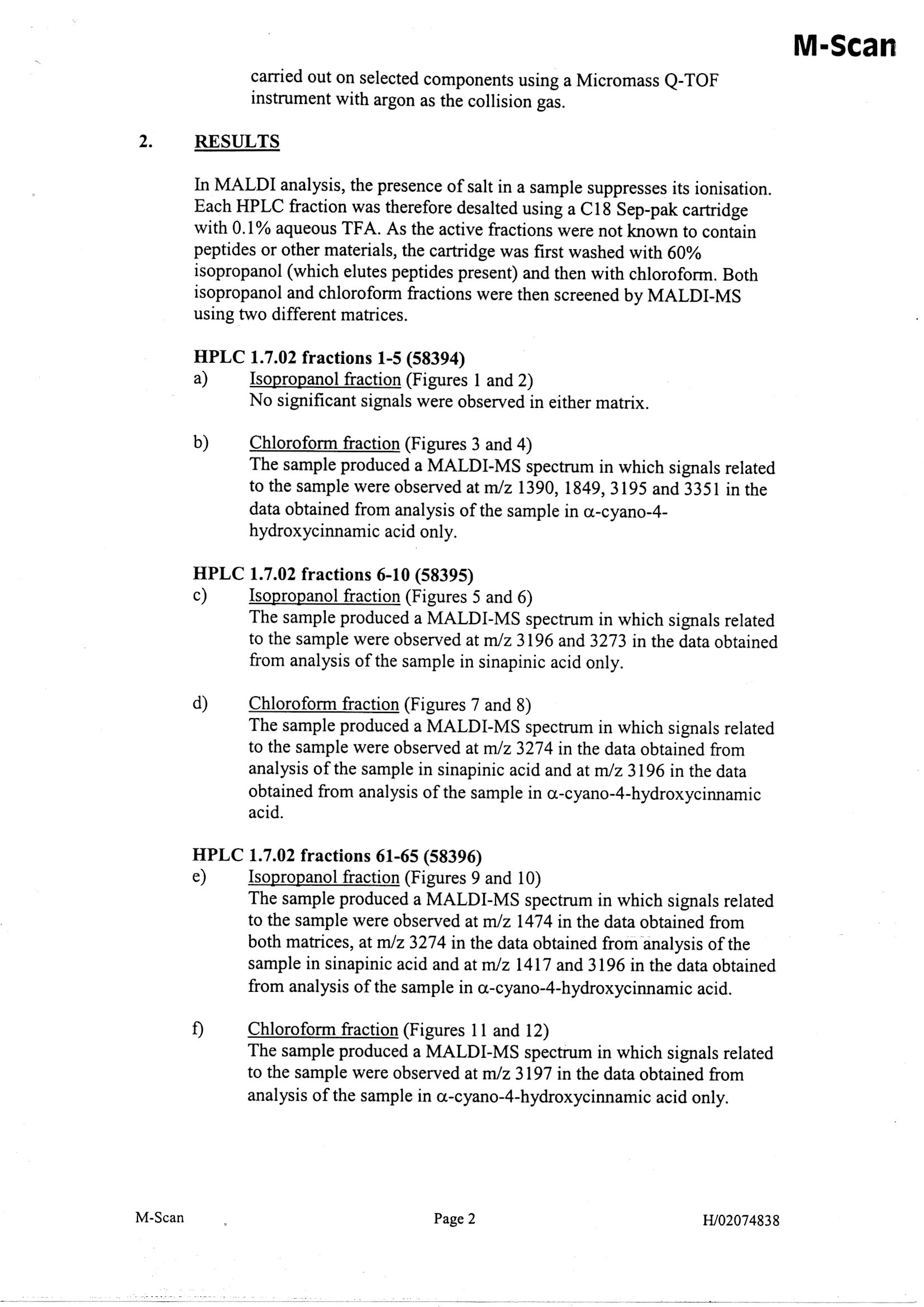


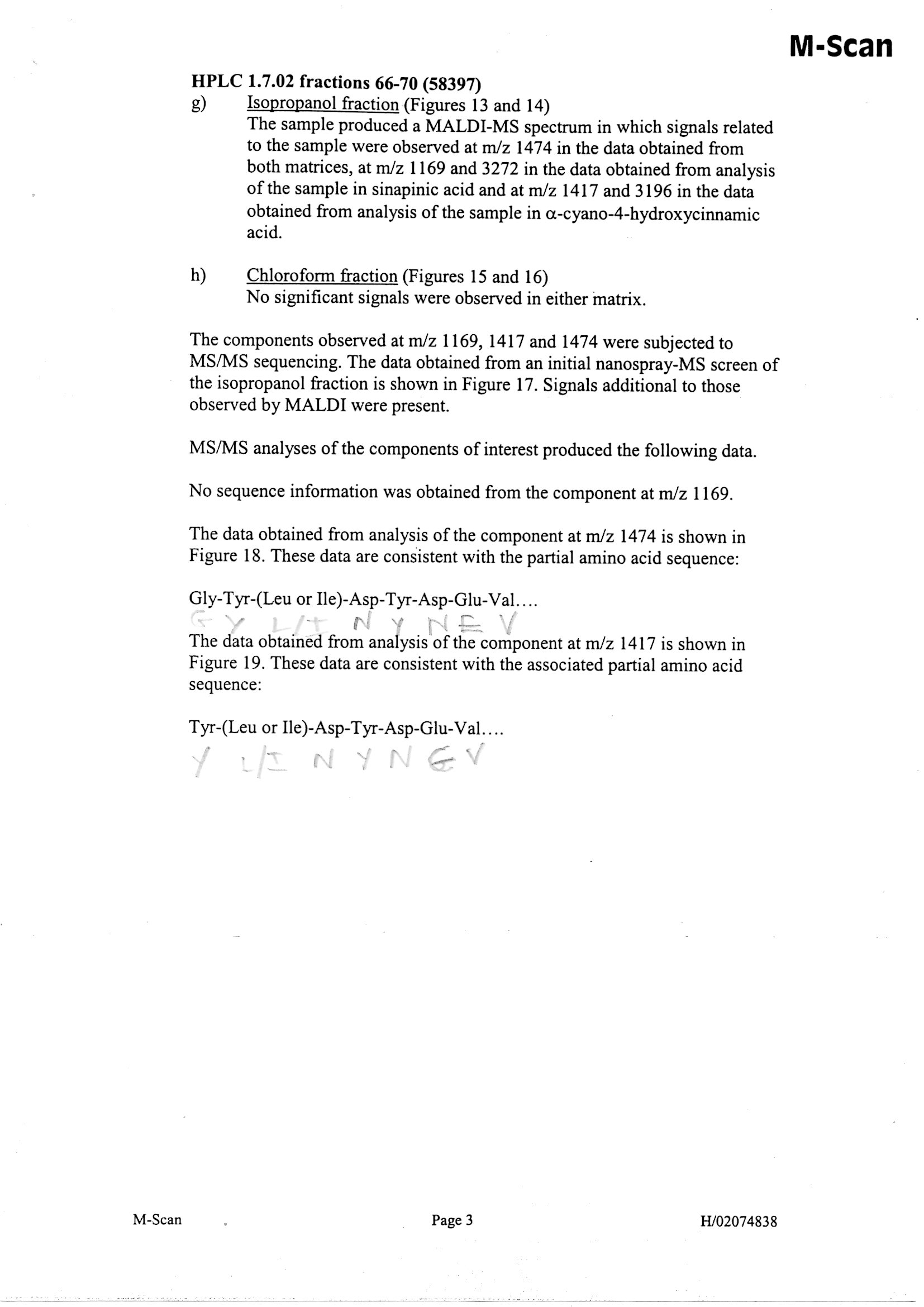


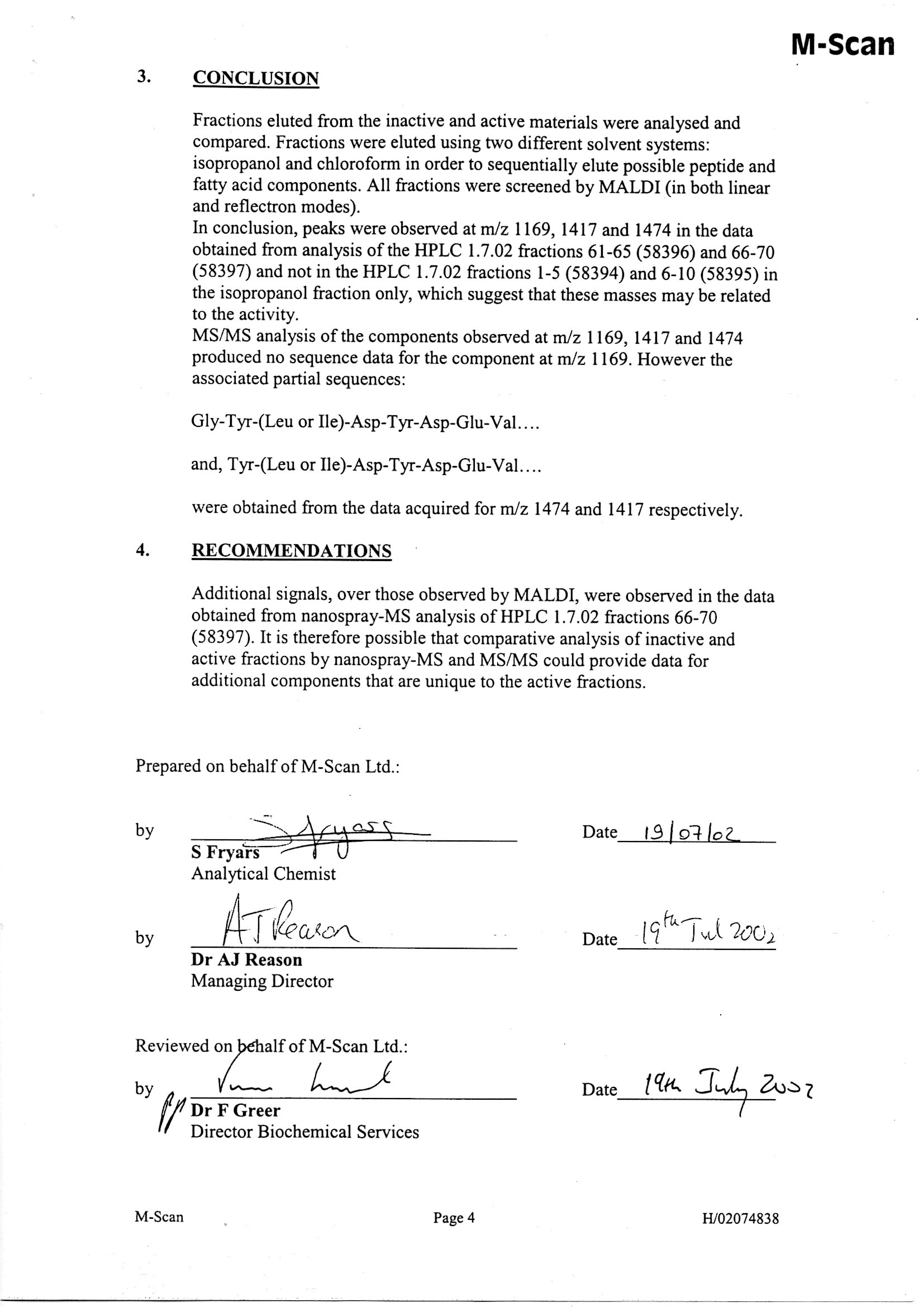


In regard to MALDI-MS peaks at *m/z* 1474 and 1417, MS/MS sequencing yielded short stretches of aa denoting the presence of fibrinogen-related ions. This is discounted Candidate VI, fibrinopeptide A (S1).

The peaks in the *m/z* 3000s were not investigated further. These data are shown in tabular form below.

**S3 Table 10.** Peaks in the *m/z* 3000s seen in MALDI-MS when HPLC anionex fractions of ovine systemic blood plasma were investigated (with sSgII mer deductions and matches in brackets, as per S3 Table 1).

| Fraction pools | Isopropanol | | Chloroform | |
| --- | --- | --- | --- | --- |
|  | Sinapinic acid | CHCA | Sinapinic acid | CHCA |
| 1-5 | - | - | 3429 (**30**, 3437)  3272 (**29**, 3272) | 3351 (**29**, 3344)  3194 (**28**, 3195) |
| 6-10 | 3272 (**29**, 3272)  3196 (**28**, 3195)  3294 (**29**, 3290) | - | 3274 (**29**, 3272) | 3195 (**28**, 3195)  3178 (**28**, 3177)  3161 (**28**, 3159) |
| 61-65 | 3274 (**29**, 3290)  3327 (**29**, 3326) | 3195 (**28**, 3195)  3179 (**28**, 3177)  3160 (**28**, 3159) | - | 3196 (**28**, 3195)  3179 (**28**, 3177)  3161 (**28**, 3159) |
| 66-70 | 3271 (**29**, 3272)  3293 (**29**, 3290) | 3195 (**28**, 3195)  3160 (**28**, 3159) | - | - |

Anionex samples desalted using a C18 Sep-Pak cartridge (Waters) with 0.1% aq. TFA. The cartridge was first eluted with 60% isopropanol (to elute peptides present) and then with chloroform (to elute lipopeptide and other lipid material). For other methodological details see above.

Peaks (*m/z*) ranked by intensity, the more intense above the less intense. Figures in brackets are matches in S3 Table 1, tiers **28-30mers**.

Sinapinic acid matrix used with MS instrument in linear mode: mainly detected putative **29mers** at circa 3272 and a water above at 3290.

CHCA matrix used with MS instrument in reflectron mode: mainly detected putative **28mers** in the water gapped series 3195, 3177 & 3159.

∑Observed (n = 23) = 74406

∑Matches = 74402

∑Observed/∑Matches x100 = 74406/74402 x100 = **100.01%**

An analysis in terms of sSgII-70 can be attempted of the ramp peaks in S3 Fig. 8, using Expasy Compute pI/Mw for sub-10mers:

912 base peak (**9mer** MH^+^ at 986 reduced by 4 x 18.015, being four water losses, match 914)

940 (**9mer** MH^+^ at 986 reduced by 3 x 18.015, being three water losses, match 932)

984 (**9mer** MH^+^ at 986, providing a match)

1028 (**10mer** S3 Table 1 match 1025)

1103 (**10mer** S3 Table 1 match 1097)

1116 (**10mer** S3 Table 1 next-integer match 1115)

∑Observed (n = 6) = 6083

∑Matches = 6069

∑Observed/∑Matches x100 = 6083/6069 x100 = **100.23%**

S3 Table 5 also appears as S1 Table 2, being subject to a separate analysis in regard to early and late eluting anionex fractions.

**S3 Figure 9**. MALDI-TOF mass spectrum of bovine follicular fluid, anionex Fraction 28 (coauthor RPN, University of Swansea, Swansea, UK, using an Applied Biosystems DE-STR Voyager instrument in linear mode). Matrix: 10mg/mL sinapinic acid in 70/30 0.3%TCA/CAN (PerSeptive Biosystems/AB). Sample was 1:50/1:100. Calibrant panel: insulin (bovine), thioredoxin (*E. coli*) and apomyoglobin (horse). Purification was by the Sheffield Method (S1). MS analysis after 5 year’s sample storage.

Observations in the 3000s from S3 Fig. 9 (with matches to S3 Table 3):

3212 (**28mer** next-integer match 3213)

3257 (**29mer** match 3254)

3274 (**29mer** match 3272)

3290 (**29mer** integer match 3290)

3306 (**29mer** match 3308)

∑Observed (n = 5) = 16339

∑Matches = 16337

∑Observed/∑Matches x100 = 16339/16337 x100 = **100.01%**

An analysis in terms of bSgII-70 can be attempted of the ramp peaks in S3 Fig. 9, using S3 Table 3:

1014 (**10mer** match 1007)

1030 base peak (**10mer** match 1025)

1072 (**10mer** match 1079)

1086 (**10mer** match 1079)

1114 (**10mer** next-integer match 1115)

1141 (**10mer** match 1135)

∑Observed (n = 6) = 6457

∑Matches = 6440

∑Observed/∑Matches x100 = 6457/6440 x100 = **100.26%**

**S3 Figure 10.** HPLC anionex Fraction 24 of ovine ultrafiltered jugular vein EDTA plasma 3-30 kDa fraction (S1, Sheffield Method) after five year’s storage. (For MS details, see S3 Fig. 9.)

Fifteen annotated peaks from the S3 Fig. 10 spectrum are analysed in the following table, including ten Superheavyweights, i.e. ions above the predicted MH^+^ mass of sSgII-70 at *m/z* 8176.

**S3 Table 11**. Putative sSgII-70 homodimers (x2) and multi-homodimers (2+2, 2+2+2, 2+2+2+2) after five year’s storage.

| **Observed (actual peaks, *m/z*)** | **Observed (monomers calculated by division)** | **MH^+^ matches in S3 Table 1 (mer) + M x n** |
| --- | --- | --- |
| 2176 | = | 2178 **(19)** + M x 0 = 2178 |
| 5356 | = | 5353 (**47**) + M x 0 = 5353 |
| 7225 | = | 7233 (**63**) + M x 0 = 7233 |
| 7101 | = | 7100 (**62**) + M x 0 = 7100 |
| 7440 | = | 7437 (**64**) + M x 0 = 7437 |
| 14058 | ÷2 = 7029 | 7021 (**61**) + M x 1 = 14041 |
| 14252 | ÷2 = 7126 | 7118 (**62**) + M x 1 = 14235 |
| 14615 (base peak) | ÷2 = 7308 | 7306 (**63**) + M x 1 = 14611 |
| 14778 | ÷2 = 7389 | 7383 (**64**) + M x 1 = 14765 |
| 28334 | ÷4 = 7084 | 7082 (**62**) + M x 3 = 28325 |
| 28831 | ÷4 = 7208 | 7208 (**62**) + M x 3 = 28829 |
| 29031 | ÷4 = 7258 | 7251 (**63**) + M x 3 = 29001 |
| 29198 | ÷4 = 7300 | 7306 (**63**) + M x 3 = 29221 |
| 43227 | ÷6 = 7205 | 7208 (**62**) + M x 5 = 43243 |
| 64974 | ÷8 = 8122 | 8121 (**70**) + M x 7 = 64961 |
| **Total = 310596** |  | **310533** |

For the five non-dimeric peaks 2176-7440 (**19-64mers**) ∑Observed (n = 5) = 29298, ∑Matches = 29301.

So, ∑Observed/∑Matches x100 = 29298/29301 x100 = **99.99%**.

The remaining ten Superheavyweight peaks have been monomerized in Column 2 of S3 Table 11 by arithmetic division: ÷2, ÷4, ÷6, ÷8. In Column 3 the MH^+^ monomer match from S3 Table 1 has been added to an appropriate number of Ms (i.e. mass of unprotonated match) to provide a comparator for the observed peak. Homodimerization is assumed.

The analysis of this spectrum thus involves five sub-8000 non-dimeric peaks plus either ten Superheavyweight putative homodimers (Observed) or ten Superheavyweight-homodimer-matches-by-calculation (Matches), thus: ∑Observed (n = 15) 310596/∑Matches 310533 x100 = **100.02%**.

This concludes the analysis of Fraction 24.

The next spectrum in the S3 Fig. 10 series is the last and relates to Fractions 25-30 pooled. Peaks are sparse, with none in the *m/z* 7-8000 range. The main peak is at 3312 (**29mer** match 3308). There is also a modest peak at 6911 (**60mer** next-integer match 6910) and a similar-sized peak at 14427 = 2 x 7214: dimerized **63mer** next-integer match 7215).

The Fraction 23 spectrum is also sparse, with the main peak at 2100 (**18mer** match 2103) and minor peaks at 7614, 10143 & 14109.

Peaks are scanty in spectra prior to Fraction 23 in the S3 Fig. 10 series and there is an absence of peaks in the 7-8000 range or multiples thereof.


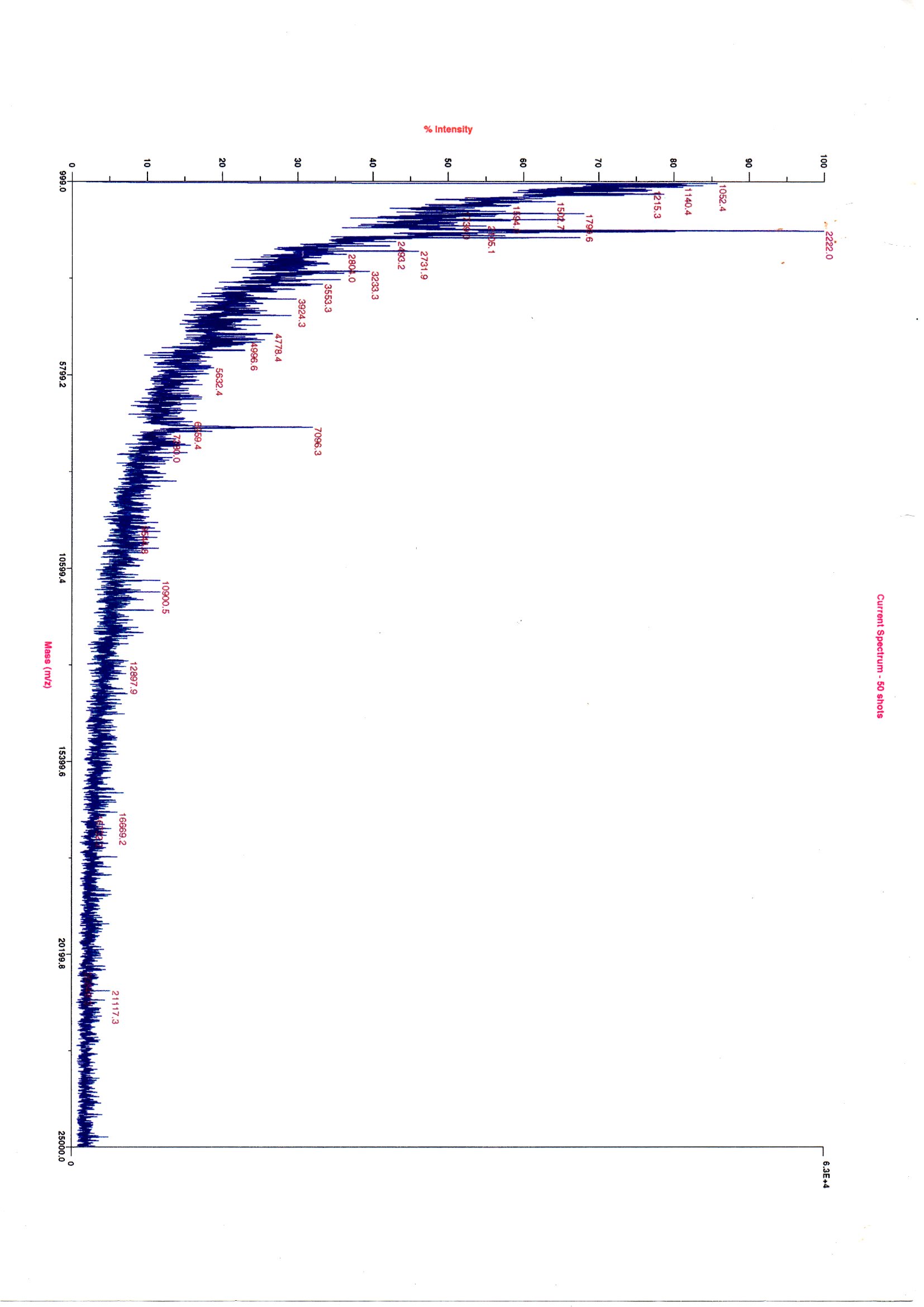


**S3 Figure 11.** Mass spectrum from Babraham of an anionex fraction of late eluting Candidate 7500 from ovine ovarian follicular fluid purified by the Babraham Method (S1). Positive ion, linear mode, sinapinic acid. This was the feedstock for Digest 7 in the trypsinisation campaign (S4).

The peak at *m/z* 7096 is interpreted as a **61mer** N-terminal fragment of sSgII-70 (S3 Table 1 match 7093).

In the paper it is contended that ovine MALDI MS peaks in the 3000s are sSgII-70 related, with support adduced from the present spectrum, which has three minor peaks of potential relevance: 3233 (**28mer**, match 3231), 3553 (**32mer**, next-integer match 3552) & 3924 (**34mer**, match 3917).

If the base peak at 2222 is interpreted as an N-terminal fragment of sSgII-70 then it is a **19mer** (match 2214). The base peak is one of 19 annotated peaks to the left of the 7096 peak. Analysis of these 19 peaks as potential sSgII-70 N-terminal fragments yields the following figures: ∑Observed (n = 19) = 55071, ∑Matches = 55025. So, ∑Observed/∑Matches x100 = 55071/55025 x100 = **100.08%**.


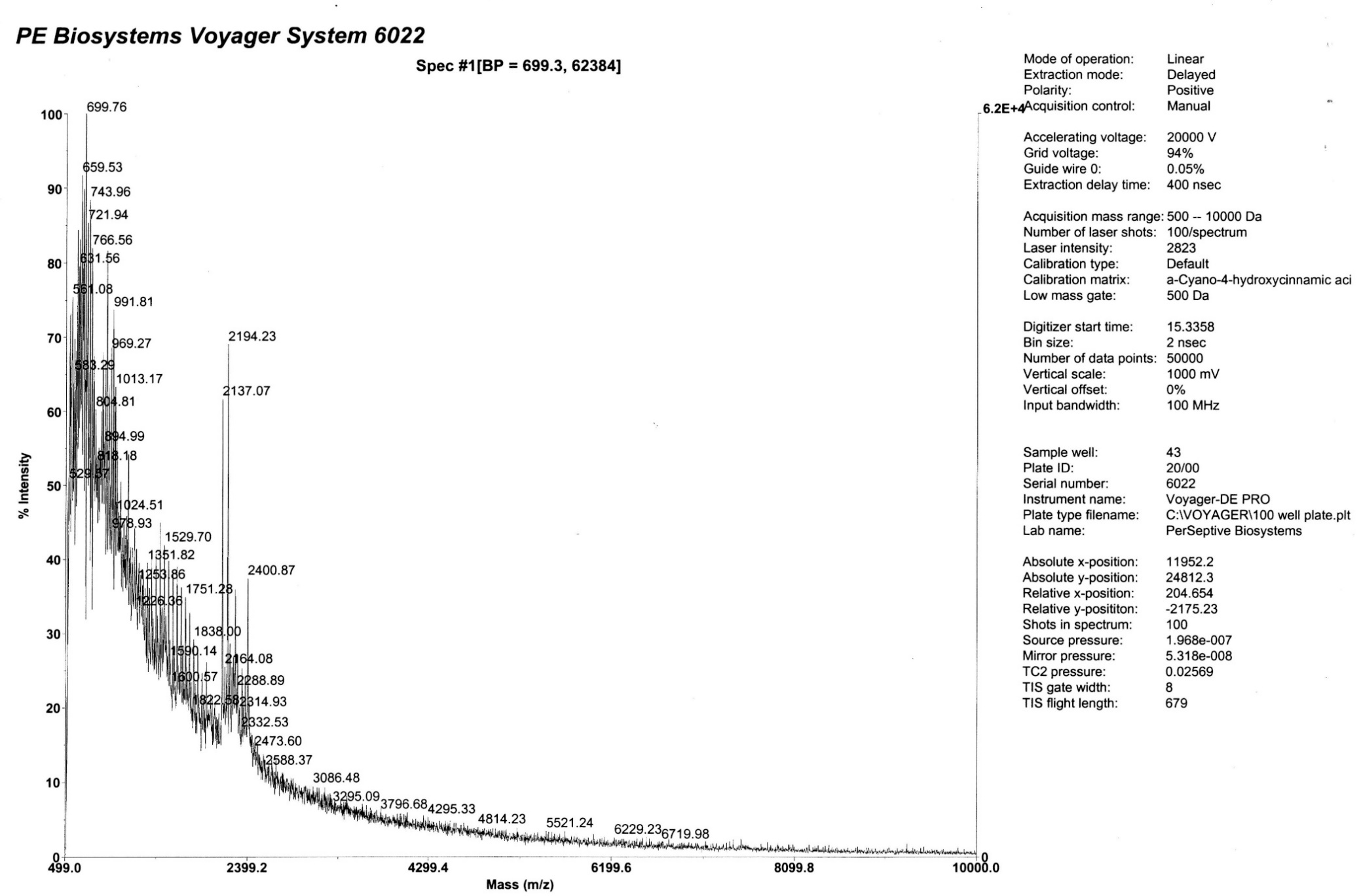


**S3 Figure 12**. Fragmentation of concentrated Ovine Candidate 7500 – with no peaks in the *m/z* 7000s evident two days after pooling of Candidate 7500 ovine blood serum anionex fractions and concentration.

The following entry in a Babraham lab notebook appeared under the heading ‘7500 material’:


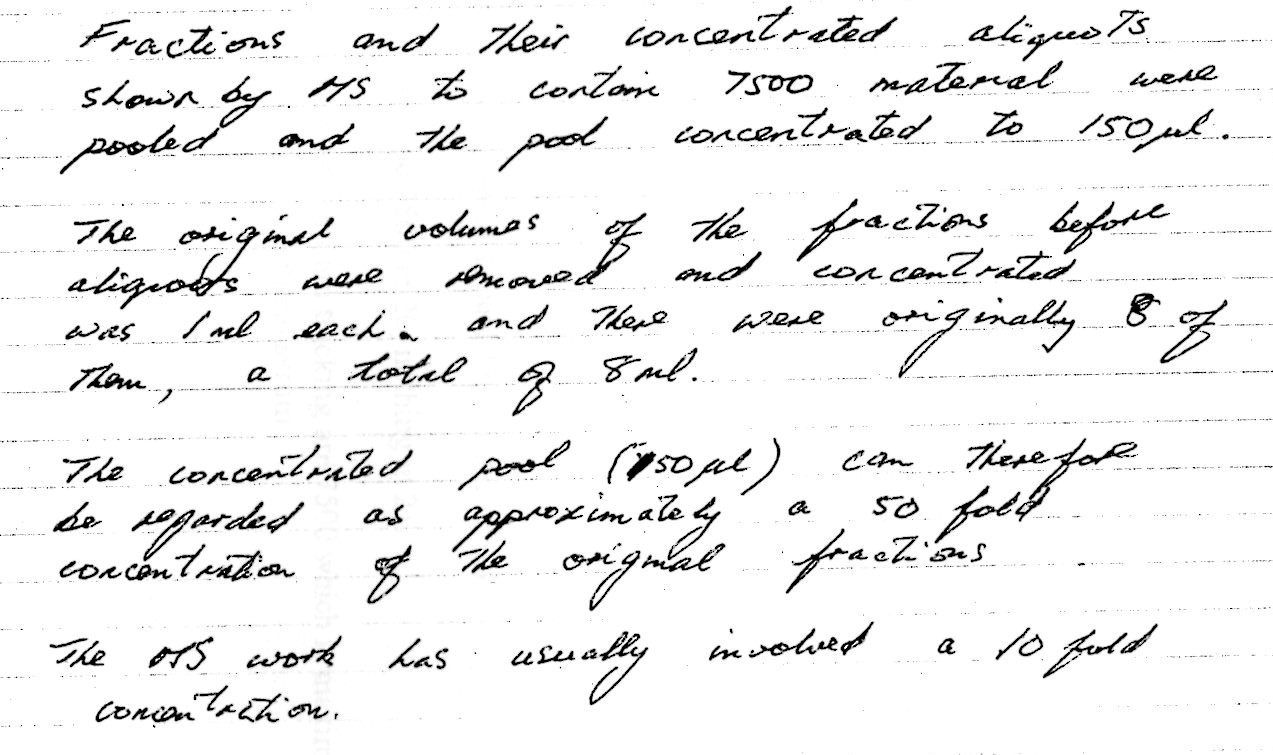


This note, penned by Dennis Beale, refers to pooled HPLC anionex Fractions 17-21 (i.e. late eluting) of systemic blood serum obtained during various phases of the oestrus cycle from different sheep. There were eight anionex fractions positive for Candidate 7500, of 1 ml each. Ovine feedstock had been purified by the Babraham Method (S1) and evaluated by MALDI two days after pooling to ~8 ml and concentration to 150 µl. (Method of concentration unspecified, but Amicon filtration was used to concentrate HPLC fractions in other experiments.)

Peaks in the 2000s can be analysed using S3 Table 1:

2137 (**19mer** match 2142)

2194 (**19mer** match 2196)

2400 (**21mer** match 2397)

∑Observed (n = 4) = 7430 (collectively connoting Candidate 7500, which is proposed to have undergone fragmentation)

∑Matches = 7434

∑Observed/∑Matches x100 = 7430/7434 x100 = **99.95%**

To capture in an sSgII-70 fragment analysis the 699 base peak (see spectrum header) the data in S3 Table 1 have to be decremented downwards, to reflect further losses of C-terminal residues and water molecules. A **7mer** N-terminal fragment of sSgII-70 (i.e. MLKTGEK) would be 788.98 Da (Expasy Compute pI/Mw). MH^+^ would therefore be 788.98 + 1.008 = 789.99. Subtracting 90.075 to reflect five water losses (i.e. H_2_O at 18.015 x 5) yields 699.92. So the summary for this ion is therefore:

699.76 base peak (**7mer** integer match 699.92, with five water losses)

Note that there is no item at 699 Da in a list of known MS contaminants that includes matrix cluster ions (Keller et al, 2008).


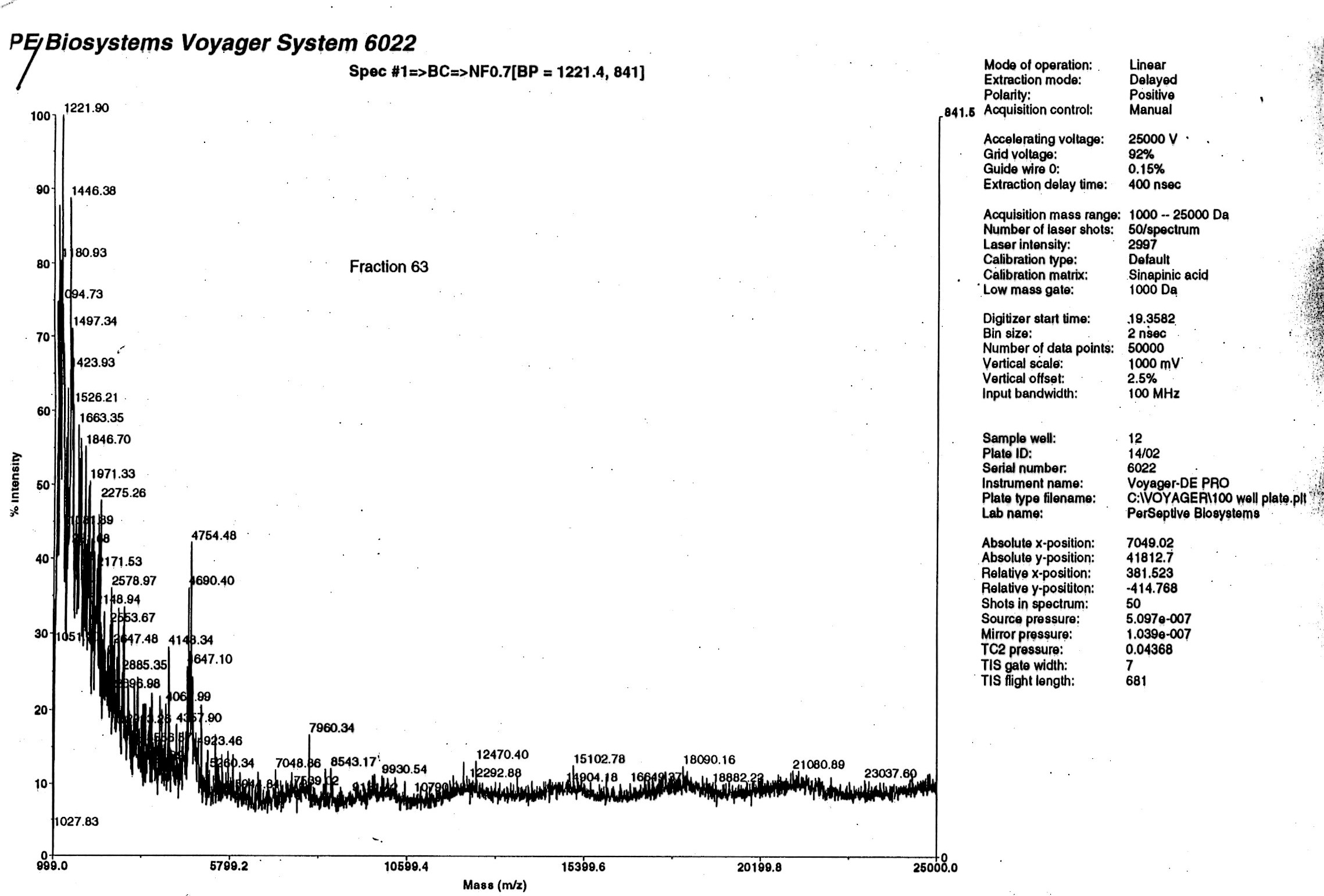
**13a**


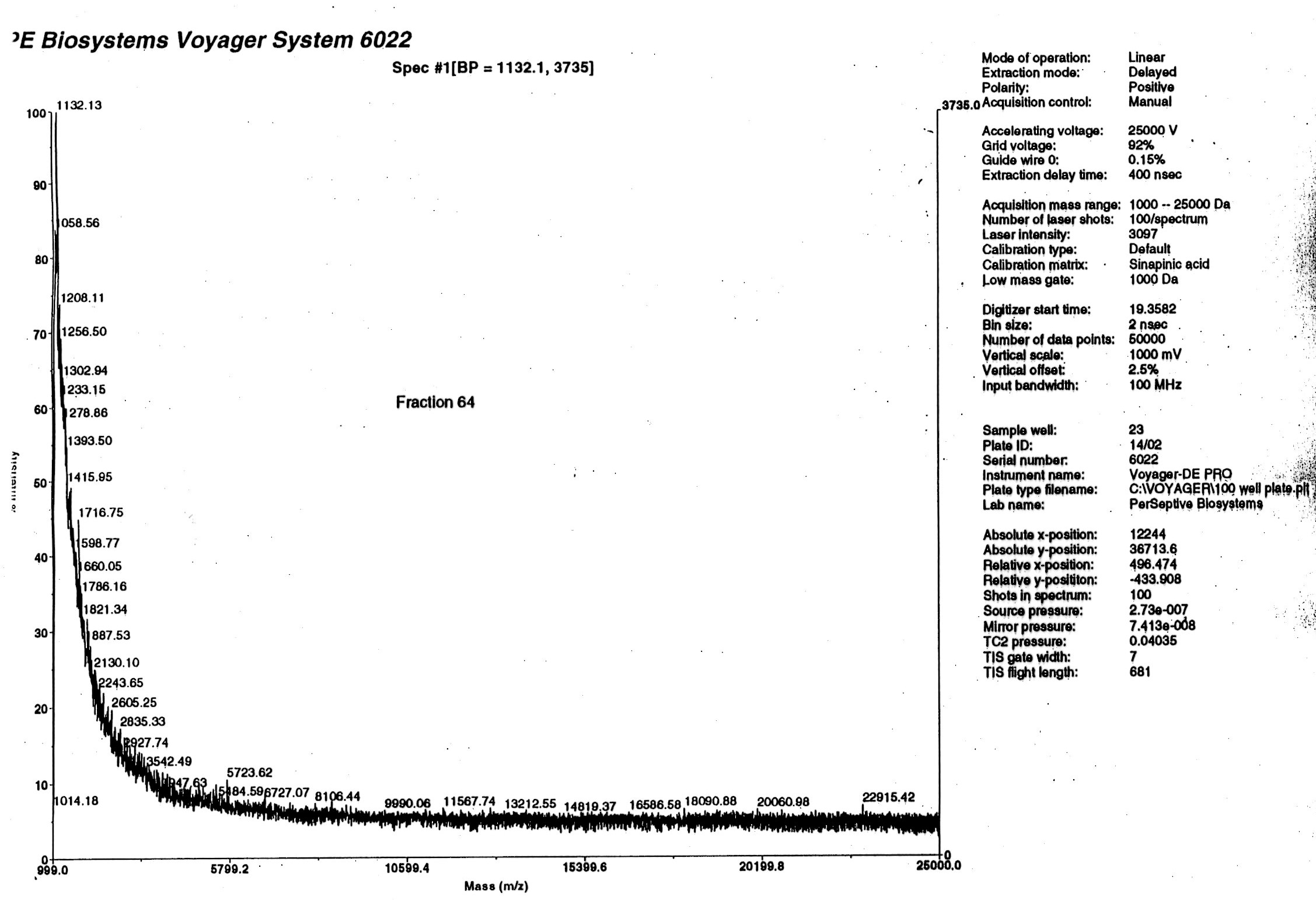
**13b**


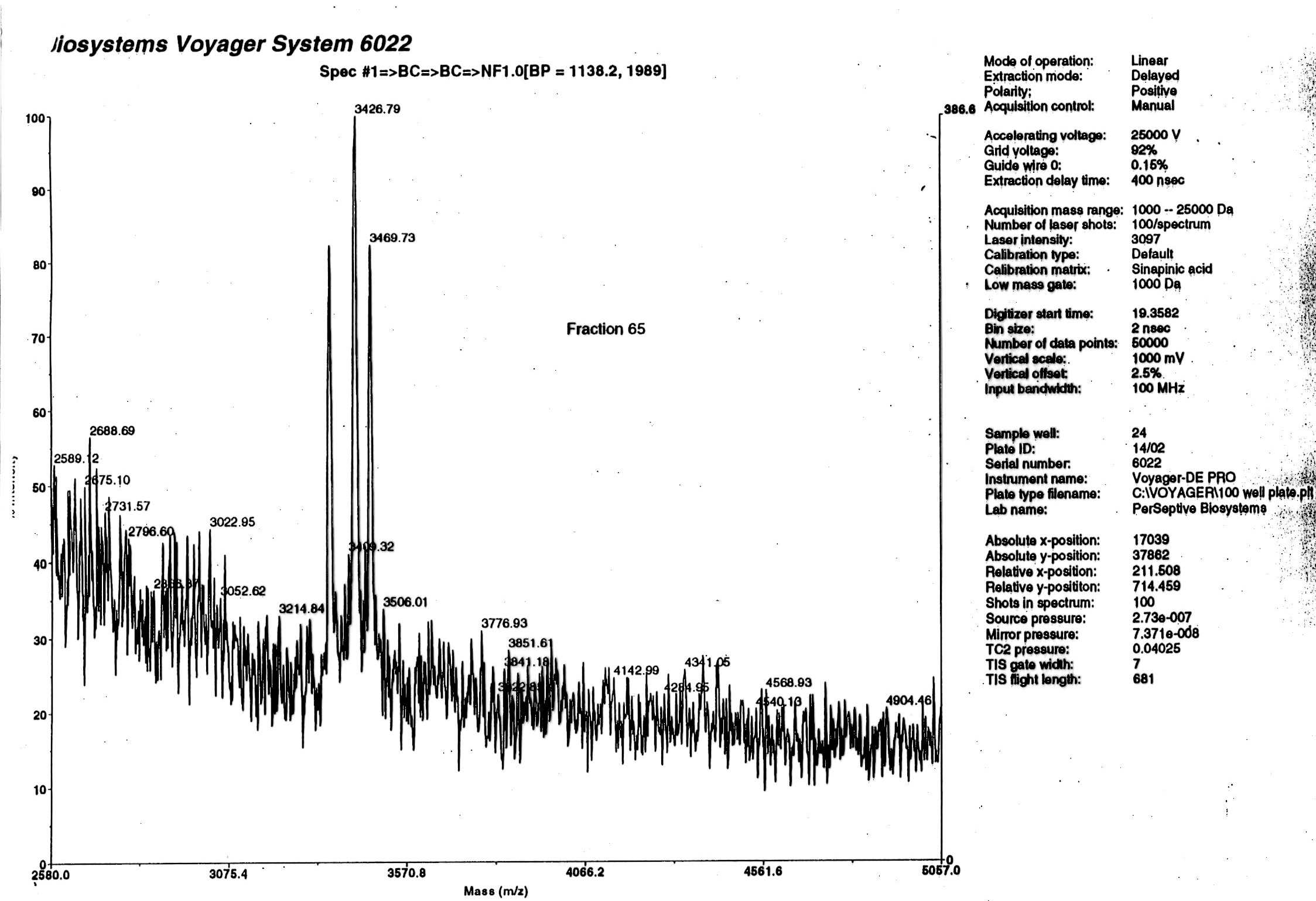
**13c**


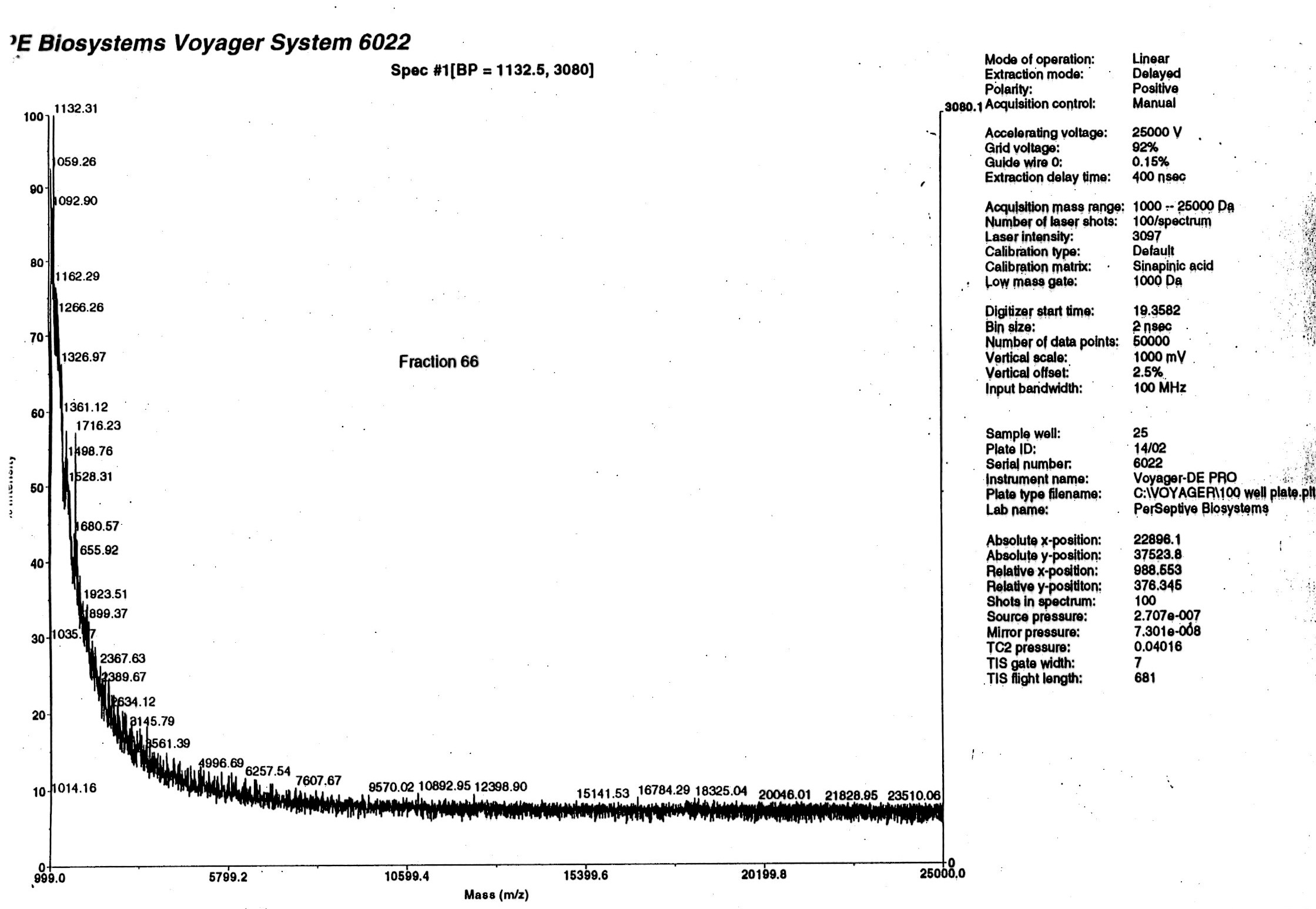
**13d**

**S3 Figure 13a-d.** MALDI mass spectra of HPLC anionex Fractions 63-66 (i.e. late eluting) of ovine jugular vein plasma subject to prior ultrafiltration (S1 Sheffield Method).

The Babraham lab notebook record is as follows: ‘Agreed to dry on Savant [vacuum concentrator] (Solvent ~20mMTris ~0.8MNaCl) ~5ml each. …[Samples] dried to small volume - <0.5 ml in Savant. Heavy white ppt found. All in 0.5ml Total Volume. Ran on Maldi, found little masses in F63 (~4.5-5) & F65 (~3.5k)’ (Pat Barker, personal communication, The Babraham Institute, Cambridge, UK.)

The four fractions are analysed here for potential sSgII-70 matches in S3 Table 1.

Fraction 63 (S3 Fig. 13a):

1221 base peak (**11mer** match 1225)

4754 (**43mer** next-integer match 4755)

7960 (**69mer** match 7955)

Fraction 64 (S3 Fig. 13b):

1132 base peak (**10mer** next-integer match 1133)

1058 (**10mer** match 1061)

Fraction 65 (S3 Fig. 13c):

1138 base peak from spectrum header (**11mer** match 1135)

3426 (**30mer** match 3419)

3469 (**31mer** match 3471)

Fraction 66 (S3 Fig. 13d):

1132 base peak (**10mer** next-integer match 1133)

1059 (**10mer** match 1061)

∑Observed (n = 10) = 26349

∑Matches = 26348

∑Observed/∑Matches x100 = 26349/26348 x100 = **100.00%**


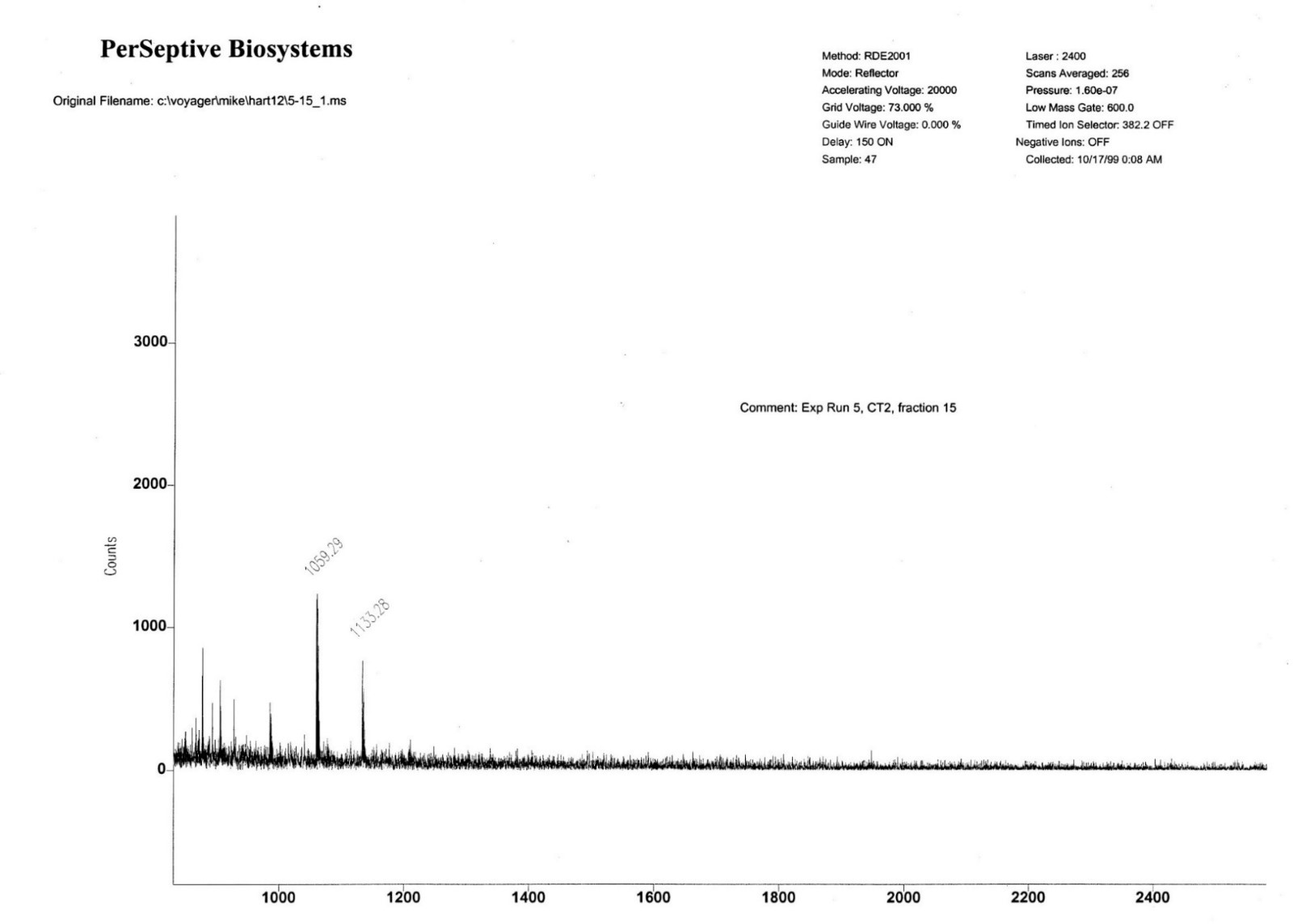


**14a**


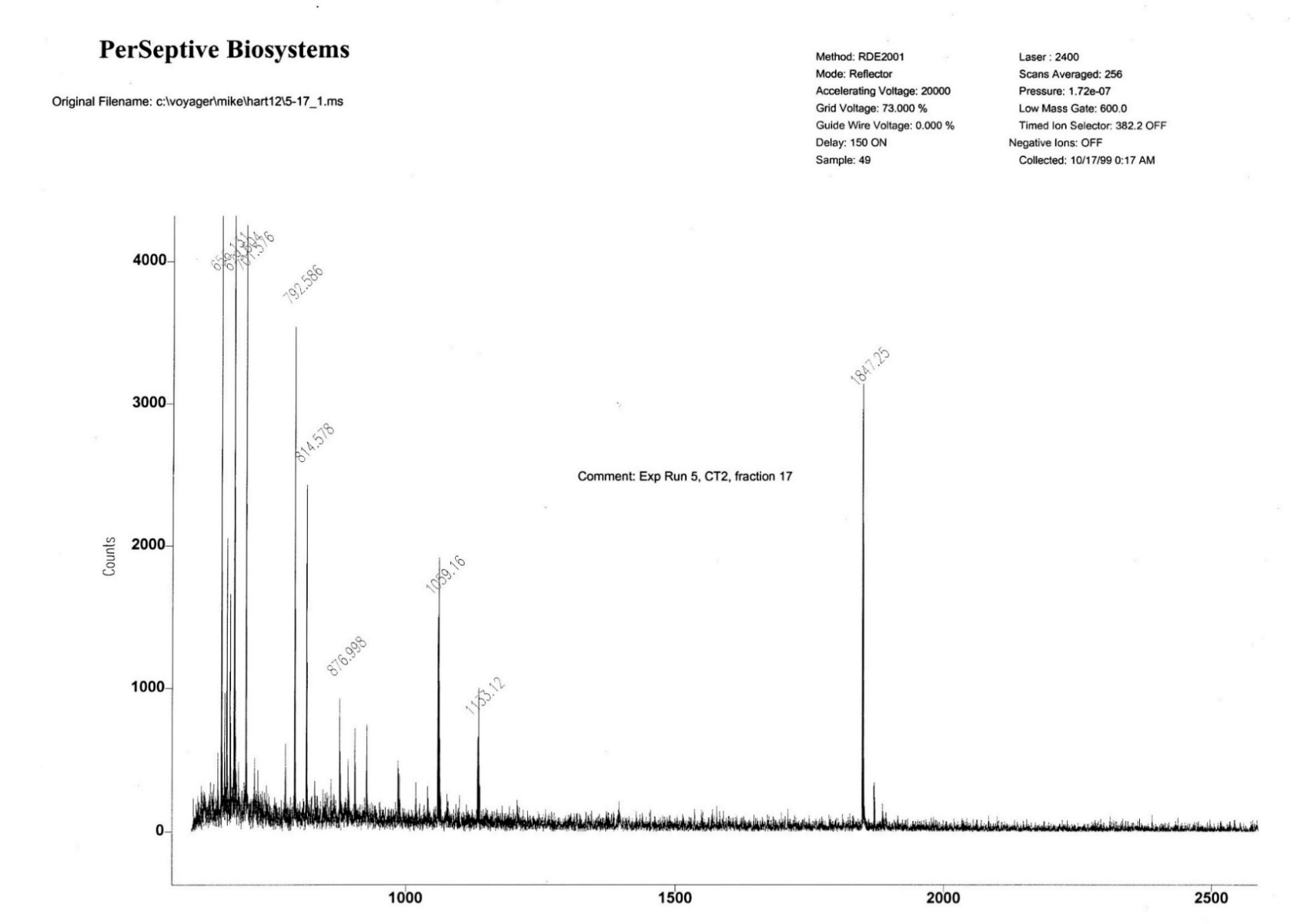
 **14b**


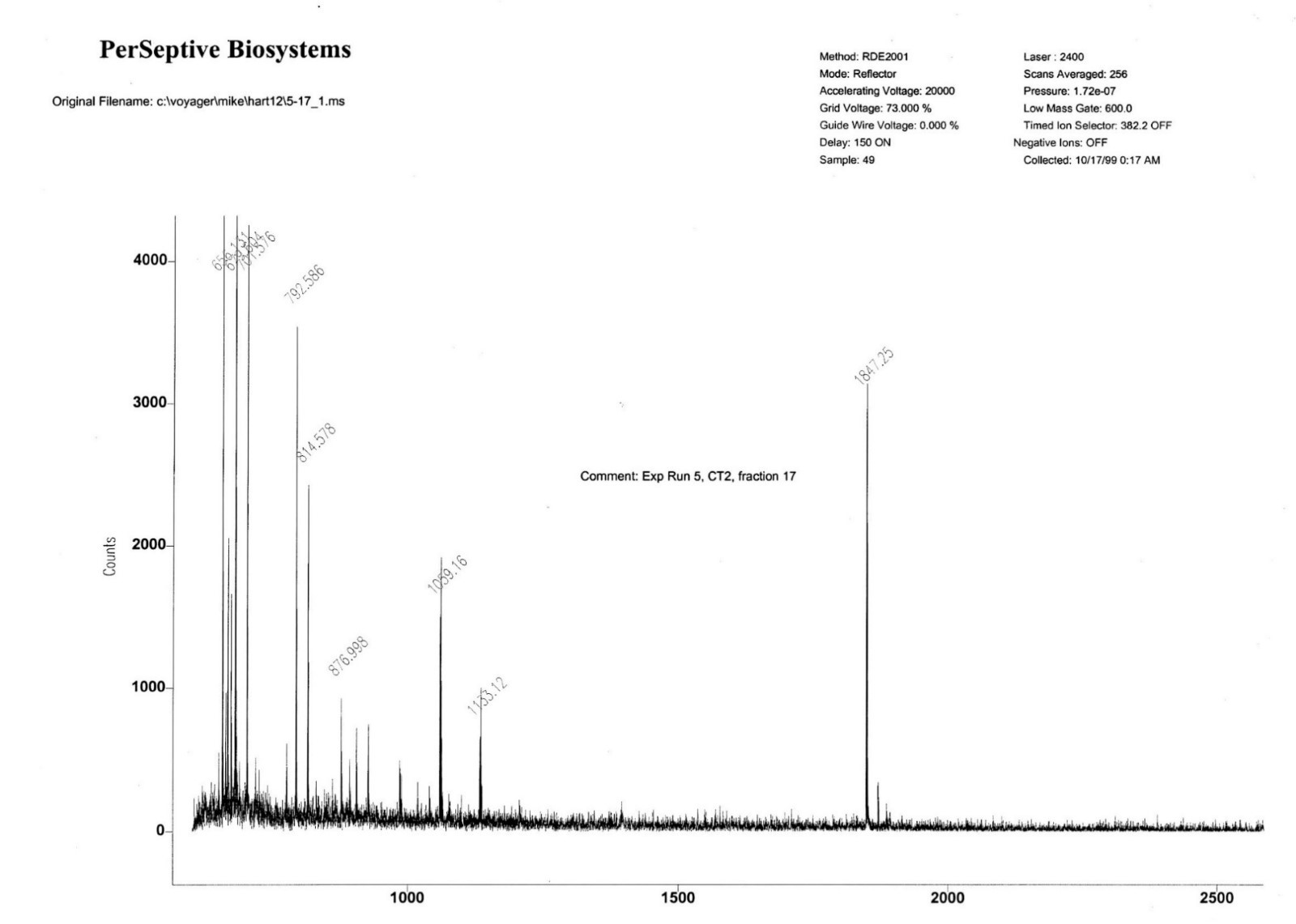


**S3 Figure 14a & b**. MALDI mass spectrum of FPLC anionex Fractions 15 & 17 (of 22) of sheep late luteal jugular vein serum (designated CT2) purified by the Harwell Method (S1). (Horizontal axes are ‘Mass (m/z)’.)

This was an investigation of anionex Fractions 15-22 (of 22) of ovine late luteal systemic blood serum, which drew a blank for late-eluting Candidate 7500. Sundry peaks of lower mass were seen however in the 8 spectra of the CT2 series, where Candidate 7500 was expected.

The dual peaks at 1133 & 1059 in S3 Fig. 14a, a Counts plot, are of interest as ostensibly the same ions as are seen at the top of ramps in the Intensity plots S3 Figs. 13b & d.

The sSgII-70 **10mer** next-integer matches at 1133 & 1059 that are seen on their own in Fraction 15 spectrum **a** appear also in Fraction 17 spectrum **b**. Additionally, there is a prominent peak at 1847.25. Fraction 17 was subject to Edman degradation (Applied Biosystems Procise, using ProSorb PVDF cartridges), yielding a 13 out of 14 residue match to the database sequence of fibrinopeptide A, a degradation product of fibrin (S1’s discounted Candidate VI). No subsidiary sequence was recorded. The molecular weight of ovine fibrinopeptide A is 1848 Da, implying an MH^+^ of 1849. This is in reasonable agreement with the MS peak at 1847. (The candidature of fibrinopeptide A for the sought-for antiorganotrophic factor was discounted when it exhibited contrary positive activity in assays in vivo and in vitro: S1.) These analytical observations relating to fibrinopeptide A confirm the accuracy of both MALDI MS and Edman sequencing at Babraham. (Note that in a starting material spectrum there is a miniscule annotated peak at 1847.9, but this material is otherwise confined to Fraction 17.)

Fraction 17 potential sSgII-70 matches (calculated using Expasy Compute pI/Mw for sub-10mers):

656 (**6mer** MH^+^ without water losses, match 661)

792 (**7mer** MH^+^ without water losses, match 789)

814 (**8mer** MH^+^ at 887 reduced by 4 x 18.015, being four water losses, next-integer match 815)

876 (**9mer** MH^+^ at 986 reduced by 6 x 18.015, being six water losses, match 878)

1059 (**10mer** S3 Table 1 match 1061)

1133 (**10mer** S3 Table 1 integer match 1133)

∑Observed (n = 6) = 5330

∑Matches = 5337

∑Observed/∑Matches x100 = 5330/5337 x100 = **99.87%**

Note that none of the putative **6-10mer** masses above match known values for MS contaminants or matrix cluster ions, though there is one of the latter at 1060 (Keller et al, 2008; see reference in paper).

As noted, the CT2 starting material contained as a minor component fibrinopeptide A. Candidate 7500 was absent from the spectrum, but was there a fragment thereof? There is only one prominent peak, at 2181. The sSgII-70 match for this in S3 Table 1 is a **19mer** N-terminal Grand Fragment at 2178.

[ENDS]
