# Supplementary material for: Detecting the body’s reproductive hormonal brake against tissue overgrowth: Micrin/SgII-70
Source: PLoS One. 2026 Mar 2;21(3):e0340980. doi: 10.1371/journal.pone.0340980 (PMC12952585; doi:10.1371/journal.pone.0340980)
Supplement: S4 File — https://doi.org/10.6084/m9.figshare.27110311.v2. This project attempts to elucidate the identity of Candidate 7500/sSGII-70 using trypsinisation. (DOCX) [file pone.0340980.s004.docx]

**Supplementary Information 4 (S4)**

**Tryptic Digests**

S4 is provided in support of ‘Detecting the body’s reproductive hormonal brake against tissue overgrowth: micrin/SgII-70’ by Hart JE, Davies KG, Mundy CR, Hart AC, Howlett DR & Newton RP (2024). Corresponding author email: [k.davies@herts.ac.uk](mailto:k.davies@herts.ac.uk)

Thirteen trypsinisations of Candidate 7500 material have been attempted using porcine trypsin, with MALDI MS sizing of the resulting fragments. These trypsinisations did not lead to factor identification using the standard tryptic fingerprint databases (see paper, ‘Candidate 7500’), nor is there any hint of to EPL001 (MKPLTGKVKEFNNI), the Edman-derived N-terminal aa sequence relating to Candidate 7500 (see paper, ‘EPL001’, and S4 Table 4 at the foot of this file for the in silico trypsinisation of EPL001). This should not occasion surprise given the co-identification espoused here of Candidate 7500 with sSgII-70, a novel entity predicted to have part of the EPL001 sequence lightly shuffled at its N terminus (MLKTGEKPVFK), with the rest of EPL001 at its C terminus (NNI). sSgII-70 is envisaged to be a reverse-spliced polypeptide of 70 aa. This is accommodated here by the provision of a break-up prediction in silico for a tryptic digest of the conjectured 9+61 reverse spliced sSgII-70 as a linear uncrosslinked polypeptide (Expasy PeptideMass, as per the paper’s references), as detailed in S4 Table 1.

**S4 Table 1.** Tryptic digestion in silico of sSgII-70.

<https://web.expasy.org/peptide_mass/>

**PeptideMass**

The entered sequence is:

10 20 30 40 50 60
MLKTGEKPVF KRTNEMVEEQ YTPQNLATLE SVFQELGKLT GPNNQKHERA DEEQKLYTDD

 70
EDDIYKANNI

The selected enzyme is: Trypsin

Maximum number of missed cleavages (MC): 1

All cysteines in reduced form.

Methionines have been oxidized to form methionine sulfoxide (MSO).

Displaying peptides with a mass bigger than 750 Dalton.

Displaying peptides with a mass lower than 3000 Dalton.

Using average masses of the occurring amino acid residues and giving peptide masses as [M+H]^+^.

**The peptide masses from the sequence are:**

[Theoretical pI: 4.64 / Mw (average mass): 8175.01]

| **mass** | **position** | **#MC** | [**modifications**](https://web.expasy.org/findmod/findmod_masses.html) | **peptide sequence** |
| --- | --- | --- | --- | --- |
| 2091.1458 | 50-66 | 1 | \|  \| \| --- \| | ADEEQKLYTDDEDDIYK |
| 1802.8884 | 56-70 | 1 | \|  \| \| --- \| | LYTDDEDDIYKANNI |
| 1390.4426 | 56-66 | 0 | \|  \| \| --- \| | LYTDDEDDIYK |
| 1294.4123 | 39-49 | 1 | \|  \| \| --- \| | LTGPNNQKHER |
| 1278.5954 | 1-11 | 1 | \| MSO 1294.5948 \| \| --- \| | MLKTGEKPVFK |
| 1142.1700 | 47-55 | 1 | \|  \| \| --- \| | HERADEEQK |
| 1062.2568 | 4-12 | 1 | \|  \| \| --- \| | TGEKPVFKR |
| 906.0693 | 4-11 | 0 | \|  \| \| --- \| | TGEKPVFK |
| 871.9682 | 39-46 | 0 | \|  \| \| --- \| | LTGPNNQK |

62.9% of sequence covered:

10 20 30 40 50 60
MLKTGEKPVF KRtnemveeq ytpqnlatle svfqelgkLT GPNNQKHERA DEEQKLYTDD

 70
EDDIYKANNI

With settings of up to one missed cleavage and fragments of 750-3000 Da, the fingerprint comprises 9 peptide fragments, with an option if M1 is oxidized (‘MSO’, +16). These 10 predictions cover 44 of the 70 residues (1-12 & 39-70, 63% of the total), as the sequence running from T13 to G37 is devoid of basic aa. Three additional predictions in silico each encompass this region in its entirety (including K38), having masses >3000 Da (each having an MSO variant relating to M16). Entities of this magnitude were not seen in any of the MALDI trypsinisations.

The 10 predictions at >750 and <3000 Da were used to interrogate the MALDI-TOF MS raw data output from 13 separate digestions using porcine trypsin (Promega, used according to the manufacturer’s instructions). Eleven of these digestions involved sheep material, with one each additionally for cow and pig (with the in silico tryptic digestions altered as appropriate to reflect SgII sequence variations). Trypsin cleaves peptide bonds C-terminal to K & R residues, if not followed by a proline, P, so SgII-70’s K_7_P_10_ is not a missed cleavage in the predicted *m/z* 1278 item, MLKTGE**KP**VFK. K3 *is* a missed cleavage.

Interfering and contaminant ions encountered in MALDI MS have been documented by Keller et al (2008; see paper’s references). In the Keller list there are more than 650 artefacts, rendering peptide mass fingerprinting, as will be attempted here, a daunting challenge. Among the 10 predicted sSgII-70 tryptic fragments three find integer matches on the Keller list: **871** (CHCA matrix cluster and, separately, trypsin-like peptide artefact); **906** (porcine trypsin autolysis peptide fragment) & **1278** (human keratin). The second and third correspondences are especially awkward as involving fragments usefully predicted to cross the 9/61 reverse peptide splicing boundary.

In the 11 ovine trypsinisation there were a total of 344 recognised MS peaks, representing a peak list of about 31 per spectrum. A total of 278 different ions were discerned, 238 as singletons, 40 as whole-number-repeats in varying degrees (x2 to x7) providing 106 peaks, many of the repeats being matrix clusters, trypsin autolysis products and keratin. A maximum of 25 of the 344 peaks (~7%) are matches at +/– 4 *m/z* to sSgII-70 predicted fragments (S4 Table 2).

**S4 Table 2.** MALDI MS candidate matches to SgII-70 tryptic fragment predictions.

| **sSgII-70**  **Predicted**  **(across)** | **871^B^** | **906** | **1062^C^** | **1142** | **1278** | **1294^D^** | **1390** | **1802** | **2091** |
| --- | --- | --- | --- | --- | --- | --- | --- | --- | --- |
| **Digest No.,**  **Feedstock^A^**  **(down)** |  |  |  |  |  |  |  |  |  |
| 1 OVP |  | 906 |  |  |  |  |  | 1805 |  |
| 2 OFF |  |  |  |  | 1282 |  |  |  |  |
| 3 OFF |  |  | 1059 |  | 1281 |  |  |  |  |
| 4 OFF |  |  | 1059 |  | 1281 |  |  |  |  |
| 5 OFF | 872 | 906 | 1060 |  | 1278 | 1298 |  |  | 2094 |
| 6 OFF |  |  | 1059 |  |  |  |  | 1805 |  |
| 7 OFF |  |  |  |  |  |  |  |  |  |
| 8 OFF  Porcine |  |  |  |  |  |  |  |  |  |
| 9 OFF  Bovine |  |  |  |  |  |  |  |  |  |
| 10 P | 875 | 906 |  |  | 1274 |  |  |  |  |
| 11 OVP | 870 |  |  |  |  | 1296 |  |  |  |
| 12 OFF | 873 |  |  |  |  |  |  |  |  |
| 13 P | 873 | 906 |  |  | 1275 |  |  | 1806 |  |

For the attempted 13 tryptic digests this is the scorecard of matches within *m/z* 4 of SgII-70. Predicted is as in the above table, top row, with *m/z* values reported without fractional masses.

A = Ovine feedstock unless otherwise stated: OVP = ovarian venous plasma; OFF = ovarian follicular fluid; P = plasma, systemic blood.

B = Matches in this column are within the matrix group of ions and are therefore moot.

C = A tryptic fragment of cytokeratin 9 at *m/z* 1060 vitiates the data in this column. No integer 1062 item has been seen, representing (_3_K)TGEKPVFKR(T_13_) at one missed cleavage.

D = Either (_38_K)LTGPNNQKHER(A_50_) or the MSO version of 1278, both one missed cleavage.

A false discovery test involved disregarding the 9+61 reverse spliced sSgII-70 model and adopting instead a 61+9 splicing, without the reversal. This aa sequence was subjected to an in silico tryptic digestion (Expasy PeptideMass). Generated were 8 fragments (plus three MSO forms), 3 not seen in the 9+61 fingerprint in silico as relating to the alternative _59_NNIMLK_64_ junction: 803.9946, 1415.6905 & 2175.4145. There are no integer matches to these 3 unique theoretical fragments anywhere in the ovine MS tryptic data, though there are two loose matches to the 1415 item (>3 *m/z* units away) and one to 2175 (also >3 *m/z* units away). The Keller artefacts list has nothing at 1415 or 2175. The false discovery test teaches that in a large dataset stray inexact matches to predictions will occur, enjoining caution in the main analysis.

Returning to the 9+61 model of sSgII-70, none of the S4 Table 2 matches was validated by de novo sequence data from MS/MS fragmentation, but three of the 13 trypsinisations were in fact allied to tandem MS, to no avail. These investigations all involved ovarian follicular fluid, two ovine purification runs (Digests 11 & 12), one bovine (Digest 9). Digest 11 delivered 7 bacterial hits based on one or two ions each. Four ions in Digest 12 were deemed to derive from keratin and another possibly from androglobin, as described elsewhere (see S1, where androglobin is Candidate IX). The molecular masses recorded in Digest 9 were 13 in all in the range *m/z* 748-2231, all singularities, with 8 garnering ‘suggested sequences’ ranging from 9 to 16 aa residues in length. The database ‘hits’ of the 8 were to bizarre disparate proteins across the taxa, with no highly accurate mass match obtained. As an indication of quality, the matches included ‘Isopropylmalate dehydrogenase from wheat leaf (fungal) pathogen’, 5 units away from the observed mass. The molecular masses deriving from Digests 9, 11 & 12, with keratin matches excluded, were submitted together to a Mascot/NCBIprot search, gaining no significant hits.

The trypsin autolysis product at *m/z* 906, sequence NKPGVYTK, is in fact 906.05 at average isotopic mass (Mascot, confirmed by Expasy Compute pI/Mw, both as referenced in the paper). This compares with the predicted sSgII-70 item at 906.0693.

The integer 906 signal was observed four times in 11 ovine trypsinisations (= 36% prevalence) involving three different feedstocks purified in two different ways (S1 Harwell Method & Babraham Method) analysed by two different MALDI-TOF mass spectrometers. (In MALDI singly charged ions are mainly generated but note that ‘906’ is a mass-to-charge *ratio*, such that multiply charged larger species could theoretically be contributing.) The quadruply available 906 was the most prevalent species bar matrix clusters, trypsin autolysis products and keratin. The *m/z* results were as follows: 906.88 (Digest 1, two places of decimals only), 906.3169 (Digest 5), 906.8600 (Digest 10) & 906.0041 (Digest 13); average = 906.5153. All four 906 items were present at low intensity. This is fitting for an analyte at low abundance which is resistant to proteolysis. The sSgII-70 predicted fragment at *m/z* 906 was present in ovarian venous plasma from intact sheep and absent from jugular vein plasma from ovariectomized controls, as detailed below (Digest 1). This is concordant with the availability and non-availability of Candidate 7500: OV+/OVX–. (See the paper’s Fig. 8 for a visual summary of OV+/OVX– factors). A distribution of OV+/OVX– would be unlikely if the detected 906 item in Digest 1 related to the trypsin autolysis fragment at 906. For the distribution of the latter to align with that of OV+/OVX– Candidate 7500 would be a notable coincidence.

There is strong evidence of trypsin autolysis products in the trypsinisations. Characteristic of such species are items at *m/z* 842 and 2011, both of which are present at high intensity in seven (64%) of the 11 ovine preparations: 842 in Digests 1,3,4,6, 7,11 & 13; and 2011, sometimes as 2012, in Digests 1,3,4,6,7,10 & 13. These high intensity autolysis peaks represent internal mass calibrants. Failure to acquire convincing hits in the peptide mass fingerprinting databases, as described in the paper, was not due to instrument inaccuracy and calibration failures. ‘The accuracy with which peptide masses are measured directly correlates with success in database searching’ (Harris WA, Janecki DJ & Reilly JP, 2002. Use of matrix clusters and trypsin autolysis fragments as mass calibrants in matrix‐assisted laser desorption/ionization time‐of‐flight mass spectrometry. *Rapid communications in mass spectrometry*, **16**, 1714-1722.)

In regard to the four observed 906 items in the 11 ovine trypsinisations the main logical possibilities are that one or more is/are (i) porcine tryptic autolysis fragments; (ii) fragments relating to other sheep proteins; & (iii) predicted sSgII-70 fragments. These possibilities are not mutually exclusive; all might be true or none.

On (ii), in the UniProt database there are 23,108 ovine proteins. Subjecting these to a tryptic digestion in silico at two missed cleavages yields *m/z* 906 fragments from 1194 proteins, representing 5% of total ovine proteins (MacPepDB, <https://macpepdb.mpc.rub.de/>). Two of the 906 trypsinisations (10 & 13) involved SDS-PAGE bands at ~7.5 kDa of ultrafiltered plasma. So, if the relevant 906 species were provided by stray ovine proteins, these are relatively small entities or larger ones behaving anomalously, drastically reducing the 1194 number accordingly. These would be proteins moreover that are undetected by mass spectrometry or Edman degradation. Possibility (ii) is discounted.

Mass spectra are available in the literature sporadically in which an item at 906 is assigned to a particular protein, but the authors have struggled to find any papers where a 906 species is assigned to tryptic autolysis. The Keller artefacts list’s 906 is supported by a reference to unpublished data; the Mascot 906 item would appear to be a theoretical prediction, with 842 and 2211 cited as main peaks and ‘other peaks’ which have been seen at 514, 1045, 2283 & 2298 (= 2283 with oxidised M) (<https://www.matrixscience.com/help/autolysis_help.html>); Promega, the commercial supplier of porcine trypsin, gives the three main trypsin autolysis peptides seen with its product as being at m/z 842, 1045 & 2211; and a paper that alludes to the equivalent product from bovine autolysis, also at *m/z* 906, has this to say: ‘We have found that many bovine trypsin digestions contain only one or two autolysis products with many digests lacking the low mass autolysis products’ (Harris et al, 2002: op. cit.). The observed 36% incidence (4 in 11 digests) of the *m/z* 906 ion here is inconsistent with this last observation: typsin autolysis is ostensibly not at issue.

The mass spectrometry for Digest 1 was conducted by an outside service provider:

M-Scan of Ascot, Berkshire, UK. In the peak list reproduced below as S4 Fig. 6 the mass/charge signal at 906.9 in the OV-intact sample, lacking in the OVX sample, is NOT designated ‘Autodigestion of trypsin’.

A peptide mass fingerprint experiment has been reported from a laboratory involved in the present project (Swansea University, UK). A MALDI-TOF mass spectrum indicated intense tryptic autolysis across the mass size range, without providing a peak at *m/z* 906 (Newton et al, 2004).

The foregoing reflections vitiate possibility (i), that the observed species at *m/z* 906 seen in four digests are due to trypsin autolysis.

The best score of potential matches to sSgII-70 predictions in S4 Table 2 is provided by Digest 5 (described in detail below), which delivers the paper’s Fig. 7. This ‘best score’ correlates with Digest 5 having the best (gold standard) feedstock of the four 906-producing trypsinisations: a maximally purified Candidate 7500 anionex fraction of ovarian follicular fluid. (The second best feedstock is the ovarian venous plasma anionex fraction of Digest 1, yielding ‘OV+/OV– 906’.) The best material delivered the best result. If this correlation is fortuitous, it would represent a coincidence.

The autolysis markers at *m/z* 842 & 2211, prominent in 8 of the 10 other ovine digests, are absentees from Digest 5. Less autolysis is expected at higher analyte concentrations as the number of enzyme-analyte interactions is higher and the number of enzyme-enzyme interactions lower (Harris et al, 2002: op. cit.). Yet two of the other four 906 digests do have both 842 & 2211/2212 items (Digests 1 & 13) and one has 2211 without 842 (Digest 10). There is thus no correlation between presence of 906, at uniform low intensity, and these key markers of autolysis at variable high intensity.

On the balance of probabilities, the proposition that is most likely to be true is this one: ‘the tryptic peptide at *m/z* 906 = a predicted sSgII-70 fragment of that mass-to-charge ratio’.

Summarising thus far: Candidate 7500 is resistant to trypsinisation and when possibly relevant tryptic fragments are suspected they are in low abundance. Such results as are obtained do not find convincing hits in databases of peptide mass fingerprints. Overall, the results of the tryptic digest MS campaign are certainly non-negative for the conjecture that Candidate 7500 is sSgII-70 and are here construed as supportive, particularly given the apparent upholding of the sSgII-70 9+61 reverse splicing conjecture by relevant integer matches to a predicted fragment at *m/z* 906 (also an integer match to 1278 in Digest 5).

Trypsinisation, within an IP/LC-MS protocol, ultimately took the project to ‘likely SgII relatedness’, but not by identifying SgII-70 in the sheep (or other ungulate) but by bringing to light a higher molecular weight splice variant of SgII in the rat (S1).

There follow data accounts of the four digests which provided a species at m/z 906. The results of Digest 5 will be detailed first, as providing the paper’s Fig. 7. Digests 10 & 13 follow, before an extended treatment of Digest 1, which designated the *m/z* 906 item OV+/OVX–. (The feedstock spectrum for unproductive Digest 7 is shown for MS data analytical purposes as S3 Fig. 11.)

**Tryptic Digest 5**

This involved ovine ovarian follicular fluid anion exchange Fraction 28 containing Candidate 7500 (personal communication, Pat Barker, The Babraham Institute, Cambridge, UK). The mass spectrum from this digest is provided as the paper’s Fig. 7. The peak list of tryptic digest peptides (*m/z*) is as follows, with matches emphasized:

840.2923

846.3044

856.2648

862.2901

***872.2498***

878.2679

894.3600

895.1875

900.3714

**906.3169**

910.1434

911.3887

918.4425

925.4656

986.4865

988.4926

1046.8209

1051.3883

1060.5322

1067.3702

1070.5316

1071.4395

1073.3820

1083.3482

1099.0695

1103.6521

1119.7348

1134.5797

1208.5995

**1278.4464**

**1298.0373**

**2094.4897**

The paper’s Fig. 7 is reproduced next for ready reference.

**S4 Figure 1.** Tryptic Digest 5 (the paper’s Fig. 7).


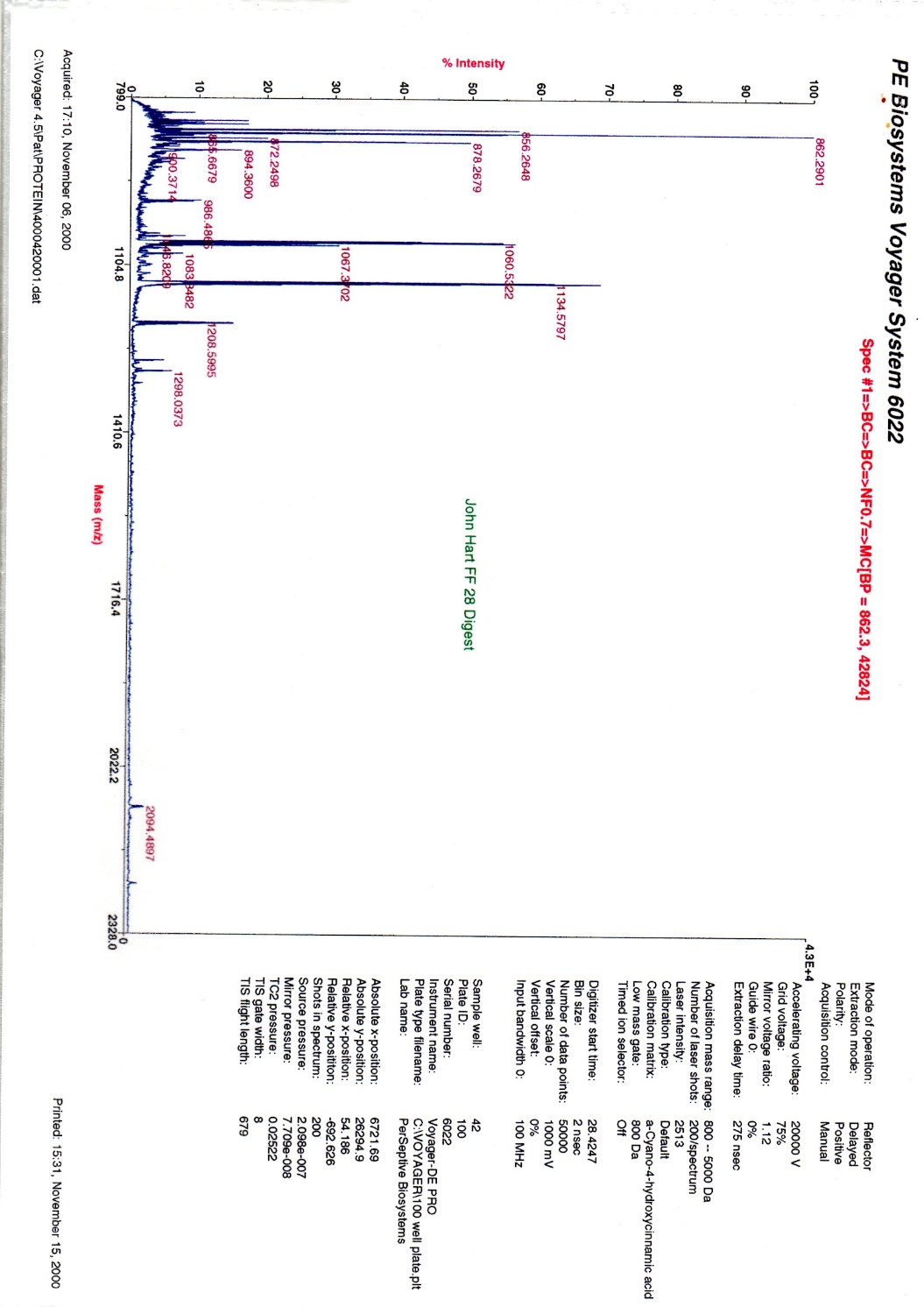


The instrument was in positive ion, reflectron, delayed extraction mode, with heavy lasering (200 shots/spectrum). The mass spectrum of the source material for this digest is shown as S4 Figure 2. Prominent peaks in the *m/z* 800s, including the most prominent peak at 862, relate to the chemical matrix (CHCA) and are presumably due to target peptides in low abundance, necessitating high laser power, with other irrelevant tall peaks (Mascot Contaminants: keratin, e.g. 1060 & 1134). Four minor peak matches (arrowed) to an in silico tryptic digestion of sSgII-70 (S4 Table 1) are in series in terms of intensity. They are as follows: (i) Observed *m/z* **906.3169**, Predicted 906.0693, Difference +0.2476 (0.03% above Predicted), sequence (_3_K)TGEKPVFK(R_12_), no missed cleavage; (ii) Observed **1278.4464**, Predicted 1278.5954, Difference -0.1490 (0.01% below Predicted), _1_MLKTGEKPVFK_11_(R_12_), one missed cleavage; (iii) Observed **1298.0373**, Predicted 1294.4123, Difference +3.6250 (0.28% above Predicted), being either (_38_K)LTGPNNQKHER(A_50_), as the Predicted, or the MSO version of 1278 (at 1294.5948), both one missed cleavage; and (iv) Observed **2094.4897**, Predicted 2091.1458, Difference +3.3439 (0.16% above Predicted), (_49_R)ADEEQKLYTDDEDDIYK(A_67_), one missed cleavage. Items (i) and (ii) relate to the proposed reverse splice 9+61 junction at _9_VF_10_. A fifth match is moot, as being at higher (but still low) intensity and within the matrix ion region: (v) Observed **872.2498**, Predicted 871.9682, Difference +0.2816 (0.03% above Predicted), (_38_K)LTGPNNQK(H_47_), no missed cleavage. The 872 item is rated ‘narrowly non-dismissible’ for the statistical analysis (see below). A contrast is the high intensity 875 item in Digest 10 that is readily dismissible as a match to the predicted sSgII-70 fragment at *m/z* 871.

The match at *m/z* 1278 is problematic, as remarked above. There is a human keratin fragment at this mass on the Keller artefacts list and there are others at 1277. However, the Digest 5 peak is in series with the other potential sSgII-70 matches and is the only integer hit among the six items in the 1278 column of S4 Table 2.

The presence of keratin intense peaks and the absence of autolysis marker peaks at 842 and 2011/2012 are of interest. Keratin may have used up sufficient of the trypsin to reduce trypsin-trypsin autolytic interactions while leaving enough to cleave sSgII-70. An ironically positive upshot, this, of contamination.

In the statistical treatment of Digest 5 in S4 Table 3 ‘Predicted’ is termed ‘Expected’ and ‘d.o.f.’ refers to degrees of freedom. There is a >98% probability that the correlation between Observed and sSgII-70 Expected is not due to chance.

|  | **S4 Table 3. Chi-squared test for Tryptic Digest 5.** | | |  |  |  |  |  |  |  |  |  |  |  |
| --- | --- | --- | --- | --- | --- | --- | --- | --- | --- | --- | --- | --- | --- | --- |
|  |  |  |  |  |  |  |  |  |  |  |  |  |  |  |
|  |  |  |  |  |  |  |  |  |  |  |  |  |  |  |
| O | E | O-E | (O-E)^2^ | (O-E)^2^/E |  |  |  |  |  |  |  |  |  |  |
| 872.2498 | 871.9682 | 0.2816 | 0.07929856 | 9.09E-05 |  | Null hypothesis: There is no correlation between the Observed (O) and Expected (E) data. | | | | | | | |  |
| 906.3169 | 906.0693 | 0.2476 | 0.06130576 | 6.77E-05 |  | The null hypothesis is rejected as there is a correlation between Observed and | | | | | | | |  |
| 1278.4464 | 1278.5954 | -0.149 | 0.022201 | 1.74E-05 |  | Expected data at the >98% significance level. | | | | |  |  |  |  |
| 1298.0373 | 1294.4123 | 3.625 | 13.140625 | 0.010152 |  | Therefore, the probability of these two sets of data correlating is greater than 98%. | | | | | | | |  |
| 2094.4897 | 2091.1458 | 3.3439 | 11.18166721 | 0.005347 |  |  |  |  |  |  |  |  |  |  |
|  |  |  | chi-squared P | 0.015675 |  |  |  |  |  |  |  |  |  |  |
|  |  |  | d.o.f. 4 |  |  |  |  |  |  |  |  |  |  |  |
|  |  |  |  |  |  | **DATA SELECTION** | |  |  |  |  |  |  |  |
|  |  |  |  |  |  | An sSgII-70 tryptic digest in silico, described above, provides the Expected values. Observed peak values are paired off, providing each is within *m/z* 4 of the expected value. (Observed peaks at | | | | | | | | |
|  |  |  |  |  |  | 1062.2568 and 1142 1700 are probably peaks for keratin and so have been omitted.) This method yields five matches. | | | | | | | | |
|  |  |  |  |  |  |  | | | | | | | | |
|  |  |  |  |  |  |  | | | | | | | | |
|  |  |  |  |  |  |  | | | | | | | | |
|  |  |  |  |  |  |  | | | | | | | | |
|  |  |  |  |  |  |  |  |  |  |  |  |  |  |  |


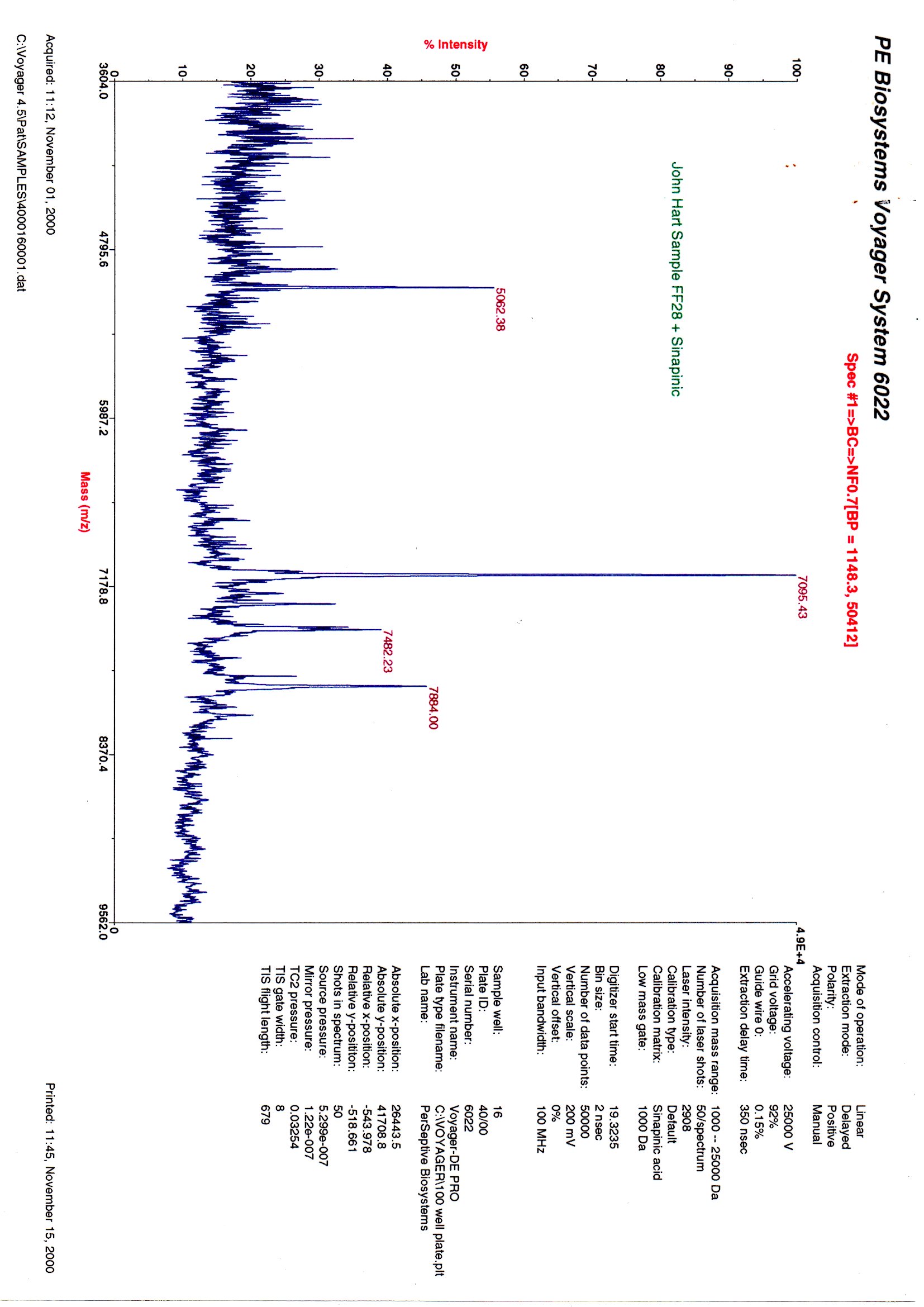


**S4 Figure 2.** MALDI-TOF mass spectrum of ovine ovarian follicular fluid anion exchange Fraction 28, the feedstock for Tryptic Digest 5.

Within the sSgII-70 concept the spectral peaks (*m/z*) in S4 Figure 2 can be interpreted thus, via an MS heterogeneity tabulation elsewhere (S3 Table 1):

7884 = 68mer

7482 = 65mer

7095 = 62mer (main peak)

5062 = 45mer (‘N terminal grand fragment’)

**Tryptic Digest 10**

This involved ovine blood plasma. The source material was the upstream precipitate of 3-30 kDa ultrafiltered sheep jugular vein plasma (S1, Sheffield Method), which was run out on a 10-20% tricine gradient gel (personal communication, Pat Barker, The Babraham Institute, Cambridge, UK). The lowest MW band discernible, at ~7.5 kDa, was excised and subjected to digestion. A MALDI spectrum of this material prior to digestion is shown in the paper’s Fig. 5, which includes the 7930.24 ion, analysed to be a 68mer within the sSgII-70 concept (S3). It was from this material that the EPL001 sequence was derived.


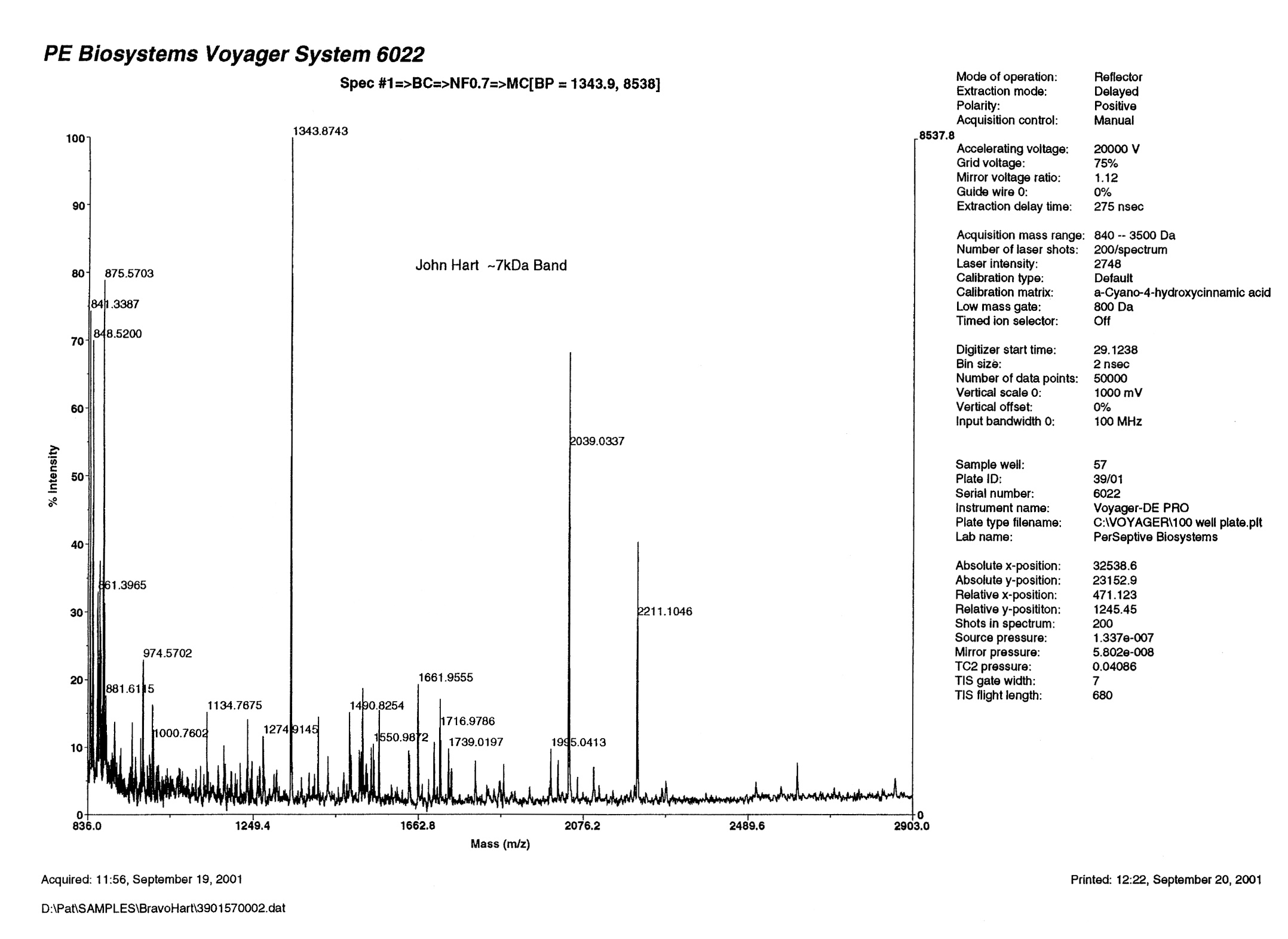


**S4 Figure 3.** Tryptic Digest 10.

The peak list for Digest 10 is as follows, with sSgII-70 matches emphasized:

841.3387

848.5200

861.3965

**875.5703**

881.6115

**906.8600** (mass spectrum peak unannotated)

974.5702

1000.7602

1134.7675

**1274.9145**

1343.8743

1490.8254

1550.9872

1661.9555

1716.9786

1739.0197

1995.0413

2039.0337

2211.1046

The spectrum features prominent peaks in the 800s representing the CHCA matrix, a trypsin autolysis product (2211) and a tall pair of suspected rogues (Mascot Contaminants: collagen, 1343 & 2039). The **906** item, (_3_K)TGEKPVFK(R_12_), is seemingly present once more (Difference +0.7907, 0.0873% above Predicted), as possibly is the **1278** item, _1_MLKTGEKPVFK(R_12_), as 1274.9145, which is 3.6809 below Predicted (-0.2879%). These two peaks are at the same intensity, the 875 loose match to an ~872 predicted fragment being dismissably intense as a matrix ion.

**Tryptic Digest 13**

The 906 analysis for Tryptic Digest 13 is Observed **906.0041**, Predicted 906.0693, Difference -0.0652 (0.007% below Predicted), (_3_K)TGEKPVFK(R_12_), no missed cleavage. The 1802 item may also be present in a crowded spectrum: Observed **1806.8485**, Predicted 1802.8884, Difference +3.96 (0.220% above Predicted), (_55_K)LYTDDEDDIYKANNI_70_, one missed cleavage. (The feedstock here was ovine blood plasma ‘upstream precipitate’, as detailed in S1, Sheffield Method.)

**S4 Figure 4.** Tryptic Digest 13.

**Tryptic Digest 1**

This digest is of interest as involving material from ovary-intact and ovariectomised (OVX) ewes, with a 906 item present in ovarian venous plasma from the former but not in jugular vein plasma from the latter (personal communication, Clare Collier, Harwell Laboratory, South Oxfordshire, UK). This is in line with MS Candidate 7500 being OV+/OVX–, supporting the identification of the 906 species as a tryptic fragment of sSgII-70 and the co-identification of Candidate 7500 with sSgII-70.

Tryptic Digest 1 will now be described in detail. The work was conducted at Harwell Laboratory, South Oxfordshire, UK, and involved a flock of sheep maintained there for experimental purposes and an animal house for which female Sprague Dawley rats were obtained from an outside supplier (Charles River, Margate, Kent, UK). The research took place when Candidate 7500 was recognized as a candidate molecule for the sought-for tissue-inhibitory factor but before it was declared Lead Candidate. At this stage the focus was on 10-20 kDa fractions, following hints from prior purification and bioassay experience at the Babraham Institute, Cambridge, UK. Candidate 7500 partitions around a 10 kDa cut-off, it can be noted.

Ovarian vein blood was obtained from anaesthetised sheep on approximately Day 8 of the oestrus cycle, when Candidate 7500 is high (see ‘Candidate 7500’ in the paper). Blood was collected from both ovaries simultaneously via cannulae. ‘Active’ sheep were selected on the basis of the organ mass reducing activity of raw plasma in a rat organometric assay in vivo (Hart, 1990b). Blood from ovariectomised sheep was obtained from the jugular vein 3-5 weeks post-ovariectomy. Heparin was used as an anticoagulant, with plasma harvested after centrifugation.

Purification was according to the Harwell Method, which involves gel filtration followed by anion exchange chromatography, with the usual prior spin (membrane) filtration steps of the Babraham Method (S1: Purification Methods) omitted, in an attempt to reduce losses. The procedure was as follows: 30 ml plasma pool; apply plasma to size calibrated Superdex 200 column (mobile phase 50 mM Tris/HCl pH 7.5); monitor eluate at 280 nm to discount breakthrough early-eluting plasma proteins; after absorbance dip, collect fractions covering MW range 10-20 kDa (volume approx. 20 ml); load 20 ml on Mono Q anion exchange FPLC and elute with salt gradient; dilute fractions underlying absorbance peaks (214 and 280 nm) with PBS; load pools of anion exchange (‘anionex’) fractions at maximum allowable volume on SDS PAGE; gels stained with GelCode colloidal Coomassie blue system; region of gel between 10 and 20 kDa excised (located by reference to MW markers and a sole visualisable band, at ~18 kDa, present in ovary-intact and OVX material, probably Candidate II in S1, ‘Haptoglobin-like protein’), stored in 10% methanol water and sent for mass spectrometric analysis at an outside supplier (M-Scan, Ascot, Berkshire, UK). A blank area of gel was excised as a control.

At the external laboratory the samples were subject to in-gel tryptic digestion. The resulting peptides were extracted from the NuPage gel material and purified using a C_18_ cartridge (30% acetonitrile = ‘AcCN’ in spectra). The peptide containing fraction was collected and a portion (1µl) analysed by Delayed Extraction-Matrix Assisted Laser Desorption Ionisation – Time of Flight Mass Spectrometry (DE-MALDI-TOF-MS).

Adjusting for controls and contaminants, no major *m/z* signals were seen. The tabulated records for the total number of peaks registered was as follows: gel blank, 42; OVX (pool of ‘post-dip’ anionex fractions collected after an initial elution phase of high UV absorbance at 280 nm), 29; ovary-intact early eluting anionex fractions (9-11), 36; and ovary-intact late eluting anionex fractions (17-19), 32. Peaks were noted consistent with autodigestion products of porcine trypsin and commonly observed keratin contaminants. When these and the gel blank signals are subtracted from OVX, these signals remain (tabulated at one decimal place): 861.5, 1007.0, 1852.8, 2196.6 & 2363.3. No database identification was made on the basis of these signals and none matched sSgII-70 predictions.

Next, considering ovary-intact early eluting anionex material, after subtracting autolysis products, keratin, gel blank matches and OVX matches, there were five signals remaining: 928.8, 1208.7, 1494.2, 1658.3 & 2255.9. No database identification was made on the basis of these signals and none matched sSgII-70 predictions.

Finally, considering ovary-intact late eluting anionex material, after subtracting autolysis products, keratin, gel blank matches, OVX matches and a single match (1658.3) from ovary intact early eluting anionex material, there remain four unique items: 906.9, 1639.6, 1805.7 & 1835.7. No database identification was made on the basis of these signals.

The conclusion of the ovary-intact/OVX MS report from M-Scan reads as follows: ‘No major signals were observed with DE-MALDI-TOF-MS data obtained from samples [designation] which were not present in the data obtained from the NuPage Gel blank. This suggests that either levels of protein below the limit of detection were present in samples [designation] (autodigestion products of porcine trypsin were detected – 22pmoles of porcine trypsin used for digestion) or the proteins present in samples [designation] are not susceptible to digestion with trypsin.’

Referring again to ovary-intact late eluting anionex material, of the four signals – 906.9, 1639.6, 1805.7 & 1835.7 – the second and fourth items (1639.6 & 1835.7) do not conform with sSgII-70 predicted fragments. In the 11 ovine trypsinisations 1639 appears as an integer three times and is an integer match for zinc finger contaminant. 1835 is unique to this trypsinisation and does not correspond to a commonly recognised contaminant on the Keller artefacts list. The other two ions do provide matches to sSgII-70 predicted fragments (using the unrounded figures from the mass spectrum on the next page): Observed **906.88**, Predicted 906.0693, Difference +0.8107 (0.089% above Predicted), (_3_K)TGEKPVFK(R_12_), no missed cleavage; and Observed **1805.73**, Predicted 1802.8884, Difference +2.841 (0.158% above Predicted), (_55_K)LYTDDEDDIYKANNI_70_, one missed cleavage. The 906 integer match in particular, with no missed cleavages, seems to afford a glimpse of late-eluting anionex Candidate 7500 in ovarian venous plasma, absent from the OVX jugular vein plasma.


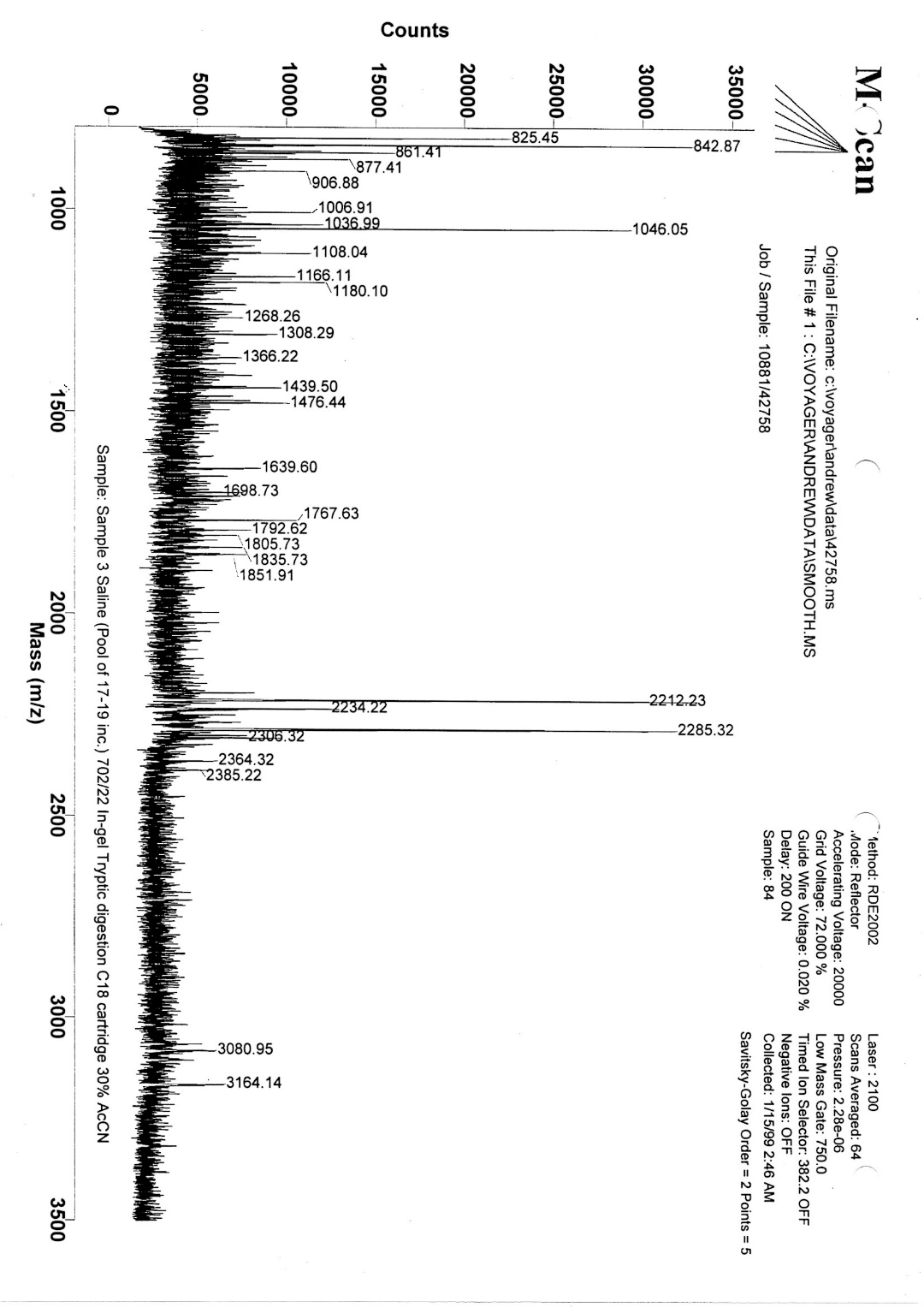


**S4 Figure 5. Digest 1:** DE-MALDI-TOF MS of ovine ovary-intact late eluting anionex fraction. Peaks at 906.88 and 1805.73.


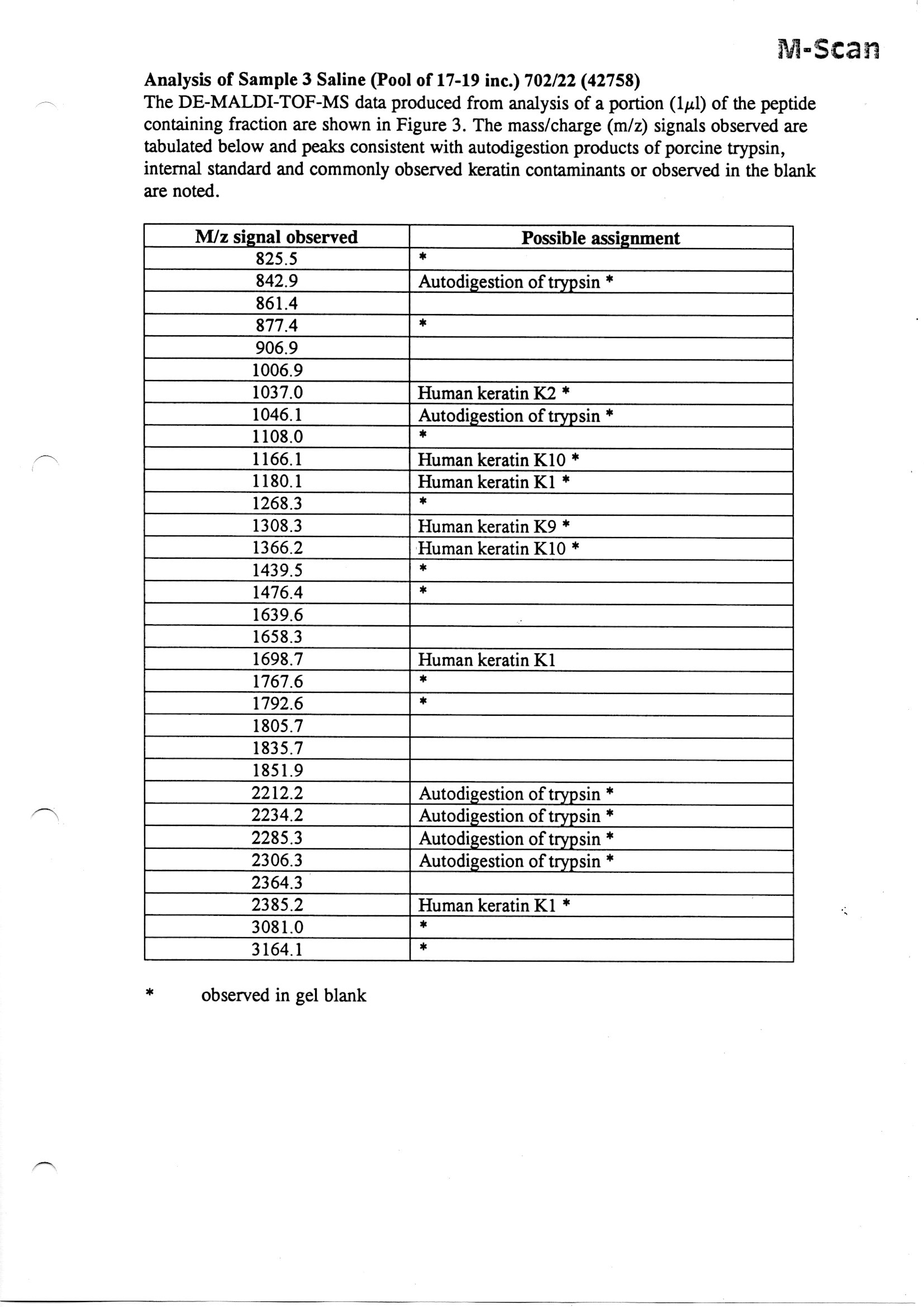


**S4 Figure 6.** Digest 1 OV+ peak list. Note that the mass/charge signal at 906.9 is NOT denoted ‘Autodigestion of trypsin’.


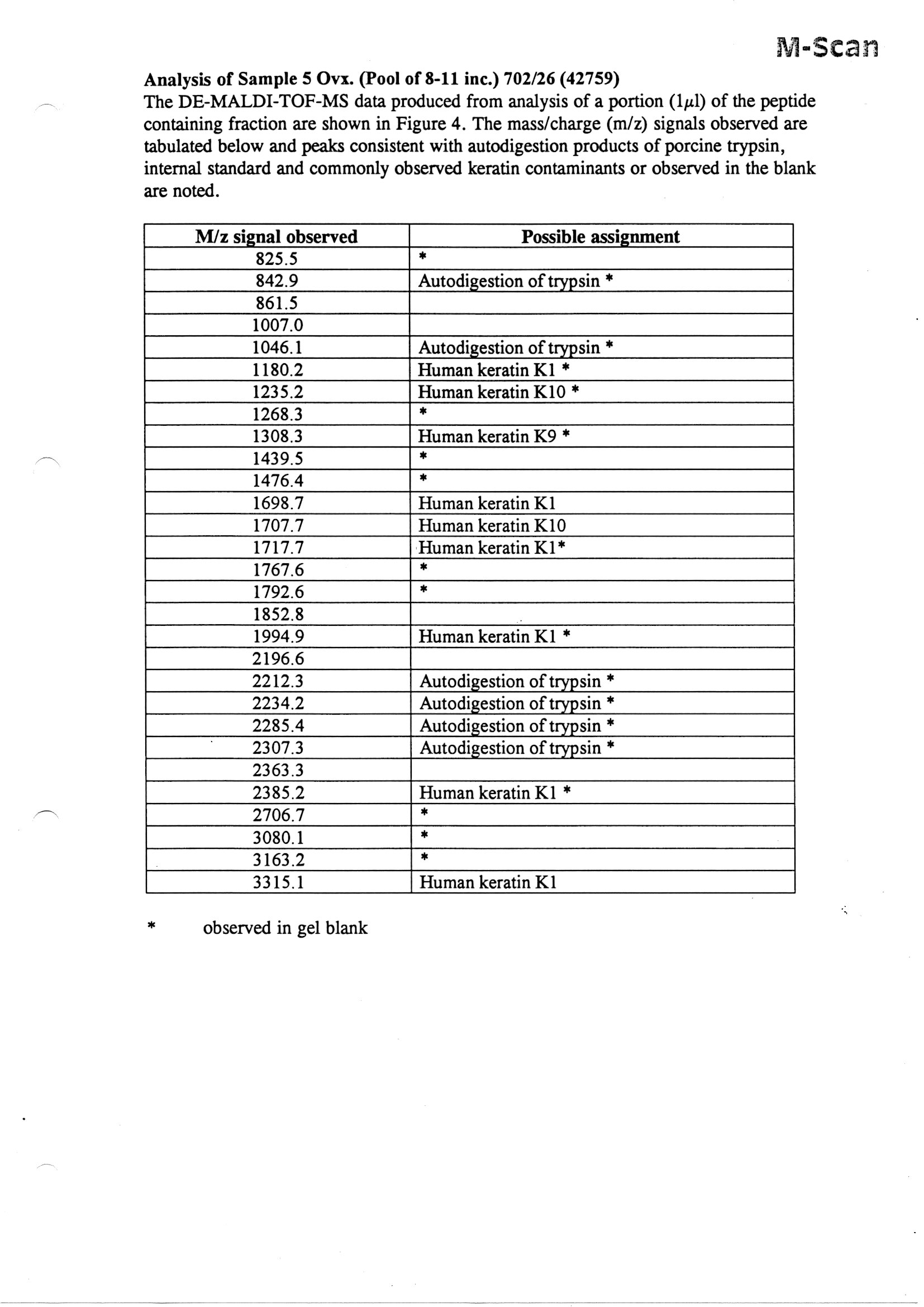


**S4 Figure 7.** Digest 1 OVX peak list. The mass/charge signal at 906, seen in OV+ material, is lacking

**S4 Table 4.** Tryptic digestion in silico of EPL001.

<https://web.expasy.org/peptide_mass/>

**PeptideMass**

The entered sequence is:

10
MKPLTGKVKE FNNI

The selected enzyme is: Trypsin

Maximum number of missed cleavages (MC): 1

All cysteines in reduced form.

Methionines have been oxidized to form methionine sulfoxide (MSO).

Using average masses of the occurring amino acid residues and giving peptide masses as [M+H]^+^.

**The peptide masses from your sequence are:**

[Theoretical pI: 9.70 / Mw (average mass): 1618.95]

| **mass** | **position** | **#MC** | [**artif.modification(s)**](https://web.expasy.org/findmod/findmod_masses.html#Others) | [**modifications**](https://web.expasy.org/findmod/findmod_masses.html) | **peptide sequence** |
| --- | --- | --- | --- | --- | --- |
| 1002.3033 | 1-9 | 1 | \| MSO: 1 \| 1018.3027 \| \| --- \| --- \| | \|  \| \| --- \| | MKPLTGKVK |
| 863.9885 | 8-14 | 1 | \|  \| \| --- \| | \|  \| \| --- \| | VKEFNNI |
| 774.9966 | 1-7 | 0 | \| MSO: 1 \| 790.9960 \| \| --- \| --- \| | \|  \| \| --- \| | MKPLTGK |
| 636.6818 | 10-14 | 0 | \|  \| \| --- \| | \|  \| \| --- \| | EFNNI |
| 246.3294 | 8-9 | 0 | \|  \| \| --- \| | \|  \| \| --- \| | VK |

100.0% of sequence covered:

10
MKPLTGKVKE FNNI

Evidence relating to the 14-residue Edman-derived ovine sequence EPL001 (S1 Table 1) is absent from the tryptic digest campaign. Eleven of the thirteen tryptic digests carried out involved purified feedstock that was relevantly ovine. A digest with porcine trypsin in silico (Expasy PeptideMass) of EPL001 (MKPLTGKVKEFNNI), at up to one missed cleavage, yields seven predicted fragments (S4 Table 4). In the MALDI ovine dataset there are no integer matches to any of these and there are no next-integer hits either, except to a predicted fragment at 863 Da (VKEFNNI), in the form of (overly prominent) peaks at *m/z* 861/2 in nine of the eleven trypsinisations, being a known CHCA matrix cluster. (This item is also present in OVX material lacking Candidate 7500: Digest 1.) Notable is an absence of integer or next-integer matches to the one predicted EPL001 fragment comfortably outside the matrix region and which does not match the mass of any known contaminant: MKPLTGKVK (1002 Da at one missed cleavage, with the MSO variant at 1018). The absence of EPL001-associated tryptic digest predicted fragments is consistent with the view argued in the paper that EPL001 does not represent a true-to-life N-terminal sequence, explaining bioinformatic and molecular biological unproductivity.

[ENDS]
