# Supplementary material for: Detecting the body’s reproductive hormonal brake against tissue overgrowth: Micrin/SgII-70
Source: PLoS One. 2026 Mar 2;21(3):e0340980. doi: 10.1371/journal.pone.0340980 (PMC12952585; doi:10.1371/journal.pone.0340980)
Supplement: S5 File — https://doi.org/10.6084/m9.figshare.27110320.v2. This project presents in silico molecular modelling of sSgII-70 directed at a conformational understanding of the molecule. (DOCX) [file pone.0340980.s005.docx]

**Supplementary Information 5 (S5)**

**Molecular Modelling**

S5 is provided in support of ‘Detecting the body’s reproductive hormonal brake against tissue overgrowth: micrin/SgII-70’ by Hart JE, Davies KG, Mundy CR, Hart AC, Howlett DR & Newton RP (2024). Corresponding author email: [k.davies@herts.ac.uk](mailto:k.davies@herts.ac.uk)

SgII-70 is a proposed hormonal proteoform of mammalian secretogranin II, of fully defined aa sequence. Here is how sSgII-70, the sheep version of the hormone, models in silico as a linear polypeptide without covalent crosslinks. (The ChemSW software used to generate this image is described under ‘Methodology’ on page 11, prior to a description of the use of AlphaFold2.)


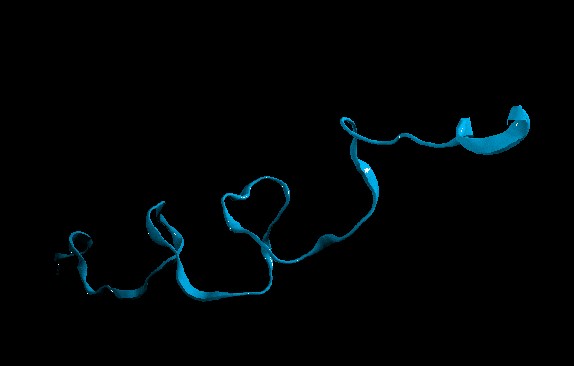


**S5 Figure 1.** sSgII-70 without covalent crosslinks, Bezier of native state derived by energy minimization.


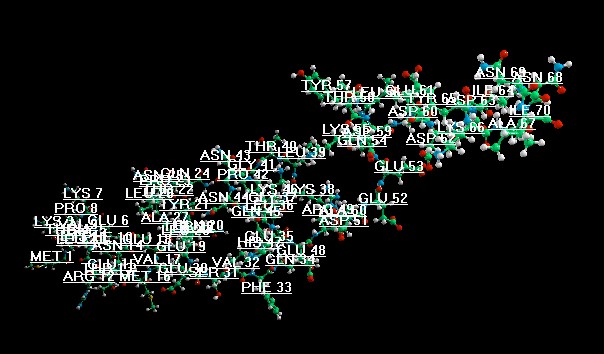


**S5 Figure 2.** sSgII-70 without covalent crosslinks, native state derived by energy minimization.

The no-crosslinks model fails to accommodate one of the paper’s two structural predictions for sSgII-70, juxtaposed ends, while coming closer on the other, spiralisation, judging by the Bezier ribbon of S5 Fig. 1.

On juxtaposed ends, the deduction is made in the paper of adjacent N and C termini. This is on the basis of the evidence from epitope mapping, an active face deduction and successful mimesis thereof with hexapeptides, magic gridding (the paper’s Fig. 10) and Edman sequencing analysis. Molecular spiralisation is also deduced, on the basis of the analysis of Edman sequencing results and reading paths, magic gridding, a playing-card riffle shuffle analysis (see the paper’s ‘Likely SgII related’) and a multi-hundred stepped grand fragmentation pattern seen in MALDI. Juxtaposed termini and spiralisation, both described in the paper’s ‘Summation’, imply that the 70mer polypeptide is in the molecular form of a twisted U. No such shape is obtainable from molecular modelling in silico of the linear 70mer, as we have just seen, and also not predicting a twisted U from linear sSgII-70 is AlphaFold2 (see below, where there is more on S5 Fig. 1 after S5 Fig. 10).

Accordingly, the attempt was made to derive the twisted U shape of expectation by inserting into the model hypothetical covalent crosslinks. As will be reported shortly, this effort failed to provide a polypeptide having the predicted structural properties and it is anyway concluded in the paper on the basis of modelling-unrelated considerations (e.g. tryptic digest results) that there are no covalent crosslinks within SgII-70. The paper suggests instead that molecular stabilisation is achieved via salt bridging involving the two ends of the polypeptide, bringing about electrostatic entwinement.

At an early stage of the analysis, then, a molecular hairpin was posited speculatively stabilised by intramolecular isopeptide crosslinks between the side chains of glutamine and lysine residues (Hart, 2021; see paper for reference). (There are no cysteine residues in sSgII-70 to provide disulphide bonds.) But exhaustive molecular modelling in silico of QK transamidation bonds has failed to deliver an image of sSgII-70 with juxtaposed termini. The present supplementary file is partially a record of this clarifying failure.

If QK crosslinks exist within sSgII-70, where might they be situated? In other words, the side chains of which aa are involved in the proposed crosslinks? The MS data analysis in the main paper assumes no QK crosslinks, but a prior analysis involved the assumption of two such bonds (Hart, 2021; op. cit.). From a detailed extension of the MS data to include sSgII-70 Grand Fragments, it seemed likely that if there were two QK bonds then they would most likely involve glutamines Q20 & Q24. The potential partner lysines are as follows: K3, K7, K11, K38, K46, K55 & K66.

To find the number of different potential combinations the general formula is:

^Q^C_B_ x ^K^C_B_ x B!

where

C = Combinations

Q = Glutamines

K = Lysines

B = Bonds

For 2Qs (Q20 & Q24), 7Ks and 2Bs:

[2!/2! x 0!) x 7!(2! x 5!)] x 2! = 1 x 21 x 2 = 42.

To facilitate molecular modelling the QK x2 possibilities were represented thus:

**01**, Q20-K3, Q24-K7; **02**, Q20-K3, Q24-K11; **03**, Q20-K3, Q24-K38;

**04**, Q20-K3, Q24-K46; **05**, Q20-K3, Q24-K55; **06**, Q20-K3, Q24-K66;

**07**, Q20-K7, Q24-K3; **08**, Q20-K7, Q24-K11; **09**, Q20-K7, Q24-K38;

**10**, Q20-K7, Q24-K3; **11**, Q20-K7, Q24-K11; **12**, Q20-K7, Q24-K66;

**13**, Q20-K11, Q24-K3; **14**, Q20-K11, Q24-K7; **15**, Q20-K11, Q24-K38;

**16**, Q20-K11, Q24-K46; **17**, Q20-K11, Q24-K55; **18**, Q20-K11, Q24-K66;

**19**, Q20-K38, Q24-K3; **20**, Q20-K38, Q24-K7; **21**, Q20-K38, Q24-K11;

**22**, Q20-K38, Q24-K46; **23**, Q20-K38, Q24-K55; **24**, Q20-K38, Q24-K66;

**25**, Q20-K46, Q24-K3; **26**, Q20-K46, Q24-K7; **27**, Q20-K46, Q24-K11;

**28**, Q20-K46, Q24-K38; **29**, Q20-K-46, Q24-K55; **30**, Q20-K46, Q24-K66;

**31**, Q20-K55, Q24-K3; **32**, Q20-K55, Q24-K7; **33**, Q20-K55, Q24-K11;

**34**, Q20-K55, Q24-K38; **35**, Q20-K55, Q24-K46; **36**, Q20-K55, Q24-K66;

**37**, Q20-K66, Q24-K3; **38**, Q20-K66, Q24-K7; **39**, Q20-K66, Q24-K11;

**40**, Q20-K66, Q24-K38; **41**, Q20-K66, Q24-K46; **42**, Q20-K66, Q24-K55.

QKs Models 06 & 37 were expected to deliver a hairpin structure for sSgII-70, as involving lysines at the extreme ends of the polypeptide: K3 & K66. But they did not, as will now be illustrated.


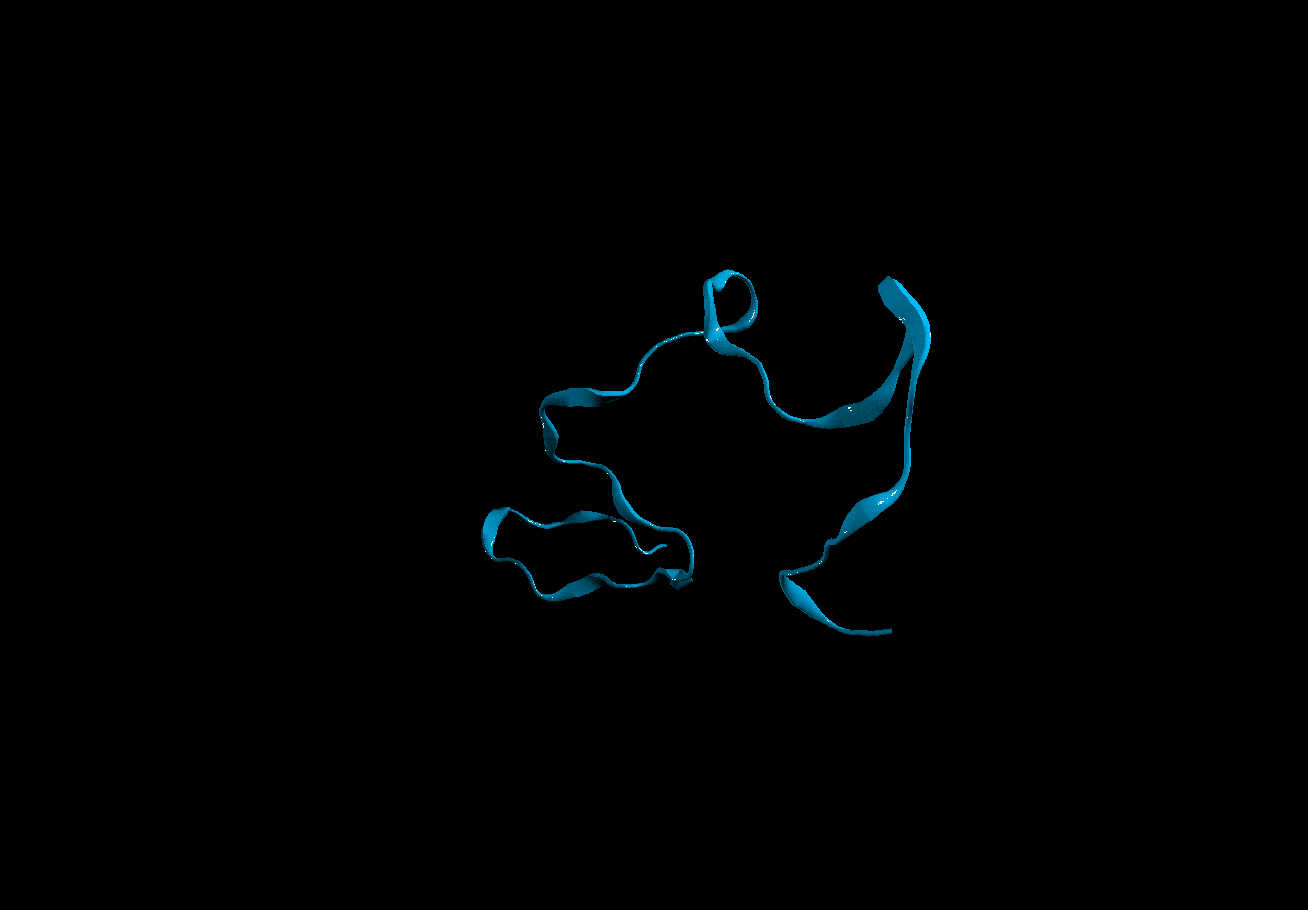


**S5 Figure 3.** sSgII-70 QKs Model 06, crosslinked (Q20-K3/Q24-K66), Bezier of native state derived by energy minimization.


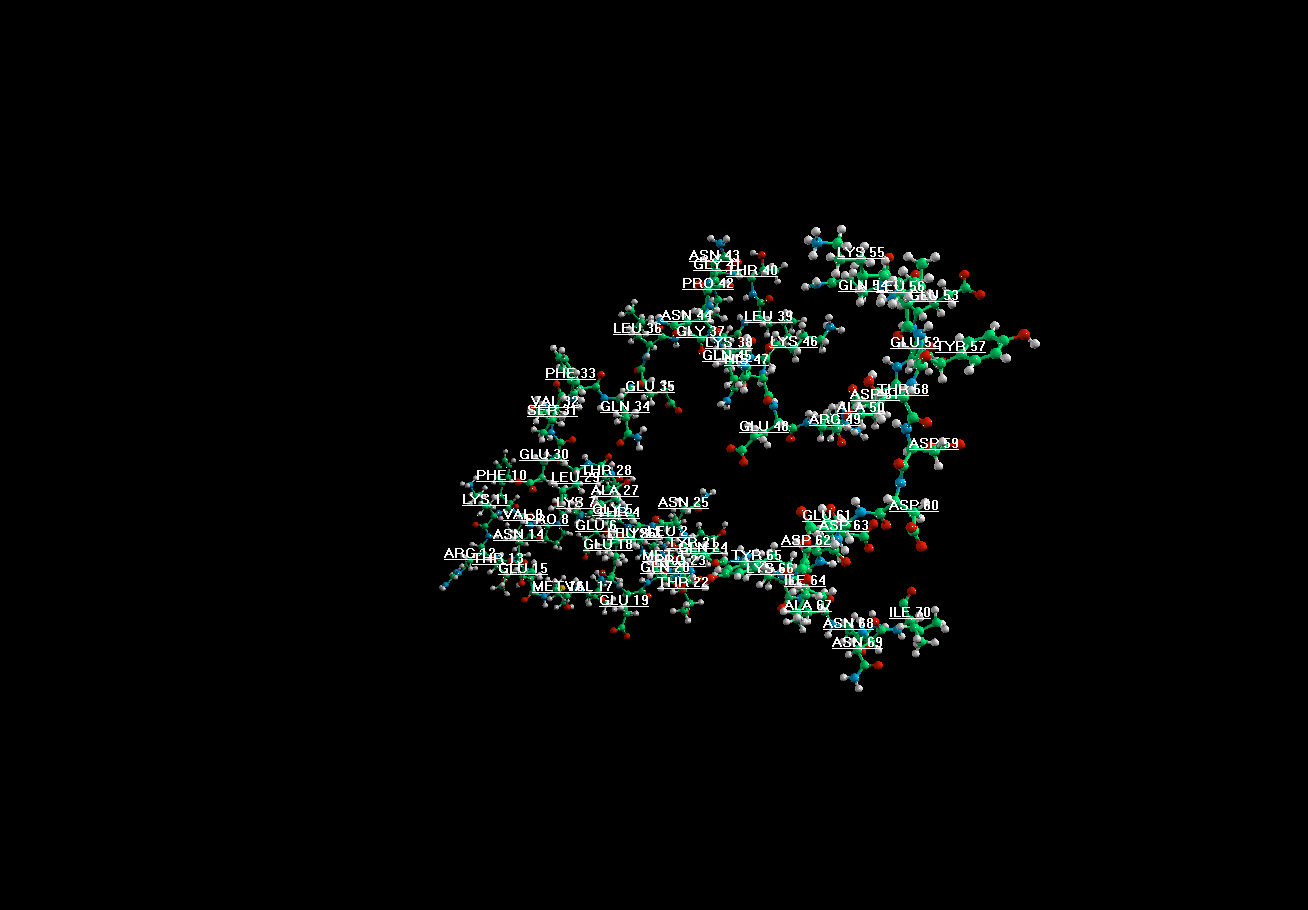


**S5 Figure 4.** sSgII-70 QKs Model 06, crosslinked (Q20-K3/Q24-K66), native state derived by energy minimization.


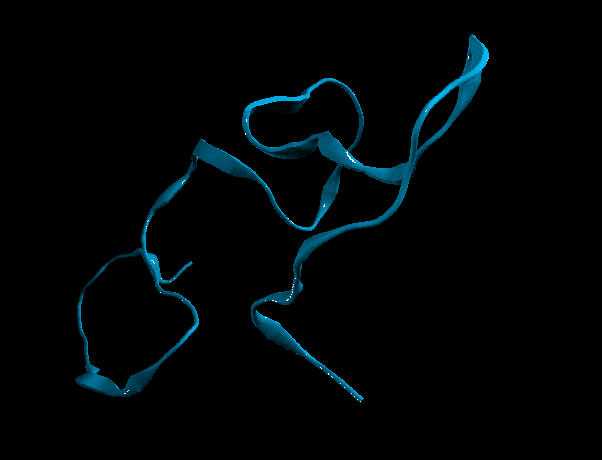


**S5 Figure 5.** sSgII-70 QKs Model 37, crosslinked (Q20-K66/Q24-K3), Bezier of native state derived by energy minimization.


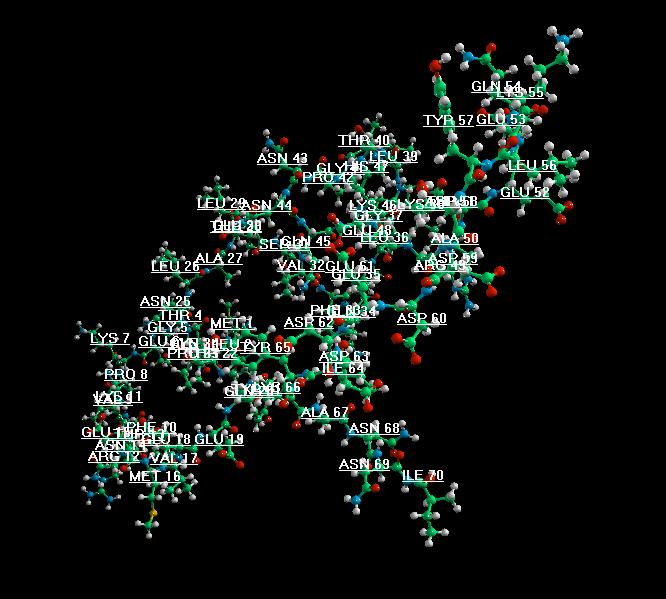


**S5 Figure 6.** sSgII-70 QKs Model 37, crosslinked (Q20-K66/Q24-K3), native state derived by energy minimization.

The existence of tryptic digest fragment integer matches at *m/z* 906 (_4_TGEKPVFK_11_) and 1278 (_1_MLKTGEKPVFK_11_) implies that sSgII-70’s three N-terminal lysines (K3, K7 & K11) are not involved in crosslinking, although these residues were included in the QK x2 analysis for completeness. The only remaining N-terminal basic partner is R12, which did not turn up as a tryptic digest fragment integer match at *m/z* 1062 (_4_TGEKPVFKR_12_) and which eluded seven Edman degradations, as might be expected in both cases from a residue constrained by crosslinking. K66 was used to place a second crosslink close to the C terminus of the 70mer.


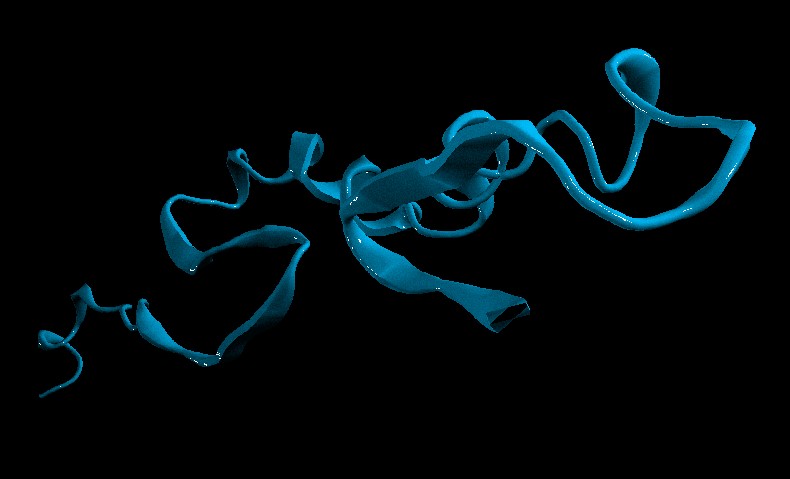


**S5 Figure 7.** sSgII-70 QR/QK Model, crosslinked (Q20-K66/Q24-R12), Bezier of native state derived by energy minimization.


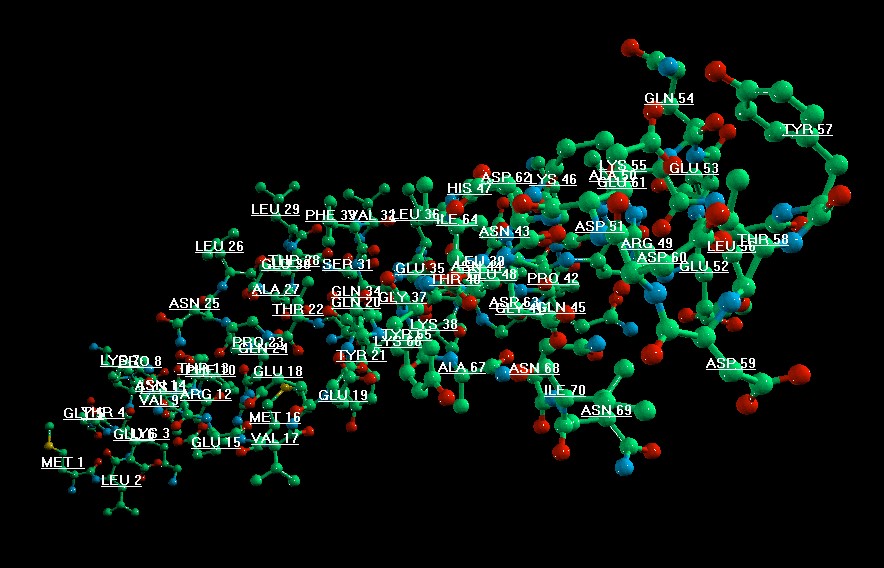


**S5 Figure 8.** sSgII-70 QR/QK Model, crosslinked (Q20-K66/Q24-R12), native state derived by energy minimization (without hydrogens).

Instead of producing a molecular hairpin, the QR/QK models, like the QK/QK models before them, produced a ‘squashed shuttlecock’ with termini far apart. Multiple crosslinking (i.e. >2) with different aa combinations likewise produced a squashed shuttlecock conformation. The termini in the models are splayed too far apart to be just repelling each other, as basic N terminus and acidic C terminus, therefore there is something intrinsic to the sequence in respect of primary, secondary and tertiary structure to produce the effect. At the N terminus proline (P8) is likely to be responsible for the kink. The orientation of the other end of the molecule is more difficult to explain, though there may be involvement in the splaying effect of polyanionic motifs towards the C terminus: _48_ExxDEExxxxxDDEDD_63_.

A key consideration as thinking evolved was that the paper’s MALDI MS analysis implies anomalous fragmentation of the target molecule. It is anomalous in terms of unexpectedness – for a soft MS technique based on the protonation of whole masses. But the view taken here is that SgII-70 loses C-terminal parts of itself plus water (‘dual decrementation’) during processing and storage and due to MALDI ‘plate tectonics’ (choice of matrix and immediate or delayed lasering), rather than as a result of MS post-source decay. The MS dual decrementation data model is consistent in the sense of yielding only N-terminal fragments of linear SgII-70. Covalent crosslinking would be expected to provide fragments discordant with a linear interpretation. None such has been seen.

Why has the deduced twisted U shape of sSgII-70 not popped out of any of the models? The paper posits that post-translational modification accounts for the shape of the molecule, involving reverse peptide splicing from one molecule of SgII or more probably joining of 9mer and 61mer modules from two different molecules of SgII. Featured is a polyacidic D/E tail looped back to a basic N terminus, potentially bringing about electrostatic self-entanglement. ‘Cliff edge lability’ is described in the paper for exported sSgII-70.

Away from the termini, what of spiralisation of the rest of the molecule? Physical modelling involved manual construction of a paper tape representation, with the 70 aa marked along the tape in the single-letter code. This simple model suggested a sole twist is likely with two twists a lesser possibility, even three, meaning the molecule probably resembles a minimally braided (single twist) molecular ampersand:

**&**

**Methodology**

Models in silico were developed using Molecular Modelling Pro Plus, version 6.22, and ChemSite, version 5.10, produced by ChemSW (Accelrys Inc., San Diego, USA). Models of polypeptides were constructed by sequential addition of amino acid residues. Each model was adjusted in conformation to minimize energy levels: energy minimization was carried out in 1-fs time steps, to a total of 10,000 fs, with 100 equilibrium steps per iteration. Iterations were continued until six repeat iterations yielded no change in energy gradient.

The ChemSW software is based primarily on physico-chemical mathematical prediction while AlphaFold2, to be described next, is based on precedents from a database.

**AlphaFold2**

AlphaFold2 is an artificial intelligence program incorporating deep learning, which predicts protein structures from amino acid sequences based on database precedents.


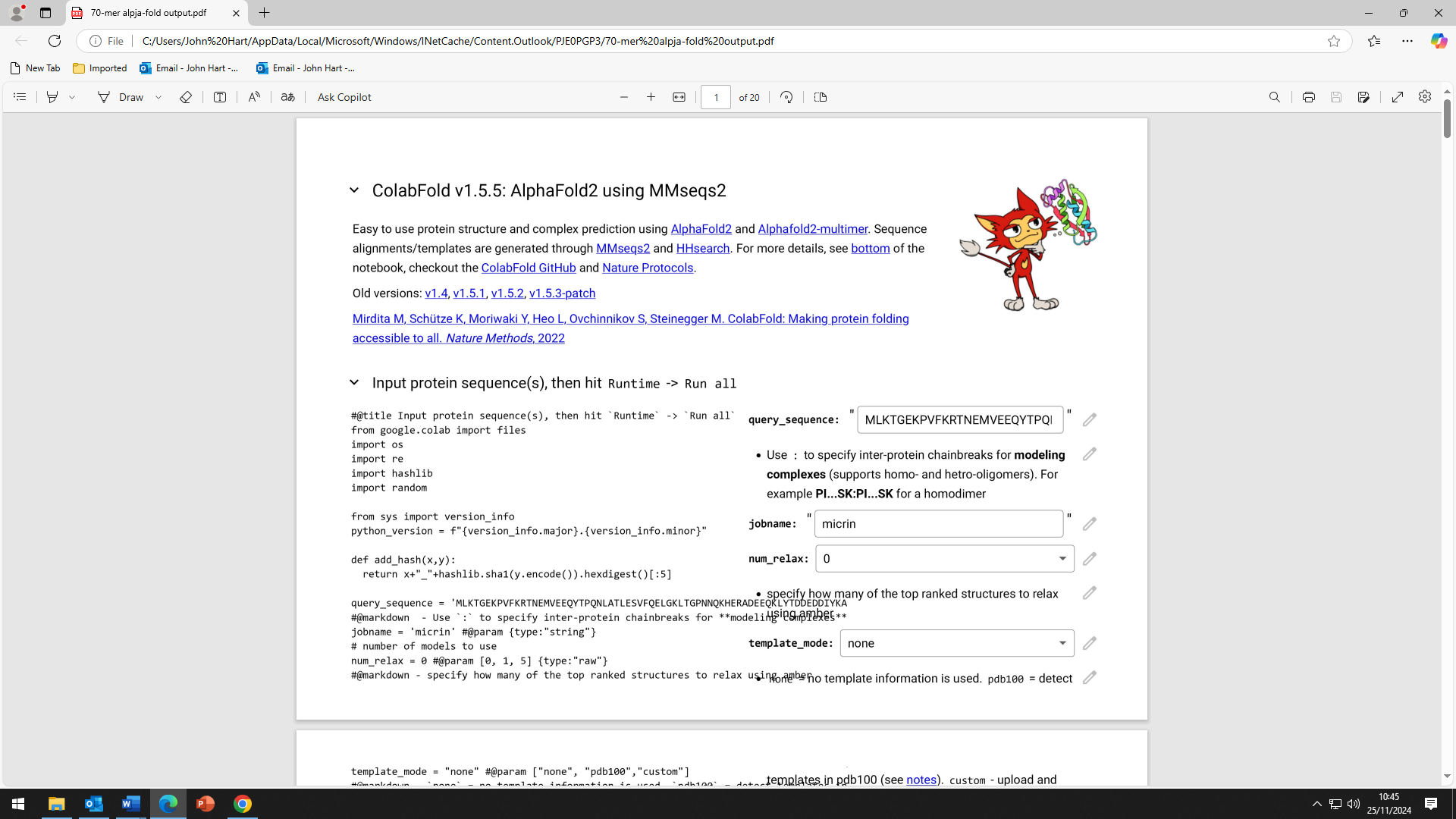


**S5 Figure 9.** AlphaFold2 data input portal for sSgII-70 sequence.


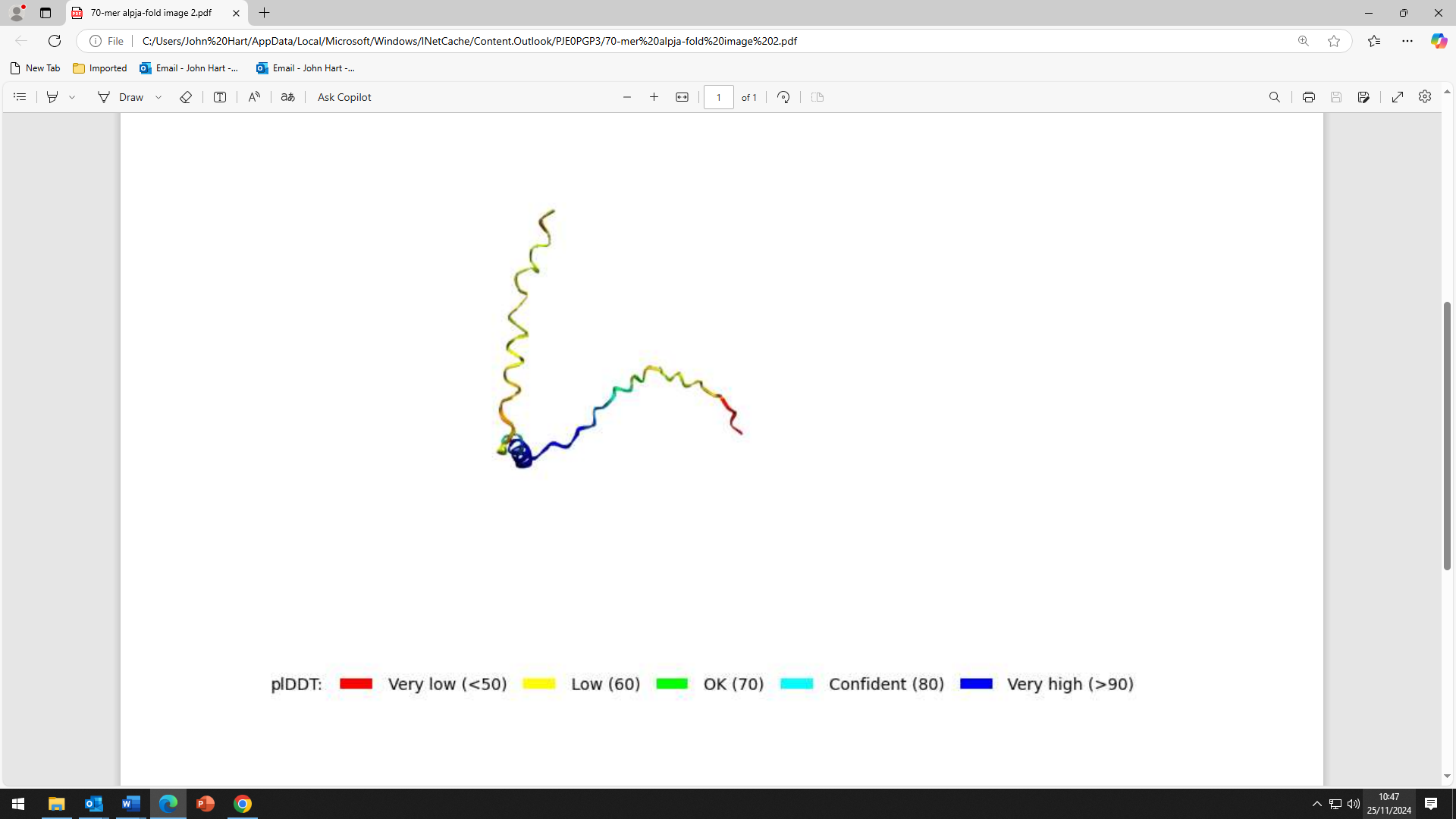


**S5 Figure 10.** AlphaFold2 sSgII-70 Bezier ribbon.

N terminus at left, C terminus at right. pLDDT = predicted Local Distance Difference Test, a per-residue measure of local confidence. It is scaled from 0 to 100 (red to blue on a spectrum), with higher scores indicating higher confidence and usually a more accurate prediction.

Three helical regions are identified potentially for sSgII-70 in the paper’s Fig. 11. These are provided by PSIPRED, a dedicated helix prediction program. These three helical motifs find echoes in the Bezier ribbon model of S5 Fig. 1, in terms of matching molecular curlicues. Only the central helix prediction is supported by AlphaFold2, with a helical region highly predicted mid-molecule. This is in line with the paper’s structural prediction for sSgII-70 of spiralisation. The other prediction is juxtaposed termini. Neither the main modelling software from ChemSW nor AlphaFold2 deliver end adjacency.


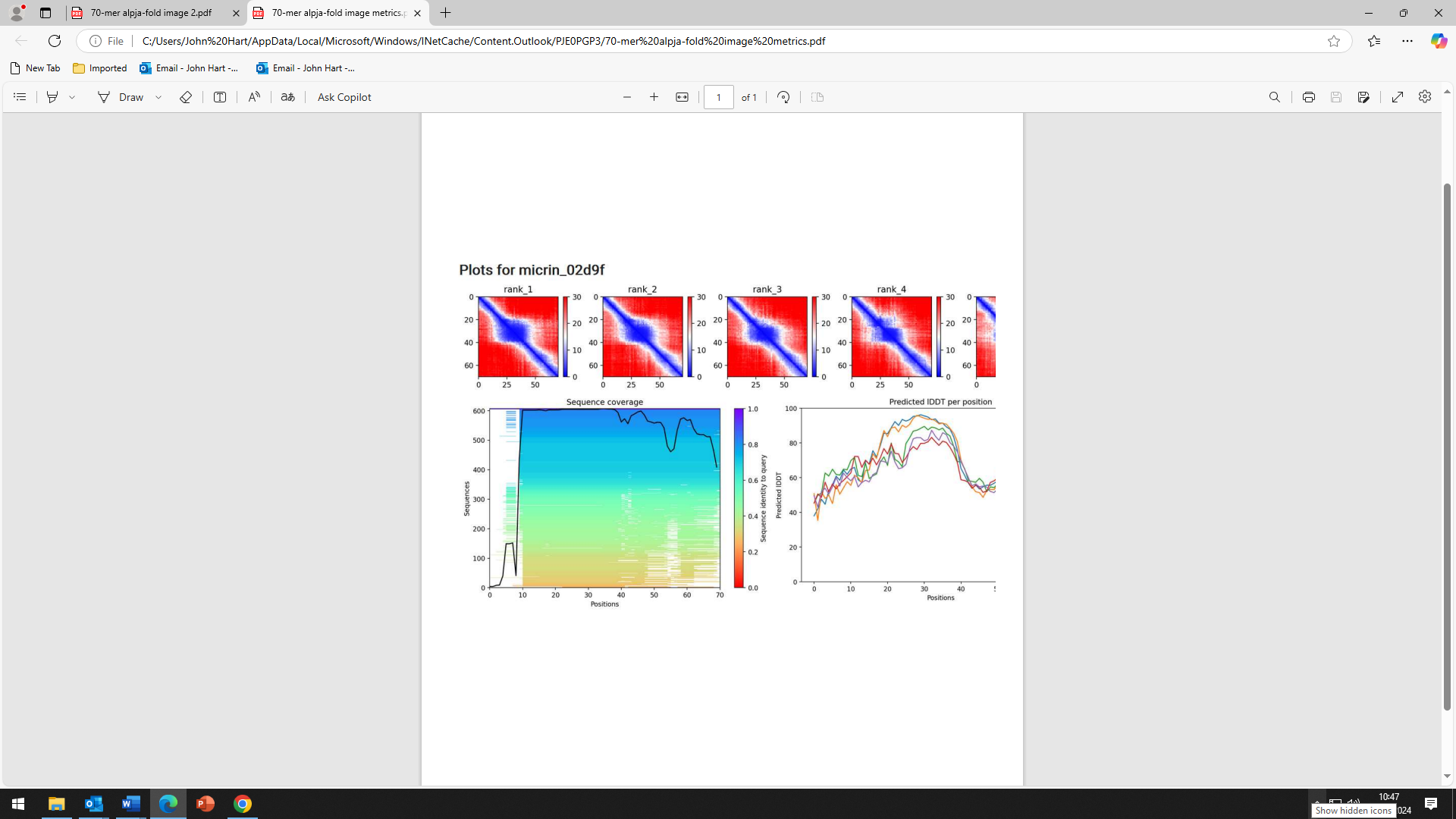


**S5 Figure 11.** AlphaFold2 metrics for sSgII-70. (Data exactly as supplied.)

[ENDS]
