# Supplementary material for: Detecting the body’s reproductive hormonal brake against tissue overgrowth: Micrin/SgII-70
Source: PLoS One. 2026 Mar 2;21(3):e0340980. doi: 10.1371/journal.pone.0340980 (PMC12952585; doi:10.1371/journal.pone.0340980)
Supplement: S6 File — https://doi.org/10.6084/m9.figshare.27110332.v2. This project provides a discussion of possible chains of logic and associated theoretical frameworks which can be associated with and possibly explain the generated organotrophic data and MALDI analyses. (DOCX) [file pone.0340980.s006.docx]

**Supplementary Information 6 (S6)**

**Extended Discussion**

S6 is provided in support of ‘Detecting the body’s reproductive hormonal brake against tissue overgrowth: micrin/SgII-70’ by Hart JE, Davies KG, Mundy CR, Hart AC, Howlett DR & Newton RP (2024). Corresponding author email: [k.davies@herts.ac.uk](mailto:k.davies@herts.ac.uk)

| **S6 Table 1** | | |
| --- | --- | --- |
| **Chain of logic** | | **Theoretical framework** |
| 1 | Testes secrete water soluble (i.e. nonsteroidal) pituitary-inhibiting hormone, ‘inhibin’, whose lack is responsible for post-castration pituitary enlargement (McCullagh, 1932). Gonadal inhibitor of female mammalian tissue masses, including pituitary, recognised in form of ‘enigmatic ovarian influence’ (Hart, 1990a). Hypothesis of the body’s reproductive hormonal brake against tissue overgrowth, ‘micrin’ (Hart, 2014). First articulation of H1 (Hart et al, 2017). | Hypothesis H1 is that an inhibitory gonadal influence on tissue masses IS accounted for by the factors described (Candidate 7500, EPL001 & SgII-70). Hypothesis H2 is that an inhibitory gonadal influence on tissue masses IS NOT accounted for by the factors described (Candidate 7500, EPL001 & SgII-70).  These hypotheses have been broken down into independent subhypotheses, as below. |
| 2 | Inhibitory gonadal influence in ovarian follicular fluid deduced to be due to proteinaceous multi-peak MALDI-TOF MS ‘Candidate 7500’ (range of peaks *m/z* 7-8000, ~70 aa, also gel band at ~7.5 kDa), based on organometric bioassay-guided fractionation and antiorganotrophic activity correlations in vivo (including seasonal and cyclic) and in vitro (sheep to rat = cross-species exemplifications). Elimination of 11 other candidate molecules. Candidate 7500 heterogeneity suggested to be due to fragmentation in MS. Candidate 7500 anionex fractions (female) inhibit compensatory renal growth (crg) in the unilaterally nephrectomised (UNx) male rat (= cross-sex activity). These fractions also anti-uterotrophic in a rat bioassay in vivo. Material (notably OVX) lacking Candidate 7500 is inactive in vitro. | Subhypothesis SH1 is that an inhibitory gonadal influence on tissue mass IS related to Candidate 7500. The alternative Subhypothesis SH2 is that an inhibitory gonadal influence on tissue masses IS NOT related to Candidate 7500.  No findings discount SH1. The evidence is judged  more supportive of SH1 than SH2. SH2 is rejected. |
| 3 | Edman degradation of Candidate 7500 gel material yields 7 related Edman N-terminal sequences (S1 Table 1). Bioinformatics, molecular biology and tryptic digestion (S4) unproductive for canonical ‘EPL001’ sequence, which if related to Candidate 7500 would be a 20% N-terminal fragment of it. EPL001 as a synthetic 14mer peptide inhibits (dose dependently) crg in the UNx rat (Haylor et al, 2009), in accordance with predictions for the inhibitory factor in its entirety (Hart, 2014), as do Candidate 7500 anionex fractions. EPL001 peptide and Candidate 7500 fractions both *inhibitory* in vitro, producing fewer smaller cells. Western blots show the circulating endogenous antigen of an anti-EPL001 antibody is OV+/OVX–, like Candidate 7500 (Hart, 2008), and such an antibody visualises early and late gel bands in OV+ anionex fractions in the same pattern as Candidate 7500 (S1 Fig. 4). | Subhypothesis SH3 is that EPL001 IS related to Candidate 7500. The alternative Subhypothesis SH4 is that EPL001 IS NOT related to Candidate 7500. |
| 4 | EPL001 detected via 4-residue partial sequence (Beale 4) in ovarian follicular fluid, where also is found Candidate 7500. Both are gonadal. FITC-EPL001 localises only to presumptive ovarian tissue in genital tract of *C. elegans* (Davies & Hart, 2008). If EPL001 and Candidate 7500 are both related to the inhibitory gonadal influence then there are receptors for the hormone in the worm ovary (= cross-phylum deduction). | No findings discount SH3. The evidence is judged  more supportive of SH3 than SH4. SH4 is rejected. |
| 5 | An anti-EPL001 antibody exaggerates crg in the UNx rat, suggesting that an endogenous brake has been neutralised in an IGF-1 model system (Haylor et al, 2009). Immunoneutralisation (IN) is also achieved in vitro (Hart et al, 2017). Westerns visualise gel bands at ~7.5 kDa. Immunohistochemistry (IHC) provides mammalian and fruit fly images of apparent neuroendocrine relevance (Hart, 2017), with the former granin-like (= cross-phylum correlation). An anti-EPL001 antibody in an immunoprecipitation protocoal with liquid chromatography-mass spectrometry (IP/LC-MS) and tryptic digestion, suggests endogenous antigen is ‘likely secretogranin II (SgII) related’ (Hart et al, 2017). The fruit fly endogenous antigen of the anti-EPL001 antibody in IP/LC-MS, Q9W2X8, has SgII homology in detail (Hart et al, 2017) (= cross-phylum correlation). The assertion that EPL001 is encrypted within the neuroendocrine prohormone sSgII delivers a swathe of potential insights (S6 Table 2), notably an active face hormonal analysis for design of hexapeptide agonist and antagonist mimetics (Hart et al, 2022). | Subhypothesis SH5 is that EPL001 IS related to SgII. The alternative Subhypothesis SH6 is that EPL001 IS NOT related to SgII.  No findings discount SH5. The evidence is judged more supportive of SH5 than SH6. SH6 is rejected. |
| 6 | As between aa sequences there is an anagrammatical relationship: MKPLTGKVKEFNNI (EPL001) and MLKTGEKPV∙FK∙NNI (‘sSgII-14’). This is claimed to be meaningful, EPL001, as arising from a secreted proteoform of sSgII of ~70-residues (‘sSgII-70’), because the Edman machine read the latter nonsequentially due to target molecule stepwise spiralised depolymerisation in the machine’s reaction chamber. Spiral reading paths have been discerned (S1 Figs. 1 & 2). The EPL001 Edman reading is a spatial mapping of the target molecule, such that an anti-EPL001 antibody sees the sSgII proteoform target molecule. | Subhypothesis SH7 is that there IS a secreted proteoform of SgII in the form of sSgII-70. The alternative Subhypothesis SH8 is that there IS NOT a secreted proteoform of SgII in the form of sSgII-70. |
| 7 | Granted a meaningful anagrammatical relationship, the elements within sSgII (MLKTGEKPV + FK + NNI) can be used to deduce the full primary structure of sSgII-70, given two further assumptions: (i) that an N-terminal methionine is needed as the Edman starting point, as this is the initial residue of EPL001; and (ii) a C-terminal NNI is required for recognition by the anti-EPL001 antibody of sSgII-70 on the basis of the potential recognition of a discontinuous endogenous SgII-related epitope (Howlett et al, 2019). | Ditto |
| 8 | The proposed primary structure of sSgII-70 surprises by implying a 70mer product of reverse peptide splicing, i.e. 9+61 (the paper’s Fig. 9). Such have been seen in proteasome production of surface antigens for immune surveillance, while hybrid peptide surface antigens have been reported from pancreatic secretory vesicles of sequence elements from insulin and unrelated proteins such as chromagranin A. | Ditto |
| 9 | A grid of EPL001 and sequence elements in sSgII predicts the shape of sSgII-9 (MLKTGEKPV) as modelled in silico (S1 Fig. 3). The 14x14 grid implies that the shuffled sequence motifs are juxtaposed in space as 9(MLKTGEKPV) + 2(FK) + 3(NNI). Epitope and active face analyses also predict adjacent termini. Covalent crosslinks rendered unlikely by molecular modelling (S5). Stabilisation via salt bridging likelier, with associated hydrogen bonding, in the form D/E^Q^K/R. | Ditto |
| 10. | The inhibitory gonadal hormone concept includes suppressed reproduction. Yet in *C. elegans* EPL001 is *pro-fecundity* (Davies & Hart, 2008). In this system, EPL001 blocks an endogenous inhibitor, according to an analysis of bimodular tri-residue (**MKP**, **VFN**) topology based on sSgII-70. (EPL001 in *C. elegans* increases lifespan, indicating an influence on fundamental processes.) EPL001 via intracerebroventricular infusion in sheep elevates peripheral GH and PRL, but so does a scrambled-sequence control, EPL030. Both peptides yield to a structure-activity analysis based on using sSgII-70 as a decryption key. | No findings discount SH7. The evidence is judged more supportive of SH7 than SH8. SH8 is rejected. |
| 11 | Granins deliver stoichiometric dose-response curves in cell assays: Candidate 7500 anionex fractions ditto (S2). Existence of sSgII-70 can account for MS and gel Candidate 7500, a polypeptide of ~70 aa. sSgII-70 predicted to be acidic; Candidate 7500 has granin-like acidic purification (anionex) characteristics. Candidate 7500 MS tryptic digest data are supportive of the 9+61 model, providing integer peak matches to trypsinisation predictions in silico for sSgII-70, with an integer match crossing the 9/61 boundary at *m/z* 906 (which is OV+/OVX–, like Candidate 7500 and the circulating antigen of an anti-EPL001 antibody). Ovine Candidate 7500 data fitting provides a picture of MS artefactual C-terminal truncation and water losses, together with (MS and storage) artefactual C-terminal Grand Fragment losses and the production due to dimerization of Superheavyweights. MS data fitting in the bovine yields the same results for bSgII-70 (= interspecies correlation). | Subhypothesis SH9 is that Candidate 7500 IS sSgII-70. The alternative Subhypothesis SH10 is that Candidate 7500 IS NOT sSgII-70.  No findings discount SH9. The evidence is judged more supportive of SH9 than SH10. SH10 is rejected. |
| 12 | The totality of evidence (Figs. 8 & 10) unites Candidate 7500, EPL001 & SgII-70 with the concept of an inhibitory gonadal hormone suppressing tissue overgrowth and curbing reproduction. The putative effector, SgII-70, is an acidic 70mer polypeptide hairpin stabilised and spiralised by salt bridging with hydrogen bonding, potentially explaining relative resistance to trypsin and heat, high solubility in water, purification amphipathicity, co-crystallization readiness in MALDI-TOF MS and Edman waywardness. Binding in full to a receptor conceptualised as dimeric is posited to involve both ends of the hairpin together (Hart et al, 2022). Short peptides based on sSgII-70’s deduced active face display inhibitory bioactivity in vivo and in vitro (Hart et al, 2022), in line with similar displays of activity by Candidate 7500 fractions and the synthetic peptide EPL001. | Hypothesis H1 is that an inhibitory gonadal influence on tissue masses IS accounted for by the factors described (Candidate 7500, EPL001 & SgII-70). The alternative Hypothesis H2 is that an inhibitory gonadal influence on tissue masses IS NOT accounted for by the factors described (Candidate 7500, EPL001 & SgII-70).  No findings discount H1. The evidence is judged more supportive of H1 than H2. H2 is rejected. |

| **S6 Table 2** | |
| --- | --- |
| **Insights if EPL001 is encrypted within sSgII** | |
| 1 | Explanation of the unproductivity of bioinformatics and molecular biology in terms of the Edman-derived EPL001 sequence MKPLTGKVKEFNNI being a shuffle of discontinuous aa sequence elements within sSgII: MLKTGEKPV (= sSgII-9) + FK + NNI (= sSgII-14, synthesized as EPL143, as described in Hart et al, 2022). EPL001 is an Edman misread of a novel hormonal proteoform of sSgII, called here sSgII-70. |
| 2 | Description of an endogenous aa sequence that at least roughly matches EPL001, a minimal requirement for any hypothesis purporting to explain a mysterious Edman-derived N-terminal sequence with an initial methionine. The endogenous match is sSgII-70, a proposed 70mer derivative of SgII, with its N-terminal region deriving from sSgII’s second sorting domain and having the methionine of this as its N terminus. sSgII-70 solves the EPL001 identification puzzle (Fig. 8). |
| 3 | Comprehension of MALDI-TOF MS findings in detail, including of tryptic digests (notably a fragment at *m/z* 906), in terms of the proposal that sSgII-70 comprises (i) a 61mer from the most evolutionarily conserved region of SgII and (ii) a 9mer C-terminal of the 61mer, sSgII-9, from SgII’s second main sorting domain (Fig. 9). The pair are reverse peptide spliced, 9+61, from a possible SgII sorting super-domain. MS datafitting using a C-terminal truncation model provides confirmation of the C-terminal aa sequence for sSgII-70 & bSgII-70. |
| 4 | Provision of a structural prediction for sSgII-9, derived from an sSgII-14/EPL001 14x14 sequence grid and upheld by molecular modelling (S1 Fig. 3). |
| 5 | Deduction of sSgII-70’s active hormonal face as **M∙KPVF∙N**, with that of human and rat being **M∙KPNF∙N**. These realizations have permitted the synthesis of short-peptide mimetics for studies of bioactivity (Hart et al, 2022). |
| 6 | Discernment of why EPL001 is anti-proliferative, in line with the inhibitory hormone hypothesis, and why substituting alanine at either end of the peptide or in the middle does not abolish the inhibition in vitro (Hart et al, 2022). The active face sextet in sSgII-70 is present in EPL001 thus: **MKP**xxxx**V**xx**FN**xx. |
| 7 | Explication of the anionex Beale 4 residues xxx**P**xxxx**V**xx**FN**xx (S1 Table 1), as partially sequential in sSgII-70 (**PVF**, Fig. 10) and contiguous in space **PVF∙N** (S1 Fig. 3). Also, insight into subsidiary readings at positions 8 & 11 (V/L & F/K) as being due to the residues on offer during particular cycles of Edman Nonsequentialism (Hart et al, 2022). |
| 8 | Analysis of anomalous activity (anti-proliferative, anti-fecundity) of EPL030, a scrambled-sequence 14mer *control* peptide for EPL001, in terms of its having by chance in its sequence three of the Beale 4 (SEQ ID NO: 4, S1 Table 1) active face residues together and an appropriately spaced K-P doubleton: KL**K**MNGKNIE**PVF**T. Bioactivity is thus explained of an anagram (EPL030) of an anagram (EPL001) of sSgII-14 (= EPL143). |
| 9 | Molecular description of reproductive activation in vivo by **IEPVFT** (EPL036), the tail end of control 14mer peptide EPL030. Involved is *antagonism* of an endogenous reproductive inhibitor at a fecundity receptor, on the basis of EPL036’s possession of part of sSgII-70’s active face, **PVF**. |
| 10 | Assessment of the endogenous epitope of the anti-EPL001 G530 antibody as the SgII-related contiguous and non-contiguous **KE∙F∙NNI** (Howlett et al, 2019). There is an interspecies immune-MW correlation (Hart et al, 2017). |
| 11 | Predictions in regard to Q9W2X8, the fruit fly IP/LC-MS antigen of the anti-EPL001 G530 antibody, of its epitope as **K∙E∙F∙NNI** and the active site of a potential secreted form as **M∙K∙PV∙F∙N**, both by analogy with sSgII-70. |
| 12 | Illumination of why dissimilar peptides produce highly similar expression profiles for differentiation marker genes and core circadian clock genes: EPL001 (**MKPLTGKVKEFNNI**) & EPL140 (**MLKTGEKPNKFNNI** = hSgII-9 + EPL001’s K & FNNI) (Hart et at, 2022). Closer to SgII-70, the EPL140s are more potently cell-inhibitory than EPL001, but they share with it SgII-relatedness, reducing to molecular practice the inhibitory hormone hypothesis. |

Micrin is predicted to be an organic acid and water soluble (Hart, 2014). sSgII-70 is likewise predicted to be acidic and hence water soluble. Taking into account the evidence pointing to ‘likely SgII relatedness’ and other evidence marshalled in the paper and summarised in S6 Tables 1 & 2, the following co-identifications are supported: **Candidate 7500 = EPL001 = sSgII-70 = endogenous inhibitory antigen of the anti-EPL001 antibodies = the postulated micrin**.

Immunohistochemistry implies the presence of micrin centrally. Hypothalamic micrin in particular is conceived of as acting locally, rather than circulating systemically to remote effect. The subjacent tissue of interest is of course the anterior pituitary, provider of plasma LH, prolactin and GH, among other hormones. Intracerebroventricular infusion of EPL001 in sheep was associated with elevated growth hormone in peripheral blood and reduced prolactin (Hart et al, 2022). Immunostaining with anti-EPL001 antibodies was apparent in individual neurons in the ovine lateral and ventromedial hypothalamus and preoptic area, for example, with heavy staining in the palisade (neuroendocrine) region of the median eminence, with axonal beading betokening transport (Hart et al, 2017, p11). Ovariectomy did not affect positive immunostaining in the ovine median eminence (Hart et al, 2017, Supplementary IHC Images therein), rendering moot any gonadal influence on hypothalamic micrin production. Hypothalamic staining in the rat was evident in the arcuate nucleus, with individual neurons staining in the retrochiasmatic nucleus. Staining was not seen in either the sheep or rat pituitary. It can be proposed that hypothalamic micrin/SgII-70 modulates hypothalamic releasing hormones in a localized paracrine manner, while also travelling to the pituitary via the median eminence to modulate anterior hypophyseal function in an endocrine fashion. Hypophysectomy of sheep did not affect the detection in blood samples of inhibitory activity using the assay in vitro involving rat bone marrow stem cells. This is consistent with the absence from the systemic circulation of hypothalamic micrin.

If not from the hypothalamus, what is the provenance of circulating micrin? The kidney studies can be cited once more. Candidate 7500 suppresses compensatory renal growth after unilaterial nephrectomy in the rat (Hart, 2001). EPL001 does likewise (Haylor et al, 2009). Meanwhile, an anti-EPL001 antibody administered on its own exaggerates compensatory renal growth (op. cit.). This is consistent with the immunoneutralisation of a circulating inhibitor. Whence this inhibitor? It is hypothesized that micrin circulating in blood is derived from the ovary and testis (Hart, 2014). In IHC using anti-EPL001 antibodies the rat ovary showed moderate staining in theca, granulosa, follicle cells and follicular fluid (Hart et al, 2017). Sheep plasma in one series was subject to the standard purification procedure involving anion exchange chromatography (Hart, 2003). In six separate fractionations involving ovarian venous plasma Candidate 7500 was seen in MALDI-TOF MS. Candidate 7500 was not seen in jugular vein plasma from two ovariectomised ewes, supporting a gonadal provenance. Likewise the circulating antigen to the anti-EPL001 antibodies is OVX–, i.e. lacking from the purified plasma of ovariectomised sheep. When samples lacked Candidate 7500, including by dint of ovariectomy, inhibitory activity was not observed in the assay in vitro involving rat bone marrow stem cells. The assumption here is that what applies to females holds for males: that circulating micrin is gonadal in origin.

Having accounted for hypothalamic micrin (modulation of pituitary hormones) and gonadal micrin (provision of circulating factor), what of the immunostaining in other tissues? Focal neuroendocrine cell staining was seen for example in human pancreas, stomach, duodenum, small bowel, appendix, colon, rectum and prostate; staining was also judged strongly positive in human salivary gland ducts and mouse placenta; and individual neurons stained in the rat cerebral cortex (pyramidal cells, layer 5) and in the sheep thalamus and diagonal band of Broca (Hart et al, 2017). Paracrine functionality and neuromodulation are available speculations.

Micrin is believed to be induced by antioestrogens and this probably partly explains the efficacy of tamoxifen in breast cancer treatment (Hart, 2014). Pituitary SgII is downregulated by oestradiol (Anouar & Duval, 1992), rendering it possible that SgII-70 is induced by antioestrogen. The provision of an sSgII-70 related hexamer peptide, **IEPVFT**, a putative *antagonist*, for use in reproductive activation (Davies et al, 2015), is of interest as it is a prediction of the micrin hypothesis that an antagonist of the hypothesized hormone might be a useful treatment in infertility.

There is enduring perplexity surrounding the initiation of puberty, notwithstanding progress in understanding kisspeptin and other relevant actors (Ezzat et al, 2015; Livadas & Chrousos, 2016). In the context of a postulated lifting at this time of a micrin brake as part of a somatic cueing system, the arcuate nucleus of the hypothalamus is of interest (Hart, 2014, p783) as is the median eminence (Ezzat et al, 2015). Meanwhile the germinal epithelium of the testis is of theoretical importance as a potential site of secretion (Hart 2014, p783) in the context for example of (a) benign prostatic hyperplasia being viewed as a micrin deficiency disorder involving insufficiently opposed androgen and (b) a rise in the incidence of neoplasms with age, the latter a reflection of systemic decline in inhibition within the body-wide organotrophic system (op. cit., p784). In the SgII-relatedness study IHC staining was seen in both the arcuate nucleus and median eminence and in the testicular germinal epithelium (also spermatogonia), without appearing in the testicular interstitial Leydig cells, whence testosterone (Hart et al, 2017, Fig. 3 therein). (Staining was also seen in the ovary: see below.) Consonantly, the SgII gene *Scg2* is expressed in rat brain and testes, as well as the adrenals, as judged by RNA transcription, with data unresolved for cell types within organs (NCBI Gene ID 24765). SgII expression is lacking in heart, kidney, liver, lung, muscle, spleen and thymus. Testicular SgII gene expression declines sharply with age (op. cit.). Micrin is predicted to fall with age in the male, leading to ballooning of the prostate in older rats and men (Hart, 2014, p784). Co-identification of sSgII-70 and micrin implies that organ mass sensing at puberty and other times could involve a stoichiometric counting process.

The conclusion in the paper from organometric assays in vivo in the rat, using ovine material, was as follows: ‘Antihypophysiotrophic and broadly antiorganotrophic, micrin is yet adrenotrophic’. Both Candidate 7500 and the endogenous antigen to the anti-EPL001 antibodies display an adrenal association. The antigen is upregulated in human pheochromocytoma, an adrenal tumour, as judged by IHC, also in adrenal cortical carcinoma. Immunoneutralisation was achieved in regard to an anti-proliferative and pro-apoptotic influence of PC12 (rat pheochromocytoma cell) conditioned medium on rat bone marrow cells in culture (Hart et al, 2017). Consider the aplastic anaemia induced in dogs and ferrets by exogenous oestrogens, not seen in rodents or humans (Hart, 1990a). A phase of bone marrow stimulation is superseded by lethal depression, with associated haematological changes, due in one model to runaway sequential changes in the pituitary-adrenal axis (Hart, 2014). Haematopoietic suppression does not occur in the rat because it is arrested adaptively by micrin, in this view, which though generally anti-organotrophic is also adrenotrophic, sparing the bone marrow. The oestrogen-treated rat could presumably be made to display ferret-like vulnerability by co-administering with oestrogen an anti-EPL001 antibody to immunoneutralise gonadal rSgII-70. Such SgII-70 studies could shed light on an oestrogen-related species differences that is one of the longest-standing enigmas in endocrine pathology. Other progress beckons, including a concept of cancer that can include organotrophic system attenuation, viz. a reduction in micrin braking.


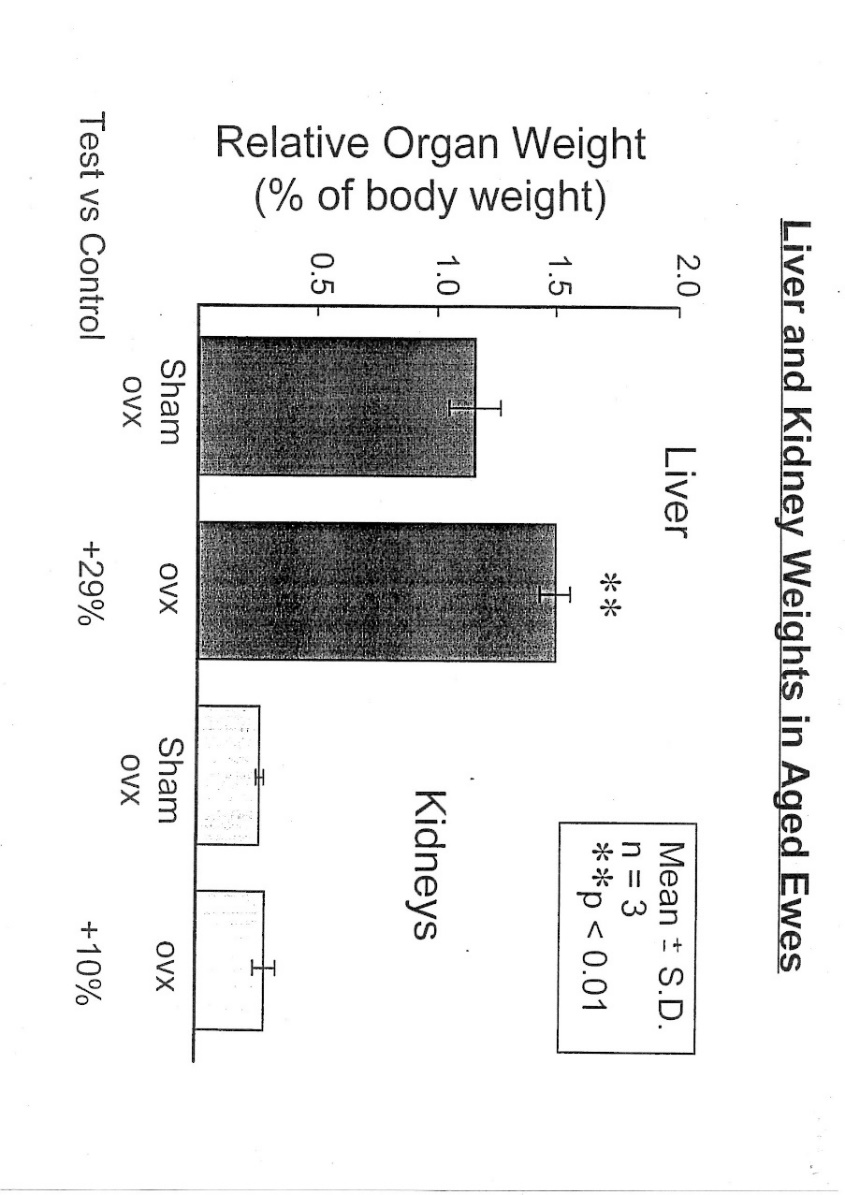


**S6 Figure 1.** Liver and kidney weights of otherwise untreated old female sheep subject to ovariectomy (18 months before study termination) or sham ovariectomy

Explaining the above figure, there is here proffered a quotation from Hart 2014, pp. 784-5, with references and cross-references omitted:

‘In the young adult female rat gonadectomy decreases the relative size of the liver, for example, and to a lesser extent that of the kidneys, within a generally antiorganotrophic picture, which includes pituitary shrinkage. The loss of a net positive ovarian influence is indicated, implying the predominance of oestrogen (and testosterone) over micrin in young adult female rats. To investigate the response to ovariectomy in older female mammals the author undertook a study with colleagues in aged sheep at Harwell Laboratory, UK. Six multiparous non-pregnant ewes of 7–13 years were assigned semi-randomly to two groups, such that the mean ages and body weights of the two groups were similar. One group was subject to surgical ovariectomy, the other to sham ovariectomy. At termination 18 months later the mean relative kidney weights (% body weight) of the ovariectomized sheep were a non-significant 10% higher than those of sham operated controls, while the mean relative liver weights were 29% above those of controls (P < 0.01). The removal of antiorganotrophic ovaries was indicated.

The mammalian ovary thus may progress from being a net organotrophic influence in young adults to a net antiorganotrophic influence when old. This implies that a micrin:sex steroids ratio favouring sex steroid in females is superseded by one favouring micrin. This is in contrast to the declining ratio of micrin:sex steroids inferred for older males. Older women appear to be getting more endogenous micrin protection against tissue overgrowth than older men and, conjecturing, perhaps this ‘micrin plus’ status helps to keep them alive longer than their men folk. Some men are regarded as worth treating with testosterone, as having too little, but almost all men are ‘micrin minus’, with an expanding prostate as the prime indicator. So as well as shrinking the prostate, supplementary micrin might boost male longevity, narrowing the sex gap in lifespan. In absolute terms the body’s brake may weaken in both sexes over time, contributing to a rise in cancers with age.’

**REFERENCES UNIQUE TO S6**

Anouar Y & Duval J (1992) Direct estradiol down-regulation of secretogranin II and chromogranin A mRNA levels in rat pituitary cells. *Molecular and Cellular Endocrinology*, **88**, 97-104

Ezzat A, Pereira A & Clarke IJ (2015) Kisspeptin is a component of the pulse generator for GnRH secretion in female sheep but not the pulse generator. *Endocrinology*, **156**, 1828-1837

Livadas S & Chrousos GP (2016) Control of the onset of puberty. *Current Opinion in Pediatrics*, **28**, 551-558

[ENDS]
